# Edentulism in China and the Group of Twenty (G20): Epidemiological Trends, Decomposition Analysis, and Forecasts to 2038

**DOI:** 10.1016/j.identj.2026.109692

**Published:** 2026-06-16

**Authors:** Yilin Zhang, Lei Zhao, Xiao Shuang Xu, Haocheng Wang, Fei He, Yuanyuan Liu, Jing Li, Xin Xu

**Affiliations:** a School of Stomatology, Shandong Second Medical University, Weifang 261053, Shandong, China; b Affiliated Hospital of Shandong Second Medical University, Weifang 261053, Shandong Province, China; c Rizhao Lanshan District People's Hospital, Rizhao 276800, Shandong, China; d Hospital of Stomatology Xi'an Jiao Tong University, Xian 710004, Shanxi, China; e School of Public Health, Shandong Second Medical University, Weifang, 261053 Shandong Province, China

**Keywords:** Edentulism, Public health, Disability-adjusted life years, Epidemiology, Disease burden

## Abstract

**Aims:**

Edentulism is a severe global oral health burden, particularly in aging societies. While previous studies have documented epidemiological trends, a direct comparison of the drivers and future trajectories between a rapidly aging economy like China and the broader Group of Twenty (G20) nations are lacking. This study aimed to compare temporal trends, decompose contributing factors, and forecast the future burden of edentulism in China versus the G20 aggregate from 1990 to 2038.

**Methods:**

Using Global Burden of Disease 2023 data, we analysed age-standardised incidence rates (ASIR) and disability-adjusted life years (DALYs) for edentulism. We employed Joinpoint regression to assess temporal trends (1990-2023), and demographic decomposition analysis to quantify contributions from population growth, aging, and epidemiological change, and Autoregressive Integrated Moving Average models to forecast burden through 2038.

**Results:**

From 1990 to 2023, China’s ASIR declined modestly by 5.9% and DALYs by 11.0%, lagging behind reductions achieved by top-performing G20 nations. Decomposition analysis revealed a fundamental divergence in drivers: population aging accounted for over 80% of the net increase in China’s burden, whereas population growth was the predominant contributor (78.7%) for the G20 overall. ARIMA forecasts indicate that China’s burden will experience a brief decline followed by a sustained rebound, stabilizing at a level higher than the G20 average.

**Conclusion:**

China’s edentulism burden is predominantly driven by population aging, contrasting with the G20’s reliance on population growth as the primary driver. Future trajectories suggest China will face greater long-term oral health pressures than the G20 average.

**Clinical Relevance:**

Oral health strategies in China must pivot from generic prevention toward age-specific interventions. Integrating comprehensive oral healthcare into geriatric medicine and national chronic disease frameworks is imperative to mitigate the escalating burden driven by an unprecedented aging population.

## Introduction

Edentulism is a prevalent global oral disease defined as the complete loss of all natural teeth, excluding infants with unerupted teeth.^1^ It is a devastating and irreversible condition and has been described as the ultimate indicator of the burden of oral health.^2^ Chronic oral diseases such as periodontitis and dental caries are the primary contributing factors to edentulism.^3^^,^^4^ Although not directly life-threatening, tooth loss is associated with an increased risk of mortality.^5^ Edentulism significantly impacts both physical and psychological health, affecting facial appearance, masticatory function, social interaction, and self-esteem.^6^ Epidemiological surveillance by the World Health Organization (WHO) indicates that the global prevalence of edentulism increases markedly with age – approximately 7% among individuals aged 20 years and older, rising to as high as 23% in those aged 60 years and above. In 2019, an estimated 35.2 million people suffered from complete tooth loss.^7^

The G20 is a premier forum for international economic cooperation, comprising the world's major advanced and emerging economies. Previous studies have indicated that the burden of edentulism is significantly higher in regions with high and middle Sociodemographic Index (SDI), whereas it remains relatively lower in low-SDI regions.^8^ As the most populous nation within the G20 and undergoing rapid socioeconomic transition and population aging, China's edentulism burden warrants in-depth investigation.^9^

Understanding the edentulism burden is not merely a dental concern but a public health imperative. Complete tooth loss is associated with increased mortality, cognitive decline, and reduced quality of life, imposing substantial costs on healthcare systems.^10^ In aging societies, the number of older adults at risk is rising rapidly, yet many countries lack integrated oral health strategies within their chronic disease and elderly care frameworks.^11^ China, facing the world’s largest aging population, provides a critical case study. By comparing China with G20 countries at different development stages, this study identifies whether the drivers of edentulism burden differ between rapidly aging economies and those driven primarily by population growth.^12^ Such evidence can guide resource allocation, inform preventive priorities (eg, targeting aging versus population expansion), and support the integration of oral health into universal health coverage and healthy aging agendas – key goals of the WHO Global Oral Health Action Plan 2023 to 2030.^13^

Based on the GBD 2023 dataset, this study comprises a comparative analysis of edentulism between China and G20 countries with three primary objectives: (1) to examine epidemiological characteristics and temporal trends from 1990 to 2023; (2) to decompose the driving factors (population growth, aging, and epidemiological change) contributing to the net change in burden; and (3) to forecast future trajectories through 2038. This analysis aims to inform resource allocation and policy development for oral health within an aging population framework.

## Methods

### Data sources and study design

All analyses were performed using age‑standardised rates. Ethical approval was not required for this study, as it relied exclusively on publicly available, anonymised data from the GBD 2023. It should be noted that GBD estimates are derived from statistical modelling based on available primary data sources (eg, vital registration, surveys, hospital records), rather than directly observed data. The GBD 2023 offers comprehensive estimates for 375 diseases and injuries across 204 countries and territories. Edentulism was defined using ICD-10 code K08.2.^14^ We extracted age-standardised estimates of incidence and DALYs for China and all G20 member countries from 1990 to 2023. In the GBD framework, the incidence of edentulism refers to the first transition from having any natural teeth to complete tooth loss – a cumulative, irreversible event.^15^ This definition differs from the incidence of acute or recurrent diseases. Incidence was prioritized over prevalence in this study to capture the rate of new transitions into edentulism, which is more informative for primary prevention strategies and trend analysis of disease progression.^16^ All extracted estimates are age‑standardised rates (per 100,000 population), not crude counts, unless otherwise specified. All data and estimation methodologies are publicly accessible through the GBD 2023 repository: https://ghdx.healthdata.org/gbd-2023.^17^

All 20 members of the G20 were included – the 19 sovereign nations (Argentina, Australia, Brazil, Canada, China, France, Germany, India, Indonesia, Italy, Japan, Mexico, the Republic of Korea, Russia, Saudi Arabia, South Africa, Turkey, the United Kingdom, and the United States) and the European Union (EU) as the 20th member, for which aggregate burden estimates from the GBD 2023 study for the EU region were used, with no entities excluded.^18^ The full time range from 1990 to 2023 was covered. Incidence and DALYs were selected as the primary burden metrics, while YLL was not analysed because it is considered negligible for periodontal disease in the GBD framework.

### Statistical analysis

#### Joinpoint regression model

We employed joinpoint regression to characterize temporal trends and detect significant breakpoints in age‑standardised edentulism rates for China and G20 countries from 1990 to 2023.^19^ This approach fits a segmented log‑linear function, regressing the natural logarithm of the rate against calendar year. For each identified segment, the annual percent change (APC) was derived from the slope; a negative APC indicates a year‑to‑year decline, whereas a positive APC reflects an increase. The overall trend over the entire observation period was summarized by the average annual percent change (AAPC), a duration‑weighted composite of segment‑specific APC. Optimal joinpoints (maximum of five segments) were selected by minimizing the Bayesian Information Criterion (BIC) following a systematic grid search, and the final model was validated using Monte Carlo permutation tests (4499 permutations, significance threshold *P* < .05). Heteroscedasticity was addressed via the parametric method.^20^

#### Decomposition analysis

Decomposition analysis provides a robust framework for quantifying the relative contributions of population growth, population aging, and changes in disease risk to the observed trends in edentulism burden. This study employed a demographic decomposition approach to disaggregate the total change in edentulism incidence and DALYs between 1990 and 2023 into the independent contributions of three factors: the population growth effect, reflecting the impact of expanding population size on disease burden; the population aging effect, measuring the burden change attributable to shifts in age structure; and the epidemiological change effect, representing the alteration in disease risk levels as captured by age-specific incidence rate changes.^21^ Compared to traditional regression methods, this decomposition framework offers the distinct advantage of precisely isolating the independent contribution of each factor, rather than merely assessing associations between variables. This approach carries significant policy implications for public oral health intervention strategies, as it enables policymakers to accurately evaluate the long-term impact of demographic shifts on healthcare resource needs.^22^ The contribution of each component was expressed as a percentage using the formula: (component value / absolute total change) × 100. If a component’s direction differed from that of the total change – for instance, a negative epidemiological shift occurring alongside an overall positive change – its contribution is reported as a negative percentage, signifying that it partially offset the net increase. It is also possible for the sum of absolute percentage contributions to surpass 100%, which arises because the components are not mutually independent and their effects may partly cancel one another out.^23^

#### Autoregressive integrated moving average (ARIMA) model

The ARIMA model is widely used in public health and epidemiology for short-term and long-term forecasting of disease burden, particularly for handling non-stationary time series data with trends and seasonality.^24^ It captures the key characteristics of time series data through its three components: Autoregressive (AR), Integrated (I), and Moving Average (MA). It effectively handles non-stationary data by differencing the series to achieve stationarity. Furthermore, by adjusting its parameters (p, d, q), the ARIMA model can flexibly adapt to time series data with different characteristics. The specific mathematical formulation of the model has been detailed in previous literature.^25^ Based on this model, the edentulism burden was projected from 2023 through 2038. Forecasts are presented as point estimates; 95% uncertainty intervals (UI) are provided in Supplementary Table S4.All statistical analyses and visualizations were performed using R software (version 4.3.1).

## Results

### Temporal trends and age distribution of edentulism in China and G20 populations, 1990 to 2023

Between 1990 and 2023, the age-standardised incidence rate (ASIR) of edentulism in China declined from 303.6 (95% UI: 242.2-373.7) to 285.6 (95% UI: 235.3-338.6) per 100,000, a cumulative reduction of 5.9%. This decline was smaller than in leading G20 nations such as Australia (30% decrease) and the Republic of Korea (16% decrease). In 2023, China's ASIR was moderate among G20 countries, lower than high-burden nations like Brazil (595.2, 95% UI: 532.9-654.7) and Mexico (424.2, 95% UI: 349.7-509.5) but higher than top performers such as Canada (188.5, 95% UI: 150.7-235.1) and the Republic of Korea (170.5, 95% UI: 131.5-213.1). Consistent with most G20 countries, China's incidence was higher in females than in males in 2023, contrasting with patterns in Japan and Saudi Arabia.

**Fig. 1:**
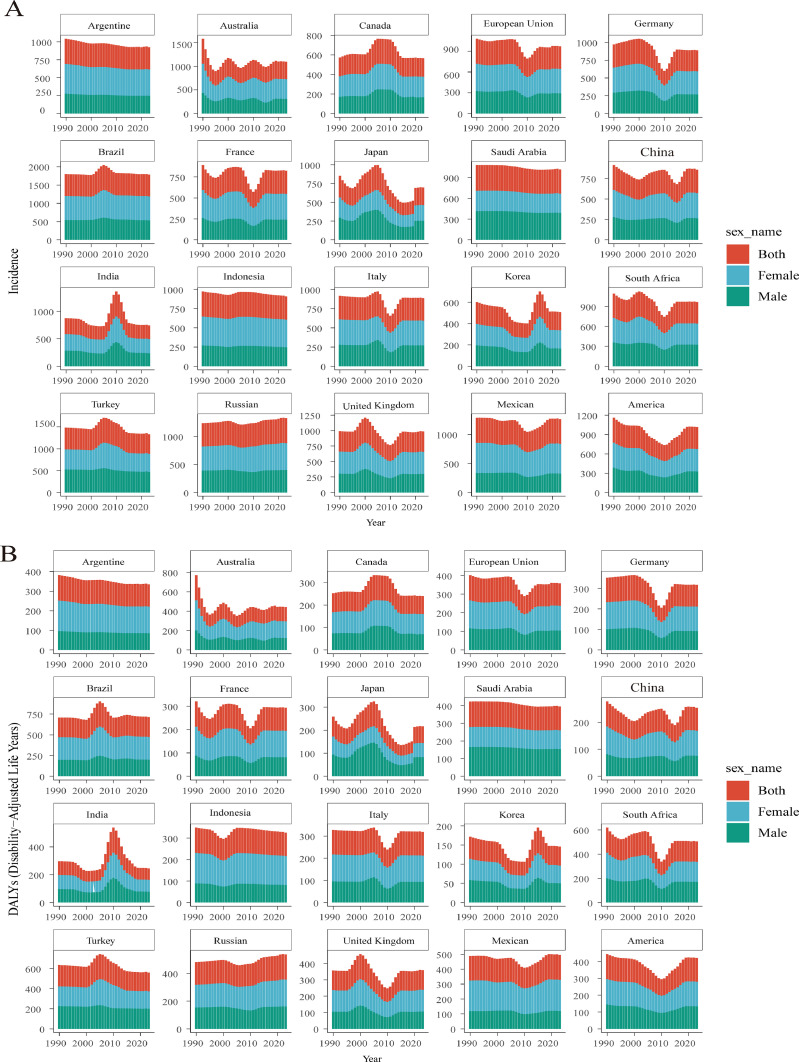
Temporal trends in the incidence and burden of edentulism in China and G20 countries, 1990 to 2023. (A) Age‑standardised incidence rates. (B) Age‑standardised disability‑adjusted life year rates.

Age-standardised DALYs also showed considerable variation. China's burden decreased from 92.7 to 84.6 per 100,000, comparable to Canada (80.1, 95% UI: 49.9-109.7) but above Japan (72.1, 95% UI: 48.8-99.3) and the Republic of Korea (49.1, 95% UI: 32.7-69.7). In contrast, Brazil, South Africa, and Russia exhibited substantially higher burdens. Most countries showed gradual improvement or relative stability. Gender disparities in DALYs were consistent across most nations, with a higher burden in females, as observed in Mexico and Germany. Saudi Arabia was an exception, with a persistently higher burden in males. Gender disparities in DALYs were prominent in most countries, with females consistently experiencing a higher burden than males in 2023, as seen in Mexico (208.5, 95% UI: 138.0-279.6 compared with 120.4, 95% UI: 78.0-160.8) and Germany (119.1, 95% UI: 75.8-168.4 compared with 92.25, 95% UI: 58.1-131.7). Saudi Arabia represented an exception, where male DALYs (154.0, 95% UI: 99.2-212.7) persistently exceeded those of females (106.1, 95% UI: 69.1-145.7) (Figure 1; Supplementary Table S1-2).

The comparative analysis of disease burden by age group revealed that edentulism emerges from the 15 to 19 age group in both populations, increases steadily with age, enters a phase of rapid growth after age 50, and remains at a high level beyond age 70. The peak age for incidence in both Chinese and G20 populations, for both sexes, was concentrated in the 65 to 69 age group. Conversely, the peak age for the overall burden, as measured by DALYs, was concentrated in the 70 to 74 age group for both sexes. A notable gender disparity was observed in China, where the DALYs among females in the 50 to 54 age group were 48% higher than those among males. This magnitude of gender difference was greater than the corresponding disparity observed in the G20 during the same period (Supplementary Figure S1; Supplementary Table S2).

### Joinpoint analysis

From 1990 to 2023, the incidence and DALYs of edentulism in China showed an overall fluctuating downward trend, with AAPC of −0.16 and −0.26, respectively. The period 2015 to 2018 marked the most rapid increase for both measures (APC: incidence 6.84, DALYs 8.54), whereas 2010 to 2014 saw the most significant decline (APC: incidence −4.94, DALYs −5.85). Gender-stratified analysis indicated that the overall decline was more pronounced in females (AAPC: incidence −0.30, DALYs −0.39) than in males (AAPC: incidence −0.1, DALYs −0.24). Both sexes experienced their most rapid increase during 2015 to 2018 and their sharpest decline during 2010 to 2014.

**Fig. 2:**
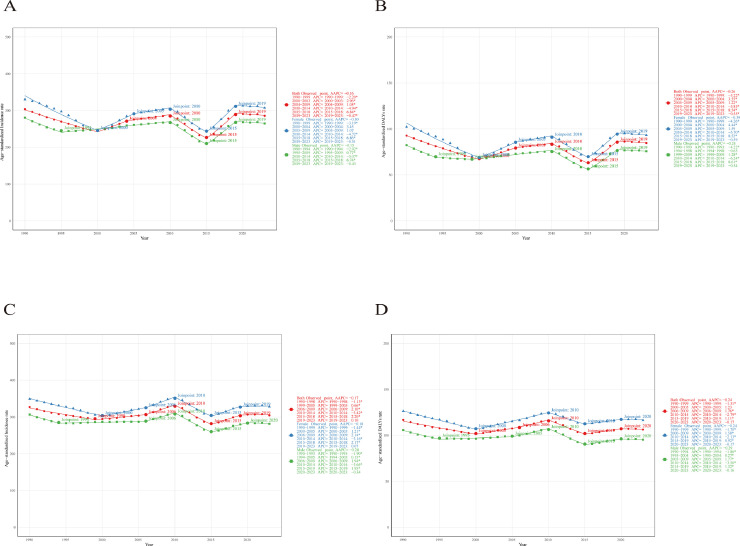
Joinpoint regression analysis of edentulism trends in China and G20 countries, 1990 to 2023. (A) Age‑standardised incidence in China. (B) Age‑standardised disability‑adjusted life years in China. (C) Age‑standardised incidence in G20 countries. (D) Age‑standardised disability‑adjusted life years in G20 countries.

For the G20 overall, incidence and DALYs also exhibited a fluctuating decline from 1990 to 2023, with AAPC of −0.17 and −0.24, respectively. The fastest increase in incidence occurred in 2015 to 2018 (APC 2.20), and its sharpest decrease was in 2010 to 2014 (APC −3.42). For DALYs, the most rapid increase was during 2006 to 2009 (APC 1.76), and the most pronounced decline also took place in 2010 to 2014 (APC −2.79). Similar gender-specific patterns were observed, with the most rapid incidence increase for both males and females occurring in 2015 to 2018, and the most significant declines in both metrics consistently concentrated in the 2010 to 2014 period (Figure 2).

### Decomposition analysis

From 1990 to 2023, the primary drivers behind the increase in edentulism burden differed markedly between China and G20 countries. In G20 nations, the net increase in incidence was primarily attributable to population growth (contribution rate: 78.65%), followed by population aging (17.88%). In contrast, the net increase in China's incidence was predominantly attributable to population aging (contribution rate: 83%-85%), with population growth serving as a secondary factor (21%-23%).

**Fig. 3:**
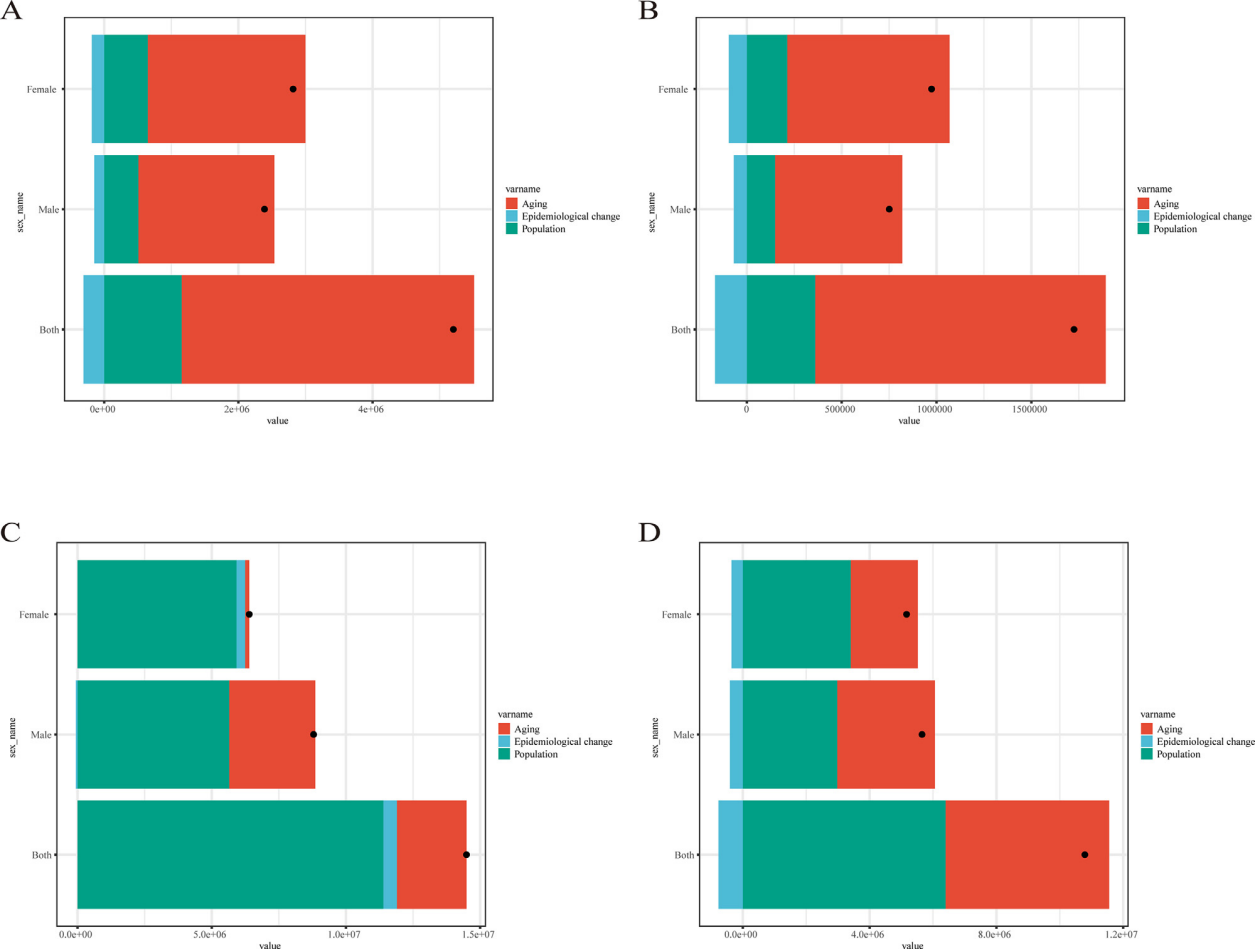
Decomposition of changes in edentulism incidence and burden in China and G20 countries, 1990 to 2023. (A) Contribution to changes in incidence, China. (B) Contribution to changes in disability‑adjusted life years, China. (C) Contribution to changes in incidence, G20. (D) Contribution to changes in disability‑adjusted life years, G20.

Regarding DALYs, the net increase in the G20 was jointly attributable to population growth (59%) and aging (48%). For China, the net increase was again largely attributable to population aging (88.77%), supplemented by population growth (20.92%). Epidemiological changes exerted a negative, offsetting effect in both populations (G20: −7.1%; China: −9.69%), indicating a genuine reduction in disease risk over the period (Figure 3; Supplementary Table S3).

### ARIMA forecasting

**Fig. 4:**
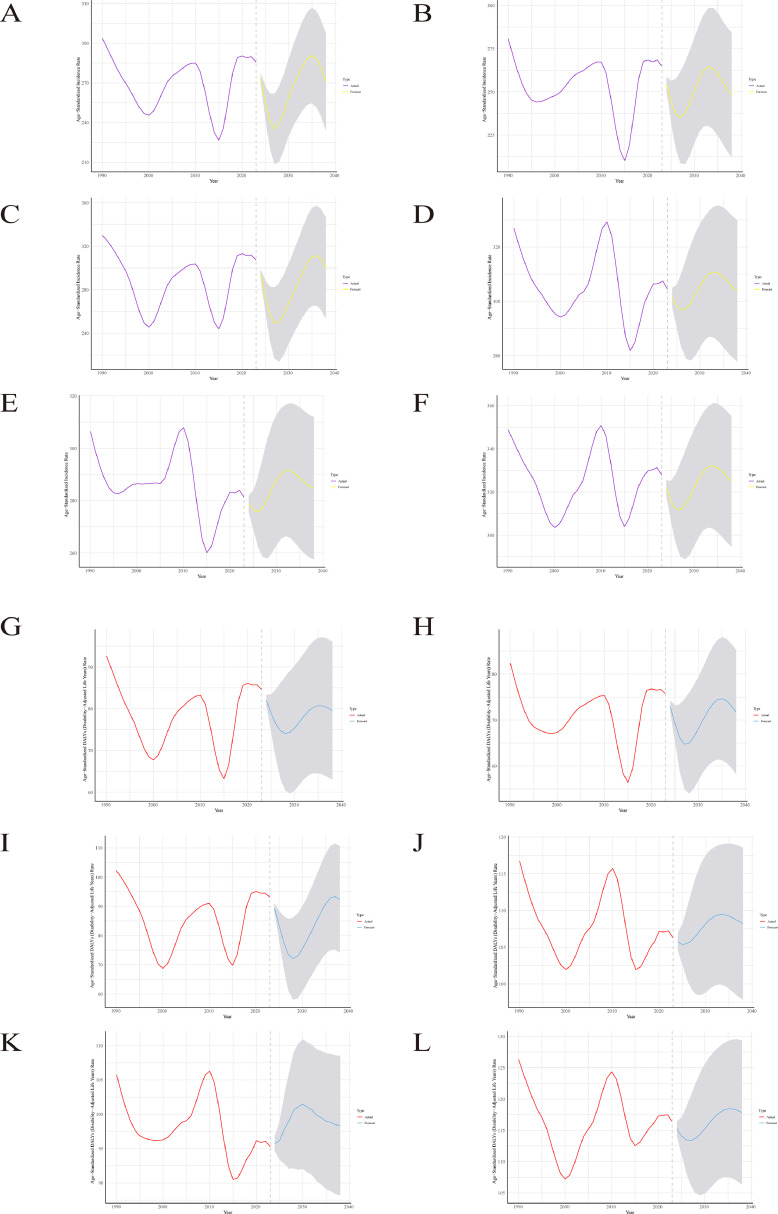
ARIMA model forecasts of edentulism incidence and burden stratified by sex and region, 1990 to 2023. (A) Incidence in China (both sexes). (B) Incidence in Chinese males. (C) Incidence in Chinese females. (D) Incidence in G20 (both sexes). (E) Incidence in G20 males. (F) Incidence in G20 females. (G) Disability‑adjusted life years in China (both sexes). (H) Disability‑adjusted life years in Chinese males. (I) Disability‑adjusted life years in Chinese females. (J) Disability‑adjusted life years in G20 (both sexes). (K) Disability‑adjusted life years in G20 males. (L) Disability‑adjusted life years in G20 females.

ARIMA modelling revealed divergent trends in edentulism incidence and DALYs between China and the G20 from 1990 to 2023 (Figure 4; Supplementary Table S4). In China, DALYs declined from 92.7 in 1990 to 67.7 in 2000, then rebounded and fluctuated, stabilizing around 86.0 during 2020 to 2023. Forecasts indicate a short-term decline to 76.4 (95% UI: 60.4-90.4), followed by gradual recovery to 78.3 (95% UI: 64.2-97.1) −80.7 (95% UI: 74.5-83.4). Incidence fluctuated downward until 2010, then rose significantly to 285.6 in 2023, and is projected to decline slightly and stabilize near 270.

For the G20, Incidence decreased from 326.9 in 1990 to 304.4 in 2023, with forecasts suggesting a low of 296.7 (95% UI: 283.1-328.5) in 2026 before fluctuating between 297.3 (95% UI: 285.8.2-331.2) and 308.5 (95% UI: 283.1-334.5). Incidence historically varied from 281.8 to 329.3 and is projected to decline short-term, rise mid-term, and stabilize between 307.0 (95% UI: 290.0-306.1) and 310.8 (95% UI: 279.3-331.2) after 2031.

Gender-stratified analysis showed that in China, male DALYs bottomed in 2015 at 56.4, rebounded, and are forecast to enter a new short-term decline. Female DALYs fell from 102.2 in 1990, then fluctuated upward; projections indicate an initial decrease then rise, remaining below the 2020 peak. Incidence in males is expected to decline anew, while females show a decrease-then-increase trend, staying below their 2020 peak.

In the G20, male DALYs recently narrowed to 95.7 (95% UI: 92.2-109.0) −96.1 (95% UI: 91.9-110.3) and are predicted to stabilize at 97.8 (95% UI: 92.0-110.9) −101.0 (95% UI: 89.1-108.9). Female DALYs initially declined then increased, with forecasts showing slight fluctuations between 114.1 (95% UI: 104.6-124.2) and 118.2 (95% UI: 106.4-129.4).

## Discussion

Based on GBD 2023 data, this study reveals key disparities in edentulism burden between China and G20 countries from 1990 to 2023. While China exhibited a long-term decline in age-standardised incidence and DALYs, the reduction lagged behind leading G20 nations. Decomposition analysis identified population aging as the primary driver in China (>80% contribution), contrasting with population growth as the dominant factor in the G20. ARIMA projections indicate that after a brief decline, China's burden is expected to rebound and stabilize at a relatively high level, suggesting greater future oral health pressures from population aging compared with the G20 average.

Descriptive and comparative analyses demonstrated that from 1990 to 2023, China witnessed a significant reduction in its age-standardised incidence rate of edentulism and associated DALYs. Although China’s current disease burden remains lower than that of high-burden countries, it exceeds that of top-performing nations, indicating considerable room for improvement in oral health promotion. Analysis across G20 countries revealed a clear association between trends in disease burden and socioeconomic development levels: SDI nations, including Australia, Canada, and the Republic of Korea, achieved substantial reductions through established public oral health systems, whereas countries such as Brazil, South Africa, and Russia showed limited progress due to socioeconomic inequalities and constrained healthcare access.^26^ China’s intermediate position reflects ongoing oral health improvements alongside persistent challenges, including rapid population aging and regional disparities in health resources. Drawing on relevant international experiences – such as the Republic of Korea’s National Oral Health Plan,^27^ and Japan’s '8020 Campaign'^28^ – could therefore provide actionable insights for China. Recent studies have further documented the burden and projections of periodontal diseases and dental caries across Asia, highlighting the region‑specific challenges in oral disease prevention.^29^^,^^30^ These include developing aging-aligned national oral health strategies, strengthening life-course prevention with a focus on midlife periodontal health, and integrating oral health into chronic disease management and essential public health services.

Pronounced gender disparities in the burden of edentulism were observed in both China and G20 countries. In 2023, the age-standardised incidence among Chinese females was significantly higher than among males. This pattern aligned with countries such as Australia and Germany but contrasted with Japan and Saudi Arabia, where males bore a higher burden. The gender gap peaked in the 50 to 54-year age group, where female DALYs substantially exceeded those of males. This life stage often coincides with perimenopause and post menopause, suggesting that oestrogen fluctuations may promote periodontal inflammation and bone resorption.^31^ Indeed, oestrogen deficiency can alter the periodontal microenvironment and stimulate osteoclast activity, thereby exacerbating periodontitis.^32^ However, biological factors alone do not fully explain this disparity. The significant gap also reflects differences in health behaviours and health literacy. Within traditional family roles in China, women often assume greater caregiving responsibilities, which can lead to the neglect of their own oral health and delays in seeking treatment.^33^ Although women generally maintain better daily oral hygiene practices, their awareness of complex oral conditions and their willingness to seek professional care during midlife may be limited by competing family obligations.^34^ Therefore, the elevated burden of edentulism among middle-aged Chinese women may be associated with the interplay of biological aging, hormonal changes, and socioeconomic pressures associated with their social roles. However, causal inference cannot be established from this descriptive study. Nevertheless, the 48% higher DALY burden in women aged 50‑54 years compared with men of the same age group supports targeted interventions, including regular periodontal screening, hormone‑related oral health education, and accessible prosthodontic services for perimenopausal and postmenopausal women.

Joinpoint regression analysis further clarifies the fluctuating nature of China's edentulism burden. While a slight long-term decline was observed, significant fluctuations occurred, marked by a pronounced rebound following a period of rapid reduction. The scale of this rebound notably exceeded the G20 average for the same period. This volatility may be linked to the aging of China's 1960s baby boom generation, the accumulation of risk factors during rapid social transition, and regional disparities in healthcare access.^35^ Notably, a clear temporal disparity exists between China and the G20 average regarding the period of most rapid burden increase: the peak occurred later in China. This time lag likely reflects the evolving oral health challenges associated with different stages of economic development. As a rapidly developing economy, China's demographic and epidemiological shifts occurred later than in more developed economies, thereby delaying its period of steepest burden increase.^36^ This comparison suggests that China's oral health strategies must not only learn from global experience but, more critically, be rooted in the specific trajectory of its own demographic and socioeconomic development to proactively plan for potential future burden fluctuations.^37^

Decomposition analysis reveals fundamental disparities in the factors contributing to changes in edentulism burden between China and the G20. In China, population aging accounts for the overwhelming majority of the net increase in burden, whereas in the G20, population growth is the primary contributing factor, with aging playing a secondary role. This contrast highlights China's unique demographic challenge, as it faces a rapid aging trend before achieving developed socioeconomic status.^38^ Epidemiological changes have negatively contributed in both populations, indicating a reduced disease risk over time; however, in China, the burden reduction from this factor is counterbalanced by the large positive contribution from population aging.^39^ Gender-specific analysis further illuminates this divergence: in the G20, aging drives the burden for males while population growth drives it for females, potentially reflecting women's longer life expectancy^8^); however, in China, aging dominates for both sexes, indicating widespread oral health deterioration across the expanding elderly population regardless of gender. Consequently, while G20 strategies should address service demands arising from population growth, China must prioritize interventions that directly target the older population, given that population aging contributed over 80% of the net increase in the country's edentulism burden. This finding calls for age‑specific strategies – such as integrating oral health assessment and basic prosthodontic care into routine geriatric medical services and long‑term care facilities – rather than focusing predominantly on gender‑specific or general prevention programs.

ARIMA model projections reveal divergent future trajectories for the edentulism burden between China and the G20. While the G20′s DALYs are predicted to stabilize and gradually decline after reaching a low point, China is projected to experience only a brief decrease followed by a sustained rebound, eventually plateauing at a level well above its historical minimum. This trend suggests that China will face substantially greater disease burden pressure in the future compared to the G20 average.^40^ This challenging outlook is closely linked to China's rapidly aging population, reflecting a shift from incidence-driven to aging- and severity-driven disease dynamics. This transition highlights that the core challenge is no longer merely reducing new cases, but also addressing accumulated health loss and rising treatment complexity within a growing elderly population.^41^ Consequently, given that ARIMA forecasts indicate only a brief decline in DALYs followed by a sustained rebound to levels above the historical minimum, China must strategically shift from a focus on prevention alone toward proactive measures that delay disease progression and enable precision management. Simultaneously, geriatric oral health should be urgently integrated into the national public health framework and long‑term care systems to effectively respond to this escalating public health challenge.^42^

The findings carry direct policy implications for China and other rapidly aging economies. First, given that population aging accounted for over 80% of the net increase in edentulism burden, China’s National Health Commission should formally incorporate oral health into the national healthy aging strategy (2025‑2030). This includes adding annual oral examination and basic prosthodontic care to the essential public health services package for all citizens aged 65 years and older. Second, because ARIMA forecasts indicate only a brief decline before a sustained rebound, pre‑emptive investment is needed in geriatric dentistry training programs and in expanding prosthodontic rehabilitation coverage under the Urban Employee Basic Medical Insurance and the New Rural Cooperative Medical Scheme.^14^ Third, the 48% higher DALY burden among women aged 50‑54 compared with men calls for gender‑sensitive policies, such as perimenopausal oral health screening integrated into existing women’s health programmers.^43^ These actions would align with the WHO Global Oral Health Action Plan 2023 to 2030 and help China avoid the high treatment costs associated with edentulism in its aging population.

While our decomposition analysis quantifies the contributions of population aging, growth, and epidemiological change, access to dental services represents an additional factor that may influence the observed trends but could not be directly measured in the GBD framework. In China, dental service access varies markedly by region and socioeconomic status. Rural areas and lower income populations often face shortages of dental professionals, limited insurance coverage for prosthodontic care, and higher out of pocket costs. These disparities likely exacerbate edentulism burden in underserved groups, particularly among the elderly who need tooth replacement the most.^44^ Conversely, improved access in urban areas may partly explain the slower decline in China’s age standardised rates compared with top performing G20 countries. However, quantifying this contribution requires linking GBD estimates with subnational health system indicators, a direction for future research.^45^

This study has several limitations. First, the use of GBD data may involve heterogeneity in original sources and diagnostic criteria for edentulism across different countries and time periods, which could affect the reliability and comparability of the estimates. Second, GBD estimates rely on multiple data sources including vital registration and surveys, but the availability and quality of these sources vary substantially across regions. Low‑ and middle‑income countries often have incomplete or poor‑quality oral health surveillance data, leading to heavier reliance on modelling rather than directly observed data. This may introduce greater uncertainty in burden estimates for certain G20 members. Third, limited representation of remote areas or certain subpopulations (eg, rural elderly, lower socioeconomic groups) within some G20 nations may reduce the generalizability of our findings, as these groups often have higher edentulism rates but are underrepresented in national surveys. Fourth, access to dental services, including preventive care, restorative treatment, and prosthodontic rehabilitation, varies widely across and within G20 countries but was not directly accounted for in our analysis due to data unavailability at the GBD level. Disparities in service access may influence observed trends, particularly in rapidly developing countries like China. Future studies integrating GBD data with health system indicators (for example, dentist to population ratio, insurance coverage for prosthodontics) are needed to disentangle the role of service access from demographic and epidemiological factors. Finally, unmeasured confounders such as genetics, socioeconomic status, oral hygiene practices, and access to dental prostheses were not accounted for, and their omission may introduce bias into the observed results.

## Conclusion

Based on GBD 2023 data, the burden of edentulism in China declined slowly from 1990 to 2023, though less substantially than in most developed G20 countries, and is projected to remain relatively high long‑term. Unlike the G20 overall, where population growth is the main driver, population aging contributed over 80% of the net increase in China's burden, and ARIMA forecasts indicate a sustained rebound after a brief decline. To mitigate this escalating pressure, China should: (1) pivot from generic prevention toward age‑specific interventions targeting the rapidly growing elderly population; (2) integrate comprehensive oral healthcare into geriatric medicine and national chronic disease frameworks to counter the projected rebound; and (3) strengthen midlife periodontal health management, particularly for women in their 50s, who face a 48% higher burden than men. These actions are imperative to address the unprecedented aging‑driven oral health challenge.

## Declaration of generative AI and AI-assisted technologies in the writing process

During the preparation of this work, the authors did not use any generative AI or AI-assisted technologies for data analysis, interpretation, or manuscript writing. Grammarly Business was used solely for grammar and spelling checking. The authors take full responsibility for the content and integrity of this work

## Ethics approval and consent to participate

Ethical approval was not required for this study as it relied exclusively on publicly available, anonymized data from the Global Burden of Disease Study 2023.

## Availability of data and materials

All data used in this study are publicly available from the Global Burden of Disease 2023 repository: https://ghdx.healthdata.org/gbd-2023.

## Funding

This study was supported by the Shandong Provincial Natural Science Foundation (No. ZR2025QC908), and the National Traditional Chinese Medicine Comprehensive Reform Demonstration Zone Science and Technology Co-construction Project (No. GZY-KJS-SD-2024-106). The funders had no role in study design, data collection, analysis, interpretation, manuscript writing, or the decision to submit for publication.

## Author contributions

*Conceptualization*: Yilin Zhang. *Methodology*: Yilin Zhang, Jing Li. *Formal analysis*: Yilin Zhang, Haocheng Wang. *Writing – original draft*: Yilin Zhang. *Writing – review & editing*: All authors. *Data curation*: Lei Zhao. *Software*: Lei Zhao, Haocheng Wang. *Visualization*: Lei Zhao, Haocheng Wang. *Resources*: Fei H. *Validation*: Xiaoshuang Xu, Fei He. *Supervision*: Yuanyuan Liu, Jing Li, Xin Xu. *Funding acquisition*: Yuanyuan Liu, Xin Xu.

## Conflict of interests

None disclosed.

## Appendix Supplementary materials

Image, application 1

## Footnotes

Supplementary material associated with this article can be found in the online version at doi:10.1016/j.identj.2026.109692.
