# Supplementary material for: Edentulism in China and the Group of Twenty (G20): Epidemiological Trends, Decomposition Analysis, and Forecasts to 2038
Source: Int Dent J. 2026 Jun 16;76(4):109692. doi: 10.1016/j.identj.2026.109692 (PMC13285697; doi:10.1016/j.identj.2026.109692)

Supplementary Materials

Table S1.Trends in age-standardized incidence and disability-adjusted life years (DALY) rates for edentulism in China and G20 countries, 1990–2023 1
Table S2. Age-group distributions of edentulism incidence and disability-adjusted life years in China and G20 countries, 1990–2023. 210
Table S3. Decomposition analysis of changes in incidence and disability-adjusted life years from edentulism in China and G20 countries. 225
Table S4. Consolidated Autoregressive Integrated Moving Average(ARIMA) model forecasts of edentulism incidence and disability-adjusted life years, by sex and region (China and G20 countries). 226
Supplemental Fig. 1. Age-group distributions of edentulism incidence and disability-adjusted life years in China and G20 countries, 1990–2023. 240

Table S1.Trends in age-standardized incidence and disability-adjusted life years (DALY) rates for edentulism in China and G20 countries, 1990–2023

measure_name	location_name	sex_name	age_name	cause_name	metric_name	year	val	upper	lower
Incidence	Australia	Male	Age-standardized	Edentulism	Rate	1990	438.3	467.4	409.2
Incidence	Australia	Female	Age-standardized	Edentulism	Rate	1990	616.3	650.7	583.7
Incidence	Australia	Both	Age-standardized	Edentulism	Rate	1990	525.9	557.2	496.5
Incidence	Australia	Male	Age-standardized	Edentulism	Rate	1991	380.9	409.0	353.5
Incidence	Australia	Female	Age-standardized	Edentulism	Rate	1991	526.4	556.2	495.7
Incidence	Australia	Both	Age-standardized	Edentulism	Rate	1991	452.8	480.1	424.6
Incidence	Australia	Male	Age-standardized	Edentulism	Rate	1992	331.8	361.2	302.4
Incidence	Australia	Female	Age-standardized	Edentulism	Rate	1992	449.1	485.9	410.4
Incidence	Australia	Both	Age-standardized	Edentulism	Rate	1992	390.1	420.6	356.5
Incidence	Australia	Male	Age-standardized	Edentulism	Rate	1993	293.7	330.0	261.7
Incidence	Australia	Female	Age-standardized	Edentulism	Rate	1993	388.7	436.5	342.2
Incidence	Australia	Both	Age-standardized	Edentulism	Rate	1993	341.2	382.6	302.1
Incidence	Australia	Male	Age-standardized	Edentulism	Rate	1994	269.0	314.7	229.8
Incidence	Australia	Female	Age-standardized	Edentulism	Rate	1994	349.7	408.5	295.1
Incidence	Australia	Both	Age-standardized	Edentulism	Rate	1994	309.5	361.8	264.0
Incidence	Australia	Male	Age-standardized	Edentulism	Rate	1995	260.2	315.0	213.4
Incidence	Australia	Female	Age-standardized	Edentulism	Rate	1995	336.2	407.4	273.7
Incidence	Australia	Both	Age-standardized	Edentulism	Rate	1995	298.5	361.2	245.7
Incidence	Australia	Male	Age-standardized	Edentulism	Rate	1996	267.5	321.0	219.0
Incidence	Australia	Female	Age-standardized	Edentulism	Rate	1996	348.0	418.3	284.7
Incidence	Australia	Both	Age-standardized	Edentulism	Rate	1996	308.0	370.1	252.6
Incidence	Australia	Male	Age-standardized	Edentulism	Rate	1997	285.1	344.5	231.3
Incidence	Australia	Female	Age-standardized	Edentulism	Rate	1997	375.0	449.4	307.0
Incidence	Australia	Both	Age-standardized	Edentulism	Rate	1997	330.3	397.2	269.7
Incidence	Australia	Male	Age-standardized	Edentulism	Rate	1998	306.5	371.6	248.8
Incidence	Australia	Female	Age-standardized	Edentulism	Rate	1998	407.3	486.5	332.7
Incidence	Australia	Both	Age-standardized	Edentulism	Rate	1998	357.0	427.8	290.3
Incidence	Australia	Male	Age-standardized	Edentulism	Rate	1999	325.2	398.4	264.0
Incidence	Australia	Female	Age-standardized	Edentulism	Rate	1999	435.0	518.2	355.1
Incidence	Australia	Both	Age-standardized	Edentulism	Rate	1999	380.2	456.2	310.8
Incidence	Australia	Male	Age-standardized	Edentulism	Rate	2000	334.6	409.4	268.2
Incidence	Australia	Female	Age-standardized	Edentulism	Rate	2000	448.0	531.5	364.2
Incidence	Australia	Both	Age-standardized	Edentulism	Rate	2000	391.4	471.0	317.3
Incidence	Australia	Male	Age-standardized	Edentulism	Rate	2001	330.4	392.4	275.5
Incidence	Australia	Female	Age-standardized	Edentulism	Rate	2001	440.7	511.6	369.7
Incidence	Australia	Both	Age-standardized	Edentulism	Rate	2001	385.6	455.2	323.6
Incidence	Australia	Male	Age-standardized	Edentulism	Rate	2002	316.7	367.0	270.2
Incidence	Australia	Female	Age-standardized	Edentulism	Rate	2002	419.8	481.3	359.1
Incidence	Australia	Both	Age-standardized	Edentulism	Rate	2002	368.3	423.8	315.3
Incidence	Australia	Male	Age-standardized	Edentulism	Rate	2003	299.8	341.9	261.8
Incidence	Australia	Female	Age-standardized	Edentulism	Rate	2003	394.2	442.6	345.2

Incidence	Australia	Both	Age-standardized	Edentulism	Rate	2003	346.9	392.9	303.2
Incidence	Australia	Male	Age-standardized	Edentulism	Rate	2004	285.8	322.7	253.6
Incidence	Australia	Female	Age-standardized	Edentulism	Rate	2004	372.5	412.4	331.1
Incidence	Australia	Both	Age-standardized	Edentulism	Rate	2004	329.1	367.5	290.8
Incidence	Australia	Male	Age-standardized	Edentulism	Rate	2005	281.0	313.8	249.5
Incidence	Australia	Female	Age-standardized	Edentulism	Rate	2005	363.5	400.8	322.0
Incidence	Australia	Both	Age-standardized	Edentulism	Rate	2005	322.2	356.0	285.9
Incidence	Australia	Male	Age-standardized	Edentulism	Rate	2006	287.3	316.3	258.0
Incidence	Australia	Female	Age-standardized	Edentulism	Rate	2006	369.3	404.0	332.5
Incidence	Australia	Both	Age-standardized	Edentulism	Rate	2006	328.3	359.1	295.7
Incidence	Australia	Male	Age-standardized	Edentulism	Rate	2007	299.9	325.7	272.3
Incidence	Australia	Female	Age-standardized	Edentulism	Rate	2007	383.2	414.6	350.8
Incidence	Australia	Both	Age-standardized	Edentulism	Rate	2007	341.5	369.8	310.9
Incidence	Australia	Male	Age-standardized	Edentulism	Rate	2008	314.4	338.0	288.2
Incidence	Australia	Female	Age-standardized	Edentulism	Rate	2008	400.3	428.0	371.6
Incidence	Australia	Both	Age-standardized	Edentulism	Rate	2008	357.4	383.4	330.4
Incidence	Australia	Male	Age-standardized	Edentulism	Rate	2009	326.8	351.0	301.0
Incidence	Australia	Female	Age-standardized	Edentulism	Rate	2009	415.7	442.1	390.0
Incidence	Australia	Both	Age-standardized	Edentulism	Rate	2009	371.4	396.2	347.1
Incidence	Australia	Male	Age-standardized	Edentulism	Rate	2010	333.0	359.3	306.8
Incidence	Australia	Female	Age-standardized	Edentulism	Rate	2010	424.7	452.5	398.6
Incidence	Australia	Both	Age-standardized	Edentulism	Rate	2010	379.1	407.2	353.3
Incidence	Australia	Male	Age-standardized	Edentulism	Rate	2011	323.2	347.2	297.6
Incidence	Australia	Female	Age-standardized	Edentulism	Rate	2011	427.5	456.9	398.7
Incidence	Australia	Both	Age-standardized	Edentulism	Rate	2011	375.7	402.2	349.1
Incidence	Australia	Male	Age-standardized	Edentulism	Rate	2012	297.4	326.4	269.0
Incidence	Australia	Female	Age-standardized	Edentulism	Rate	2012	428.1	466.0	390.2
Incidence	Australia	Both	Age-standardized	Edentulism	Rate	2012	363.5	393.9	331.1
Incidence	Australia	Male	Age-standardized	Edentulism	Rate	2013	266.3	302.6	235.4
Incidence	Australia	Female	Age-standardized	Edentulism	Rate	2013	427.4	474.4	377.7
Incidence	Australia	Both	Age-standardized	Edentulism	Rate	2013	348.1	388.5	307.4
Incidence	Australia	Male	Age-standardized	Edentulism	Rate	2014	240.5	283.3	203.6
Incidence	Australia	Female	Age-standardized	Edentulism	Rate	2014	426.4	489.7	361.1
Incidence	Australia	Both	Age-standardized	Edentulism	Rate	2014	335.1	385.6	285.1
Incidence	Australia	Male	Age-standardized	Edentulism	Rate	2015	230.6	280.1	187.4
Incidence	Australia	Female	Age-standardized	Edentulism	Rate	2015	425.9	506.3	350.1
Incidence	Australia	Both	Age-standardized	Edentulism	Rate	2015	330.1	392.2	270.3
Incidence	Australia	Male	Age-standardized	Edentulism	Rate	2016	244.4	299.6	198.6
Incidence	Australia	Female	Age-standardized	Edentulism	Rate	2016	426.0	504.1	350.0
Incidence	Australia	Both	Age-standardized	Edentulism	Rate	2016	337.1	401.8	276.6
Incidence	Australia	Male	Age-standardized	Edentulism	Rate	2017	273.3	336.2	224.0
Incidence	Australia	Female	Age-standardized	Edentulism	Rate	2017	426.1	504.3	349.7
Incidence	Australia	Both	Age-standardized	Edentulism	Rate	2017	351.3	421.1	289.2
Incidence	Australia	Male	Age-standardized	Edentulism	Rate	2018	302.6	374.8	244.4

Incidence	Australia	Female	Age-standardized	Edentulism	Rate	2018	426.2	511.0	349.1
Incidence	Australia	Both	Age-standardized	Edentulism	Rate	2018	365.7	439.2	300.1
Incidence	Australia	Male	Age-standardized	Edentulism	Rate	2019	317.9	392.5	254.7
Incidence	Australia	Female	Age-standardized	Edentulism	Rate	2019	426.2	512.5	347.7
Incidence	Australia	Both	Age-standardized	Edentulism	Rate	2019	373.2	452.7	303.9
Incidence	Australia	Male	Age-standardized	Edentulism	Rate	2020	314.8	387.9	248.0
Incidence	Australia	Female	Age-standardized	Edentulism	Rate	2020	418.3	501.2	339.4
Incidence	Australia	Both	Age-standardized	Edentulism	Rate	2020	367.7	447.5	295.7
Incidence	Australia	Male	Age-standardized	Edentulism	Rate	2021	316.4	388.0	255.0
Incidence	Australia	Female	Age-standardized	Edentulism	Rate	2021	421.0	501.8	339.7
Incidence	Australia	Both	Age-standardized	Edentulism	Rate	2021	369.8	446.5	300.3
Incidence	Australia	Male	Age-standardized	Edentulism	Rate	2022	315.2	388.2	250.6
Incidence	Australia	Female	Age-standardized	Edentulism	Rate	2022	419.8	496.8	341.9
Incidence	Australia	Both	Age-standardized	Edentulism	Rate	2022	368.7	443.0	297.4
Incidence	Australia	Male	Age-standardized	Edentulism	Rate	2023	313.3	385.9	249.1
Incidence	Australia	Female	Age-standardized	Edentulism	Rate	2023	416.9	493.3	339.6
Incidence	Australia	Both	Age-standardized	Edentulism	Rate	2023	366.3	440.1	295.5
Incidence	Republic of Korea	Male	Age-standardized	Edentulism	Rate	1990	195.7	245.6	153.5
Incidence	Republic of Korea	Female	Age-standardized	Edentulism	Rate	1990	201.8	256.0	152.9
Incidence	Republic of Korea	Both	Age-standardized	Edentulism	Rate	1990	203.4	255.6	160.0
Incidence	Republic of Korea	Male	Age-standardized	Edentulism	Rate	1991	193.5	242.3	151.2
Incidence	Republic of Korea	Female	Age-standardized	Edentulism	Rate	1991	198.9	251.7	151.7
Incidence	Republic of Korea	Both	Age-standardized	Edentulism	Rate	1991	200.8	252.1	157.6
Incidence	Republic of Korea	Male	Age-standardized	Edentulism	Rate	1992	191.5	239.4	149.3
Incidence	Republic of Korea	Female	Age-standardized	Edentulism	Rate	1992	196.2	248.4	150.6
Incidence	Republic of Korea	Both	Age-standardized	Edentulism	Rate	1992	198.4	249.3	155.2
Incidence	Republic of Korea	Male	Age-standardized	Edentulism	Rate	1993	189.6	236.0	147.3
Incidence	Republic of Korea	Female	Age-standardized	Edentulism	Rate	1993	193.8	244.3	149.4
Incidence	Republic of Korea	Both	Age-standardized	Edentulism	Rate	1993	196.2	246.1	152.8
Incidence	Republic of Korea	Male	Age-standardized	Edentulism	Rate	1994	187.8	233.5	145.2
Incidence	Republic of Korea	Female	Age-standardized	Edentulism	Rate	1994	191.7	241.6	147.9
Incidence	Republic of Korea	Both	Age-standardized	Edentulism	Rate	1994	194.3	243.5	150.7
Incidence	Republic of Korea	Male	Age-standardized	Edentulism	Rate	1995	186.3	232.2	143.7
Incidence	Republic of Korea	Female	Age-standardized	Edentulism	Rate	1995	190.1	238.9	146.3
Incidence	Republic of Korea	Both	Age-standardized	Edentulism	Rate	1995	192.8	241.4	148.7
Incidence	Republic of Korea	Male	Age-standardized	Edentulism	Rate	1996	185.0	230.9	142.5
Incidence	Republic of Korea	Female	Age-standardized	Edentulism	Rate	1996	189.0	237.7	146.0
Incidence	Republic of Korea	Both	Age-standardized	Edentulism	Rate	1996	191.6	239.5	148.1
Incidence	Republic of Korea	Male	Age-standardized	Edentulism	Rate	1997	183.9	231.4	142.4
Incidence	Republic of Korea	Female	Age-standardized	Edentulism	Rate	1997	188.1	236.8	145.9
Incidence	Republic of Korea	Both	Age-standardized	Edentulism	Rate	1997	190.6	237.7	147.6
Incidence	Republic of Korea	Male	Age-standardized	Edentulism	Rate	1998	182.9	230.8	141.8
Incidence	Republic of Korea	Female	Age-standardized	Edentulism	Rate	1998	187.3	236.4	145.8
Incidence	Republic of Korea	Both	Age-standardized	Edentulism	Rate	1998	189.7	236.7	147.1

Incidence	Republic of Korea	Male	Age-standardized	Edentulism	Rate	1999	181.6	229.5	140.8
Incidence	Republic of Korea	Female	Age-standardized	Edentulism	Rate	1999	186.3	235.7	145.5
Incidence	Republic of Korea	Both	Age-standardized	Edentulism	Rate	1999	188.5	235.5	146.4
Incidence	Republic of Korea	Male	Age-standardized	Edentulism	Rate	2000	180.1	229.0	140.3
Incidence	Republic of Korea	Female	Age-standardized	Edentulism	Rate	2000	184.9	234.4	145.0
Incidence	Republic of Korea	Both	Age-standardized	Edentulism	Rate	2000	187.0	234.0	145.4
Incidence	Republic of Korea	Male	Age-standardized	Edentulism	Rate	2001	174.6	214.4	139.9
Incidence	Republic of Korea	Female	Age-standardized	Edentulism	Rate	2001	179.5	221.0	144.0
Incidence	Republic of Korea	Both	Age-standardized	Edentulism	Rate	2001	181.1	221.0	145.3
Incidence	Republic of Korea	Male	Age-standardized	Edentulism	Rate	2002	163.9	194.9	135.7
Incidence	Republic of Korea	Female	Age-standardized	Edentulism	Rate	2002	168.7	200.1	141.4
Incidence	Republic of Korea	Both	Age-standardized	Edentulism	Rate	2002	169.8	201.3	142.3
Incidence	Republic of Korea	Male	Age-standardized	Edentulism	Rate	2003	151.7	175.3	129.7
Incidence	Republic of Korea	Female	Age-standardized	Edentulism	Rate	2003	156.2	180.8	135.0
Incidence	Republic of Korea	Both	Age-standardized	Edentulism	Rate	2003	156.7	180.4	136.0
Incidence	Republic of Korea	Male	Age-standardized	Edentulism	Rate	2004	141.3	160.5	123.2
Incidence	Republic of Korea	Female	Age-standardized	Edentulism	Rate	2004	145.5	165.1	127.0
Incidence	Republic of Korea	Both	Age-standardized	Edentulism	Rate	2004	145.5	164.3	126.9
Incidence	Republic of Korea	Male	Age-standardized	Edentulism	Rate	2005	136.3	154.3	117.8
Incidence	Republic of Korea	Female	Age-standardized	Edentulism	Rate	2005	140.1	159.5	123.1
Incidence	Republic of Korea	Both	Age-standardized	Edentulism	Rate	2005	140.0	158.9	122.9
Incidence	Republic of Korea	Male	Age-standardized	Edentulism	Rate	2006	135.2	153.0	117.5
Incidence	Republic of Korea	Female	Age-standardized	Edentulism	Rate	2006	138.5	157.2	122.5
Incidence	Republic of Korea	Both	Age-standardized	Edentulism	Rate	2006	138.5	156.1	121.7
Incidence	Republic of Korea	Male	Age-standardized	Edentulism	Rate	2007	134.0	151.2	116.9
Incidence	Republic of Korea	Female	Age-standardized	Edentulism	Rate	2007	137.2	154.8	121.0
Incidence	Republic of Korea	Both	Age-standardized	Edentulism	Rate	2007	137.1	154.2	120.1
Incidence	Republic of Korea	Male	Age-standardized	Edentulism	Rate	2008	133.0	149.8	115.8
Incidence	Republic of Korea	Female	Age-standardized	Edentulism	Rate	2008	136.0	152.8	119.6
Incidence	Republic of Korea	Both	Age-standardized	Edentulism	Rate	2008	136.0	152.6	119.2
Incidence	Republic of Korea	Male	Age-standardized	Edentulism	Rate	2009	132.3	149.3	114.6
Incidence	Republic of Korea	Female	Age-standardized	Edentulism	Rate	2009	135.3	151.8	118.7
Incidence	Republic of Korea	Both	Age-standardized	Edentulism	Rate	2009	135.2	151.2	118.8
Incidence	Republic of Korea	Male	Age-standardized	Edentulism	Rate	2010	132.0	149.6	114.0
Incidence	Republic of Korea	Female	Age-standardized	Edentulism	Rate	2010	135.0	151.9	118.1
Incidence	Republic of Korea	Both	Age-standardized	Edentulism	Rate	2010	134.8	151.0	118.4
Incidence	Republic of Korea	Male	Age-standardized	Edentulism	Rate	2011	141.5	159.7	123.0
Incidence	Republic of Korea	Female	Age-standardized	Edentulism	Rate	2011	146.2	163.2	129.1
Incidence	Republic of Korea	Both	Age-standardized	Edentulism	Rate	2011	145.4	162.4	128.5
Incidence	Republic of Korea	Male	Age-standardized	Edentulism	Rate	2012	164.1	185.5	142.9
Incidence	Republic of Korea	Female	Age-standardized	Edentulism	Rate	2012	172.8	193.3	153.4
Incidence	Republic of Korea	Both	Age-standardized	Edentulism	Rate	2012	170.7	190.3	151.9
Incidence	Republic of Korea	Male	Age-standardized	Edentulism	Rate	2013	191.1	217.8	167.1
Incidence	Republic of Korea	Female	Age-standardized	Edentulism	Rate	2013	204.5	228.5	181.6

Incidence	Republic of Korea	Both	Age-standardized	Edentulism	Rate	2013	200.8	223.7	179.7
Incidence	Republic of Korea	Male	Age-standardized	Edentulism	Rate	2014	213.7	246.0	186.9
Incidence	Republic of Korea	Female	Age-standardized	Edentulism	Rate	2014	231.2	258.6	204.7
Incidence	Republic of Korea	Both	Age-standardized	Edentulism	Rate	2014	226.0	252.6	201.7
Incidence	Republic of Korea	Male	Age-standardized	Edentulism	Rate	2015	223.2	257.8	194.9
Incidence	Republic of Korea	Female	Age-standardized	Edentulism	Rate	2015	242.3	271.6	214.9
Incidence	Republic of Korea	Both	Age-standardized	Edentulism	Rate	2015	236.4	266.1	210.2
Incidence	Republic of Korea	Male	Age-standardized	Edentulism	Rate	2016	214.6	251.0	185.1
Incidence	Republic of Korea	Female	Age-standardized	Edentulism	Rate	2016	231.7	261.5	203.9
Incidence	Republic of Korea	Both	Age-standardized	Edentulism	Rate	2016	226.4	258.5	199.3
Incidence	Republic of Korea	Male	Age-standardized	Edentulism	Rate	2017	195.7	233.8	165.4
Incidence	Republic of Korea	Female	Age-standardized	Edentulism	Rate	2017	208.1	242.1	178.8
Incidence	Republic of Korea	Both	Age-standardized	Edentulism	Rate	2017	204.7	239.4	175.9
Incidence	Republic of Korea	Male	Age-standardized	Edentulism	Rate	2018	176.6	217.3	143.0
Incidence	Republic of Korea	Female	Age-standardized	Edentulism	Rate	2018	184.3	225.1	149.6
Incidence	Republic of Korea	Both	Age-standardized	Edentulism	Rate	2018	182.8	221.8	149.3
Incidence	Republic of Korea	Male	Age-standardized	Edentulism	Rate	2019	167.6	214.0	130.4
Incidence	Republic of Korea	Female	Age-standardized	Edentulism	Rate	2019	173.0	220.7	134.3
Incidence	Republic of Korea	Both	Age-standardized	Edentulism	Rate	2019	172.3	216.2	134.1
Incidence	Republic of Korea	Male	Age-standardized	Edentulism	Rate	2020	168.1	214.8	129.0
Incidence	Republic of Korea	Female	Age-standardized	Edentulism	Rate	2020	173.2	222.4	134.0
Incidence	Republic of Korea	Both	Age-standardized	Edentulism	Rate	2020	172.6	217.7	135.3
Incidence	Republic of Korea	Male	Age-standardized	Edentulism	Rate	2021	168.2	214.0	130.3
Incidence	Republic of Korea	Female	Age-standardized	Edentulism	Rate	2021	172.5	217.5	130.6
Incidence	Republic of Korea	Both	Age-standardized	Edentulism	Rate	2021	172.2	213.8	133.9
Incidence	Republic of Korea	Male	Age-standardized	Edentulism	Rate	2022	167.6	212.6	130.0
Incidence	Republic of Korea	Female	Age-standardized	Edentulism	Rate	2022	172.3	217.3	130.2
Incidence	Republic of Korea	Both	Age-standardized	Edentulism	Rate	2022	171.7	214.6	132.5
Incidence	Republic of Korea	Male	Age-standardized	Edentulism	Rate	2023	166.5	211.2	129.2
Incidence	Republic of Korea	Female	Age-standardized	Edentulism	Rate	2023	171.1	215.7	129.2
Incidence	Republic of Korea	Both	Age-standardized	Edentulism	Rate	2023	170.5	213.1	131.5
Incidence	Federal Republic of Germany	Male	Age-standardized	Edentulism	Rate	1990	294.7	363.9	235.3
Incidence	Federal Republic of Germany	Female	Age-standardized	Edentulism	Rate	1990	352.5	432.4	282.6
Incidence	Federal Republic of Germany	Both	Age-standardized	Edentulism	Rate	1990	323.5	395.9	258.3
Incidence	Federal Republic of Germany	Male	Age-standardized	Edentulism	Rate	1991	298.8	352.1	249.8
Incidence	Federal Republic of Germany	Female	Age-standardized	Edentulism	Rate	1991	355.8	418.1	300.2
Incidence	Federal Republic of Germany	Both	Age-standardized	Edentulism	Rate	1991	326.9	384.0	275.5
Incidence	Federal Republic	Male	Age-standardized	Edentulism	Rate	1992	302.6	346.0	260.5

	of Germany								
Incidence	Federal Republic of Germany	Female	Age-standardized	Edentulism	Rate	1992	358.9	406.7	314.0
Incidence	Federal Republic of Germany	Both	Age-standardized	Edentulism	Rate	1992	330.2	372.8	288.0
Incidence	Federal Republic of Germany	Male	Age-standardized	Edentulism	Rate	1993	306.2	342.9	269.4
Incidence	Federal Republic of Germany	Female	Age-standardized	Edentulism	Rate	1993	361.8	399.8	323.2
Incidence	Federal Republic of Germany	Both	Age-standardized	Edentulism	Rate	1993	333.2	370.3	296.1
Incidence	Federal Republic of Germany	Male	Age-standardized	Edentulism	Rate	1994	309.4	341.4	277.1
Incidence	Federal Republic of Germany	Female	Age-standardized	Edentulism	Rate	1994	364.6	396.8	330.7
Incidence	Federal Republic of Germany	Both	Age-standardized	Edentulism	Rate	1994	336.0	366.9	302.6
Incidence	Federal Republic of Germany	Male	Age-standardized	Edentulism	Rate	1995	312.3	340.8	282.2
Incidence	Federal Republic of Germany	Female	Age-standardized	Edentulism	Rate	1995	367.1	396.8	336.2
Incidence	Federal Republic of Germany	Both	Age-standardized	Edentulism	Rate	1995	338.6	366.4	308.4
Incidence	Federal Republic of Germany	Male	Age-standardized	Edentulism	Rate	1996	315.1	342.5	288.7
Incidence	Federal Republic of Germany	Female	Age-standardized	Edentulism	Rate	1996	369.9	398.2	341.9
Incidence	Federal Republic of Germany	Both	Age-standardized	Edentulism	Rate	1996	341.2	368.1	314.8
Incidence	Federal Republic of Germany	Male	Age-standardized	Edentulism	Rate	1997	318.0	344.3	293.5
Incidence	Federal Republic of Germany	Female	Age-standardized	Edentulism	Rate	1997	372.8	398.0	347.1
Incidence	Federal Republic of Germany	Both	Age-standardized	Edentulism	Rate	1997	344.0	369.7	320.4
Incidence	Federal Republic of Germany	Male	Age-standardized	Edentulism	Rate	1998	320.5	344.7	297.5
Incidence	Federal Republic of Germany	Female	Age-standardized	Edentulism	Rate	1998	375.5	400.0	352.6
Incidence	Federal Republic of Germany	Both	Age-standardized	Edentulism	Rate	1998	346.6	370.2	324.1
Incidence	Federal Republic of Germany	Male	Age-standardized	Edentulism	Rate	1999	322.2	345.7	300.8
Incidence	Federal Republic	Female	Age-standardized	Edentulism	Rate	1999	377.4	400.8	355.4

	of Germany								
Incidence	Federal Republic of Germany	Both	Age-standardized	Edentulism	Rate	1999	348.4	370.6	325.9
Incidence	Federal Republic of Germany	Male	Age-standardized	Edentulism	Rate	2000	322.8	346.5	302.0
Incidence	Federal Republic of Germany	Female	Age-standardized	Edentulism	Rate	2000	378.0	401.6	354.6
Incidence	Federal Republic of Germany	Both	Age-standardized	Edentulism	Rate	2000	349.0	371.2	327.4
Incidence	Federal Republic of Germany	Male	Age-standardized	Edentulism	Rate	2001	322.2	345.5	301.7
Incidence	Federal Republic of Germany	Female	Age-standardized	Edentulism	Rate	2001	374.6	397.4	351.0
Incidence	Federal Republic of Germany	Both	Age-standardized	Edentulism	Rate	2001	346.7	368.5	325.4
Incidence	Federal Republic of Germany	Male	Age-standardized	Edentulism	Rate	2002	320.3	344.1	298.7
Incidence	Federal Republic of Germany	Female	Age-standardized	Edentulism	Rate	2002	366.5	390.8	340.4
Incidence	Federal Republic of Germany	Both	Age-standardized	Edentulism	Rate	2002	340.9	363.6	318.8
Incidence	Federal Republic of Germany	Male	Age-standardized	Edentulism	Rate	2003	316.9	340.0	292.9
Incidence	Federal Republic of Germany	Female	Age-standardized	Edentulism	Rate	2003	355.6	383.8	326.2
Incidence	Federal Republic of Germany	Both	Age-standardized	Edentulism	Rate	2003	333.0	357.9	308.2
Incidence	Federal Republic of Germany	Male	Age-standardized	Edentulism	Rate	2004	311.4	336.8	286.2
Incidence	Federal Republic of Germany	Female	Age-standardized	Edentulism	Rate	2004	344.0	375.6	313.5
Incidence	Federal Republic of Germany	Both	Age-standardized	Edentulism	Rate	2004	324.1	350.4	296.4
Incidence	Federal Republic of Germany	Male	Age-standardized	Edentulism	Rate	2005	303.5	329.5	278.2
Incidence	Federal Republic of Germany	Female	Age-standardized	Edentulism	Rate	2005	333.8	366.6	301.6
Incidence	Federal Republic of Germany	Both	Age-standardized	Edentulism	Rate	2005	315.1	341.7	287.4
Incidence	Federal Republic of Germany	Male	Age-standardized	Edentulism	Rate	2006	284.4	308.4	260.3
Incidence	Federal Republic of Germany	Female	Age-standardized	Edentulism	Rate	2006	316.6	347.7	287.3
Incidence	Federal Republic	Both	Age-standardized	Edentulism	Rate	2006	297.5	323.3	271.8

	of Germany								
Incidence	Federal Republic of Germany	Male	Age-standardized	Edentulism	Rate	2007	252.2	272.7	230.4
Incidence	Federal Republic of Germany	Female	Age-standardized	Edentulism	Rate	2007	288.2	313.8	263.7
Incidence	Federal Republic of Germany	Both	Age-standardized	Edentulism	Rate	2007	268.1	290.1	244.8
Incidence	Federal Republic of Germany	Male	Age-standardized	Edentulism	Rate	2008	216.9	234.3	198.2
Incidence	Federal Republic of Germany	Female	Age-standardized	Edentulism	Rate	2008	257.3	276.8	236.6
Incidence	Federal Republic of Germany	Both	Age-standardized	Edentulism	Rate	2008	235.9	254.3	215.7
Incidence	Federal Republic of Germany	Male	Age-standardized	Edentulism	Rate	2009	188.4	203.4	173.0
Incidence	Federal Republic of Germany	Female	Age-standardized	Edentulism	Rate	2009	232.5	249.2	213.9
Incidence	Federal Republic of Germany	Both	Age-standardized	Edentulism	Rate	2009	209.9	226.0	192.9
Incidence	Federal Republic of Germany	Male	Age-standardized	Edentulism	Rate	2010	176.7	190.3	161.9
Incidence	Federal Republic of Germany	Female	Age-standardized	Edentulism	Rate	2010	222.3	238.9	204.1
Incidence	Federal Republic of Germany	Both	Age-standardized	Edentulism	Rate	2010	199.3	214.8	183.0
Incidence	Federal Republic of Germany	Male	Age-standardized	Edentulism	Rate	2011	186.8	202.9	170.5
Incidence	Federal Republic of Germany	Female	Age-standardized	Edentulism	Rate	2011	233.1	252.4	212.6
Incidence	Federal Republic of Germany	Both	Age-standardized	Edentulism	Rate	2011	209.7	228.0	190.9
Incidence	Federal Republic of Germany	Male	Age-standardized	Edentulism	Rate	2012	210.6	233.9	186.9
Incidence	Federal Republic of Germany	Female	Age-standardized	Edentulism	Rate	2012	259.0	287.9	233.1
Incidence	Federal Republic of Germany	Both	Age-standardized	Edentulism	Rate	2012	234.4	260.4	209.5
Incidence	Federal Republic of Germany	Male	Age-standardized	Edentulism	Rate	2013	239.1	276.2	204.1
Incidence	Federal Republic of Germany	Female	Age-standardized	Edentulism	Rate	2013	289.8	333.3	251.7
Incidence	Federal Republic of Germany	Both	Age-standardized	Edentulism	Rate	2013	263.9	303.7	228.9
Incidence	Federal Republic	Male	Age-standardized	Edentulism	Rate	2014	263.1	316.7	215.2

	of Germany								
Incidence	Federal Republic of Germany	Female	Age-standardized	Edentulism	Rate	2014	315.7	377.7	264.1
Incidence	Federal Republic of Germany	Both	Age-standardized	Edentulism	Rate	2014	288.8	345.3	237.9
Incidence	Federal Republic of Germany	Male	Age-standardized	Edentulism	Rate	2015	273.2	338.7	213.5
Incidence	Federal Republic of Germany	Female	Age-standardized	Edentulism	Rate	2015	326.6	403.6	265.3
Incidence	Federal Republic of Germany	Both	Age-standardized	Edentulism	Rate	2015	299.3	367.2	240.3
Incidence	Federal Republic of Germany	Male	Age-standardized	Edentulism	Rate	2016	272.9	337.8	214.3
Incidence	Federal Republic of Germany	Female	Age-standardized	Edentulism	Rate	2016	326.3	402.0	262.2
Incidence	Federal Republic of Germany	Both	Age-standardized	Edentulism	Rate	2016	299.0	366.3	239.6
Incidence	Federal Republic of Germany	Male	Age-standardized	Edentulism	Rate	2017	272.0	336.2	213.2
Incidence	Federal Republic of Germany	Female	Age-standardized	Edentulism	Rate	2017	325.3	402.8	259.5
Incidence	Federal Republic of Germany	Both	Age-standardized	Edentulism	Rate	2017	298.1	367.1	238.3
Incidence	Federal Republic of Germany	Male	Age-standardized	Edentulism	Rate	2018	271.0	335.1	214.9
Incidence	Federal Republic of Germany	Female	Age-standardized	Edentulism	Rate	2018	324.2	400.6	256.5
Incidence	Federal Republic of Germany	Both	Age-standardized	Edentulism	Rate	2018	297.0	367.5	236.9
Incidence	Federal Republic of Germany	Male	Age-standardized	Edentulism	Rate	2019	270.3	334.3	215.4
Incidence	Federal Republic of Germany	Female	Age-standardized	Edentulism	Rate	2019	323.4	398.3	255.8
Incidence	Federal Republic of Germany	Both	Age-standardized	Edentulism	Rate	2019	296.3	367.0	236.0
Incidence	Federal Republic of Germany	Male	Age-standardized	Edentulism	Rate	2020	270.2	333.0	214.3
Incidence	Federal Republic of Germany	Female	Age-standardized	Edentulism	Rate	2020	323.5	397.0	255.1
Incidence	Federal Republic of Germany	Both	Age-standardized	Edentulism	Rate	2020	296.4	364.7	234.6
Incidence	Federal Republic of Germany	Male	Age-standardized	Edentulism	Rate	2021	271.1	337.3	211.9
Incidence	Federal Republic	Female	Age-standardized	Edentulism	Rate	2021	323.4	398.9	258.4

	of Germany								
Incidence	Federal Republic of Germany	Both	Age-standardized	Edentulism	Rate	2021	296.8	365.4	235.7
Incidence	Federal Republic of Germany	Male	Age-standardized	Edentulism	Rate	2022	271.1	337.4	214.2
Incidence	Federal Republic of Germany	Female	Age-standardized	Edentulism	Rate	2022	324.9	396.2	260.7
Incidence	Federal Republic of Germany	Both	Age-standardized	Edentulism	Rate	2022	297.5	365.9	238.3
Incidence	Federal Republic of Germany	Male	Age-standardized	Edentulism	Rate	2023	270.0	335.9	213.2
Incidence	Federal Republic of Germany	Female	Age-standardized	Edentulism	Rate	2023	323.3	394.2	259.5
Incidence	Federal Republic of Germany	Both	Age-standardized	Edentulism	Rate	2023	296.1	364.7	237.2
Incidence	People's Republic of China	Male	Age-standardized	Edentulism	Rate	1990	280.6	345.1	221.9
Incidence	People's Republic of China	Female	Age-standardized	Edentulism	Rate	1990	329.9	403.3	264.8
Incidence	People's Republic of China	Both	Age-standardized	Edentulism	Rate	1990	303.6	373.7	242.2
Incidence	People's Republic of China	Male	Age-standardized	Edentulism	Rate	1991	270.8	333.6	213.5
Incidence	People's Republic of China	Female	Age-standardized	Edentulism	Rate	1991	325.3	398.0	261.8
Incidence	People's Republic of China	Both	Age-standardized	Edentulism	Rate	1991	296.4	365.5	235.9
Incidence	People's Republic of China	Male	Age-standardized	Edentulism	Rate	1992	261.9	323.0	207.0
Incidence	People's Republic of China	Female	Age-standardized	Edentulism	Rate	1992	319.3	391.1	256.6
Incidence	People's Republic of China	Both	Age-standardized	Edentulism	Rate	1992	288.9	356.8	229.4
Incidence	People's Republic of China	Male	Age-standardized	Edentulism	Rate	1993	254.3	313.8	200.6
Incidence	People's Republic of China	Female	Age-standardized	Edentulism	Rate	1993	312.4	383.4	250.2
Incidence	People's Republic of China	Both	Age-standardized	Edentulism	Rate	1993	281.7	348.1	223.2
Incidence	People's Republic of China	Male	Age-standardized	Edentulism	Rate	1994	248.6	307.0	194.8
Incidence	People's Republic of China	Female	Age-standardized	Edentulism	Rate	1994	305.0	375.4	243.7
Incidence	People's Republic	Both	Age-standardized	Edentulism	Rate	1994	275.1	340.1	217.6

	of China								
Incidence	People's Republic of China	Male	Age-standardized	Edentulism	Rate	1995	245.2	303.3	190.7
Incidence	People's Republic of China	Female	Age-standardized	Edentulism	Rate	1995	297.4	366.1	238.2
Incidence	People's Republic of China	Both	Age-standardized	Edentulism	Rate	1995	269.7	333.5	213.1
Incidence	People's Republic of China	Male	Age-standardized	Edentulism	Rate	1996	244.1	300.7	190.2
Incidence	People's Republic of China	Female	Age-standardized	Edentulism	Rate	1996	286.9	353.6	229.6
Incidence	People's Republic of China	Both	Age-standardized	Edentulism	Rate	1996	263.9	325.6	208.7
Incidence	People's Republic of China	Male	Age-standardized	Edentulism	Rate	1997	244.3	301.0	190.9
Incidence	People's Republic of China	Female	Age-standardized	Edentulism	Rate	1997	273.1	336.9	217.6
Incidence	People's Republic of China	Both	Age-standardized	Edentulism	Rate	1997	257.2	316.1	203.7
Incidence	People's Republic of China	Male	Age-standardized	Edentulism	Rate	1998	245.4	303.2	192.4
Incidence	People's Republic of China	Female	Age-standardized	Edentulism	Rate	1998	259.3	319.7	207.2
Incidence	People's Republic of China	Both	Age-standardized	Edentulism	Rate	1998	251.1	307.7	198.7
Incidence	People's Republic of China	Male	Age-standardized	Edentulism	Rate	1999	246.8	305.5	193.5
Incidence	People's Republic of China	Female	Age-standardized	Edentulism	Rate	1999	249.1	306.4	197.8
Incidence	People's Republic of China	Both	Age-standardized	Edentulism	Rate	1999	246.8	303.2	194.6
Incidence	People's Republic of China	Male	Age-standardized	Edentulism	Rate	2000	247.8	307.0	194.3
Incidence	People's Republic of China	Female	Age-standardized	Edentulism	Rate	2000	245.5	302.2	194.4
Incidence	People's Republic of China	Both	Age-standardized	Edentulism	Rate	2000	245.6	302.0	193.3
Incidence	People's Republic of China	Male	Age-standardized	Edentulism	Rate	2001	249.4	308.3	196.0
Incidence	People's Republic of China	Female	Age-standardized	Edentulism	Rate	2001	250.3	307.8	198.6
Incidence	People's Republic of China	Both	Age-standardized	Edentulism	Rate	2001	248.8	305.8	196.4
Incidence	People's Republic	Male	Age-standardized	Edentulism	Rate	2002	252.4	311.3	199.0

	of China								
Incidence	People's Republic of China	Female	Age-standardized	Edentulism	Rate	2002	260.6	320.5	207.5
Incidence	People's Republic of China	Both	Age-standardized	Edentulism	Rate	2002	255.6	314.0	202.4
Incidence	People's Republic of China	Male	Age-standardized	Edentulism	Rate	2003	256.0	314.7	202.3
Incidence	People's Republic of China	Female	Age-standardized	Edentulism	Rate	2003	273.1	335.2	218.3
Incidence	People's Republic of China	Both	Age-standardized	Edentulism	Rate	2003	263.8	324.0	209.6
Incidence	People's Republic of China	Male	Age-standardized	Edentulism	Rate	2004	259.1	317.9	205.3
Incidence	People's Republic of China	Female	Age-standardized	Edentulism	Rate	2004	284.4	349.6	228.0
Incidence	People's Republic of China	Both	Age-standardized	Edentulism	Rate	2004	271.0	333.0	215.9
Incidence	People's Republic of China	Male	Age-standardized	Edentulism	Rate	2005	260.9	319.7	206.9
Incidence	People's Republic of China	Female	Age-standardized	Edentulism	Rate	2005	291.0	358.2	233.7
Incidence	People's Republic of China	Both	Age-standardized	Edentulism	Rate	2005	275.2	338.0	219.6
Incidence	People's Republic of China	Male	Age-standardized	Edentulism	Rate	2006	262.2	321.4	208.1
Incidence	People's Republic of China	Female	Age-standardized	Edentulism	Rate	2006	294.4	362.0	236.9
Incidence	People's Republic of China	Both	Age-standardized	Edentulism	Rate	2006	277.6	340.9	221.8
Incidence	People's Republic of China	Male	Age-standardized	Edentulism	Rate	2007	264.1	323.9	209.9
Incidence	People's Republic of China	Female	Age-standardized	Edentulism	Rate	2007	298.0	365.8	240.4
Incidence	People's Republic of China	Both	Age-standardized	Edentulism	Rate	2007	280.4	344.1	224.3
Incidence	People's Republic of China	Male	Age-standardized	Edentulism	Rate	2008	266.0	326.4	211.5
Incidence	People's Republic of China	Female	Age-standardized	Edentulism	Rate	2008	301.1	369.0	243.7
Incidence	People's Republic of China	Both	Age-standardized	Edentulism	Rate	2008	283.0	347.0	226.7
Incidence	People's Republic of China	Male	Age-standardized	Edentulism	Rate	2009	267.3	328.1	212.6
Incidence	People's Republic	Female	Age-standardized	Edentulism	Rate	2009	303.2	371.0	246.0

	of China								
Incidence	People's Republic of China	Both	Age-standardized	Edentulism	Rate	2009	284.8	349.0	228.4
Incidence	People's Republic of China	Male	Age-standardized	Edentulism	Rate	2010	267.2	328.4	212.6
Incidence	People's Republic of China	Female	Age-standardized	Edentulism	Rate	2010	303.7	371.5	247.0
Incidence	People's Republic of China	Both	Age-standardized	Edentulism	Rate	2010	285.0	349.2	228.9
Incidence	People's Republic of China	Male	Age-standardized	Edentulism	Rate	2011	260.7	319.9	207.8
Incidence	People's Republic of China	Female	Age-standardized	Edentulism	Rate	2011	297.1	362.8	241.3
Incidence	People's Republic of China	Both	Age-standardized	Edentulism	Rate	2011	278.5	340.7	224.0
Incidence	People's Republic of China	Male	Age-standardized	Edentulism	Rate	2012	246.5	302.5	196.9
Incidence	People's Republic of China	Female	Age-standardized	Edentulism	Rate	2012	282.4	344.3	229.3
Incidence	People's Republic of China	Both	Age-standardized	Edentulism	Rate	2012	264.0	322.7	212.9
Incidence	People's Republic of China	Male	Age-standardized	Edentulism	Rate	2013	229.9	282.4	183.9
Incidence	People's Republic of China	Female	Age-standardized	Edentulism	Rate	2013	265.0	322.7	215.5
Incidence	People's Republic of China	Both	Age-standardized	Edentulism	Rate	2013	247.0	301.8	199.5
Incidence	People's Republic of China	Male	Age-standardized	Edentulism	Rate	2014	216.1	265.7	173.0
Incidence	People's Republic of China	Female	Age-standardized	Edentulism	Rate	2014	250.3	304.4	204.0
Incidence	People's Republic of China	Both	Age-standardized	Edentulism	Rate	2014	232.8	284.3	188.3
Incidence	People's Republic of China	Male	Age-standardized	Edentulism	Rate	2015	210.3	258.2	168.7
Incidence	People's Republic of China	Female	Age-standardized	Edentulism	Rate	2015	243.9	296.3	199.1
Incidence	People's Republic of China	Both	Age-standardized	Edentulism	Rate	2015	226.7	276.4	183.8
Incidence	People's Republic of China	Male	Age-standardized	Edentulism	Rate	2016	218.9	266.4	177.0
Incidence	People's Republic of China	Female	Age-standardized	Edentulism	Rate	2016	253.8	306.9	207.7
Incidence	People's Republic	Both	Age-standardized	Edentulism	Rate	2016	235.9	285.6	192.6

	of China								
Incidence	People's Republic of China	Male	Age-standardized	Edentulism	Rate	2017	238.1	288.4	194.2
Incidence	People's Republic of China	Female	Age-standardized	Edentulism	Rate	2017	276.3	332.1	225.6
Incidence	People's Republic of China	Both	Age-standardized	Edentulism	Rate	2017	256.8	310.3	210.1
Incidence	People's Republic of China	Male	Age-standardized	Edentulism	Rate	2018	257.7	310.8	210.3
Incidence	People's Republic of China	Female	Age-standardized	Edentulism	Rate	2018	299.5	357.4	245.9
Incidence	People's Republic of China	Both	Age-standardized	Edentulism	Rate	2018	278.3	333.6	227.2
Incidence	People's Republic of China	Male	Age-standardized	Edentulism	Rate	2019	267.6	318.8	219.2
Incidence	People's Republic of China	Female	Age-standardized	Edentulism	Rate	2019	311.3	369.1	256.8
Incidence	People's Republic of China	Both	Age-standardized	Edentulism	Rate	2019	289.1	343.2	238.1
Incidence	People's Republic of China	Male	Age-standardized	Edentulism	Rate	2020	268.3	319.9	220.1
Incidence	People's Republic of China	Female	Age-standardized	Edentulism	Rate	2020	313.1	370.6	258.4
Incidence	People's Republic of China	Both	Age-standardized	Edentulism	Rate	2020	290.4	344.8	238.9
Incidence	People's Republic of China	Male	Age-standardized	Edentulism	Rate	2021	267.4	319.0	219.7
Incidence	People's Republic of China	Female	Age-standardized	Edentulism	Rate	2021	311.4	369.0	256.0
Incidence	People's Republic of China	Both	Age-standardized	Edentulism	Rate	2021	289.0	343.6	237.6
Incidence	People's Republic of China	Male	Age-standardized	Edentulism	Rate	2022	268.4	320.2	220.6
Incidence	People's Republic of China	Female	Age-standardized	Edentulism	Rate	2022	311.9	368.0	257.5
Incidence	People's Republic of China	Both	Age-standardized	Edentulism	Rate	2022	289.8	343.6	238.9
Incidence	People's Republic of China	Male	Age-standardized	Edentulism	Rate	2023	264.8	315.9	217.5
Incidence	People's Republic of China	Female	Age-standardized	Edentulism	Rate	2023	307.1	362.4	253.4
Incidence	People's Republic of China	Both	Age-standardized	Edentulism	Rate	2023	285.6	338.6	235.3
Incidence	Republic of Italy	Male	Age-standardized	Edentulism	Rate	1990	278.9	344.3	222.3

Incidence	Republic of Italy	Female	Age-standardized	Edentulism	Rate	1990	331.6	403.9	269.2
Incidence	Republic of Italy	Both	Age-standardized	Edentulism	Rate	1990	305.2	374.2	246.2
Incidence	Republic of Italy	Male	Age-standardized	Edentulism	Rate	1991	277.8	343.0	221.4
Incidence	Republic of Italy	Female	Age-standardized	Edentulism	Rate	1991	330.5	402.6	268.6
Incidence	Republic of Italy	Both	Age-standardized	Edentulism	Rate	1991	304.2	372.6	245.1
Incidence	Republic of Italy	Male	Age-standardized	Edentulism	Rate	1992	276.8	342.1	220.3
Incidence	Republic of Italy	Female	Age-standardized	Edentulism	Rate	1992	329.4	401.3	267.8
Incidence	Republic of Italy	Both	Age-standardized	Edentulism	Rate	1992	303.1	371.4	244.0
Incidence	Republic of Italy	Male	Age-standardized	Edentulism	Rate	1993	276.0	340.9	219.2
Incidence	Republic of Italy	Female	Age-standardized	Edentulism	Rate	1993	328.6	400.0	267.0
Incidence	Republic of Italy	Both	Age-standardized	Edentulism	Rate	1993	302.2	370.4	243.0
Incidence	Republic of Italy	Male	Age-standardized	Edentulism	Rate	1994	275.3	340.1	218.3
Incidence	Republic of Italy	Female	Age-standardized	Edentulism	Rate	1994	327.9	399.1	266.1
Incidence	Republic of Italy	Both	Age-standardized	Edentulism	Rate	1994	301.5	369.6	242.1
Incidence	Republic of Italy	Male	Age-standardized	Edentulism	Rate	1995	275.0	339.6	217.6
Incidence	Republic of Italy	Female	Age-standardized	Edentulism	Rate	1995	327.4	398.4	265.3
Incidence	Republic of Italy	Both	Age-standardized	Edentulism	Rate	1995	301.1	369.2	241.5
Incidence	Republic of Italy	Male	Age-standardized	Edentulism	Rate	1996	274.7	339.3	217.7
Incidence	Republic of Italy	Female	Age-standardized	Edentulism	Rate	1996	327.1	398.2	265.0
Incidence	Republic of Italy	Both	Age-standardized	Edentulism	Rate	1996	300.7	368.5	241.8
Incidence	Republic of Italy	Male	Age-standardized	Edentulism	Rate	1997	274.4	338.9	217.7
Incidence	Republic of Italy	Female	Age-standardized	Edentulism	Rate	1997	326.7	397.7	264.6
Incidence	Republic of Italy	Both	Age-standardized	Edentulism	Rate	1997	300.4	367.6	242.0
Incidence	Republic of Italy	Male	Age-standardized	Edentulism	Rate	1998	274.1	338.2	217.9
Incidence	Republic of Italy	Female	Age-standardized	Edentulism	Rate	1998	326.3	397.3	264.1
Incidence	Republic of Italy	Both	Age-standardized	Edentulism	Rate	1998	300.0	367.2	242.3
Incidence	Republic of Italy	Male	Age-standardized	Edentulism	Rate	1999	274.0	337.7	218.3
Incidence	Republic of Italy	Female	Age-standardized	Edentulism	Rate	1999	325.9	397.1	263.7
Incidence	Republic of Italy	Both	Age-standardized	Edentulism	Rate	1999	299.7	367.1	242.5
Incidence	Republic of Italy	Male	Age-standardized	Edentulism	Rate	2000	274.1	337.6	218.9
Incidence	Republic of Italy	Female	Age-standardized	Edentulism	Rate	2000	325.6	397.2	263.4
Incidence	Republic of Italy	Both	Age-standardized	Edentulism	Rate	2000	299.5	366.7	242.3
Incidence	Republic of Italy	Male	Age-standardized	Edentulism	Rate	2001	281.2	344.9	224.8
Incidence	Republic of Italy	Female	Age-standardized	Edentulism	Rate	2001	324.2	394.9	263.5
Incidence	Republic of Italy	Both	Age-standardized	Edentulism	Rate	2001	302.1	369.6	244.9
Incidence	Republic of Italy	Male	Age-standardized	Edentulism	Rate	2002	297.6	364.4	238.5
Incidence	Republic of Italy	Female	Age-standardized	Edentulism	Rate	2002	321.1	390.8	261.2
Incidence	Republic of Italy	Both	Age-standardized	Edentulism	Rate	2002	308.3	376.5	248.7
Incidence	Republic of Italy	Male	Age-standardized	Edentulism	Rate	2003	317.2	386.4	254.9
Incidence	Republic of Italy	Female	Age-standardized	Edentulism	Rate	2003	317.4	387.1	255.9
Incidence	Republic of Italy	Both	Age-standardized	Edentulism	Rate	2003	315.7	385.3	253.0
Incidence	Republic of Italy	Male	Age-standardized	Edentulism	Rate	2004	333.5	406.2	268.6
Incidence	Republic of Italy	Female	Age-standardized	Edentulism	Rate	2004	313.7	382.2	250.5
Incidence	Republic of Italy	Both	Age-standardized	Edentulism	Rate	2004	321.6	392.6	256.9

Incidence	Republic of Italy	Male	Age-standardized	Edentulism	Rate	2005	340.3	414.9	274.2
Incidence	Republic of Italy	Female	Age-standardized	Edentulism	Rate	2005	311.1	380.2	247.2
Incidence	Republic of Italy	Both	Age-standardized	Edentulism	Rate	2005	323.5	395.3	259.0
Incidence	Republic of Italy	Male	Age-standardized	Edentulism	Rate	2006	323.7	395.2	260.1
Incidence	Republic of Italy	Female	Age-standardized	Edentulism	Rate	2006	303.8	371.9	241.1
Incidence	Republic of Italy	Both	Age-standardized	Edentulism	Rate	2006	311.9	382.3	248.9
Incidence	Republic of Italy	Male	Age-standardized	Edentulism	Rate	2007	284.3	348.4	227.8
Incidence	Republic of Italy	Female	Age-standardized	Edentulism	Rate	2007	289.3	355.0	230.4
Incidence	Republic of Italy	Both	Age-standardized	Edentulism	Rate	2007	285.6	350.9	227.4
Incidence	Republic of Italy	Male	Age-standardized	Edentulism	Rate	2008	237.2	293.1	189.8
Incidence	Republic of Italy	Female	Age-standardized	Edentulism	Rate	2008	273.0	336.2	218.2
Incidence	Republic of Italy	Both	Age-standardized	Edentulism	Rate	2008	254.7	313.7	202.2
Incidence	Republic of Italy	Male	Age-standardized	Edentulism	Rate	2009	197.8	246.3	158.0
Incidence	Republic of Italy	Female	Age-standardized	Edentulism	Rate	2009	260.3	322.5	207.5
Incidence	Republic of Italy	Both	Age-standardized	Edentulism	Rate	2009	229.3	284.7	182.5
Incidence	Republic of Italy	Male	Age-standardized	Edentulism	Rate	2010	181.3	227.1	144.2
Incidence	Republic of Italy	Female	Age-standardized	Edentulism	Rate	2010	256.9	319.3	203.2
Incidence	Republic of Italy	Both	Age-standardized	Edentulism	Rate	2010	219.6	274.1	174.4
Incidence	Republic of Italy	Male	Age-standardized	Edentulism	Rate	2011	190.6	237.9	151.6
Incidence	Republic of Italy	Female	Age-standardized	Edentulism	Rate	2011	265.7	328.4	211.2
Incidence	Republic of Italy	Both	Age-standardized	Edentulism	Rate	2011	228.6	284.1	182.1
Incidence	Republic of Italy	Male	Age-standardized	Edentulism	Rate	2012	212.9	265.9	169.0
Incidence	Republic of Italy	Female	Age-standardized	Edentulism	Rate	2012	282.4	347.0	225.8
Incidence	Republic of Italy	Both	Age-standardized	Edentulism	Rate	2012	247.9	306.5	198.4
Incidence	Republic of Italy	Male	Age-standardized	Edentulism	Rate	2013	239.5	298.5	189.8
Incidence	Republic of Italy	Female	Age-standardized	Edentulism	Rate	2013	301.4	368.9	242.4
Incidence	Republic of Italy	Both	Age-standardized	Edentulism	Rate	2013	270.5	332.9	217.6
Incidence	Republic of Italy	Male	Age-standardized	Edentulism	Rate	2014	261.9	326.1	208.0
Incidence	Republic of Italy	Female	Age-standardized	Edentulism	Rate	2014	317.2	386.8	256.4
Incidence	Republic of Italy	Both	Age-standardized	Edentulism	Rate	2014	289.5	355.0	233.8
Incidence	Republic of Italy	Male	Age-standardized	Edentulism	Rate	2015	271.7	338.0	216.3
Incidence	Republic of Italy	Female	Age-standardized	Edentulism	Rate	2015	324.1	393.6	262.2
Incidence	Republic of Italy	Both	Age-standardized	Edentulism	Rate	2015	297.7	364.3	241.2
Incidence	Republic of Italy	Male	Age-standardized	Edentulism	Rate	2016	271.9	338.3	216.9
Incidence	Republic of Italy	Female	Age-standardized	Edentulism	Rate	2016	324.3	394.3	262.6
Incidence	Republic of Italy	Both	Age-standardized	Edentulism	Rate	2016	297.9	365.2	241.3
Incidence	Republic of Italy	Male	Age-standardized	Edentulism	Rate	2017	271.8	337.9	216.9
Incidence	Republic of Italy	Female	Age-standardized	Edentulism	Rate	2017	324.0	395.4	262.2
Incidence	Republic of Italy	Both	Age-standardized	Edentulism	Rate	2017	297.7	365.9	241.1
Incidence	Republic of Italy	Male	Age-standardized	Edentulism	Rate	2018	271.5	337.4	216.5
Incidence	Republic of Italy	Female	Age-standardized	Edentulism	Rate	2018	323.6	396.6	261.6
Incidence	Republic of Italy	Both	Age-standardized	Edentulism	Rate	2018	297.4	366.3	240.4
Incidence	Republic of Italy	Male	Age-standardized	Edentulism	Rate	2019	271.3	336.9	216.2
Incidence	Republic of Italy	Female	Age-standardized	Edentulism	Rate	2019	323.4	398.2	261.0

Incidence	Republic of Italy	Both	Age-standardized	Edentulism	Rate	2019	297.2	367.0	239.6
Incidence	Republic of Italy	Male	Age-standardized	Edentulism	Rate	2020	271.3	336.4	215.1
Incidence	Republic of Italy	Female	Age-standardized	Edentulism	Rate	2020	323.9	394.8	263.0
Incidence	Republic of Italy	Both	Age-standardized	Edentulism	Rate	2020	297.5	365.4	239.4
Incidence	Republic of Italy	Male	Age-standardized	Edentulism	Rate	2021	271.1	337.0	216.0
Incidence	Republic of Italy	Female	Age-standardized	Edentulism	Rate	2021	323.9	396.2	260.8
Incidence	Republic of Italy	Both	Age-standardized	Edentulism	Rate	2021	297.4	365.5	240.7
Incidence	Republic of Italy	Male	Age-standardized	Edentulism	Rate	2022	272.3	336.8	216.7
Incidence	Republic of Italy	Female	Age-standardized	Edentulism	Rate	2022	324.3	396.4	262.5
Incidence	Republic of Italy	Both	Age-standardized	Edentulism	Rate	2022	298.1	366.9	240.1
Incidence	Republic of Italy	Male	Age-standardized	Edentulism	Rate	2023	270.7	334.8	215.4
Incidence	Republic of Italy	Female	Age-standardized	Edentulism	Rate	2023	322.1	393.7	260.8
Incidence	Republic of Italy	Both	Age-standardized	Edentulism	Rate	2023	296.2	364.5	238.6
Incidence	Japan	Male	Age-standardized	Edentulism	Rate	1990	297.2	366.4	238.7
Incidence	Japan	Female	Age-standardized	Edentulism	Rate	1990	270.4	331.0	212.1
Incidence	Japan	Both	Age-standardized	Edentulism	Rate	1990	286.2	347.1	229.8
Incidence	Japan	Male	Age-standardized	Edentulism	Rate	1991	278.8	344.4	224.1
Incidence	Japan	Female	Age-standardized	Edentulism	Rate	1991	250.3	306.0	195.9
Incidence	Japan	Both	Age-standardized	Edentulism	Rate	1991	266.6	323.4	214.1
Incidence	Japan	Male	Age-standardized	Edentulism	Rate	1992	266.0	328.9	213.8
Incidence	Japan	Female	Age-standardized	Edentulism	Rate	1992	232.8	284.6	183.0
Incidence	Japan	Both	Age-standardized	Edentulism	Rate	1992	251.0	307.1	201.3
Incidence	Japan	Male	Age-standardized	Edentulism	Rate	1993	257.9	319.2	207.0
Incidence	Japan	Female	Age-standardized	Edentulism	Rate	1993	218.8	268.3	172.1
Incidence	Japan	Both	Age-standardized	Edentulism	Rate	1993	239.7	293.8	191.6
Incidence	Japan	Male	Age-standardized	Edentulism	Rate	1994	253.6	314.6	203.1
Incidence	Japan	Female	Age-standardized	Edentulism	Rate	1994	209.5	258.3	164.8
Incidence	Japan	Both	Age-standardized	Edentulism	Rate	1994	232.7	285.9	185.3
Incidence	Japan	Male	Age-standardized	Edentulism	Rate	1995	252.2	313.7	201.4
Incidence	Japan	Female	Age-standardized	Edentulism	Rate	1995	206.1	255.1	161.8
Incidence	Japan	Both	Age-standardized	Edentulism	Rate	1995	230.3	283.7	182.5
Incidence	Japan	Male	Age-standardized	Edentulism	Rate	1996	263.8	327.2	212.0
Incidence	Japan	Female	Age-standardized	Edentulism	Rate	1996	207.0	256.1	162.6
Incidence	Japan	Both	Age-standardized	Edentulism	Rate	1996	236.4	291.4	187.4
Incidence	Japan	Male	Age-standardized	Edentulism	Rate	1997	291.9	360.1	235.7
Incidence	Japan	Female	Age-standardized	Edentulism	Rate	1997	209.4	258.9	164.5
Incidence	Japan	Both	Age-standardized	Edentulism	Rate	1997	251.6	311.6	199.5
Incidence	Japan	Male	Age-standardized	Edentulism	Rate	1998	326.2	400.4	264.3
Incidence	Japan	Female	Age-standardized	Edentulism	Rate	1998	212.7	262.9	167.2
Incidence	Japan	Both	Age-standardized	Edentulism	Rate	1998	270.3	333.8	214.6
Incidence	Japan	Male	Age-standardized	Edentulism	Rate	1999	355.8	437.1	289.4
Incidence	Japan	Female	Age-standardized	Edentulism	Rate	1999	216.5	267.4	170.3
Incidence	Japan	Both	Age-standardized	Edentulism	Rate	1999	287.0	353.6	228.0
Incidence	Japan	Male	Age-standardized	Edentulism	Rate	2000	370.3	454.6	302.3

Incidence	Japan	Female	Age-standardized	Edentulism	Rate	2000	220.2	271.8	173.4
Incidence	Japan	Both	Age-standardized	Edentulism	Rate	2000	296.1	363.5	236.1
Incidence	Japan	Male	Age-standardized	Edentulism	Rate	2001	376.9	458.7	307.6
Incidence	Japan	Female	Age-standardized	Edentulism	Rate	2001	226.6	277.5	178.5
Incidence	Japan	Both	Age-standardized	Edentulism	Rate	2001	302.9	369.9	242.5
Incidence	Japan	Male	Age-standardized	Edentulism	Rate	2002	387.2	467.7	315.7
Incidence	Japan	Female	Age-standardized	Edentulism	Rate	2002	237.1	287.9	186.0
Incidence	Japan	Both	Age-standardized	Edentulism	Rate	2002	313.8	380.9	253.9
Incidence	Japan	Male	Age-standardized	Edentulism	Rate	2003	397.2	477.7	325.9
Incidence	Japan	Female	Age-standardized	Edentulism	Rate	2003	248.4	299.7	194.2
Incidence	Japan	Both	Age-standardized	Edentulism	Rate	2003	325.0	391.2	265.0
Incidence	Japan	Male	Age-standardized	Edentulism	Rate	2004	403.2	483.0	330.3
Incidence	Japan	Female	Age-standardized	Edentulism	Rate	2004	257.6	308.0	202.9
Incidence	Japan	Both	Age-standardized	Edentulism	Rate	2004	332.9	397.8	272.4
Incidence	Japan	Male	Age-standardized	Edentulism	Rate	2005	401.2	479.5	328.3
Incidence	Japan	Female	Age-standardized	Edentulism	Rate	2005	261.5	309.9	206.2
Incidence	Japan	Both	Age-standardized	Edentulism	Rate	2005	334.0	397.9	274.5
Incidence	Japan	Male	Age-standardized	Edentulism	Rate	2006	381.4	456.8	311.2
Incidence	Japan	Female	Age-standardized	Edentulism	Rate	2006	257.8	307.7	203.8
Incidence	Japan	Both	Age-standardized	Edentulism	Rate	2006	321.9	384.5	263.7
Incidence	Japan	Male	Age-standardized	Edentulism	Rate	2007	343.7	412.5	280.3
Incidence	Japan	Female	Age-standardized	Edentulism	Rate	2007	248.1	296.7	195.8
Incidence	Japan	Both	Age-standardized	Edentulism	Rate	2007	297.8	356.1	243.1
Incidence	Japan	Male	Age-standardized	Edentulism	Rate	2008	298.9	361.6	244.7
Incidence	Japan	Female	Age-standardized	Edentulism	Rate	2008	235.1	283.2	184.8
Incidence	Japan	Both	Age-standardized	Edentulism	Rate	2008	268.4	322.9	218.6
Incidence	Japan	Male	Age-standardized	Edentulism	Rate	2009	257.9	314.0	209.1
Incidence	Japan	Female	Age-standardized	Edentulism	Rate	2009	221.5	269.6	174.8
Incidence	Japan	Both	Age-standardized	Edentulism	Rate	2009	240.6	291.4	195.6
Incidence	Japan	Male	Age-standardized	Edentulism	Rate	2010	231.5	283.3	186.4
Incidence	Japan	Female	Age-standardized	Edentulism	Rate	2010	210.2	257.9	166.2
Incidence	Japan	Both	Age-standardized	Edentulism	Rate	2010	221.5	269.8	178.3
Incidence	Japan	Male	Age-standardized	Edentulism	Rate	2011	215.4	262.7	173.5
Incidence	Japan	Female	Age-standardized	Edentulism	Rate	2011	198.8	244.7	157.1
Incidence	Japan	Both	Age-standardized	Edentulism	Rate	2011	207.7	253.7	166.6
Incidence	Japan	Male	Age-standardized	Edentulism	Rate	2012	199.5	242.9	160.4
Incidence	Japan	Female	Age-standardized	Edentulism	Rate	2012	184.8	228.4	146.5
Incidence	Japan	Both	Age-standardized	Edentulism	Rate	2012	192.9	235.8	154.3
Incidence	Japan	Male	Age-standardized	Edentulism	Rate	2013	185.8	226.0	149.1
Incidence	Japan	Female	Age-standardized	Edentulism	Rate	2013	171.2	210.6	137.3
Incidence	Japan	Both	Age-standardized	Edentulism	Rate	2013	179.5	219.1	144.2
Incidence	Japan	Male	Age-standardized	Edentulism	Rate	2014	176.0	214.2	142.2
Incidence	Japan	Female	Age-standardized	Edentulism	Rate	2014	160.9	196.8	130.6
Incidence	Japan	Both	Age-standardized	Edentulism	Rate	2014	169.6	206.1	137.5

Incidence	Japan	Male	Age-standardized	Edentulism	Rate	2015	172.1	209.5	139.9
Incidence	Japan	Female	Age-standardized	Edentulism	Rate	2015	156.8	191.2	126.9
Incidence	Japan	Both	Age-standardized	Edentulism	Rate	2015	165.7	200.2	135.1
Incidence	Japan	Male	Age-standardized	Edentulism	Rate	2016	172.3	209.5	140.1
Incidence	Japan	Female	Age-standardized	Edentulism	Rate	2016	157.4	191.8	127.6
Incidence	Japan	Both	Age-standardized	Edentulism	Rate	2016	166.1	200.4	135.5
Incidence	Japan	Male	Age-standardized	Edentulism	Rate	2017	173.9	211.2	141.4
Incidence	Japan	Female	Age-standardized	Edentulism	Rate	2017	159.2	193.8	129.2
Incidence	Japan	Both	Age-standardized	Edentulism	Rate	2017	167.8	202.1	137.0
Incidence	Japan	Male	Age-standardized	Edentulism	Rate	2018	176.9	214.6	144.5
Incidence	Japan	Female	Age-standardized	Edentulism	Rate	2018	161.8	197.0	131.5
Incidence	Japan	Both	Age-standardized	Edentulism	Rate	2018	170.7	205.3	139.5
Incidence	Japan	Male	Age-standardized	Edentulism	Rate	2019	181.3	219.7	148.2
Incidence	Japan	Female	Age-standardized	Edentulism	Rate	2019	165.3	200.6	134.5
Incidence	Japan	Both	Age-standardized	Edentulism	Rate	2019	174.6	210.2	143.0
Incidence	Japan	Male	Age-standardized	Edentulism	Rate	2020	252.5	304.3	206.4
Incidence	Japan	Female	Age-standardized	Edentulism	Rate	2020	208.3	249.9	167.6
Incidence	Japan	Both	Age-standardized	Edentulism	Rate	2020	231.1	279.0	188.2
Incidence	Japan	Male	Age-standardized	Edentulism	Rate	2021	254.1	307.1	208.3
Incidence	Japan	Female	Age-standardized	Edentulism	Rate	2021	209.4	250.9	169.5
Incidence	Japan	Both	Age-standardized	Edentulism	Rate	2021	232.5	279.9	190.4
Incidence	Japan	Male	Age-standardized	Edentulism	Rate	2022	255.4	305.2	211.4
Incidence	Japan	Female	Age-standardized	Edentulism	Rate	2022	211.8	252.8	172.5
Incidence	Japan	Both	Age-standardized	Edentulism	Rate	2022	234.3	279.8	192.7
Incidence	Japan	Male	Age-standardized	Edentulism	Rate	2023	254.3	303.8	210.5
Incidence	Japan	Female	Age-standardized	Edentulism	Rate	2023	210.8	251.6	171.7
Incidence	Japan	Both	Age-standardized	Edentulism	Rate	2023	233.3	278.5	191.9
Incidence	Russian Federation	Male	Age-standardized	Edentulism	Rate	1990	390.2	467.4	323.1
Incidence	Russian Federation	Female	Age-standardized	Edentulism	Rate	1990	430.2	509.8	359.9
Incidence	Russian Federation	Both	Age-standardized	Edentulism	Rate	1990	417.2	496.0	349.1
Incidence	Russian Federation	Male	Age-standardized	Edentulism	Rate	1991	390.9	468.1	323.5
Incidence	Russian Federation	Female	Age-standardized	Edentulism	Rate	1991	431.3	511.3	360.8
Incidence	Russian Federation	Both	Age-standardized	Edentulism	Rate	1991	417.9	497.1	349.6
Incidence	Russian Federation	Male	Age-standardized	Edentulism	Rate	1992	391.8	469.1	324.1
Incidence	Russian Federation	Female	Age-standardized	Edentulism	Rate	1992	432.5	513.1	361.8
Incidence	Russian Federation	Both	Age-standardized	Edentulism	Rate	1992	418.7	498.3	350.1
Incidence	Russian Federation	Male	Age-standardized	Edentulism	Rate	1993	392.7	470.4	324.8
Incidence	Russian Federation	Female	Age-standardized	Edentulism	Rate	1993	433.7	514.9	362.8
Incidence	Russian Federation	Both	Age-standardized	Edentulism	Rate	1993	419.7	499.3	350.7
Incidence	Russian Federation	Male	Age-standardized	Edentulism	Rate	1994	393.8	471.2	325.5
Incidence	Russian Federation	Female	Age-standardized	Edentulism	Rate	1994	434.9	516.6	363.7
Incidence	Russian Federation	Both	Age-standardized	Edentulism	Rate	1994	420.6	500.2	351.4
Incidence	Russian Federation	Male	Age-standardized	Edentulism	Rate	1995	394.7	472.0	326.2
Incidence	Russian Federation	Female	Age-standardized	Edentulism	Rate	1995	435.9	518.1	364.4

Incidence	Russian Federation	Both	Age-standardized	Edentulism	Rate	1995	421.5	501.2	351.9
Incidence	Russian Federation	Male	Age-standardized	Edentulism	Rate	1996	396.2	473.9	327.4
Incidence	Russian Federation	Female	Age-standardized	Edentulism	Rate	1996	437.0	519.5	365.4
Incidence	Russian Federation	Both	Age-standardized	Edentulism	Rate	1996	422.6	502.4	353.1
Incidence	Russian Federation	Male	Age-standardized	Edentulism	Rate	1997	398.4	476.8	329.1
Incidence	Russian Federation	Female	Age-standardized	Edentulism	Rate	1997	438.6	521.2	366.6
Incidence	Russian Federation	Both	Age-standardized	Edentulism	Rate	1997	424.3	504.3	354.7
Incidence	Russian Federation	Male	Age-standardized	Edentulism	Rate	1998	400.9	479.7	331.0
Incidence	Russian Federation	Female	Age-standardized	Edentulism	Rate	1998	440.3	523.0	368.0
Incidence	Russian Federation	Both	Age-standardized	Edentulism	Rate	1998	426.1	506.5	356.4
Incidence	Russian Federation	Male	Age-standardized	Edentulism	Rate	1999	403.0	482.0	332.7
Incidence	Russian Federation	Female	Age-standardized	Edentulism	Rate	1999	441.8	524.8	369.2
Incidence	Russian Federation	Both	Age-standardized	Edentulism	Rate	1999	427.8	508.5	358.0
Incidence	Russian Federation	Male	Age-standardized	Edentulism	Rate	2000	404.3	483.3	333.8
Incidence	Russian Federation	Female	Age-standardized	Edentulism	Rate	2000	442.9	526.0	370.1
Incidence	Russian Federation	Both	Age-standardized	Edentulism	Rate	2000	428.9	509.5	359.2
Incidence	Russian Federation	Male	Age-standardized	Edentulism	Rate	2001	402.6	481.6	333.3
Incidence	Russian Federation	Female	Age-standardized	Edentulism	Rate	2001	441.6	523.8	368.8
Incidence	Russian Federation	Both	Age-standardized	Edentulism	Rate	2001	427.4	507.5	357.8
Incidence	Russian Federation	Male	Age-standardized	Edentulism	Rate	2002	397.1	475.5	330.7
Incidence	Russian Federation	Female	Age-standardized	Edentulism	Rate	2002	437.2	517.9	365.6
Incidence	Russian Federation	Both	Age-standardized	Edentulism	Rate	2002	422.6	501.4	354.3
Incidence	Russian Federation	Male	Age-standardized	Edentulism	Rate	2003	390.1	467.1	325.7
Incidence	Russian Federation	Female	Age-standardized	Edentulism	Rate	2003	431.7	509.4	361.8
Incidence	Russian Federation	Both	Age-standardized	Edentulism	Rate	2003	416.6	492.9	350.0
Incidence	Russian Federation	Male	Age-standardized	Edentulism	Rate	2004	383.6	459.3	321.0
Incidence	Russian Federation	Female	Age-standardized	Edentulism	Rate	2004	427.1	502.2	360.0
Incidence	Russian Federation	Both	Age-standardized	Edentulism	Rate	2004	411.2	485.0	347.6
Incidence	Russian Federation	Male	Age-standardized	Edentulism	Rate	2005	379.6	454.5	318.8
Incidence	Russian Federation	Female	Age-standardized	Edentulism	Rate	2005	425.0	499.0	357.9
Incidence	Russian Federation	Both	Age-standardized	Edentulism	Rate	2005	408.3	481.1	345.6
Incidence	Russian Federation	Male	Age-standardized	Edentulism	Rate	2006	376.9	448.6	320.2
Incidence	Russian Federation	Female	Age-standardized	Edentulism	Rate	2006	428.2	496.7	366.4
Incidence	Russian Federation	Both	Age-standardized	Edentulism	Rate	2006	408.4	476.6	349.0
Incidence	Russian Federation	Male	Age-standardized	Edentulism	Rate	2007	373.6	439.8	320.4
Incidence	Russian Federation	Female	Age-standardized	Edentulism	Rate	2007	435.8	499.3	378.3
Incidence	Russian Federation	Both	Age-standardized	Edentulism	Rate	2007	410.4	474.3	354.5
Incidence	Russian Federation	Male	Age-standardized	Edentulism	Rate	2008	370.3	434.2	320.3
Incidence	Russian Federation	Female	Age-standardized	Edentulism	Rate	2008	444.9	504.3	390.5
Incidence	Russian Federation	Both	Age-standardized	Edentulism	Rate	2008	413.3	473.4	362.0
Incidence	Russian Federation	Male	Age-standardized	Edentulism	Rate	2009	367.8	427.8	320.4
Incidence	Russian Federation	Female	Age-standardized	Edentulism	Rate	2009	452.5	508.0	401.2
Incidence	Russian Federation	Both	Age-standardized	Edentulism	Rate	2009	415.7	472.7	365.1
Incidence	Russian Federation	Male	Age-standardized	Edentulism	Rate	2010	366.6	423.9	319.1

Incidence	Russian Federation	Female	Age-standardized	Edentulism	Rate	2010	455.6	513.9	405.0
Incidence	Russian Federation	Both	Age-standardized	Edentulism	Rate	2010	416.5	470.5	367.9
Incidence	Russian Federation	Male	Age-standardized	Edentulism	Rate	2011	369.1	424.9	322.5
Incidence	Russian Federation	Female	Age-standardized	Edentulism	Rate	2011	456.3	516.1	403.9
Incidence	Russian Federation	Both	Age-standardized	Edentulism	Rate	2011	418.1	473.5	370.2
Incidence	Russian Federation	Male	Age-standardized	Edentulism	Rate	2012	375.6	433.1	328.9
Incidence	Russian Federation	Female	Age-standardized	Edentulism	Rate	2012	458.4	519.2	404.2
Incidence	Russian Federation	Both	Age-standardized	Edentulism	Rate	2012	422.4	478.6	374.8
Incidence	Russian Federation	Male	Age-standardized	Edentulism	Rate	2013	383.7	443.1	337.1
Incidence	Russian Federation	Female	Age-standardized	Edentulism	Rate	2013	461.0	523.3	405.4
Incidence	Russian Federation	Both	Age-standardized	Edentulism	Rate	2013	427.9	485.1	380.0
Incidence	Russian Federation	Male	Age-standardized	Edentulism	Rate	2014	390.7	450.4	344.2
Incidence	Russian Federation	Female	Age-standardized	Edentulism	Rate	2014	463.3	527.2	406.8
Incidence	Russian Federation	Both	Age-standardized	Edentulism	Rate	2014	432.7	493.6	381.3
Incidence	Russian Federation	Male	Age-standardized	Edentulism	Rate	2015	394.3	457.2	345.8
Incidence	Russian Federation	Female	Age-standardized	Edentulism	Rate	2015	464.5	531.9	407.1
Incidence	Russian Federation	Both	Age-standardized	Edentulism	Rate	2015	435.0	498.2	382.7
Incidence	Russian Federation	Male	Age-standardized	Edentulism	Rate	2016	395.3	458.8	345.7
Incidence	Russian Federation	Female	Age-standardized	Edentulism	Rate	2016	465.5	533.6	406.7
Incidence	Russian Federation	Both	Age-standardized	Edentulism	Rate	2016	435.8	499.1	381.0
Incidence	Russian Federation	Male	Age-standardized	Edentulism	Rate	2017	396.3	461.6	343.8
Incidence	Russian Federation	Female	Age-standardized	Edentulism	Rate	2017	467.2	536.6	405.8
Incidence	Russian Federation	Both	Age-standardized	Edentulism	Rate	2017	436.9	501.6	379.4
Incidence	Russian Federation	Male	Age-standardized	Edentulism	Rate	2018	397.0	464.3	341.3
Incidence	Russian Federation	Female	Age-standardized	Edentulism	Rate	2018	468.7	540.3	405.0
Incidence	Russian Federation	Both	Age-standardized	Edentulism	Rate	2018	438.0	505.8	378.4
Incidence	Russian Federation	Male	Age-standardized	Edentulism	Rate	2019	397.4	464.8	339.8
Incidence	Russian Federation	Female	Age-standardized	Edentulism	Rate	2019	469.4	543.2	403.8
Incidence	Russian Federation	Both	Age-standardized	Edentulism	Rate	2019	438.4	509.6	376.9
Incidence	Russian Federation	Male	Age-standardized	Edentulism	Rate	2020	397.5	467.1	336.5
Incidence	Russian Federation	Female	Age-standardized	Edentulism	Rate	2020	476.6	553.1	403.9
Incidence	Russian Federation	Both	Age-standardized	Edentulism	Rate	2020	442.0	514.2	375.9
Incidence	Russian Federation	Male	Age-standardized	Edentulism	Rate	2021	400.4	471.6	335.4
Incidence	Russian Federation	Female	Age-standardized	Edentulism	Rate	2021	483.9	568.2	404.0
Incidence	Russian Federation	Both	Age-standardized	Edentulism	Rate	2021	446.9	525.6	373.8
Incidence	Russian Federation	Male	Age-standardized	Edentulism	Rate	2022	402.2	484.3	332.4
Incidence	Russian Federation	Female	Age-standardized	Edentulism	Rate	2022	484.9	569.9	406.2
Incidence	Russian Federation	Both	Age-standardized	Edentulism	Rate	2022	448.3	532.3	373.7
Incidence	Russian Federation	Male	Age-standardized	Edentulism	Rate	2023	400.0	481.8	330.6
Incidence	Russian Federation	Female	Age-standardized	Edentulism	Rate	2023	481.9	566.6	403.7
Incidence	Russian Federation	Both	Age-standardized	Edentulism	Rate	2023	445.6	529.2	371.4
Incidence	Republic of Indonesia	Male	Age-standardized	Edentulism	Rate	1990	270.0	331.2	217.5
Incidence	Republic of	Female	Age-standardized	Edentulism	Rate	1990	378.8	458.3	310.3

	Indonesia								
Incidence	Republic of Indonesia	Both	Age-standardized	Edentulism	Rate	1990	325.3	395.1	265.4
Incidence	Republic of Indonesia	Male	Age-standardized	Edentulism	Rate	1991	268.0	329.0	215.8
Incidence	Republic of Indonesia	Female	Age-standardized	Edentulism	Rate	1991	376.7	456.3	308.4
Incidence	Republic of Indonesia	Both	Age-standardized	Edentulism	Rate	1991	323.2	392.4	263.4
Incidence	Republic of Indonesia	Male	Age-standardized	Edentulism	Rate	1992	266.3	326.9	214.5
Incidence	Republic of Indonesia	Female	Age-standardized	Edentulism	Rate	1992	375.1	454.3	306.4
Incidence	Republic of Indonesia	Both	Age-standardized	Edentulism	Rate	1992	321.6	390.3	261.8
Incidence	Republic of Indonesia	Male	Age-standardized	Edentulism	Rate	1993	265.1	325.0	213.5
Incidence	Republic of Indonesia	Female	Age-standardized	Edentulism	Rate	1993	373.7	452.5	304.6
Incidence	Republic of Indonesia	Both	Age-standardized	Edentulism	Rate	1993	320.3	389.2	260.7
Incidence	Republic of Indonesia	Male	Age-standardized	Edentulism	Rate	1994	264.0	325.0	212.6
Incidence	Republic of Indonesia	Female	Age-standardized	Edentulism	Rate	1994	372.6	451.2	303.6
Incidence	Republic of Indonesia	Both	Age-standardized	Edentulism	Rate	1994	319.2	387.7	259.8
Incidence	Republic of Indonesia	Male	Age-standardized	Edentulism	Rate	1995	263.2	323.7	211.8
Incidence	Republic of Indonesia	Female	Age-standardized	Edentulism	Rate	1995	371.5	450.0	302.1
Incidence	Republic of Indonesia	Both	Age-standardized	Edentulism	Rate	1995	318.3	386.4	259.0
Incidence	Republic of Indonesia	Male	Age-standardized	Edentulism	Rate	1996	261.4	322.7	210.6
Incidence	Republic of Indonesia	Female	Age-standardized	Edentulism	Rate	1996	370.2	445.9	303.4
Incidence	Republic of Indonesia	Both	Age-standardized	Edentulism	Rate	1996	316.7	383.5	258.5
Incidence	Republic of Indonesia	Male	Age-standardized	Edentulism	Rate	1997	258.3	320.0	207.8
Incidence	Republic of Indonesia	Female	Age-standardized	Edentulism	Rate	1997	368.7	441.8	304.4
Incidence	Republic of	Both	Age-standardized	Edentulism	Rate	1997	314.6	380.9	257.3

	Indonesia								
Incidence	Republic of Indonesia	Male	Age-standardized	Edentulism	Rate	1998	254.9	315.6	202.5
Incidence	Republic of Indonesia	Female	Age-standardized	Edentulism	Rate	1998	367.3	438.0	305.6
Incidence	Republic of Indonesia	Both	Age-standardized	Edentulism	Rate	1998	312.4	377.8	256.3
Incidence	Republic of Indonesia	Male	Age-standardized	Edentulism	Rate	1999	252.1	311.9	199.4
Incidence	Republic of Indonesia	Female	Age-standardized	Edentulism	Rate	1999	366.3	434.3	305.4
Incidence	Republic of Indonesia	Both	Age-standardized	Edentulism	Rate	1999	310.6	373.9	254.8
Incidence	Republic of Indonesia	Male	Age-standardized	Edentulism	Rate	2000	251.0	310.7	198.4
Incidence	Republic of Indonesia	Female	Age-standardized	Edentulism	Rate	2000	366.1	431.3	306.9
Incidence	Republic of Indonesia	Both	Age-standardized	Edentulism	Rate	2000	310.0	371.5	254.5
Incidence	Republic of Indonesia	Male	Age-standardized	Edentulism	Rate	2001	252.3	311.1	202.2
Incidence	Republic of Indonesia	Female	Age-standardized	Edentulism	Rate	2001	368.0	429.9	313.1
Incidence	Republic of Indonesia	Both	Age-standardized	Edentulism	Rate	2001	311.5	371.6	257.8
Incidence	Republic of Indonesia	Male	Age-standardized	Edentulism	Rate	2002	255.5	314.1	206.8
Incidence	Republic of Indonesia	Female	Age-standardized	Edentulism	Rate	2002	372.2	434.2	321.4
Incidence	Republic of Indonesia	Both	Age-standardized	Edentulism	Rate	2002	315.1	373.0	264.1
Incidence	Republic of Indonesia	Male	Age-standardized	Edentulism	Rate	2003	259.2	315.5	211.5
Incidence	Republic of Indonesia	Female	Age-standardized	Edentulism	Rate	2003	377.2	438.2	326.3
Incidence	Republic of Indonesia	Both	Age-standardized	Edentulism	Rate	2003	319.2	375.0	272.3
Incidence	Republic of Indonesia	Male	Age-standardized	Edentulism	Rate	2004	262.4	316.5	216.8
Incidence	Republic of Indonesia	Female	Age-standardized	Edentulism	Rate	2004	381.0	442.6	333.1
Incidence	Republic of Indonesia	Both	Age-standardized	Edentulism	Rate	2004	322.5	377.7	277.2
Incidence	Republic of	Male	Age-standardized	Edentulism	Rate	2005	263.7	316.9	220.5

	Indonesia								
Incidence	Republic of Indonesia	Female	Age-standardized	Edentulism	Rate	2005	382.0	442.6	334.9
Incidence	Republic of Indonesia	Both	Age-standardized	Edentulism	Rate	2005	323.5	379.4	278.4
Incidence	Republic of Indonesia	Male	Age-standardized	Edentulism	Rate	2006	263.7	315.2	222.8
Incidence	Republic of Indonesia	Female	Age-standardized	Edentulism	Rate	2006	381.3	439.6	333.5
Incidence	Republic of Indonesia	Both	Age-standardized	Edentulism	Rate	2006	323.1	378.2	279.4
Incidence	Republic of Indonesia	Male	Age-standardized	Edentulism	Rate	2007	263.9	312.8	223.0
Incidence	Republic of Indonesia	Female	Age-standardized	Edentulism	Rate	2007	380.9	439.3	331.8
Incidence	Republic of Indonesia	Both	Age-standardized	Edentulism	Rate	2007	323.0	376.6	280.6
Incidence	Republic of Indonesia	Male	Age-standardized	Edentulism	Rate	2008	264.0	311.9	224.9
Incidence	Republic of Indonesia	Female	Age-standardized	Edentulism	Rate	2008	380.5	442.0	330.4
Incidence	Republic of Indonesia	Both	Age-standardized	Edentulism	Rate	2008	322.8	374.5	281.6
Incidence	Republic of Indonesia	Male	Age-standardized	Edentulism	Rate	2009	263.9	311.9	227.0
Incidence	Republic of Indonesia	Female	Age-standardized	Edentulism	Rate	2009	380.0	441.3	330.6
Incidence	Republic of Indonesia	Both	Age-standardized	Edentulism	Rate	2009	322.4	377.4	279.1
Incidence	Republic of Indonesia	Male	Age-standardized	Edentulism	Rate	2010	263.4	314.0	225.8
Incidence	Republic of Indonesia	Female	Age-standardized	Edentulism	Rate	2010	379.0	442.1	328.5
Incidence	Republic of Indonesia	Both	Age-standardized	Edentulism	Rate	2010	321.6	377.5	276.9
Incidence	Republic of Indonesia	Male	Age-standardized	Edentulism	Rate	2011	262.5	313.0	225.1
Incidence	Republic of Indonesia	Female	Age-standardized	Edentulism	Rate	2011	377.4	440.5	326.4
Incidence	Republic of Indonesia	Both	Age-standardized	Edentulism	Rate	2011	320.2	376.1	275.7
Incidence	Republic of Indonesia	Male	Age-standardized	Edentulism	Rate	2012	261.2	311.8	224.1
Incidence	Republic of	Female	Age-standardized	Edentulism	Rate	2012	375.2	438.3	324.0

	Indonesia								
Incidence	Republic of Indonesia	Both	Age-standardized	Edentulism	Rate	2012	318.4	374.2	274.1
Incidence	Republic of Indonesia	Male	Age-standardized	Edentulism	Rate	2013	259.7	310.4	223.1
Incidence	Republic of Indonesia	Female	Age-standardized	Edentulism	Rate	2013	372.8	436.2	321.6
Incidence	Republic of Indonesia	Both	Age-standardized	Edentulism	Rate	2013	316.5	372.1	272.4
Incidence	Republic of Indonesia	Male	Age-standardized	Edentulism	Rate	2014	258.4	309.2	222.3
Incidence	Republic of Indonesia	Female	Age-standardized	Edentulism	Rate	2014	370.7	434.1	319.2
Incidence	Republic of Indonesia	Both	Age-standardized	Edentulism	Rate	2014	314.6	370.2	270.7
Incidence	Republic of Indonesia	Male	Age-standardized	Edentulism	Rate	2015	257.2	308.2	221.4
Incidence	Republic of Indonesia	Female	Age-standardized	Edentulism	Rate	2015	368.9	432.6	317.5
Incidence	Republic of Indonesia	Both	Age-standardized	Edentulism	Rate	2015	313.2	368.7	269.4
Incidence	Republic of Indonesia	Male	Age-standardized	Edentulism	Rate	2016	256.1	305.4	220.6
Incidence	Republic of Indonesia	Female	Age-standardized	Edentulism	Rate	2016	367.4	431.0	316.7
Incidence	Republic of Indonesia	Both	Age-standardized	Edentulism	Rate	2016	311.8	366.5	268.1
Incidence	Republic of Indonesia	Male	Age-standardized	Edentulism	Rate	2017	254.7	302.6	219.6
Incidence	Republic of Indonesia	Female	Age-standardized	Edentulism	Rate	2017	365.8	428.8	316.0
Incidence	Republic of Indonesia	Both	Age-standardized	Edentulism	Rate	2017	310.3	364.2	266.6
Incidence	Republic of Indonesia	Male	Age-standardized	Edentulism	Rate	2018	253.5	300.4	218.4
Incidence	Republic of Indonesia	Female	Age-standardized	Edentulism	Rate	2018	364.2	426.6	315.3
Incidence	Republic of Indonesia	Both	Age-standardized	Edentulism	Rate	2018	308.9	362.2	265.4
Incidence	Republic of Indonesia	Male	Age-standardized	Edentulism	Rate	2019	252.7	299.4	217.7
Incidence	Republic of Indonesia	Female	Age-standardized	Edentulism	Rate	2019	363.2	425.3	315.0
Incidence	Republic of	Both	Age-standardized	Edentulism	Rate	2019	307.9	360.6	264.5

	Indonesia								
Incidence	Republic of Indonesia	Male	Age-standardized	Edentulism	Rate	2020	252.9	301.5	216.9
Incidence	Republic of Indonesia	Female	Age-standardized	Edentulism	Rate	2020	362.4	423.2	314.8
Incidence	Republic of Indonesia	Both	Age-standardized	Edentulism	Rate	2020	307.6	362.6	264.9
Incidence	Republic of Indonesia	Male	Age-standardized	Edentulism	Rate	2021	251.5	300.3	215.7
Incidence	Republic of Indonesia	Female	Age-standardized	Edentulism	Rate	2021	361.4	423.8	312.0
Incidence	Republic of Indonesia	Both	Age-standardized	Edentulism	Rate	2021	306.4	360.7	262.5
Incidence	Republic of Indonesia	Male	Age-standardized	Edentulism	Rate	2022	251.2	297.9	215.3
Incidence	Republic of Indonesia	Female	Age-standardized	Edentulism	Rate	2022	360.7	421.2	310.9
Incidence	Republic of Indonesia	Both	Age-standardized	Edentulism	Rate	2022	306.0	358.5	263.6
Incidence	Republic of Indonesia	Male	Age-standardized	Edentulism	Rate	2023	248.7	295.3	213.2
Incidence	Republic of Indonesia	Female	Age-standardized	Edentulism	Rate	2023	356.7	416.8	307.4
Incidence	Republic of Indonesia	Both	Age-standardized	Edentulism	Rate	2023	302.7	355.0	260.7
Incidence	French Republic	Male	Age-standardized	Edentulism	Rate	1990	260.6	322.0	205.6
Incidence	French Republic	Female	Age-standardized	Edentulism	Rate	1990	333.3	407.7	265.1
Incidence	French Republic	Both	Age-standardized	Edentulism	Rate	1990	297.2	364.4	235.7
Incidence	French Republic	Male	Age-standardized	Edentulism	Rate	1991	244.9	301.9	196.3
Incidence	French Republic	Female	Age-standardized	Edentulism	Rate	1991	313.5	383.4	251.7
Incidence	French Republic	Both	Age-standardized	Edentulism	Rate	1991	279.5	343.1	224.3
Incidence	French Republic	Male	Age-standardized	Edentulism	Rate	1992	232.5	285.9	188.0
Incidence	French Republic	Female	Age-standardized	Edentulism	Rate	1992	297.5	363.7	241.4
Incidence	French Republic	Both	Age-standardized	Edentulism	Rate	1992	265.4	325.0	215.0
Incidence	French Republic	Male	Age-standardized	Edentulism	Rate	1993	223.3	276.4	181.3
Incidence	French Republic	Female	Age-standardized	Edentulism	Rate	1993	285.8	350.1	234.0
Incidence	French Republic	Both	Age-standardized	Edentulism	Rate	1993	254.9	312.8	208.5
Incidence	French Republic	Male	Age-standardized	Edentulism	Rate	1994	217.6	271.3	177.5
Incidence	French Republic	Female	Age-standardized	Edentulism	Rate	1994	278.4	343.8	230.1
Incidence	French Republic	Both	Age-standardized	Edentulism	Rate	1994	248.4	306.4	203.8
Incidence	French Republic	Male	Age-standardized	Edentulism	Rate	1995	215.7	269.8	176.3
Incidence	French Republic	Female	Age-standardized	Edentulism	Rate	1995	275.9	341.0	228.3
Incidence	French Republic	Both	Age-standardized	Edentulism	Rate	1995	246.1	305.5	202.3
Incidence	French Republic	Male	Age-standardized	Edentulism	Rate	1996	219.1	273.8	178.8

Incidence	French Republic	Female	Age-standardized	Edentulism	Rate	1996	280.1	346.2	230.7
Incidence	French Republic	Both	Age-standardized	Edentulism	Rate	1996	249.8	309.5	204.8
Incidence	French Republic	Male	Age-standardized	Edentulism	Rate	1997	227.4	283.4	184.2
Incidence	French Republic	Female	Age-standardized	Edentulism	Rate	1997	290.2	357.6	237.2
Incidence	French Republic	Both	Age-standardized	Edentulism	Rate	1997	259.0	321.0	211.3
Incidence	French Republic	Male	Age-standardized	Edentulism	Rate	1998	237.6	295.8	190.6
Incidence	French Republic	Female	Age-standardized	Edentulism	Rate	1998	302.7	371.5	244.6
Incidence	French Republic	Both	Age-standardized	Edentulism	Rate	1998	270.2	334.9	218.0
Incidence	French Republic	Male	Age-standardized	Edentulism	Rate	1999	246.7	307.6	195.9
Incidence	French Republic	Female	Age-standardized	Edentulism	Rate	1999	313.8	383.5	252.5
Incidence	French Republic	Both	Age-standardized	Edentulism	Rate	1999	280.2	347.7	224.6
Incidence	French Republic	Male	Age-standardized	Edentulism	Rate	2000	251.5	314.0	199.6
Incidence	French Republic	Female	Age-standardized	Edentulism	Rate	2000	320.0	391.2	255.5
Incidence	French Republic	Both	Age-standardized	Edentulism	Rate	2000	285.7	353.7	229.1
Incidence	French Republic	Male	Age-standardized	Edentulism	Rate	2001	252.9	310.1	204.0
Incidence	French Republic	Female	Age-standardized	Edentulism	Rate	2001	322.5	387.5	262.3
Incidence	French Republic	Both	Age-standardized	Edentulism	Rate	2001	287.8	347.2	234.0
Incidence	French Republic	Male	Age-standardized	Edentulism	Rate	2002	253.8	304.1	208.4
Incidence	French Republic	Female	Age-standardized	Edentulism	Rate	2002	324.7	384.7	269.8
Incidence	French Republic	Both	Age-standardized	Edentulism	Rate	2002	289.7	343.8	239.3
Incidence	French Republic	Male	Age-standardized	Edentulism	Rate	2003	253.8	299.2	212.4
Incidence	French Republic	Female	Age-standardized	Edentulism	Rate	2003	326.0	381.2	277.7
Incidence	French Republic	Both	Age-standardized	Edentulism	Rate	2003	290.7	340.3	246.2
Incidence	French Republic	Male	Age-standardized	Edentulism	Rate	2004	252.8	294.4	215.4
Incidence	French Republic	Female	Age-standardized	Edentulism	Rate	2004	325.8	373.9	281.5
Incidence	French Republic	Both	Age-standardized	Edentulism	Rate	2004	290.4	335.8	250.5
Incidence	French Republic	Male	Age-standardized	Edentulism	Rate	2005	250.7	289.8	215.9
Incidence	French Republic	Female	Age-standardized	Edentulism	Rate	2005	323.5	365.5	280.7
Incidence	French Republic	Both	Age-standardized	Edentulism	Rate	2005	288.3	328.8	249.4
Incidence	French Republic	Male	Age-standardized	Edentulism	Rate	2006	240.3	274.1	210.4
Incidence	French Republic	Female	Age-standardized	Edentulism	Rate	2006	309.9	346.8	271.1
Incidence	French Republic	Both	Age-standardized	Edentulism	Rate	2006	276.1	309.6	241.1
Incidence	French Republic	Male	Age-standardized	Edentulism	Rate	2007	219.2	246.9	192.9
Incidence	French Republic	Female	Age-standardized	Edentulism	Rate	2007	282.6	314.4	248.8
Incidence	French Republic	Both	Age-standardized	Edentulism	Rate	2007	251.6	280.7	222.5
Incidence	French Republic	Male	Age-standardized	Edentulism	Rate	2008	194.9	217.2	172.6
Incidence	French Republic	Female	Age-standardized	Edentulism	Rate	2008	251.3	278.6	223.3
Incidence	French Republic	Both	Age-standardized	Edentulism	Rate	2008	223.6	248.0	198.5
Incidence	French Republic	Male	Age-standardized	Edentulism	Rate	2009	174.9	193.8	156.6
Incidence	French Republic	Female	Age-standardized	Edentulism	Rate	2009	225.6	248.8	202.5
Incidence	French Republic	Both	Age-standardized	Edentulism	Rate	2009	200.6	221.3	180.1
Incidence	French Republic	Male	Age-standardized	Edentulism	Rate	2010	166.6	184.9	150.6
Incidence	French Republic	Female	Age-standardized	Edentulism	Rate	2010	215.3	236.6	193.8
Incidence	French Republic	Both	Age-standardized	Edentulism	Rate	2010	191.2	210.8	172.7

Incidence	French Republic	Male	Age-standardized	Edentulism	Rate	2011	174.7	194.9	155.9
Incidence	French Republic	Female	Age-standardized	Edentulism	Rate	2011	225.4	248.4	201.1
Incidence	French Republic	Both	Age-standardized	Edentulism	Rate	2011	200.3	221.2	178.7
Incidence	French Republic	Male	Age-standardized	Edentulism	Rate	2012	194.0	222.3	169.5
Incidence	French Republic	Female	Age-standardized	Edentulism	Rate	2012	248.8	279.3	217.8
Incidence	French Republic	Both	Age-standardized	Edentulism	Rate	2012	221.6	250.9	193.5
Incidence	French Republic	Male	Age-standardized	Edentulism	Rate	2013	216.9	256.3	183.8
Incidence	French Republic	Female	Age-standardized	Edentulism	Rate	2013	276.6	319.4	235.1
Incidence	French Republic	Both	Age-standardized	Edentulism	Rate	2013	246.9	286.7	210.4
Incidence	French Republic	Male	Age-standardized	Edentulism	Rate	2014	236.2	288.0	192.2
Incidence	French Republic	Female	Age-standardized	Edentulism	Rate	2014	299.9	357.9	248.3
Incidence	French Republic	Both	Age-standardized	Edentulism	Rate	2014	268.2	320.1	220.5
Incidence	French Republic	Male	Age-standardized	Edentulism	Rate	2015	244.3	307.7	189.0
Incidence	French Republic	Female	Age-standardized	Edentulism	Rate	2015	309.7	379.5	248.0
Incidence	French Republic	Both	Age-standardized	Edentulism	Rate	2015	277.2	343.5	218.2
Incidence	French Republic	Male	Age-standardized	Edentulism	Rate	2016	243.8	305.3	188.9
Incidence	French Republic	Female	Age-standardized	Edentulism	Rate	2016	309.6	378.8	248.2
Incidence	French Republic	Both	Age-standardized	Edentulism	Rate	2016	276.9	343.0	218.7
Incidence	French Republic	Male	Age-standardized	Edentulism	Rate	2017	242.6	302.2	189.6
Incidence	French Republic	Female	Age-standardized	Edentulism	Rate	2017	308.9	378.5	245.9
Incidence	French Republic	Both	Age-standardized	Edentulism	Rate	2017	276.1	340.7	217.4
Incidence	French Republic	Male	Age-standardized	Edentulism	Rate	2018	241.4	299.6	191.0
Incidence	French Republic	Female	Age-standardized	Edentulism	Rate	2018	308.2	378.0	243.8
Incidence	French Republic	Both	Age-standardized	Edentulism	Rate	2018	275.2	338.2	215.7
Incidence	French Republic	Male	Age-standardized	Edentulism	Rate	2019	240.7	298.6	190.5
Incidence	French Republic	Female	Age-standardized	Edentulism	Rate	2019	307.7	377.7	242.1
Incidence	French Republic	Both	Age-standardized	Edentulism	Rate	2019	274.7	337.4	214.9
Incidence	French Republic	Male	Age-standardized	Edentulism	Rate	2020	241.4	301.0	192.4
Incidence	French Republic	Female	Age-standardized	Edentulism	Rate	2020	308.3	377.8	244.5
Incidence	French Republic	Both	Age-standardized	Edentulism	Rate	2020	275.4	338.3	218.9
Incidence	French Republic	Male	Age-standardized	Edentulism	Rate	2021	242.2	297.5	193.8
Incidence	French Republic	Female	Age-standardized	Edentulism	Rate	2021	308.1	387.8	246.5
Incidence	French Republic	Both	Age-standardized	Edentulism	Rate	2021	275.7	342.7	221.3
Incidence	French Republic	Male	Age-standardized	Edentulism	Rate	2022	242.4	300.8	187.8
Incidence	French Republic	Female	Age-standardized	Edentulism	Rate	2022	309.4	385.9	244.1
Incidence	French Republic	Both	Age-standardized	Edentulism	Rate	2022	276.4	344.3	217.6
Incidence	French Republic	Male	Age-standardized	Edentulism	Rate	2023	241.1	299.2	186.8
Incidence	French Republic	Female	Age-standardized	Edentulism	Rate	2023	307.5	383.6	242.7
Incidence	French Republic	Both	Age-standardized	Edentulism	Rate	2023	274.9	342.4	216.4
Incidence	Federative Republic of Brazil	Male	Age-standardized	Edentulism	Rate	1990	541.6	628.4	461.8
Incidence	Federative Republic of Brazil	Female	Age-standardized	Edentulism	Rate	1990	656.0	738.9	572.9
Incidence	Federative	Both	Age-standardized	Edentulism	Rate	1990	599.5	681.7	521.5

	Republic of Brazil								
Incidence	Federative Republic of Brazil	Male	Age-standardized	Edentulism	Rate	1991	540.7	627.3	461.4
Incidence	Federative Republic of Brazil	Female	Age-standardized	Edentulism	Rate	1991	655.8	739.2	572.6
Incidence	Federative Republic of Brazil	Both	Age-standardized	Edentulism	Rate	1991	599.0	680.3	520.8
Incidence	Federative Republic of Brazil	Male	Age-standardized	Edentulism	Rate	1992	540.1	626.5	461.4
Incidence	Federative Republic of Brazil	Female	Age-standardized	Edentulism	Rate	1992	655.4	738.7	572.0
Incidence	Federative Republic of Brazil	Both	Age-standardized	Edentulism	Rate	1992	598.6	679.4	520.2
Incidence	Federative Republic of Brazil	Male	Age-standardized	Edentulism	Rate	1993	539.7	626.0	461.6
Incidence	Federative Republic of Brazil	Female	Age-standardized	Edentulism	Rate	1993	654.9	737.8	571.4
Incidence	Federative Republic of Brazil	Both	Age-standardized	Edentulism	Rate	1993	598.2	678.5	519.9
Incidence	Federative Republic of Brazil	Male	Age-standardized	Edentulism	Rate	1994	539.4	625.7	462.0
Incidence	Federative Republic of Brazil	Female	Age-standardized	Edentulism	Rate	1994	654.4	736.6	571.2
Incidence	Federative Republic of Brazil	Both	Age-standardized	Edentulism	Rate	1994	597.8	677.7	519.5
Incidence	Federative Republic of Brazil	Male	Age-standardized	Edentulism	Rate	1995	539.2	625.6	462.2
Incidence	Federative Republic of Brazil	Female	Age-standardized	Edentulism	Rate	1995	653.7	735.4	570.9
Incidence	Federative Republic of Brazil	Both	Age-standardized	Edentulism	Rate	1995	597.4	676.9	518.9
Incidence	Federative Republic of Brazil	Male	Age-standardized	Edentulism	Rate	1996	539.5	623.0	462.5
Incidence	Federative Republic of Brazil	Female	Age-standardized	Edentulism	Rate	1996	651.4	732.0	569.5
Incidence	Federative Republic of Brazil	Both	Age-standardized	Edentulism	Rate	1996	596.4	675.4	518.6
Incidence	Federative Republic of Brazil	Male	Age-standardized	Edentulism	Rate	1997	540.9	621.3	465.1
Incidence	Federative Republic of Brazil	Female	Age-standardized	Edentulism	Rate	1997	646.8	726.4	565.8
Incidence	Federative Republic of Brazil	Both	Age-standardized	Edentulism	Rate	1997	594.7	673.3	518.5
Incidence	Federative	Male	Age-standardized	Edentulism	Rate	1998	542.7	620.4	467.5

	Republic of Brazil								
Incidence	Federative Republic of Brazil	Female	Age-standardized	Edentulism	Rate	1998	641.6	720.6	562.5
Incidence	Federative Republic of Brazil	Both	Age-standardized	Edentulism	Rate	1998	592.9	671.2	518.7
Incidence	Federative Republic of Brazil	Male	Age-standardized	Edentulism	Rate	1999	544.4	620.3	471.6
Incidence	Federative Republic of Brazil	Female	Age-standardized	Edentulism	Rate	1999	637.3	716.0	559.3
Incidence	Federative Republic of Brazil	Both	Age-standardized	Edentulism	Rate	1999	591.5	669.7	518.1
Incidence	Federative Republic of Brazil	Male	Age-standardized	Edentulism	Rate	2000	545.5	620.0	474.3
Incidence	Federative Republic of Brazil	Female	Age-standardized	Edentulism	Rate	2000	635.4	714.4	557.4
Incidence	Federative Republic of Brazil	Both	Age-standardized	Edentulism	Rate	2000	591.0	669.1	518.1
Incidence	Federative Republic of Brazil	Male	Age-standardized	Edentulism	Rate	2001	552.3	627.3	480.4
Incidence	Federative Republic of Brazil	Female	Age-standardized	Edentulism	Rate	2001	647.4	719.4	576.7
Incidence	Federative Republic of Brazil	Both	Age-standardized	Edentulism	Rate	2001	600.4	674.2	530.1
Incidence	Federative Republic of Brazil	Male	Age-standardized	Edentulism	Rate	2002	567.6	646.4	496.2
Incidence	Federative Republic of Brazil	Female	Age-standardized	Edentulism	Rate	2002	676.2	742.5	607.0
Incidence	Federative Republic of Brazil	Both	Age-standardized	Edentulism	Rate	2002	622.5	693.7	553.8
Incidence	Federative Republic of Brazil	Male	Age-standardized	Edentulism	Rate	2003	585.7	662.4	513.6
Incidence	Federative Republic of Brazil	Female	Age-standardized	Edentulism	Rate	2003	710.9	776.2	642.2
Incidence	Federative Republic of Brazil	Both	Age-standardized	Edentulism	Rate	2003	648.9	720.6	581.6
Incidence	Federative Republic of Brazil	Male	Age-standardized	Edentulism	Rate	2004	601.0	680.8	527.4
Incidence	Federative Republic of Brazil	Female	Age-standardized	Edentulism	Rate	2004	740.4	803.4	674.8
Incidence	Federative Republic of Brazil	Both	Age-standardized	Edentulism	Rate	2004	671.3	740.2	603.8
Incidence	Federative Republic of Brazil	Male	Age-standardized	Edentulism	Rate	2005	607.7	688.9	534.5
Incidence	Federative	Female	Age-standardized	Edentulism	Rate	2005	753.7	814.2	693.3

	Republic of Brazil								
Incidence	Federative Republic of Brazil	Both	Age-standardized	Edentulism	Rate	2005	681.3	744.2	617.7
Incidence	Federative Republic of Brazil	Male	Age-standardized	Edentulism	Rate	2006	603.0	676.6	537.2
Incidence	Federative Republic of Brazil	Female	Age-standardized	Edentulism	Rate	2006	745.1	801.9	687.2
Incidence	Federative Republic of Brazil	Both	Age-standardized	Edentulism	Rate	2006	674.7	733.9	614.1
Incidence	Federative Republic of Brazil	Male	Age-standardized	Edentulism	Rate	2007	591.0	651.1	530.1
Incidence	Federative Republic of Brazil	Female	Age-standardized	Edentulism	Rate	2007	722.4	775.8	665.7
Incidence	Federative Republic of Brazil	Both	Age-standardized	Edentulism	Rate	2007	657.5	710.5	601.0
Incidence	Federative Republic of Brazil	Male	Age-standardized	Edentulism	Rate	2008	576.4	628.8	519.4
Incidence	Federative Republic of Brazil	Female	Age-standardized	Edentulism	Rate	2008	694.7	746.0	638.3
Incidence	Federative Republic of Brazil	Both	Age-standardized	Edentulism	Rate	2008	636.4	686.4	580.6
Incidence	Federative Republic of Brazil	Male	Age-standardized	Edentulism	Rate	2009	564.0	616.0	511.7
Incidence	Federative Republic of Brazil	Female	Age-standardized	Edentulism	Rate	2009	670.9	723.7	617.7
Incidence	Federative Republic of Brazil	Both	Age-standardized	Edentulism	Rate	2009	618.5	669.5	565.9
Incidence	Federative Republic of Brazil	Male	Age-standardized	Edentulism	Rate	2010	558.5	612.7	507.2
Incidence	Federative Republic of Brazil	Female	Age-standardized	Edentulism	Rate	2010	660.2	718.3	606.1
Incidence	Federative Republic of Brazil	Both	Age-standardized	Edentulism	Rate	2010	610.5	663.8	558.6
Incidence	Federative Republic of Brazil	Male	Age-standardized	Edentulism	Rate	2011	557.9	614.5	505.3
Incidence	Federative Republic of Brazil	Female	Age-standardized	Edentulism	Rate	2011	659.3	717.8	603.1
Incidence	Federative Republic of Brazil	Both	Age-standardized	Edentulism	Rate	2011	609.7	664.2	556.2
Incidence	Federative Republic of Brazil	Male	Age-standardized	Edentulism	Rate	2012	556.9	615.1	502.1
Incidence	Federative Republic of Brazil	Female	Age-standardized	Edentulism	Rate	2012	658.8	717.6	600.6
Incidence	Federative	Both	Age-standardized	Edentulism	Rate	2012	609.0	664.5	552.7

	Republic of Brazil								
Incidence	Federative Republic of Brazil	Male	Age-standardized	Edentulism	Rate	2013	555.7	616.3	498.9
Incidence	Federative Republic of Brazil	Female	Age-standardized	Edentulism	Rate	2013	658.6	716.9	598.7
Incidence	Federative Republic of Brazil	Both	Age-standardized	Edentulism	Rate	2013	608.3	664.7	549.2
Incidence	Federative Republic of Brazil	Male	Age-standardized	Edentulism	Rate	2014	554.4	616.2	497.0
Incidence	Federative Republic of Brazil	Female	Age-standardized	Edentulism	Rate	2014	658.7	717.8	597.0
Incidence	Federative Republic of Brazil	Both	Age-standardized	Edentulism	Rate	2014	607.7	664.8	545.4
Incidence	Federative Republic of Brazil	Male	Age-standardized	Edentulism	Rate	2015	553.2	617.2	492.9
Incidence	Federative Republic of Brazil	Female	Age-standardized	Edentulism	Rate	2015	658.8	719.2	593.6
Incidence	Federative Republic of Brazil	Both	Age-standardized	Edentulism	Rate	2015	607.2	666.1	541.2
Incidence	Federative Republic of Brazil	Male	Age-standardized	Edentulism	Rate	2016	550.2	614.2	489.2
Incidence	Federative Republic of Brazil	Female	Age-standardized	Edentulism	Rate	2016	658.9	719.0	593.5
Incidence	Federative Republic of Brazil	Both	Age-standardized	Edentulism	Rate	2016	605.8	665.2	539.0
Incidence	Federative Republic of Brazil	Male	Age-standardized	Edentulism	Rate	2017	545.2	610.5	483.0
Incidence	Federative Republic of Brazil	Female	Age-standardized	Edentulism	Rate	2017	659.0	720.1	592.3
Incidence	Federative Republic of Brazil	Both	Age-standardized	Edentulism	Rate	2017	603.5	663.4	537.3
Incidence	Federative Republic of Brazil	Male	Age-standardized	Edentulism	Rate	2018	540.2	605.9	477.0
Incidence	Federative Republic of Brazil	Female	Age-standardized	Edentulism	Rate	2018	659.1	722.5	591.5
Incidence	Federative Republic of Brazil	Both	Age-standardized	Edentulism	Rate	2018	601.2	661.8	536.0
Incidence	Federative Republic of Brazil	Male	Age-standardized	Edentulism	Rate	2019	537.6	603.4	473.7
Incidence	Federative Republic of Brazil	Female	Age-standardized	Edentulism	Rate	2019	659.3	724.1	591.0
Incidence	Federative Republic of Brazil	Both	Age-standardized	Edentulism	Rate	2019	600.1	661.3	534.9
Incidence	Federative	Male	Age-standardized	Edentulism	Rate	2020	538.1	604.1	476.2

	Republic of Brazil								
Incidence	Federative Republic of Brazil	Female	Age-standardized	Edentulism	Rate	2020	659.5	721.8	592.7
Incidence	Federative Republic of Brazil	Both	Age-standardized	Edentulism	Rate	2020	600.4	662.2	536.1
Incidence	Federative Republic of Brazil	Male	Age-standardized	Edentulism	Rate	2021	537.8	602.0	477.5
Incidence	Federative Republic of Brazil	Female	Age-standardized	Edentulism	Rate	2021	659.3	720.8	591.2
Incidence	Federative Republic of Brazil	Both	Age-standardized	Edentulism	Rate	2021	600.2	661.0	535.3
Incidence	Federative Republic of Brazil	Male	Age-standardized	Edentulism	Rate	2022	538.0	601.9	477.7
Incidence	Federative Republic of Brazil	Female	Age-standardized	Edentulism	Rate	2022	659.6	719.9	592.9
Incidence	Federative Republic of Brazil	Both	Age-standardized	Edentulism	Rate	2022	600.5	660.5	537.8
Incidence	Federative Republic of Brazil	Male	Age-standardized	Edentulism	Rate	2023	533.6	597.5	473.7
Incidence	Federative Republic of Brazil	Female	Age-standardized	Edentulism	Rate	2023	653.5	713.3	587.4
Incidence	Federative Republic of Brazil	Both	Age-standardized	Edentulism	Rate	2023	595.2	654.7	532.9
Incidence	United States of America	Male	Age-standardized	Edentulism	Rate	1990	386.2	478.1	308.3
Incidence	United States of America	Female	Age-standardized	Edentulism	Rate	1990	388.9	481.1	308.1
Incidence	United States of America	Both	Age-standardized	Edentulism	Rate	1990	386.1	478.0	307.5
Incidence	United States of America	Male	Age-standardized	Edentulism	Rate	1991	371.0	456.1	297.8
Incidence	United States of America	Female	Age-standardized	Edentulism	Rate	1991	381.2	468.8	303.1
Incidence	United States of America	Both	Age-standardized	Edentulism	Rate	1991	375.3	462.0	299.5
Incidence	United States of America	Male	Age-standardized	Edentulism	Rate	1992	357.7	437.2	287.7
Incidence	United States of America	Female	Age-standardized	Edentulism	Rate	1992	374.0	457.4	298.2
Incidence	United States of America	Both	Age-standardized	Edentulism	Rate	1992	365.6	447.1	292.2
Incidence	United States of America	Male	Age-standardized	Edentulism	Rate	1993	347.0	423.6	278.7
Incidence	United States of	Female	Age-standardized	Edentulism	Rate	1993	367.8	447.3	294.6

	America								
Incidence	United States of America	Both	Age-standardized	Edentulism	Rate	1993	357.4	435.4	286.8
Incidence	United States of America	Male	Age-standardized	Edentulism	Rate	1994	339.5	415.1	273.9
Incidence	United States of America	Female	Age-standardized	Edentulism	Rate	1994	363.0	440.0	292.4
Incidence	United States of America	Both	Age-standardized	Edentulism	Rate	1994	351.4	427.6	283.0
Incidence	United States of America	Male	Age-standardized	Edentulism	Rate	1995	335.7	410.1	272.3
Incidence	United States of America	Female	Age-standardized	Edentulism	Rate	1995	359.7	435.8	291.7
Incidence	United States of America	Both	Age-standardized	Edentulism	Rate	1995	348.0	421.8	281.9
Incidence	United States of America	Male	Age-standardized	Edentulism	Rate	1996	335.6	410.4	271.6
Incidence	United States of America	Female	Age-standardized	Edentulism	Rate	1996	357.6	433.1	289.1
Incidence	United States of America	Both	Age-standardized	Edentulism	Rate	1996	346.6	420.1	280.2
Incidence	United States of America	Male	Age-standardized	Edentulism	Rate	1997	337.3	411.8	273.1
Incidence	United States of America	Female	Age-standardized	Edentulism	Rate	1997	355.5	431.4	287.4
Incidence	United States of America	Both	Age-standardized	Edentulism	Rate	1997	346.1	420.8	280.0
Incidence	United States of America	Male	Age-standardized	Edentulism	Rate	1998	339.7	415.4	276.3
Incidence	United States of America	Female	Age-standardized	Edentulism	Rate	1998	353.4	429.8	286.4
Incidence	United States of America	Both	Age-standardized	Edentulism	Rate	1998	345.9	421.8	280.5
Incidence	United States of America	Male	Age-standardized	Edentulism	Rate	1999	341.2	419.7	278.2
Incidence	United States of America	Female	Age-standardized	Edentulism	Rate	1999	351.3	428.6	285.3
Incidence	United States of America	Both	Age-standardized	Edentulism	Rate	1999	345.3	422.9	280.3
Incidence	United States of America	Male	Age-standardized	Edentulism	Rate	2000	340.7	421.3	278.4
Incidence	United States of America	Female	Age-standardized	Edentulism	Rate	2000	349.0	427.1	283.9
Incidence	United States of	Both	Age-standardized	Edentulism	Rate	2000	343.7	422.9	279.4

	America								
Incidence	United States of America	Male	Age-standardized	Edentulism	Rate	2001	332.4	407.6	272.4
Incidence	United States of America	Female	Age-standardized	Edentulism	Rate	2001	342.7	416.6	281.2
Incidence	United States of America	Both	Age-standardized	Edentulism	Rate	2001	336.6	411.2	275.8
Incidence	United States of America	Male	Age-standardized	Edentulism	Rate	2002	315.3	381.6	259.2
Incidence	United States of America	Female	Age-standardized	Edentulism	Rate	2002	331.1	398.6	273.6
Incidence	United States of America	Both	Age-standardized	Edentulism	Rate	2002	322.6	389.3	265.8
Incidence	United States of America	Male	Age-standardized	Edentulism	Rate	2003	295.1	355.3	243.9
Incidence	United States of America	Female	Age-standardized	Edentulism	Rate	2003	317.4	379.8	264.6
Incidence	United States of America	Both	Age-standardized	Edentulism	Rate	2003	306.0	367.2	253.8
Incidence	United States of America	Male	Age-standardized	Edentulism	Rate	2004	277.2	331.8	230.3
Incidence	United States of America	Female	Age-standardized	Edentulism	Rate	2004	305.2	363.9	258.1
Incidence	United States of America	Both	Age-standardized	Edentulism	Rate	2004	291.2	347.7	244.5
Incidence	United States of America	Male	Age-standardized	Edentulism	Rate	2005	267.3	317.6	224.6
Incidence	United States of America	Female	Age-standardized	Edentulism	Rate	2005	297.6	352.3	252.2
Incidence	United States of America	Both	Age-standardized	Edentulism	Rate	2005	282.6	334.6	238.5
Incidence	United States of America	Male	Age-standardized	Edentulism	Rate	2006	261.5	309.2	221.3
Incidence	United States of America	Female	Age-standardized	Edentulism	Rate	2006	291.0	344.2	247.7
Incidence	United States of America	Both	Age-standardized	Edentulism	Rate	2006	276.4	326.3	234.6
Incidence	United States of America	Male	Age-standardized	Edentulism	Rate	2007	253.6	298.6	215.8
Incidence	United States of America	Female	Age-standardized	Edentulism	Rate	2007	281.1	332.4	240.5
Incidence	United States of America	Both	Age-standardized	Edentulism	Rate	2007	267.6	315.5	228.3
Incidence	United States of	Male	Age-standardized	Edentulism	Rate	2008	245.3	288.1	209.4

	America								
Incidence	United States of America	Female	Age-standardized	Edentulism	Rate	2008	270.3	319.2	232.5
Incidence	United States of America	Both	Age-standardized	Edentulism	Rate	2008	258.0	304.7	220.6
Incidence	United States of America	Male	Age-standardized	Edentulism	Rate	2009	238.2	280.5	203.3
Incidence	United States of America	Female	Age-standardized	Edentulism	Rate	2009	261.1	307.7	224.5
Incidence	United States of America	Both	Age-standardized	Edentulism	Rate	2009	249.8	295.2	214.3
Incidence	United States of America	Male	Age-standardized	Edentulism	Rate	2010	234.2	276.0	200.6
Incidence	United States of America	Female	Age-standardized	Edentulism	Rate	2010	255.7	299.9	219.9
Incidence	United States of America	Both	Age-standardized	Edentulism	Rate	2010	245.2	289.0	210.5
Incidence	United States of America	Male	Age-standardized	Edentulism	Rate	2011	237.4	280.8	203.1
Incidence	United States of America	Female	Age-standardized	Edentulism	Rate	2011	257.9	304.1	221.5
Incidence	United States of America	Both	Age-standardized	Edentulism	Rate	2011	247.8	293.6	212.3
Incidence	United States of America	Male	Age-standardized	Edentulism	Rate	2012	247.9	294.2	211.5
Incidence	United States of America	Female	Age-standardized	Edentulism	Rate	2012	267.0	317.2	229.0
Incidence	United States of America	Both	Age-standardized	Edentulism	Rate	2012	257.4	305.7	220.2
Incidence	United States of America	Male	Age-standardized	Edentulism	Rate	2013	261.4	313.0	222.6
Incidence	United States of America	Female	Age-standardized	Edentulism	Rate	2013	279.0	332.7	237.8
Incidence	United States of America	Both	Age-standardized	Edentulism	Rate	2013	270.0	322.6	229.9
Incidence	United States of America	Male	Age-standardized	Edentulism	Rate	2014	273.7	330.2	232.8
Incidence	United States of America	Female	Age-standardized	Edentulism	Rate	2014	289.9	347.0	246.3
Incidence	United States of America	Both	Age-standardized	Edentulism	Rate	2014	281.5	338.0	239.2
Incidence	United States of America	Male	Age-standardized	Edentulism	Rate	2015	280.6	339.5	237.4
Incidence	United States of	Female	Age-standardized	Edentulism	Rate	2015	295.8	354.8	249.8

	America								
Incidence	United States of America	Both	Age-standardized	Edentulism	Rate	2015	287.8	346.5	243.3
Incidence	United States of America	Male	Age-standardized	Edentulism	Rate	2016	288.8	349.2	244.2
Incidence	United States of America	Female	Age-standardized	Edentulism	Rate	2016	305.6	367.2	256.9
Incidence	United States of America	Both	Age-standardized	Edentulism	Rate	2016	296.8	357.6	250.2
Incidence	United States of America	Male	Age-standardized	Edentulism	Rate	2017	303.6	366.7	254.3
Incidence	United States of America	Female	Age-standardized	Edentulism	Rate	2017	324.6	390.0	270.5
Incidence	United States of America	Both	Age-standardized	Edentulism	Rate	2017	313.8	377.7	261.9
Incidence	United States of America	Male	Age-standardized	Edentulism	Rate	2018	318.3	385.0	263.7
Incidence	United States of America	Female	Age-standardized	Edentulism	Rate	2018	343.5	413.9	284.3
Incidence	United States of America	Both	Age-standardized	Edentulism	Rate	2018	330.7	399.1	273.3
Incidence	United States of America	Male	Age-standardized	Edentulism	Rate	2019	326.1	395.1	268.3
Incidence	United States of America	Female	Age-standardized	Edentulism	Rate	2019	352.9	425.8	291.4
Incidence	United States of America	Both	Age-standardized	Edentulism	Rate	2019	339.4	410.3	279.7
Incidence	United States of America	Male	Age-standardized	Edentulism	Rate	2020	327.8	395.7	270.0
Incidence	United States of America	Female	Age-standardized	Edentulism	Rate	2020	353.6	424.9	292.3
Incidence	United States of America	Both	Age-standardized	Edentulism	Rate	2020	340.5	409.8	281.3
Incidence	United States of America	Male	Age-standardized	Edentulism	Rate	2021	326.7	394.4	268.5
Incidence	United States of America	Female	Age-standardized	Edentulism	Rate	2021	354.0	426.6	291.6
Incidence	United States of America	Both	Age-standardized	Edentulism	Rate	2021	340.1	409.7	280.0
Incidence	United States of America	Male	Age-standardized	Edentulism	Rate	2022	326.9	396.2	269.1
Incidence	United States of America	Female	Age-standardized	Edentulism	Rate	2022	353.2	424.9	292.3
Incidence	United States of	Both	Age-standardized	Edentulism	Rate	2022	339.8	410.2	280.9

	America								
Incidence	United States of America	Male	Age-standardized	Edentulism	Rate	2023	325.4	394.3	267.9
Incidence	United States of America	Female	Age-standardized	Edentulism	Rate	2023	351.3	422.6	290.7
Incidence	United States of America	Both	Age-standardized	Edentulism	Rate	2023	338.1	408.1	279.4
Incidence	United Kingdom of Great Britain and Northern Ireland	Male	Age-standardized	Edentulism	Rate	1990	305.1	375.0	244.3
Incidence	United Kingdom of Great Britain and Northern Ireland	Female	Age-standardized	Edentulism	Rate	1990	356.7	431.7	289.0
Incidence	United Kingdom of Great Britain and Northern Ireland	Both	Age-standardized	Edentulism	Rate	1990	329.9	401.0	267.5
Incidence	United Kingdom of Great Britain and Northern Ireland	Male	Age-standardized	Edentulism	Rate	1991	304.3	374.0	243.8
Incidence	United Kingdom of Great Britain and Northern Ireland	Female	Age-standardized	Edentulism	Rate	1991	355.5	430.2	288.0
Incidence	United Kingdom of Great Britain and Northern Ireland	Both	Age-standardized	Edentulism	Rate	1991	328.8	399.7	266.6
Incidence	United Kingdom of Great Britain and Northern Ireland	Male	Age-standardized	Edentulism	Rate	1992	303.5	373.2	243.3
Incidence	United Kingdom of Great Britain and Northern Ireland	Female	Age-standardized	Edentulism	Rate	1992	354.7	429.2	287.3
Incidence	United Kingdom of Great Britain and Northern Ireland	Both	Age-standardized	Edentulism	Rate	1992	328.1	398.8	265.9
Incidence	United Kingdom of Great Britain and Northern Ireland	Male	Age-standardized	Edentulism	Rate	1993	302.9	372.5	243.0
Incidence	United Kingdom of Great Britain and Northern Ireland	Female	Age-standardized	Edentulism	Rate	1993	354.2	428.5	286.9
Incidence	United Kingdom of Great Britain and Northern Ireland	Both	Age-standardized	Edentulism	Rate	1993	327.5	398.2	265.3
Incidence	United Kingdom of	Male	Age-standardized	Edentulism	Rate	1994	302.4	371.8	242.6

	Great Britain and Northern Ireland								
Incidence	United Kingdom of Great Britain and Northern Ireland	Female	Age-standardized	Edentulism	Rate	1994	353.8	427.9	286.6
Incidence	United Kingdom of Great Britain and Northern Ireland	Both	Age-standardized	Edentulism	Rate	1994	327.1	397.9	264.9
Incidence	United Kingdom of Great Britain and Northern Ireland	Male	Age-standardized	Edentulism	Rate	1995	301.9	371.2	242.3
Incidence	United Kingdom of Great Britain and Northern Ireland	Female	Age-standardized	Edentulism	Rate	1995	353.6	427.1	286.4
Incidence	United Kingdom of Great Britain and Northern Ireland	Both	Age-standardized	Edentulism	Rate	1995	326.8	397.6	264.6
Incidence	United Kingdom of Great Britain and Northern Ireland	Male	Age-standardized	Edentulism	Rate	1996	309.6	378.1	250.1
Incidence	United Kingdom of Great Britain and Northern Ireland	Female	Age-standardized	Edentulism	Rate	1996	361.2	434.3	294.7
Incidence	United Kingdom of Great Britain and Northern Ireland	Both	Age-standardized	Edentulism	Rate	1996	334.5	404.3	272.1
Incidence	United Kingdom of Great Britain and Northern Ireland	Male	Age-standardized	Edentulism	Rate	1997	328.7	398.3	266.3
Incidence	United Kingdom of Great Britain and Northern Ireland	Female	Age-standardized	Edentulism	Rate	1997	379.5	454.4	311.3
Incidence	United Kingdom of Great Britain and Northern Ireland	Both	Age-standardized	Edentulism	Rate	1997	353.3	424.7	288.4
Incidence	United Kingdom of Great Britain and Northern Ireland	Male	Age-standardized	Edentulism	Rate	1998	351.7	423.5	286.5
Incidence	United Kingdom of Great Britain and Northern Ireland	Female	Age-standardized	Edentulism	Rate	1998	401.4	478.5	331.1
Incidence	United Kingdom of Great Britain and Northern Ireland	Both	Age-standardized	Edentulism	Rate	1998	375.8	449.9	308.1

Incidence	United Kingdom of Great Britain and Northern Ireland	Male	Age-standardized	Edentulism	Rate	1999	371.0	445.8	302.7
Incidence	United Kingdom of Great Britain and Northern Ireland	Female	Age-standardized	Edentulism	Rate	1999	419.8	499.0	347.4
Incidence	United Kingdom of Great Britain and Northern Ireland	Both	Age-standardized	Edentulism	Rate	1999	394.7	471.3	325.1
Incidence	United Kingdom of Great Britain and Northern Ireland	Male	Age-standardized	Edentulism	Rate	2000	379.2	453.9	310.8
Incidence	United Kingdom of Great Britain and Northern Ireland	Female	Age-standardized	Edentulism	Rate	2000	427.5	508.0	355.0
Incidence	United Kingdom of Great Britain and Northern Ireland	Both	Age-standardized	Edentulism	Rate	2000	402.6	480.2	332.9
Incidence	United Kingdom of Great Britain and Northern Ireland	Male	Age-standardized	Edentulism	Rate	2001	372.4	447.6	303.7
Incidence	United Kingdom of Great Britain and Northern Ireland	Female	Age-standardized	Edentulism	Rate	2001	421.4	501.3	348.6
Incidence	United Kingdom of Great Britain and Northern Ireland	Both	Age-standardized	Edentulism	Rate	2001	396.2	473.2	326.2
Incidence	United Kingdom of Great Britain and Northern Ireland	Male	Age-standardized	Edentulism	Rate	2002	355.5	429.4	288.7
Incidence	United Kingdom of Great Britain and Northern Ireland	Female	Age-standardized	Edentulism	Rate	2002	405.9	484.7	334.2
Incidence	United Kingdom of Great Britain and Northern Ireland	Both	Age-standardized	Edentulism	Rate	2002	379.9	455.7	311.3
Incidence	United Kingdom of Great Britain and Northern Ireland	Male	Age-standardized	Edentulism	Rate	2003	333.4	405.3	269.7
Incidence	United Kingdom of Great Britain and Northern Ireland	Female	Age-standardized	Edentulism	Rate	2003	385.3	462.8	315.7
Incidence	United Kingdom of Great Britain and	Both	Age-standardized	Edentulism	Rate	2003	358.6	432.6	291.9

	Northern Ireland								
Incidence	United Kingdom of Great Britain and Northern Ireland	Male	Age-standardized	Edentulism	Rate	2004	311.1	381.1	250.6
Incidence	United Kingdom of Great Britain and Northern Ireland	Female	Age-standardized	Edentulism	Rate	2004	363.9	439.9	295.7
Incidence	United Kingdom of Great Britain and Northern Ireland	Both	Age-standardized	Edentulism	Rate	2004	336.8	409.3	273.2
Incidence	United Kingdom of Great Britain and Northern Ireland	Male	Age-standardized	Edentulism	Rate	2005	293.7	362.0	234.2
Incidence	United Kingdom of Great Britain and Northern Ireland	Female	Age-standardized	Edentulism	Rate	2005	346.0	420.5	279.3
Incidence	United Kingdom of Great Britain and Northern Ireland	Both	Age-standardized	Edentulism	Rate	2005	319.1	389.6	258.4
Incidence	United Kingdom of Great Britain and Northern Ireland	Male	Age-standardized	Edentulism	Rate	2006	278.7	341.9	223.5
Incidence	United Kingdom of Great Britain and Northern Ireland	Female	Age-standardized	Edentulism	Rate	2006	329.4	397.6	269.0
Incidence	United Kingdom of Great Britain and Northern Ireland	Both	Age-standardized	Edentulism	Rate	2006	303.5	368.6	246.5
Incidence	United Kingdom of Great Britain and Northern Ireland	Male	Age-standardized	Edentulism	Rate	2007	262.4	320.5	212.5
Incidence	United Kingdom of Great Britain and Northern Ireland	Female	Age-standardized	Edentulism	Rate	2007	311.1	374.0	255.9
Incidence	United Kingdom of Great Britain and Northern Ireland	Both	Age-standardized	Edentulism	Rate	2007	286.3	346.5	233.1
Incidence	United Kingdom of Great Britain and Northern Ireland	Male	Age-standardized	Edentulism	Rate	2008	247.5	301.0	202.3
Incidence	United Kingdom of Great Britain and Northern Ireland	Female	Age-standardized	Edentulism	Rate	2008	294.3	352.7	243.1
Incidence	United Kingdom of	Both	Age-standardized	Edentulism	Rate	2008	270.6	326.3	221.9

	Great Britain and Northern Ireland								
Incidence	United Kingdom of Great Britain and Northern Ireland	Male	Age-standardized	Edentulism	Rate	2009	236.7	286.2	194.4
Incidence	United Kingdom of Great Britain and Northern Ireland	Female	Age-standardized	Edentulism	Rate	2009	282.1	337.3	234.7
Incidence	United Kingdom of Great Britain and Northern Ireland	Both	Age-standardized	Edentulism	Rate	2009	259.2	311.8	214.1
Incidence	United Kingdom of Great Britain and Northern Ireland	Male	Age-standardized	Edentulism	Rate	2010	232.6	280.3	191.6
Incidence	United Kingdom of Great Britain and Northern Ireland	Female	Age-standardized	Edentulism	Rate	2010	277.5	330.3	231.8
Incidence	United Kingdom of Great Britain and Northern Ireland	Both	Age-standardized	Edentulism	Rate	2010	254.9	306.2	211.4
Incidence	United Kingdom of Great Britain and Northern Ireland	Male	Age-standardized	Edentulism	Rate	2011	239.8	288.9	198.0
Incidence	United Kingdom of Great Britain and Northern Ireland	Female	Age-standardized	Edentulism	Rate	2011	285.5	339.5	239.9
Incidence	United Kingdom of Great Britain and Northern Ireland	Both	Age-standardized	Edentulism	Rate	2011	262.5	314.8	218.9
Incidence	United Kingdom of Great Britain and Northern Ireland	Male	Age-standardized	Edentulism	Rate	2012	256.6	308.6	213.0
Incidence	United Kingdom of Great Britain and Northern Ireland	Female	Age-standardized	Edentulism	Rate	2012	304.2	359.5	256.9
Incidence	United Kingdom of Great Britain and Northern Ireland	Both	Age-standardized	Edentulism	Rate	2012	280.2	334.1	235.1
Incidence	United Kingdom of Great Britain and Northern Ireland	Male	Age-standardized	Edentulism	Rate	2013	276.8	331.4	230.2
Incidence	United Kingdom of Great Britain and Northern Ireland	Female	Age-standardized	Edentulism	Rate	2013	326.4	383.7	277.7

Incidence	United Kingdom of Great Britain and Northern Ireland	Both	Age-standardized	Edentulism	Rate	2013	301.3	357.9	253.7
Incidence	United Kingdom of Great Britain and Northern Ireland	Male	Age-standardized	Edentulism	Rate	2014	294.0	351.4	245.8
Incidence	United Kingdom of Great Britain and Northern Ireland	Female	Age-standardized	Edentulism	Rate	2014	345.2	404.3	295.6
Incidence	United Kingdom of Great Britain and Northern Ireland	Both	Age-standardized	Edentulism	Rate	2014	319.3	377.4	270.8
Incidence	United Kingdom of Great Britain and Northern Ireland	Male	Age-standardized	Edentulism	Rate	2015	301.9	359.1	254.3
Incidence	United Kingdom of Great Britain and Northern Ireland	Female	Age-standardized	Edentulism	Rate	2015	353.6	413.1	303.2
Incidence	United Kingdom of Great Britain and Northern Ireland	Both	Age-standardized	Edentulism	Rate	2015	327.4	385.7	279.4
Incidence	United Kingdom of Great Britain and Northern Ireland	Male	Age-standardized	Edentulism	Rate	2016	301.9	359.1	254.8
Incidence	United Kingdom of Great Britain and Northern Ireland	Female	Age-standardized	Edentulism	Rate	2016	353.5	413.4	303.4
Incidence	United Kingdom of Great Britain and Northern Ireland	Both	Age-standardized	Edentulism	Rate	2016	327.4	385.5	279.7
Incidence	United Kingdom of Great Britain and Northern Ireland	Male	Age-standardized	Edentulism	Rate	2017	300.4	357.9	253.8
Incidence	United Kingdom of Great Britain and Northern Ireland	Female	Age-standardized	Edentulism	Rate	2017	352.1	413.3	301.8
Incidence	United Kingdom of Great Britain and Northern Ireland	Both	Age-standardized	Edentulism	Rate	2017	326.0	384.4	278.3
Incidence	United Kingdom of Great Britain and Northern Ireland	Male	Age-standardized	Edentulism	Rate	2018	298.7	357.0	251.9
Incidence	United Kingdom of Great Britain and	Female	Age-standardized	Edentulism	Rate	2018	350.6	413.9	299.7

	Northern Ireland								
Incidence	United Kingdom of Great Britain and Northern Ireland	Both	Age-standardized	Edentulism	Rate	2018	324.4	383.9	276.4
Incidence	United Kingdom of Great Britain and Northern Ireland	Male	Age-standardized	Edentulism	Rate	2019	297.9	357.8	251.0
Incidence	United Kingdom of Great Britain and Northern Ireland	Female	Age-standardized	Edentulism	Rate	2019	349.9	415.5	298.5
Incidence	United Kingdom of Great Britain and Northern Ireland	Both	Age-standardized	Edentulism	Rate	2019	323.8	384.9	275.2
Incidence	United Kingdom of Great Britain and Northern Ireland	Male	Age-standardized	Edentulism	Rate	2020	298.4	357.1	252.0
Incidence	United Kingdom of Great Britain and Northern Ireland	Female	Age-standardized	Edentulism	Rate	2020	351.1	414.4	299.0
Incidence	United Kingdom of Great Britain and Northern Ireland	Both	Age-standardized	Edentulism	Rate	2020	324.7	385.6	277.1
Incidence	United Kingdom of Great Britain and Northern Ireland	Male	Age-standardized	Edentulism	Rate	2021	299.0	357.2	254.5
Incidence	United Kingdom of Great Britain and Northern Ireland	Female	Age-standardized	Edentulism	Rate	2021	355.0	416.9	304.6
Incidence	United Kingdom of Great Britain and Northern Ireland	Both	Age-standardized	Edentulism	Rate	2021	327.0	387.1	278.8
Incidence	United Kingdom of Great Britain and Northern Ireland	Male	Age-standardized	Edentulism	Rate	2022	302.9	360.2	258.2
Incidence	United Kingdom of Great Britain and Northern Ireland	Female	Age-standardized	Edentulism	Rate	2022	358.0	417.8	307.0
Incidence	United Kingdom of Great Britain and Northern Ireland	Both	Age-standardized	Edentulism	Rate	2022	330.4	389.7	283.2
Incidence	United Kingdom of Great Britain and Northern Ireland	Male	Age-standardized	Edentulism	Rate	2023	301.5	358.6	256.9
Incidence	United Kingdom of	Female	Age-standardized	Edentulism	Rate	2023	356.2	415.7	305.4

	Great Britain and Northern Ireland								
Incidence	United Kingdom of Great Britain and Northern Ireland	Both	Age-standardized	Edentulism	Rate	2023	328.9	387.8	281.9
Incidence	United Mexican States	Male	Age-standardized	Edentulism	Rate	1990	338.5	410.5	277.0
Incidence	United Mexican States	Female	Age-standardized	Edentulism	Rate	1990	518.5	609.5	434.5
Incidence	United Mexican States	Both	Age-standardized	Edentulism	Rate	1990	431.4	512.4	358.0
Incidence	United Mexican States	Male	Age-standardized	Edentulism	Rate	1991	338.2	409.7	277.0
Incidence	United Mexican States	Female	Age-standardized	Edentulism	Rate	1991	519.0	609.9	435.0
Incidence	United Mexican States	Both	Age-standardized	Edentulism	Rate	1991	431.5	513.3	358.4
Incidence	United Mexican States	Male	Age-standardized	Edentulism	Rate	1992	338.0	410.0	276.9
Incidence	United Mexican States	Female	Age-standardized	Edentulism	Rate	1992	519.1	610.8	435.7
Incidence	United Mexican States	Both	Age-standardized	Edentulism	Rate	1992	431.5	513.7	358.6
Incidence	United Mexican States	Male	Age-standardized	Edentulism	Rate	1993	337.8	410.3	276.7
Incidence	United Mexican States	Female	Age-standardized	Edentulism	Rate	1993	518.8	611.3	436.1
Incidence	United Mexican States	Both	Age-standardized	Edentulism	Rate	1993	431.2	513.9	358.6
Incidence	United Mexican States	Male	Age-standardized	Edentulism	Rate	1994	337.6	410.2	276.4
Incidence	United Mexican States	Female	Age-standardized	Edentulism	Rate	1994	518.3	611.6	436.2
Incidence	United Mexican States	Both	Age-standardized	Edentulism	Rate	1994	430.8	514.0	358.6
Incidence	United Mexican States	Male	Age-standardized	Edentulism	Rate	1995	337.4	410.3	276.1
Incidence	United Mexican States	Female	Age-standardized	Edentulism	Rate	1995	517.7	611.6	435.7
Incidence	United Mexican States	Both	Age-standardized	Edentulism	Rate	1995	430.4	514.1	358.5
Incidence	United Mexican States	Male	Age-standardized	Edentulism	Rate	1996	337.8	409.8	277.7
Incidence	United Mexican	Female	Age-standardized	Edentulism	Rate	1996	513.0	605.7	431.8

	States									
Incidence	United States	Mexican	Both	Age-standardized	Edentulism	Rate	1996	428.3	510.8	357.2
Incidence	United States	Mexican	Male	Age-standardized	Edentulism	Rate	1997	339.0	410.7	280.3
Incidence	United States	Mexican	Female	Age-standardized	Edentulism	Rate	1997	503.0	593.4	424.1
Incidence	United States	Mexican	Both	Age-standardized	Edentulism	Rate	1997	423.8	504.6	354.4
Incidence	United States	Mexican	Male	Age-standardized	Edentulism	Rate	1998	340.5	411.4	282.9
Incidence	United States	Mexican	Female	Age-standardized	Edentulism	Rate	1998	491.1	578.5	414.1
Incidence	United States	Mexican	Both	Age-standardized	Edentulism	Rate	1998	418.6	497.2	350.9
Incidence	United States	Mexican	Male	Age-standardized	Edentulism	Rate	1999	341.8	412.1	284.5
Incidence	United States	Mexican	Female	Age-standardized	Edentulism	Rate	1999	481.1	565.5	405.8
Incidence	United States	Mexican	Both	Age-standardized	Edentulism	Rate	1999	414.1	490.9	347.7
Incidence	United States	Mexican	Male	Age-standardized	Edentulism	Rate	2000	342.4	412.3	286.1
Incidence	United States	Mexican	Female	Age-standardized	Edentulism	Rate	2000	476.6	559.2	402.3
Incidence	United States	Mexican	Both	Age-standardized	Edentulism	Rate	2000	412.2	488.2	346.5
Incidence	United States	Mexican	Male	Age-standardized	Edentulism	Rate	2001	342.7	410.7	288.9
Incidence	United States	Mexican	Female	Age-standardized	Edentulism	Rate	2001	477.4	551.5	410.6
Incidence	United States	Mexican	Both	Age-standardized	Edentulism	Rate	2001	412.8	483.1	351.1
Incidence	United States	Mexican	Male	Age-standardized	Edentulism	Rate	2002	343.1	409.2	290.2
Incidence	United States	Mexican	Female	Age-standardized	Edentulism	Rate	2002	480.3	550.4	424.2
Incidence	United States	Mexican	Both	Age-standardized	Edentulism	Rate	2002	414.6	479.9	359.1
Incidence	United States	Mexican	Male	Age-standardized	Edentulism	Rate	2003	343.3	407.6	291.4
Incidence	United States	Mexican	Female	Age-standardized	Edentulism	Rate	2003	483.6	549.6	429.3
Incidence	United	Mexican	Both	Age-standardized	Edentulism	Rate	2003	416.5	480.3	365.8

	States									
Incidence	United States	Mexican	Male	Age-standardized	Edentulism	Rate	2004	343.2	406.2	294.9
Incidence	United States	Mexican	Female	Age-standardized	Edentulism	Rate	2004	485.8	545.9	429.4
Incidence	United States	Mexican	Both	Age-standardized	Edentulism	Rate	2004	417.6	478.4	366.6
Incidence	United States	Mexican	Male	Age-standardized	Edentulism	Rate	2005	342.3	404.3	295.6
Incidence	United States	Mexican	Female	Age-standardized	Edentulism	Rate	2005	485.7	551.6	426.7
Incidence	United States	Mexican	Both	Age-standardized	Edentulism	Rate	2005	417.1	476.0	366.1
Incidence	United States	Mexican	Male	Age-standardized	Edentulism	Rate	2006	333.6	389.0	289.3
Incidence	United States	Mexican	Female	Age-standardized	Edentulism	Rate	2006	478.2	546.0	419.0
Incidence	United States	Mexican	Both	Age-standardized	Edentulism	Rate	2006	409.0	467.9	359.2
Incidence	United States	Mexican	Male	Age-standardized	Edentulism	Rate	2007	314.5	367.1	273.3
Incidence	United States	Mexican	Female	Age-standardized	Edentulism	Rate	2007	463.1	530.9	401.4
Incidence	United States	Mexican	Both	Age-standardized	Edentulism	Rate	2007	392.2	451.6	342.4
Incidence	United States	Mexican	Male	Age-standardized	Edentulism	Rate	2008	292.2	340.2	254.2
Incidence	United States	Mexican	Female	Age-standardized	Edentulism	Rate	2008	445.9	515.7	382.0
Incidence	United States	Mexican	Both	Age-standardized	Edentulism	Rate	2008	372.7	431.9	322.9
Incidence	United States	Mexican	Male	Age-standardized	Edentulism	Rate	2009	273.7	323.7	235.5
Incidence	United States	Mexican	Female	Age-standardized	Edentulism	Rate	2009	432.3	502.9	368.8
Incidence	United States	Mexican	Both	Age-standardized	Edentulism	Rate	2009	356.8	415.2	304.9
Incidence	United States	Mexican	Male	Age-standardized	Edentulism	Rate	2010	266.0	315.7	225.2
Incidence	United States	Mexican	Female	Age-standardized	Edentulism	Rate	2010	427.6	503.1	361.4
Incidence	United States	Mexican	Both	Age-standardized	Edentulism	Rate	2010	350.8	414.3	297.1
Incidence	United	Mexican	Male	Age-standardized	Edentulism	Rate	2011	267.8	319.7	224.6

	States									
Incidence	United States	Mexican	Female	Age-standardized	Edentulism	Rate	2011	431.0	508.7	361.5
Incidence	United States	Mexican	Both	Age-standardized	Edentulism	Rate	2011	353.4	419.0	297.0
Incidence	United States	Mexican	Male	Age-standardized	Edentulism	Rate	2012	272.1	325.9	225.6
Incidence	United States	Mexican	Female	Age-standardized	Edentulism	Rate	2012	437.1	516.2	363.9
Incidence	United States	Mexican	Both	Age-standardized	Edentulism	Rate	2012	358.6	424.3	299.5
Incidence	United States	Mexican	Male	Age-standardized	Edentulism	Rate	2013	278.0	332.9	228.7
Incidence	United States	Mexican	Female	Age-standardized	Edentulism	Rate	2013	444.9	527.8	369.9
Incidence	United States	Mexican	Both	Age-standardized	Edentulism	Rate	2013	365.5	433.9	303.3
Incidence	United States	Mexican	Male	Age-standardized	Edentulism	Rate	2014	284.4	344.2	231.8
Incidence	United States	Mexican	Female	Age-standardized	Edentulism	Rate	2014	453.2	540.2	374.4
Incidence	United States	Mexican	Both	Age-standardized	Edentulism	Rate	2014	372.9	447.5	306.3
Incidence	United States	Mexican	Male	Age-standardized	Edentulism	Rate	2015	290.3	355.6	233.2
Incidence	United States	Mexican	Female	Age-standardized	Edentulism	Rate	2015	461.1	552.5	378.4
Incidence	United States	Mexican	Both	Age-standardized	Edentulism	Rate	2015	379.8	460.6	311.5
Incidence	United States	Mexican	Male	Age-standardized	Edentulism	Rate	2016	299.1	365.8	240.9
Incidence	United States	Mexican	Female	Age-standardized	Edentulism	Rate	2016	473.1	565.9	388.3
Incidence	United States	Mexican	Both	Age-standardized	Edentulism	Rate	2016	390.3	471.6	320.6
Incidence	United States	Mexican	Male	Age-standardized	Edentulism	Rate	2017	311.6	380.6	252.0
Incidence	United States	Mexican	Female	Age-standardized	Edentulism	Rate	2017	490.3	586.1	403.0
Incidence	United States	Mexican	Both	Age-standardized	Edentulism	Rate	2017	405.2	487.3	333.6
Incidence	United States	Mexican	Male	Age-standardized	Edentulism	Rate	2018	323.2	394.4	262.3
Incidence	United	Mexican	Female	Age-standardized	Edentulism	Rate	2018	506.4	603.6	417.1

	States									
Incidence	United States	Mexican	Both	Age-standardized	Edentulism	Rate	2018	419.2	502.1	345.5
Incidence	United States	Mexican	Male	Age-standardized	Edentulism	Rate	2019	329.3	401.8	267.8
Incidence	United States	Mexican	Female	Age-standardized	Edentulism	Rate	2019	514.9	611.4	424.9
Incidence	United States	Mexican	Both	Age-standardized	Edentulism	Rate	2019	426.6	510.4	352.5
Incidence	United States	Mexican	Male	Age-standardized	Edentulism	Rate	2020	329.4	403.9	264.4
Incidence	United States	Mexican	Female	Age-standardized	Edentulism	Rate	2020	515.6	612.2	427.1
Incidence	United States	Mexican	Both	Age-standardized	Edentulism	Rate	2020	427.1	511.6	352.1
Incidence	United States	Mexican	Male	Age-standardized	Edentulism	Rate	2021	329.6	400.2	268.3
Incidence	United States	Mexican	Female	Age-standardized	Edentulism	Rate	2021	515.3	611.6	427.7
Incidence	United States	Mexican	Both	Age-standardized	Edentulism	Rate	2021	427.2	510.7	351.9
Incidence	United States	Mexican	Male	Age-standardized	Edentulism	Rate	2022	329.9	404.7	268.9
Incidence	United States	Mexican	Female	Age-standardized	Edentulism	Rate	2022	517.2	613.5	428.5
Incidence	United States	Mexican	Both	Age-standardized	Edentulism	Rate	2022	428.5	514.4	353.4
Incidence	United States	Mexican	Male	Age-standardized	Edentulism	Rate	2023	326.9	401.1	266.4
Incidence	United States	Mexican	Female	Age-standardized	Edentulism	Rate	2023	511.8	607.4	424.0
Incidence	United States	Mexican	Both	Age-standardized	Edentulism	Rate	2023	424.2	509.5	349.7
Incidence	Argentine Republic		Male	Age-standardized	Edentulism	Rate	1990	278.1	342.9	222.1
Incidence	Argentine Republic		Female	Age-standardized	Edentulism	Rate	1990	413.9	503.6	337.5
Incidence	Argentine Republic		Both	Age-standardized	Edentulism	Rate	1990	348.5	424.6	284.9
Incidence	Argentine Republic		Male	Age-standardized	Edentulism	Rate	1991	276.0	340.3	219.9
Incidence	Argentine Republic		Female	Age-standardized	Edentulism	Rate	1991	411.9	498.4	335.9
Incidence	Argentine Republic		Both	Age-standardized	Edentulism	Rate	1991	346.6	421.3	283.2
Incidence	Argentine Republic		Male	Age-standardized	Edentulism	Rate	1992	274.0	337.6	217.2
Incidence	Argentine Republic		Female	Age-standardized	Edentulism	Rate	1992	409.8	494.5	334.3
Incidence	Argentine Republic		Both	Age-standardized	Edentulism	Rate	1992	344.6	417.7	280.6
Incidence	Argentine Republic		Male	Age-standardized	Edentulism	Rate	1993	272.1	335.8	214.5
Incidence	Argentine Republic		Female	Age-standardized	Edentulism	Rate	1993	407.6	495.0	332.5

Incidence	Argentine Republic	Both	Age-standardized	Edentulism	Rate	1993	342.6	414.4	277.3
Incidence	Argentine Republic	Male	Age-standardized	Edentulism	Rate	1994	270.2	333.8	211.3
Incidence	Argentine Republic	Female	Age-standardized	Edentulism	Rate	1994	405.4	490.4	328.6
Incidence	Argentine Republic	Both	Age-standardized	Edentulism	Rate	1994	340.6	411.6	274.5
Incidence	Argentine Republic	Male	Age-standardized	Edentulism	Rate	1995	268.5	332.0	209.0
Incidence	Argentine Republic	Female	Age-standardized	Edentulism	Rate	1995	403.1	492.0	324.9
Incidence	Argentine Republic	Both	Age-standardized	Edentulism	Rate	1995	338.6	409.7	271.9
Incidence	Argentine Republic	Male	Age-standardized	Edentulism	Rate	1996	266.6	330.2	209.1
Incidence	Argentine Republic	Female	Age-standardized	Edentulism	Rate	1996	400.2	486.0	324.7
Incidence	Argentine Republic	Both	Age-standardized	Edentulism	Rate	1996	336.2	406.6	269.2
Incidence	Argentine Republic	Male	Age-standardized	Edentulism	Rate	1997	264.4	329.3	210.2
Incidence	Argentine Republic	Female	Age-standardized	Edentulism	Rate	1997	396.6	478.6	320.7
Incidence	Argentine Republic	Both	Age-standardized	Edentulism	Rate	1997	333.3	402.4	267.8
Incidence	Argentine Republic	Male	Age-standardized	Edentulism	Rate	1998	262.3	326.1	209.1
Incidence	Argentine Republic	Female	Age-standardized	Edentulism	Rate	1998	393.0	472.6	316.8
Incidence	Argentine Republic	Both	Age-standardized	Edentulism	Rate	1998	330.4	399.9	267.0
Incidence	Argentine Republic	Male	Age-standardized	Edentulism	Rate	1999	260.7	324.1	211.2
Incidence	Argentine Republic	Female	Age-standardized	Edentulism	Rate	1999	390.1	469.2	315.5
Incidence	Argentine Republic	Both	Age-standardized	Edentulism	Rate	1999	328.1	398.6	265.5
Incidence	Argentine Republic	Male	Age-standardized	Edentulism	Rate	2000	260.0	325.6	211.8
Incidence	Argentine Republic	Female	Age-standardized	Edentulism	Rate	2000	388.7	469.3	315.4
Incidence	Argentine Republic	Both	Age-standardized	Edentulism	Rate	2000	327.0	398.2	264.2
Incidence	Argentine Republic	Male	Age-standardized	Edentulism	Rate	2001	260.2	324.6	211.7
Incidence	Argentine Republic	Female	Age-standardized	Edentulism	Rate	2001	388.5	467.3	313.0
Incidence	Argentine Republic	Both	Age-standardized	Edentulism	Rate	2001	326.9	398.1	264.6
Incidence	Argentine Republic	Male	Age-standardized	Edentulism	Rate	2002	260.7	328.0	209.7
Incidence	Argentine Republic	Female	Age-standardized	Edentulism	Rate	2002	388.6	468.3	310.8
Incidence	Argentine Republic	Both	Age-standardized	Edentulism	Rate	2002	327.3	398.5	264.8
Incidence	Argentine Republic	Male	Age-standardized	Edentulism	Rate	2003	261.4	329.4	207.6
Incidence	Argentine Republic	Female	Age-standardized	Edentulism	Rate	2003	388.9	472.9	312.9
Incidence	Argentine Republic	Both	Age-standardized	Edentulism	Rate	2003	327.7	399.3	263.2
Incidence	Argentine Republic	Male	Age-standardized	Edentulism	Rate	2004	262.0	329.5	207.9
Incidence	Argentine Republic	Female	Age-standardized	Edentulism	Rate	2004	389.1	477.5	312.5
Incidence	Argentine Republic	Both	Age-standardized	Edentulism	Rate	2004	328.1	400.3	263.6
Incidence	Argentine Republic	Male	Age-standardized	Edentulism	Rate	2005	262.2	328.0	208.6
Incidence	Argentine Republic	Female	Age-standardized	Edentulism	Rate	2005	389.0	480.3	311.0
Incidence	Argentine Republic	Both	Age-standardized	Edentulism	Rate	2005	328.1	400.6	263.7
Incidence	Argentine Republic	Male	Age-standardized	Edentulism	Rate	2006	261.5	327.0	207.0
Incidence	Argentine Republic	Female	Age-standardized	Edentulism	Rate	2006	388.1	478.7	310.9
Incidence	Argentine Republic	Both	Age-standardized	Edentulism	Rate	2006	327.2	400.0	263.1
Incidence	Argentine Republic	Male	Age-standardized	Edentulism	Rate	2007	259.9	325.0	204.7
Incidence	Argentine Republic	Female	Age-standardized	Edentulism	Rate	2007	386.2	476.9	308.9
Incidence	Argentine Republic	Both	Age-standardized	Edentulism	Rate	2007	325.5	398.3	260.2
Incidence	Argentine Republic	Male	Age-standardized	Edentulism	Rate	2008	257.8	322.3	201.9

Incidence	Argentine Republic	Female	Age-standardized	Edentulism	Rate	2008	383.9	474.4	305.1
Incidence	Argentine Republic	Both	Age-standardized	Edentulism	Rate	2008	323.2	396.6	259.0
Incidence	Argentine Republic	Male	Age-standardized	Edentulism	Rate	2009	255.7	318.3	200.3
Incidence	Argentine Republic	Female	Age-standardized	Edentulism	Rate	2009	381.4	473.4	301.5
Incidence	Argentine Republic	Both	Age-standardized	Edentulism	Rate	2009	321.0	395.6	256.2
Incidence	Argentine Republic	Male	Age-standardized	Edentulism	Rate	2010	254.1	315.0	198.9
Incidence	Argentine Republic	Female	Age-standardized	Edentulism	Rate	2010	379.2	471.4	299.7
Incidence	Argentine Republic	Both	Age-standardized	Edentulism	Rate	2010	319.0	394.1	252.4
Incidence	Argentine Republic	Male	Age-standardized	Edentulism	Rate	2011	252.7	312.0	198.0
Incidence	Argentine Republic	Female	Age-standardized	Edentulism	Rate	2011	376.9	467.2	297.0
Incidence	Argentine Republic	Both	Age-standardized	Edentulism	Rate	2011	317.2	390.9	251.0
Incidence	Argentine Republic	Male	Age-standardized	Edentulism	Rate	2012	251.2	308.9	197.9
Incidence	Argentine Republic	Female	Age-standardized	Edentulism	Rate	2012	374.0	461.7	295.4
Incidence	Argentine Republic	Both	Age-standardized	Edentulism	Rate	2012	314.9	386.7	249.1
Incidence	Argentine Republic	Male	Age-standardized	Edentulism	Rate	2013	249.7	306.9	198.6
Incidence	Argentine Republic	Female	Age-standardized	Edentulism	Rate	2013	371.2	455.9	292.4
Incidence	Argentine Republic	Both	Age-standardized	Edentulism	Rate	2013	312.8	380.4	247.4
Incidence	Argentine Republic	Male	Age-standardized	Edentulism	Rate	2014	248.6	304.5	199.5
Incidence	Argentine Republic	Female	Age-standardized	Edentulism	Rate	2014	368.9	452.3	290.6
Incidence	Argentine Republic	Both	Age-standardized	Edentulism	Rate	2014	311.1	378.6	246.8
Incidence	Argentine Republic	Male	Age-standardized	Edentulism	Rate	2015	248.0	305.6	200.4
Incidence	Argentine Republic	Female	Age-standardized	Edentulism	Rate	2015	367.9	452.5	289.3
Incidence	Argentine Republic	Both	Age-standardized	Edentulism	Rate	2015	310.3	380.0	246.8
Incidence	Argentine Republic	Male	Age-standardized	Edentulism	Rate	2016	247.9	305.6	199.9
Incidence	Argentine Republic	Female	Age-standardized	Edentulism	Rate	2016	367.9	452.6	290.5
Incidence	Argentine Republic	Both	Age-standardized	Edentulism	Rate	2016	310.3	379.9	245.8
Incidence	Argentine Republic	Male	Age-standardized	Edentulism	Rate	2017	248.0	308.7	199.0
Incidence	Argentine Republic	Female	Age-standardized	Edentulism	Rate	2017	368.1	453.5	291.4
Incidence	Argentine Republic	Both	Age-standardized	Edentulism	Rate	2017	310.5	380.1	246.5
Incidence	Argentine Republic	Male	Age-standardized	Edentulism	Rate	2018	248.2	311.0	198.1
Incidence	Argentine Republic	Female	Age-standardized	Edentulism	Rate	2018	368.5	454.2	293.1
Incidence	Argentine Republic	Both	Age-standardized	Edentulism	Rate	2018	310.8	381.9	247.1
Incidence	Argentine Republic	Male	Age-standardized	Edentulism	Rate	2019	248.2	312.7	197.3
Incidence	Argentine Republic	Female	Age-standardized	Edentulism	Rate	2019	368.7	453.0	294.7
Incidence	Argentine Republic	Both	Age-standardized	Edentulism	Rate	2019	310.9	384.4	248.1
Incidence	Argentine Republic	Male	Age-standardized	Edentulism	Rate	2020	247.5	310.3	199.4
Incidence	Argentine Republic	Female	Age-standardized	Edentulism	Rate	2020	367.6	457.4	291.3
Incidence	Argentine Republic	Both	Age-standardized	Edentulism	Rate	2020	310.1	384.7	248.9
Incidence	Argentine Republic	Male	Age-standardized	Edentulism	Rate	2021	248.2	311.9	200.1
Incidence	Argentine Republic	Female	Age-standardized	Edentulism	Rate	2021	369.7	460.7	293.2
Incidence	Argentine Republic	Both	Age-standardized	Edentulism	Rate	2021	311.6	385.4	249.8
Incidence	Argentine Republic	Male	Age-standardized	Edentulism	Rate	2022	248.9	308.4	199.0
Incidence	Argentine Republic	Female	Age-standardized	Edentulism	Rate	2022	371.2	461.3	295.8
Incidence	Argentine Republic	Both	Age-standardized	Edentulism	Rate	2022	312.7	389.2	252.3

Incidence	Argentine Republic	Male	Age-standardized	Edentulism	Rate	2023	246.9	305.9	197.5
Incidence	Argentine Republic	Female	Age-standardized	Edentulism	Rate	2023	367.8	457.2	293.2
Incidence	Argentine Republic	Both	Age-standardized	Edentulism	Rate	2023	310.1	385.9	250.2
Incidence	Republic of India	Male	Age-standardized	Edentulism	Rate	1990	283.5	352.0	230.4
Incidence	Republic of India	Female	Age-standardized	Edentulism	Rate	1990	299.5	371.3	242.6
Incidence	Republic of India	Both	Age-standardized	Edentulism	Rate	1990	291.1	361.6	236.1
Incidence	Republic of India	Male	Age-standardized	Edentulism	Rate	1991	282.8	351.2	229.7
Incidence	Republic of India	Female	Age-standardized	Edentulism	Rate	1991	299.3	371.7	242.3
Incidence	Republic of India	Both	Age-standardized	Edentulism	Rate	1991	290.6	361.0	235.6
Incidence	Republic of India	Male	Age-standardized	Edentulism	Rate	1992	281.9	350.0	228.9
Incidence	Republic of India	Female	Age-standardized	Edentulism	Rate	1992	298.7	371.2	241.8
Incidence	Republic of India	Both	Age-standardized	Edentulism	Rate	1992	289.9	360.1	234.9
Incidence	Republic of India	Male	Age-standardized	Edentulism	Rate	1993	280.8	348.7	227.9
Incidence	Republic of India	Female	Age-standardized	Edentulism	Rate	1993	298.0	370.1	241.1
Incidence	Republic of India	Both	Age-standardized	Edentulism	Rate	1993	289.0	358.9	234.1
Incidence	Republic of India	Male	Age-standardized	Edentulism	Rate	1994	279.5	346.8	226.7
Incidence	Republic of India	Female	Age-standardized	Edentulism	Rate	1994	297.0	368.7	240.3
Incidence	Republic of India	Both	Age-standardized	Edentulism	Rate	1994	287.9	357.4	233.2
Incidence	Republic of India	Male	Age-standardized	Edentulism	Rate	1995	278.0	344.5	225.5
Incidence	Republic of India	Female	Age-standardized	Edentulism	Rate	1995	295.8	367.1	239.4
Incidence	Republic of India	Both	Age-standardized	Edentulism	Rate	1995	286.6	355.8	232.2
Incidence	Republic of India	Male	Age-standardized	Edentulism	Rate	1996	273.6	338.6	221.3
Incidence	Republic of India	Female	Age-standardized	Edentulism	Rate	1996	290.9	360.4	235.3
Incidence	Republic of India	Both	Age-standardized	Edentulism	Rate	1996	282.0	349.7	228.0
Incidence	Republic of India	Male	Age-standardized	Edentulism	Rate	1997	265.2	327.4	214.4
Incidence	Republic of India	Female	Age-standardized	Edentulism	Rate	1997	280.9	346.9	227.4
Incidence	Republic of India	Both	Age-standardized	Edentulism	Rate	1997	272.8	337.4	220.5
Incidence	Republic of India	Male	Age-standardized	Edentulism	Rate	1998	255.4	315.8	206.5
Incidence	Republic of India	Female	Age-standardized	Edentulism	Rate	1998	269.0	332.5	217.5
Incidence	Republic of India	Both	Age-standardized	Edentulism	Rate	1998	262.0	323.2	212.0
Incidence	Republic of India	Male	Age-standardized	Edentulism	Rate	1999	246.9	304.9	199.7
Incidence	Republic of India	Female	Age-standardized	Edentulism	Rate	1999	258.7	319.1	208.7
Incidence	Republic of India	Both	Age-standardized	Edentulism	Rate	1999	252.7	311.6	204.1
Incidence	Republic of India	Male	Age-standardized	Edentulism	Rate	2000	242.2	298.5	195.7
Incidence	Republic of India	Female	Age-standardized	Edentulism	Rate	2000	253.2	312.9	204.1
Incidence	Republic of India	Both	Age-standardized	Edentulism	Rate	2000	247.6	305.6	199.8
Incidence	Republic of India	Male	Age-standardized	Edentulism	Rate	2001	239.7	295.7	193.9
Incidence	Republic of India	Female	Age-standardized	Edentulism	Rate	2001	251.4	310.3	202.8
Incidence	Republic of India	Both	Age-standardized	Edentulism	Rate	2001	245.5	302.9	198.4
Incidence	Republic of India	Male	Age-standardized	Edentulism	Rate	2002	236.9	292.5	191.8
Incidence	Republic of India	Female	Age-standardized	Edentulism	Rate	2002	250.0	307.3	202.6
Incidence	Republic of India	Both	Age-standardized	Edentulism	Rate	2002	243.4	300.4	197.2
Incidence	Republic of India	Male	Age-standardized	Edentulism	Rate	2003	234.6	289.8	189.7
Incidence	Republic of India	Female	Age-standardized	Edentulism	Rate	2003	249.5	305.7	202.2

Incidence	Republic of India	Both	Age-standardized	Edentulism	Rate	2003	242.0	297.7	195.7
Incidence	Republic of India	Male	Age-standardized	Edentulism	Rate	2004	233.6	288.2	188.9
Incidence	Republic of India	Female	Age-standardized	Edentulism	Rate	2004	250.1	306.7	203.4
Incidence	Republic of India	Both	Age-standardized	Edentulism	Rate	2004	241.8	297.4	196.1
Incidence	Republic of India	Male	Age-standardized	Edentulism	Rate	2005	234.8	289.5	190.1
Incidence	Republic of India	Female	Age-standardized	Edentulism	Rate	2005	252.2	308.8	205.9
Incidence	Republic of India	Both	Age-standardized	Edentulism	Rate	2005	243.6	299.8	197.9
Incidence	Republic of India	Male	Age-standardized	Edentulism	Rate	2006	257.9	314.8	211.2
Incidence	Republic of India	Female	Age-standardized	Edentulism	Rate	2006	276.5	334.3	227.4
Incidence	Republic of India	Both	Age-standardized	Edentulism	Rate	2006	267.2	323.9	219.1
Incidence	Republic of India	Male	Age-standardized	Edentulism	Rate	2007	309.1	375.3	255.4
Incidence	Republic of India	Female	Age-standardized	Edentulism	Rate	2007	329.7	397.5	272.4
Incidence	Republic of India	Both	Age-standardized	Edentulism	Rate	2007	319.6	386.5	263.8
Incidence	Republic of India	Male	Age-standardized	Edentulism	Rate	2008	369.3	447.2	306.7
Incidence	Republic of India	Female	Age-standardized	Edentulism	Rate	2008	392.4	470.9	325.4
Incidence	Republic of India	Both	Age-standardized	Edentulism	Rate	2008	381.0	459.1	316.4
Incidence	Republic of India	Male	Age-standardized	Edentulism	Rate	2009	419.2	506.7	350.3
Incidence	Republic of India	Female	Age-standardized	Edentulism	Rate	2009	444.7	530.7	371.1
Incidence	Republic of India	Both	Age-standardized	Edentulism	Rate	2009	432.2	517.7	361.0
Incidence	Republic of India	Male	Age-standardized	Edentulism	Rate	2010	439.9	529.6	369.7
Incidence	Republic of India	Female	Age-standardized	Edentulism	Rate	2010	467.0	553.6	393.4
Incidence	Republic of India	Both	Age-standardized	Edentulism	Rate	2010	453.7	540.8	382.1
Incidence	Republic of India	Male	Age-standardized	Edentulism	Rate	2011	421.0	508.3	352.7
Incidence	Republic of India	Female	Age-standardized	Edentulism	Rate	2011	447.4	531.0	375.5
Incidence	Republic of India	Both	Age-standardized	Edentulism	Rate	2011	434.4	521.1	364.3
Incidence	Republic of India	Male	Age-standardized	Edentulism	Rate	2012	375.8	452.8	315.1
Incidence	Republic of India	Female	Age-standardized	Edentulism	Rate	2012	399.4	477.4	336.3
Incidence	Republic of India	Both	Age-standardized	Edentulism	Rate	2012	387.7	466.2	324.9
Incidence	Republic of India	Male	Age-standardized	Edentulism	Rate	2013	321.5	386.2	270.2
Incidence	Republic of India	Female	Age-standardized	Edentulism	Rate	2013	341.7	408.2	288.6
Incidence	Republic of India	Both	Age-standardized	Edentulism	Rate	2013	331.6	396.8	279.0
Incidence	Republic of India	Male	Age-standardized	Edentulism	Rate	2014	274.8	329.6	231.0
Incidence	Republic of India	Female	Age-standardized	Edentulism	Rate	2014	293.1	350.5	247.8
Incidence	Republic of India	Both	Age-standardized	Edentulism	Rate	2014	283.8	339.3	239.2
Incidence	Republic of India	Male	Age-standardized	Edentulism	Rate	2015	252.7	305.7	210.6
Incidence	Republic of India	Female	Age-standardized	Edentulism	Rate	2015	272.4	326.4	229.9
Incidence	Republic of India	Both	Age-standardized	Edentulism	Rate	2015	262.3	314.2	220.2
Incidence	Republic of India	Male	Age-standardized	Edentulism	Rate	2016	248.5	299.0	208.0
Incidence	Republic of India	Female	Age-standardized	Edentulism	Rate	2016	269.9	322.5	228.6
Incidence	Republic of India	Both	Age-standardized	Edentulism	Rate	2016	259.0	308.8	219.2
Incidence	Republic of India	Male	Age-standardized	Edentulism	Rate	2017	244.9	294.4	206.2
Incidence	Republic of India	Female	Age-standardized	Edentulism	Rate	2017	265.3	317.9	224.8
Incidence	Republic of India	Both	Age-standardized	Edentulism	Rate	2017	255.0	305.6	215.3
Incidence	Republic of India	Male	Age-standardized	Edentulism	Rate	2018	242.4	291.3	204.0

Incidence	Republic of India	Female	Age-standardized	Edentulism	Rate	2018	260.8	313.1	221.0
Incidence	Republic of India	Both	Age-standardized	Edentulism	Rate	2018	251.6	301.9	213.2
Incidence	Republic of India	Male	Age-standardized	Edentulism	Rate	2019	241.6	288.2	204.4
Incidence	Republic of India	Female	Age-standardized	Edentulism	Rate	2019	258.8	308.8	220.0
Incidence	Republic of India	Both	Age-standardized	Edentulism	Rate	2019	250.2	298.5	211.7
Incidence	Republic of India	Male	Age-standardized	Edentulism	Rate	2020	241.6	287.0	204.8
Incidence	Republic of India	Female	Age-standardized	Edentulism	Rate	2020	258.7	304.5	222.4
Incidence	Republic of India	Both	Age-standardized	Edentulism	Rate	2020	250.2	294.1	214.6
Incidence	Republic of India	Male	Age-standardized	Edentulism	Rate	2021	239.9	280.9	206.2
Incidence	Republic of India	Female	Age-standardized	Edentulism	Rate	2021	260.2	303.3	223.5
Incidence	Republic of India	Both	Age-standardized	Edentulism	Rate	2021	250.1	291.4	215.0
Incidence	Republic of India	Male	Age-standardized	Edentulism	Rate	2022	240.6	287.3	204.3
Incidence	Republic of India	Female	Age-standardized	Edentulism	Rate	2022	261.5	313.7	221.7
Incidence	Republic of India	Both	Age-standardized	Edentulism	Rate	2022	251.2	300.8	212.9
Incidence	Republic of India	Male	Age-standardized	Edentulism	Rate	2023	237.2	283.1	201.3
Incidence	Republic of India	Female	Age-standardized	Edentulism	Rate	2023	257.3	308.5	218.1
Incidence	Republic of India	Both	Age-standardized	Edentulism	Rate	2023	247.4	296.2	209.6
Incidence	Canada	Male	Age-standardized	Edentulism	Rate	1990	171.3	215.6	138.2
Incidence	Canada	Female	Age-standardized	Edentulism	Rate	1990	209.8	257.1	172.6
Incidence	Canada	Both	Age-standardized	Edentulism	Rate	1990	190.8	235.4	155.8
Incidence	Canada	Male	Age-standardized	Edentulism	Rate	1991	173.8	217.8	140.7
Incidence	Canada	Female	Age-standardized	Edentulism	Rate	1991	215.0	264.2	175.8
Incidence	Canada	Both	Age-standardized	Edentulism	Rate	1991	194.7	239.7	158.5
Incidence	Canada	Male	Age-standardized	Edentulism	Rate	1992	176.0	219.3	142.5
Incidence	Canada	Female	Age-standardized	Edentulism	Rate	1992	219.6	271.2	178.8
Incidence	Canada	Both	Age-standardized	Edentulism	Rate	1992	198.2	244.2	160.9
Incidence	Canada	Male	Age-standardized	Edentulism	Rate	1993	177.8	219.2	143.5
Incidence	Canada	Female	Age-standardized	Edentulism	Rate	1993	223.5	274.5	180.7
Incidence	Canada	Both	Age-standardized	Edentulism	Rate	1993	201.0	246.3	161.9
Incidence	Canada	Male	Age-standardized	Edentulism	Rate	1994	178.9	220.7	143.5
Incidence	Canada	Female	Age-standardized	Edentulism	Rate	1994	226.1	275.4	181.9
Incidence	Canada	Both	Age-standardized	Edentulism	Rate	1994	202.9	246.7	163.0
Incidence	Canada	Male	Age-standardized	Edentulism	Rate	1995	179.2	220.9	143.1
Incidence	Canada	Female	Age-standardized	Edentulism	Rate	1995	227.1	276.3	182.2
Incidence	Canada	Both	Age-standardized	Edentulism	Rate	1995	203.6	249.4	163.3
Incidence	Canada	Male	Age-standardized	Edentulism	Rate	1996	178.9	221.4	142.8
Incidence	Canada	Female	Age-standardized	Edentulism	Rate	1996	227.1	275.6	181.4
Incidence	Canada	Both	Age-standardized	Edentulism	Rate	1996	203.4	249.5	162.6
Incidence	Canada	Male	Age-standardized	Edentulism	Rate	1997	178.3	221.6	142.5
Incidence	Canada	Female	Age-standardized	Edentulism	Rate	1997	226.9	275.0	180.7
Incidence	Canada	Both	Age-standardized	Edentulism	Rate	1997	203.0	249.5	161.7
Incidence	Canada	Male	Age-standardized	Edentulism	Rate	1998	177.6	220.4	141.7
Incidence	Canada	Female	Age-standardized	Edentulism	Rate	1998	226.7	275.3	180.0
Incidence	Canada	Both	Age-standardized	Edentulism	Rate	1998	202.5	249.3	160.9

Incidence	Canada	Male	Age-standardized	Edentulism	Rate	1999	177.0	219.6	140.2
Incidence	Canada	Female	Age-standardized	Edentulism	Rate	1999	226.6	275.6	179.6
Incidence	Canada	Both	Age-standardized	Edentulism	Rate	1999	202.2	249.1	160.3
Incidence	Canada	Male	Age-standardized	Edentulism	Rate	2000	176.8	219.3	139.3
Incidence	Canada	Female	Age-standardized	Edentulism	Rate	2000	226.6	276.1	179.0
Incidence	Canada	Both	Age-standardized	Edentulism	Rate	2000	202.1	248.9	159.6
Incidence	Canada	Male	Age-standardized	Edentulism	Rate	2001	184.1	221.7	152.2
Incidence	Canada	Female	Age-standardized	Edentulism	Rate	2001	230.4	274.7	190.7
Incidence	Canada	Both	Age-standardized	Edentulism	Rate	2001	207.6	248.5	171.1
Incidence	Canada	Male	Age-standardized	Edentulism	Rate	2002	201.6	233.6	174.7
Incidence	Canada	Female	Age-standardized	Edentulism	Rate	2002	239.1	275.1	205.0
Incidence	Canada	Both	Age-standardized	Edentulism	Rate	2002	220.6	253.4	190.1
Incidence	Canada	Male	Age-standardized	Edentulism	Rate	2003	222.5	252.4	197.7
Incidence	Canada	Female	Age-standardized	Edentulism	Rate	2003	249.4	280.8	221.0
Incidence	Canada	Both	Age-standardized	Edentulism	Rate	2003	236.1	266.4	209.7
Incidence	Canada	Male	Age-standardized	Edentulism	Rate	2004	240.0	268.3	213.8
Incidence	Canada	Female	Age-standardized	Edentulism	Rate	2004	258.0	287.6	232.9
Incidence	Canada	Both	Age-standardized	Edentulism	Rate	2004	249.1	278.2	223.1
Incidence	Canada	Male	Age-standardized	Edentulism	Rate	2005	247.3	274.3	218.7
Incidence	Canada	Female	Age-standardized	Edentulism	Rate	2005	261.7	289.0	236.0
Incidence	Canada	Both	Age-standardized	Edentulism	Rate	2005	254.6	281.8	228.3
Incidence	Canada	Male	Age-standardized	Edentulism	Rate	2006	247.2	275.3	218.6
Incidence	Canada	Female	Age-standardized	Edentulism	Rate	2006	261.6	288.8	237.0
Incidence	Canada	Both	Age-standardized	Edentulism	Rate	2006	254.5	282.1	228.6
Incidence	Canada	Male	Age-standardized	Edentulism	Rate	2007	246.8	275.1	218.1
Incidence	Canada	Female	Age-standardized	Edentulism	Rate	2007	261.2	288.3	236.1
Incidence	Canada	Both	Age-standardized	Edentulism	Rate	2007	254.1	281.4	228.6
Incidence	Canada	Male	Age-standardized	Edentulism	Rate	2008	246.1	275.0	217.3
Incidence	Canada	Female	Age-standardized	Edentulism	Rate	2008	260.7	287.5	235.4
Incidence	Canada	Both	Age-standardized	Edentulism	Rate	2008	253.5	280.5	228.0
Incidence	Canada	Male	Age-standardized	Edentulism	Rate	2009	245.2	274.1	216.2
Incidence	Canada	Female	Age-standardized	Edentulism	Rate	2009	259.9	286.4	234.8
Incidence	Canada	Both	Age-standardized	Edentulism	Rate	2009	252.6	279.3	227.4
Incidence	Canada	Male	Age-standardized	Edentulism	Rate	2010	244.0	272.2	215.2
Incidence	Canada	Female	Age-standardized	Edentulism	Rate	2010	258.9	285.4	233.5
Incidence	Canada	Both	Age-standardized	Edentulism	Rate	2010	251.6	278.1	226.4
Incidence	Canada	Male	Age-standardized	Edentulism	Rate	2011	235.4	263.4	209.0
Incidence	Canada	Female	Age-standardized	Edentulism	Rate	2011	253.3	279.8	228.3
Incidence	Canada	Both	Age-standardized	Edentulism	Rate	2011	244.4	272.7	218.7
Incidence	Canada	Male	Age-standardized	Edentulism	Rate	2012	216.6	244.3	190.9
Incidence	Canada	Female	Age-standardized	Edentulism	Rate	2012	241.3	270.8	215.0
Incidence	Canada	Both	Age-standardized	Edentulism	Rate	2012	229.1	257.3	202.9
Incidence	Canada	Male	Age-standardized	Edentulism	Rate	2013	194.7	225.8	167.2
Incidence	Canada	Female	Age-standardized	Edentulism	Rate	2013	227.1	264.7	197.6

Incidence	Canada	Both	Age-standardized	Edentulism	Rate	2013	211.1	245.0	182.5
Incidence	Canada	Male	Age-standardized	Edentulism	Rate	2014	176.4	212.1	145.8
Incidence	Canada	Female	Age-standardized	Edentulism	Rate	2014	215.1	258.2	178.9
Incidence	Canada	Both	Age-standardized	Edentulism	Rate	2014	196.0	234.2	162.5
Incidence	Canada	Male	Age-standardized	Edentulism	Rate	2015	168.8	210.5	135.0
Incidence	Canada	Female	Age-standardized	Edentulism	Rate	2015	209.4	259.4	167.2
Incidence	Canada	Both	Age-standardized	Edentulism	Rate	2015	189.4	235.3	151.2
Incidence	Canada	Male	Age-standardized	Edentulism	Rate	2016	169.3	211.8	135.9
Incidence	Canada	Female	Age-standardized	Edentulism	Rate	2016	208.4	258.6	166.3
Incidence	Canada	Both	Age-standardized	Edentulism	Rate	2016	189.1	235.0	151.1
Incidence	Canada	Male	Age-standardized	Edentulism	Rate	2017	170.3	213.1	137.2
Incidence	Canada	Female	Age-standardized	Edentulism	Rate	2017	207.6	258.2	167.0
Incidence	Canada	Both	Age-standardized	Edentulism	Rate	2017	189.2	235.8	151.3
Incidence	Canada	Male	Age-standardized	Edentulism	Rate	2018	171.3	213.9	138.9
Incidence	Canada	Female	Age-standardized	Edentulism	Rate	2018	207.1	256.5	166.6
Incidence	Canada	Both	Age-standardized	Edentulism	Rate	2018	189.4	236.8	152.2
Incidence	Canada	Male	Age-standardized	Edentulism	Rate	2019	171.8	212.9	139.7
Incidence	Canada	Female	Age-standardized	Edentulism	Rate	2019	206.9	256.9	166.2
Incidence	Canada	Both	Age-standardized	Edentulism	Rate	2019	189.6	236.7	152.9
Incidence	Canada	Male	Age-standardized	Edentulism	Rate	2020	169.4	209.5	133.4
Incidence	Canada	Female	Age-standardized	Edentulism	Rate	2020	210.7	260.2	169.3
Incidence	Canada	Both	Age-standardized	Edentulism	Rate	2020	190.4	235.0	151.6
Incidence	Canada	Male	Age-standardized	Edentulism	Rate	2021	166.2	205.8	132.7
Incidence	Canada	Female	Age-standardized	Edentulism	Rate	2021	211.1	257.9	168.9
Incidence	Canada	Both	Age-standardized	Edentulism	Rate	2021	189.0	232.6	151.3
Incidence	Canada	Male	Age-standardized	Edentulism	Rate	2022	169.2	210.2	134.7
Incidence	Canada	Female	Age-standardized	Edentulism	Rate	2022	208.6	262.7	165.2
Incidence	Canada	Both	Age-standardized	Edentulism	Rate	2022	189.2	235.9	151.2
Incidence	Canada	Male	Age-standardized	Edentulism	Rate	2023	168.7	209.5	134.4
Incidence	Canada	Female	Age-standardized	Edentulism	Rate	2023	207.8	261.7	164.6
Incidence	Canada	Both	Age-standardized	Edentulism	Rate	2023	188.5	235.1	150.7
Incidence	Kingdom of Saudi Arabia	Male	Age-standardized	Edentulism	Rate	1990	417.1	501.8	341.7
Incidence	Kingdom of Saudi Arabia	Female	Age-standardized	Edentulism	Rate	1990	294.0	358.3	237.5
Incidence	Kingdom of Saudi Arabia	Both	Age-standardized	Edentulism	Rate	1990	368.4	446.7	301.4
Incidence	Kingdom of Saudi Arabia	Male	Age-standardized	Edentulism	Rate	1991	417.4	500.9	341.1
Incidence	Kingdom of Saudi Arabia	Female	Age-standardized	Edentulism	Rate	1991	293.9	359.5	238.2
Incidence	Kingdom of Saudi Arabia	Both	Age-standardized	Edentulism	Rate	1991	368.4	446.9	300.5
Incidence	Kingdom of Saudi	Male	Age-standardized	Edentulism	Rate	1992	417.6	501.8	340.3

	Arabia								
Incidence	Kingdom of Saudi Arabia	Female	Age-standardized	Edentulism	Rate	1992	293.8	361.1	237.3
Incidence	Kingdom of Saudi Arabia	Both	Age-standardized	Edentulism	Rate	1992	368.4	446.3	299.6
Incidence	Kingdom of Saudi Arabia	Male	Age-standardized	Edentulism	Rate	1993	417.8	501.0	340.1
Incidence	Kingdom of Saudi Arabia	Female	Age-standardized	Edentulism	Rate	1993	293.7	361.9	237.0
Incidence	Kingdom of Saudi Arabia	Both	Age-standardized	Edentulism	Rate	1993	368.2	445.5	298.7
Incidence	Kingdom of Saudi Arabia	Male	Age-standardized	Edentulism	Rate	1994	417.9	500.0	340.7
Incidence	Kingdom of Saudi Arabia	Female	Age-standardized	Edentulism	Rate	1994	293.7	362.9	237.0
Incidence	Kingdom of Saudi Arabia	Both	Age-standardized	Edentulism	Rate	1994	368.0	444.7	297.9
Incidence	Kingdom of Saudi Arabia	Male	Age-standardized	Edentulism	Rate	1995	418.0	499.7	342.2
Incidence	Kingdom of Saudi Arabia	Female	Age-standardized	Edentulism	Rate	1995	293.6	364.1	237.5
Incidence	Kingdom of Saudi Arabia	Both	Age-standardized	Edentulism	Rate	1995	367.8	444.5	298.1
Incidence	Kingdom of Saudi Arabia	Male	Age-standardized	Edentulism	Rate	1996	417.9	501.5	342.3
Incidence	Kingdom of Saudi Arabia	Female	Age-standardized	Edentulism	Rate	1996	293.6	362.1	237.0
Incidence	Kingdom of Saudi Arabia	Both	Age-standardized	Edentulism	Rate	1996	367.4	445.0	298.0
Incidence	Kingdom of Saudi Arabia	Male	Age-standardized	Edentulism	Rate	1997	417.8	502.9	341.7
Incidence	Kingdom of Saudi Arabia	Female	Age-standardized	Edentulism	Rate	1997	293.6	360.1	237.0
Incidence	Kingdom of Saudi Arabia	Both	Age-standardized	Edentulism	Rate	1997	366.9	445.4	297.9
Incidence	Kingdom of Saudi Arabia	Male	Age-standardized	Edentulism	Rate	1998	417.7	504.0	341.1
Incidence	Kingdom of Saudi Arabia	Female	Age-standardized	Edentulism	Rate	1998	293.6	358.8	237.8
Incidence	Kingdom of Saudi Arabia	Both	Age-standardized	Edentulism	Rate	1998	366.5	444.6	297.7
Incidence	Kingdom of Saudi Arabia	Male	Age-standardized	Edentulism	Rate	1999	417.4	503.4	340.8
Incidence	Kingdom of Saudi	Female	Age-standardized	Edentulism	Rate	1999	293.5	358.8	237.6

	Arabia								
Incidence	Kingdom of Saudi Arabia	Both	Age-standardized	Edentulism	Rate	1999	366.1	444.7	297.5
Incidence	Kingdom of Saudi Arabia	Male	Age-standardized	Edentulism	Rate	2000	417.2	503.0	340.4
Incidence	Kingdom of Saudi Arabia	Female	Age-standardized	Edentulism	Rate	2000	293.3	358.8	238.0
Incidence	Kingdom of Saudi Arabia	Both	Age-standardized	Edentulism	Rate	2000	365.9	445.2	297.1
Incidence	Kingdom of Saudi Arabia	Male	Age-standardized	Edentulism	Rate	2001	416.5	503.6	341.6
Incidence	Kingdom of Saudi Arabia	Female	Age-standardized	Edentulism	Rate	2001	293.0	358.2	237.3
Incidence	Kingdom of Saudi Arabia	Both	Age-standardized	Edentulism	Rate	2001	365.1	443.7	297.6
Incidence	Kingdom of Saudi Arabia	Male	Age-standardized	Edentulism	Rate	2002	415.2	503.1	342.2
Incidence	Kingdom of Saudi Arabia	Female	Age-standardized	Edentulism	Rate	2002	292.4	356.6	237.4
Incidence	Kingdom of Saudi Arabia	Both	Age-standardized	Edentulism	Rate	2002	364.0	443.3	297.4
Incidence	Kingdom of Saudi Arabia	Male	Age-standardized	Edentulism	Rate	2003	413.6	501.7	342.7
Incidence	Kingdom of Saudi Arabia	Female	Age-standardized	Edentulism	Rate	2003	291.7	354.0	237.4
Incidence	Kingdom of Saudi Arabia	Both	Age-standardized	Edentulism	Rate	2003	362.8	441.0	297.6
Incidence	Kingdom of Saudi Arabia	Male	Age-standardized	Edentulism	Rate	2004	411.9	500.8	341.5
Incidence	Kingdom of Saudi Arabia	Female	Age-standardized	Edentulism	Rate	2004	290.9	354.0	237.2
Incidence	Kingdom of Saudi Arabia	Both	Age-standardized	Edentulism	Rate	2004	361.6	439.6	297.1
Incidence	Kingdom of Saudi Arabia	Male	Age-standardized	Edentulism	Rate	2005	410.2	498.8	341.1
Incidence	Kingdom of Saudi Arabia	Female	Age-standardized	Edentulism	Rate	2005	290.1	356.0	236.9
Incidence	Kingdom of Saudi Arabia	Both	Age-standardized	Edentulism	Rate	2005	360.8	438.5	295.5
Incidence	Kingdom of Saudi Arabia	Male	Age-standardized	Edentulism	Rate	2006	408.1	493.8	338.1
Incidence	Kingdom of Saudi Arabia	Female	Age-standardized	Edentulism	Rate	2006	288.9	352.8	235.5
Incidence	Kingdom of Saudi	Both	Age-standardized	Edentulism	Rate	2006	359.2	434.6	293.1

	Arabia								
Incidence	Kingdom of Saudi Arabia	Male	Age-standardized	Edentulism	Rate	2007	405.2	488.0	334.3
Incidence	Kingdom of Saudi Arabia	Female	Age-standardized	Edentulism	Rate	2007	287.0	348.4	233.2
Incidence	Kingdom of Saudi Arabia	Both	Age-standardized	Edentulism	Rate	2007	356.9	429.9	291.2
Incidence	Kingdom of Saudi Arabia	Male	Age-standardized	Edentulism	Rate	2008	402.0	481.3	330.3
Incidence	Kingdom of Saudi Arabia	Female	Age-standardized	Edentulism	Rate	2008	284.8	344.5	230.7
Incidence	Kingdom of Saudi Arabia	Both	Age-standardized	Edentulism	Rate	2008	354.3	425.6	289.3
Incidence	Kingdom of Saudi Arabia	Male	Age-standardized	Edentulism	Rate	2009	399.0	476.7	325.8
Incidence	Kingdom of Saudi Arabia	Female	Age-standardized	Edentulism	Rate	2009	282.8	343.0	228.2
Incidence	Kingdom of Saudi Arabia	Both	Age-standardized	Edentulism	Rate	2009	352.0	422.2	287.0
Incidence	Kingdom of Saudi Arabia	Male	Age-standardized	Edentulism	Rate	2010	396.8	473.9	320.9
Incidence	Kingdom of Saudi Arabia	Female	Age-standardized	Edentulism	Rate	2010	281.4	341.9	226.4
Incidence	Kingdom of Saudi Arabia	Both	Age-standardized	Edentulism	Rate	2010	350.5	420.8	284.8
Incidence	Kingdom of Saudi Arabia	Male	Age-standardized	Edentulism	Rate	2011	395.1	472.8	322.4
Incidence	Kingdom of Saudi Arabia	Female	Age-standardized	Edentulism	Rate	2011	280.3	340.7	225.9
Incidence	Kingdom of Saudi Arabia	Both	Age-standardized	Edentulism	Rate	2011	349.1	419.2	284.4
Incidence	Kingdom of Saudi Arabia	Male	Age-standardized	Edentulism	Rate	2012	393.2	470.3	321.8
Incidence	Kingdom of Saudi Arabia	Female	Age-standardized	Edentulism	Rate	2012	279.2	339.4	225.5
Incidence	Kingdom of Saudi Arabia	Both	Age-standardized	Edentulism	Rate	2012	347.5	417.4	283.5
Incidence	Kingdom of Saudi Arabia	Male	Age-standardized	Edentulism	Rate	2013	391.5	469.1	321.9
Incidence	Kingdom of Saudi Arabia	Female	Age-standardized	Edentulism	Rate	2013	278.1	338.0	223.9
Incidence	Kingdom of Saudi Arabia	Both	Age-standardized	Edentulism	Rate	2013	346.1	416.1	282.1
Incidence	Kingdom of Saudi	Male	Age-standardized	Edentulism	Rate	2014	390.2	469.8	321.0

	Arabia								
Incidence	Kingdom of Saudi Arabia	Female	Age-standardized	Edentulism	Rate	2014	277.3	338.2	222.9
Incidence	Kingdom of Saudi Arabia	Both	Age-standardized	Edentulism	Rate	2014	345.2	416.3	280.9
Incidence	Kingdom of Saudi Arabia	Male	Age-standardized	Edentulism	Rate	2015	389.6	470.8	320.4
Incidence	Kingdom of Saudi Arabia	Female	Age-standardized	Edentulism	Rate	2015	276.9	340.1	222.7
Incidence	Kingdom of Saudi Arabia	Both	Age-standardized	Edentulism	Rate	2015	344.9	417.4	280.4
Incidence	Kingdom of Saudi Arabia	Male	Age-standardized	Edentulism	Rate	2016	389.7	470.7	318.6
Incidence	Kingdom of Saudi Arabia	Female	Age-standardized	Edentulism	Rate	2016	277.0	337.8	222.9
Incidence	Kingdom of Saudi Arabia	Both	Age-standardized	Edentulism	Rate	2016	345.1	417.3	280.1
Incidence	Kingdom of Saudi Arabia	Male	Age-standardized	Edentulism	Rate	2017	390.2	471.4	317.0
Incidence	Kingdom of Saudi Arabia	Female	Age-standardized	Edentulism	Rate	2017	277.2	338.9	224.3
Incidence	Kingdom of Saudi Arabia	Both	Age-standardized	Edentulism	Rate	2017	345.7	417.6	280.2
Incidence	Kingdom of Saudi Arabia	Male	Age-standardized	Edentulism	Rate	2018	390.8	473.3	317.1
Incidence	Kingdom of Saudi Arabia	Female	Age-standardized	Edentulism	Rate	2018	277.5	338.4	225.8
Incidence	Kingdom of Saudi Arabia	Both	Age-standardized	Edentulism	Rate	2018	346.4	419.2	280.8
Incidence	Kingdom of Saudi Arabia	Male	Age-standardized	Edentulism	Rate	2019	391.2	474.9	316.8
Incidence	Kingdom of Saudi Arabia	Female	Age-standardized	Edentulism	Rate	2019	277.9	339.7	227.8
Incidence	Kingdom of Saudi Arabia	Both	Age-standardized	Edentulism	Rate	2019	347.1	421.4	281.9
Incidence	Kingdom of Saudi Arabia	Male	Age-standardized	Edentulism	Rate	2020	391.2	477.1	320.3
Incidence	Kingdom of Saudi Arabia	Female	Age-standardized	Edentulism	Rate	2020	279.1	342.0	224.9
Incidence	Kingdom of Saudi Arabia	Both	Age-standardized	Edentulism	Rate	2020	348.0	423.6	284.1
Incidence	Kingdom of Saudi Arabia	Male	Age-standardized	Edentulism	Rate	2021	393.5	481.1	323.7
Incidence	Kingdom of Saudi	Female	Age-standardized	Edentulism	Rate	2021	280.0	348.9	223.1

	Arabia								
Incidence	Kingdom of Saudi Arabia	Both	Age-standardized	Edentulism	Rate	2021	350.1	430.1	285.5
Incidence	Kingdom of Saudi Arabia	Male	Age-standardized	Edentulism	Rate	2022	394.5	477.3	317.6
Incidence	Kingdom of Saudi Arabia	Female	Age-standardized	Edentulism	Rate	2022	281.2	344.5	225.1
Incidence	Kingdom of Saudi Arabia	Both	Age-standardized	Edentulism	Rate	2022	351.6	426.4	282.3
Incidence	Kingdom of Saudi Arabia	Male	Age-standardized	Edentulism	Rate	2023	390.5	472.4	314.5
Incidence	Kingdom of Saudi Arabia	Female	Age-standardized	Edentulism	Rate	2023	277.9	340.5	222.5
Incidence	Kingdom of Saudi Arabia	Both	Age-standardized	Edentulism	Rate	2023	348.4	422.4	279.8
Incidence	Republic of Turkey	Male	Age-standardized	Edentulism	Rate	1990	526.3	623.6	437.8
Incidence	Republic of Turkey	Female	Age-standardized	Edentulism	Rate	1990	462.2	548.8	384.9
Incidence	Republic of Turkey	Both	Age-standardized	Edentulism	Rate	1990	493.4	585.9	408.7
Incidence	Republic of Turkey	Male	Age-standardized	Edentulism	Rate	1991	525.7	622.5	437.9
Incidence	Republic of Turkey	Female	Age-standardized	Edentulism	Rate	1991	461.7	547.6	382.7
Incidence	Republic of Turkey	Both	Age-standardized	Edentulism	Rate	1991	492.8	585.1	408.2
Incidence	Republic of Turkey	Male	Age-standardized	Edentulism	Rate	1992	525.0	620.0	437.6
Incidence	Republic of Turkey	Female	Age-standardized	Edentulism	Rate	1992	461.1	549.7	380.9
Incidence	Republic of Turkey	Both	Age-standardized	Edentulism	Rate	1992	492.0	583.4	407.7
Incidence	Republic of Turkey	Male	Age-standardized	Edentulism	Rate	1993	524.2	618.1	438.4
Incidence	Republic of Turkey	Female	Age-standardized	Edentulism	Rate	1993	460.4	552.4	380.2
Incidence	Republic of Turkey	Both	Age-standardized	Edentulism	Rate	1993	491.2	584.1	407.1
Incidence	Republic of Turkey	Male	Age-standardized	Edentulism	Rate	1994	523.4	617.7	440.0
Incidence	Republic of Turkey	Female	Age-standardized	Edentulism	Rate	1994	459.7	552.3	378.5
Incidence	Republic of Turkey	Both	Age-standardized	Edentulism	Rate	1994	490.4	582.8	407.7
Incidence	Republic of Turkey	Male	Age-standardized	Edentulism	Rate	1995	522.6	618.5	440.2
Incidence	Republic of Turkey	Female	Age-standardized	Edentulism	Rate	1995	459.1	553.2	378.7
Incidence	Republic of Turkey	Both	Age-standardized	Edentulism	Rate	1995	489.7	582.4	408.4
Incidence	Republic of Turkey	Male	Age-standardized	Edentulism	Rate	1996	521.6	616.5	439.2
Incidence	Republic of Turkey	Female	Age-standardized	Edentulism	Rate	1996	458.2	551.3	377.6
Incidence	Republic of Turkey	Both	Age-standardized	Edentulism	Rate	1996	488.6	581.9	406.8
Incidence	Republic of Turkey	Male	Age-standardized	Edentulism	Rate	1997	520.0	613.6	436.4
Incidence	Republic of Turkey	Female	Age-standardized	Edentulism	Rate	1997	456.8	548.9	376.6
Incidence	Republic of Turkey	Both	Age-standardized	Edentulism	Rate	1997	487.1	581.1	404.8
Incidence	Republic of Turkey	Male	Age-standardized	Edentulism	Rate	1998	518.2	612.2	433.2
Incidence	Republic of Turkey	Female	Age-standardized	Edentulism	Rate	1998	455.1	546.5	375.8
Incidence	Republic of Turkey	Both	Age-standardized	Edentulism	Rate	1998	485.3	578.2	403.0
Incidence	Republic of Turkey	Male	Age-standardized	Edentulism	Rate	1999	516.5	610.5	430.9
Incidence	Republic of Turkey	Female	Age-standardized	Edentulism	Rate	1999	453.6	544.2	375.0

Incidence	Republic of Turkey	Both	Age-standardized	Edentulism	Rate	1999	483.6	575.3	400.8
Incidence	Republic of Turkey	Male	Age-standardized	Edentulism	Rate	2000	515.2	611.6	429.4
Incidence	Republic of Turkey	Female	Age-standardized	Edentulism	Rate	2000	452.5	542.0	374.4
Incidence	Republic of Turkey	Both	Age-standardized	Edentulism	Rate	2000	482.4	574.1	399.8
Incidence	Republic of Turkey	Male	Age-standardized	Edentulism	Rate	2001	518.7	603.8	443.4
Incidence	Republic of Turkey	Female	Age-standardized	Edentulism	Rate	2001	465.1	542.4	395.2
Incidence	Republic of Turkey	Both	Age-standardized	Edentulism	Rate	2001	490.4	567.7	417.5
Incidence	Republic of Turkey	Male	Age-standardized	Edentulism	Rate	2002	528.6	598.1	465.6
Incidence	Republic of Turkey	Female	Age-standardized	Edentulism	Rate	2002	496.5	561.6	433.7
Incidence	Republic of Turkey	Both	Age-standardized	Edentulism	Rate	2002	511.0	578.7	445.1
Incidence	Republic of Turkey	Male	Age-standardized	Edentulism	Rate	2003	541.0	601.2	483.8
Incidence	Republic of Turkey	Female	Age-standardized	Edentulism	Rate	2003	534.8	591.9	476.8
Incidence	Republic of Turkey	Both	Age-standardized	Edentulism	Rate	2003	536.2	593.6	478.2
Incidence	Republic of Turkey	Male	Age-standardized	Edentulism	Rate	2004	551.7	605.1	498.2
Incidence	Republic of Turkey	Female	Age-standardized	Edentulism	Rate	2004	568.2	618.7	516.2
Incidence	Republic of Turkey	Both	Age-standardized	Edentulism	Rate	2004	558.1	609.1	506.8
Incidence	Republic of Turkey	Male	Age-standardized	Edentulism	Rate	2005	556.8	606.6	510.2
Incidence	Republic of Turkey	Female	Age-standardized	Edentulism	Rate	2005	584.9	632.8	530.5
Incidence	Republic of Turkey	Both	Age-standardized	Edentulism	Rate	2005	569.0	615.4	520.2
Incidence	Republic of Turkey	Male	Age-standardized	Edentulism	Rate	2006	552.4	596.2	512.2
Incidence	Republic of Turkey	Female	Age-standardized	Edentulism	Rate	2006	585.7	627.1	541.2
Incidence	Republic of Turkey	Both	Age-standardized	Edentulism	Rate	2006	567.4	607.3	525.4
Incidence	Republic of Turkey	Male	Age-standardized	Edentulism	Rate	2007	540.3	575.3	504.5
Incidence	Republic of Turkey	Female	Age-standardized	Edentulism	Rate	2007	581.0	613.8	547.3
Incidence	Republic of Turkey	Both	Age-standardized	Edentulism	Rate	2007	559.4	592.0	523.6
Incidence	Republic of Turkey	Male	Age-standardized	Edentulism	Rate	2008	525.3	555.8	495.1
Incidence	Republic of Turkey	Female	Age-standardized	Edentulism	Rate	2008	573.0	602.3	545.1
Incidence	Republic of Turkey	Both	Age-standardized	Edentulism	Rate	2008	548.3	576.5	521.5
Incidence	Republic of Turkey	Male	Age-standardized	Edentulism	Rate	2009	511.9	537.5	483.2
Incidence	Republic of Turkey	Female	Age-standardized	Edentulism	Rate	2009	563.9	587.3	536.2
Incidence	Republic of Turkey	Both	Age-standardized	Edentulism	Rate	2009	537.6	561.3	512.3
Incidence	Republic of Turkey	Male	Age-standardized	Edentulism	Rate	2010	505.0	529.9	478.4
Incidence	Republic of Turkey	Female	Age-standardized	Edentulism	Rate	2010	556.2	580.8	530.3
Incidence	Republic of Turkey	Both	Age-standardized	Edentulism	Rate	2010	530.4	554.4	504.1
Incidence	Republic of Turkey	Male	Age-standardized	Edentulism	Rate	2011	501.3	529.0	469.4
Incidence	Republic of Turkey	Female	Age-standardized	Edentulism	Rate	2011	538.7	564.1	509.0
Incidence	Republic of Turkey	Both	Age-standardized	Edentulism	Rate	2011	519.7	545.3	492.0
Incidence	Republic of Turkey	Male	Age-standardized	Edentulism	Rate	2012	495.6	533.6	455.2
Incidence	Republic of Turkey	Female	Age-standardized	Edentulism	Rate	2012	505.9	540.1	469.7
Incidence	Republic of Turkey	Both	Age-standardized	Edentulism	Rate	2012	500.2	536.1	464.8
Incidence	Republic of Turkey	Male	Age-standardized	Edentulism	Rate	2013	489.5	544.5	438.0
Incidence	Republic of Turkey	Female	Age-standardized	Edentulism	Rate	2013	468.7	520.3	424.1
Incidence	Republic of Turkey	Both	Age-standardized	Edentulism	Rate	2013	478.2	529.4	430.6
Incidence	Republic of Turkey	Male	Age-standardized	Edentulism	Rate	2014	484.3	557.3	418.8

Incidence	Republic of Turkey	Female	Age-standardized	Edentulism	Rate	2014	437.9	507.0	378.3
Incidence	Republic of Turkey	Both	Age-standardized	Edentulism	Rate	2014	459.9	530.5	397.6
Incidence	Republic of Turkey	Male	Age-standardized	Edentulism	Rate	2015	481.5	575.6	399.1
Incidence	Republic of Turkey	Female	Age-standardized	Edentulism	Rate	2015	424.1	514.4	350.2
Incidence	Republic of Turkey	Both	Age-standardized	Edentulism	Rate	2015	451.6	537.1	376.4
Incidence	Republic of Turkey	Male	Age-standardized	Edentulism	Rate	2016	480.3	569.3	398.3
Incidence	Republic of Turkey	Female	Age-standardized	Edentulism	Rate	2016	422.8	509.8	348.0
Incidence	Republic of Turkey	Both	Age-standardized	Edentulism	Rate	2016	450.4	535.9	373.2
Incidence	Republic of Turkey	Male	Age-standardized	Edentulism	Rate	2017	478.7	563.2	396.1
Incidence	Republic of Turkey	Female	Age-standardized	Edentulism	Rate	2017	421.7	505.6	345.3
Incidence	Republic of Turkey	Both	Age-standardized	Edentulism	Rate	2017	449.1	533.1	370.6
Incidence	Republic of Turkey	Male	Age-standardized	Edentulism	Rate	2018	477.2	563.0	395.9
Incidence	Republic of Turkey	Female	Age-standardized	Edentulism	Rate	2018	420.9	503.8	342.9
Incidence	Republic of Turkey	Both	Age-standardized	Edentulism	Rate	2018	448.1	531.5	369.1
Incidence	Republic of Turkey	Male	Age-standardized	Edentulism	Rate	2019	476.1	561.5	393.4
Incidence	Republic of Turkey	Female	Age-standardized	Edentulism	Rate	2019	420.3	504.4	341.2
Incidence	Republic of Turkey	Both	Age-standardized	Edentulism	Rate	2019	447.3	530.4	367.2
Incidence	Republic of Turkey	Male	Age-standardized	Edentulism	Rate	2020	474.8	572.2	397.4
Incidence	Republic of Turkey	Female	Age-standardized	Edentulism	Rate	2020	420.0	509.9	343.5
Incidence	Republic of Turkey	Both	Age-standardized	Edentulism	Rate	2020	446.4	538.3	370.6
Incidence	Republic of Turkey	Male	Age-standardized	Edentulism	Rate	2021	475.1	565.0	400.2
Incidence	Republic of Turkey	Female	Age-standardized	Edentulism	Rate	2021	422.0	503.6	349.4
Incidence	Republic of Turkey	Both	Age-standardized	Edentulism	Rate	2021	447.5	531.3	372.5
Incidence	Republic of Turkey	Male	Age-standardized	Edentulism	Rate	2022	480.2	574.9	391.0
Incidence	Republic of Turkey	Female	Age-standardized	Edentulism	Rate	2022	422.8	507.6	350.1
Incidence	Republic of Turkey	Both	Age-standardized	Edentulism	Rate	2022	450.4	539.9	369.4
Incidence	Republic of Turkey	Male	Age-standardized	Edentulism	Rate	2023	473.1	566.6	385.3
Incidence	Republic of Turkey	Female	Age-standardized	Edentulism	Rate	2023	415.8	499.1	344.4
Incidence	Republic of Turkey	Both	Age-standardized	Edentulism	Rate	2023	443.4	531.6	364.0
Incidence	Republic of South Africa	Male	Age-standardized	Edentulism	Rate	1990	358.6	418.9	303.2
Incidence	Republic of South Africa	Female	Age-standardized	Edentulism	Rate	1990	370.6	433.0	311.1
Incidence	Republic of South Africa	Both	Age-standardized	Edentulism	Rate	1990	364.4	426.5	306.6
Incidence	Republic of South Africa	Male	Age-standardized	Edentulism	Rate	1991	350.7	410.6	295.2
Incidence	Republic of South Africa	Female	Age-standardized	Edentulism	Rate	1991	357.6	418.4	299.5
Incidence	Republic of South Africa	Both	Age-standardized	Edentulism	Rate	1991	353.9	414.9	297.0
Incidence	Republic of South Africa	Male	Age-standardized	Edentulism	Rate	1992	344.1	403.8	289.1
Incidence	Republic of South	Female	Age-standardized	Edentulism	Rate	1992	347.1	406.6	290.2

	Africa								
Incidence	Republic of South Africa	Both	Age-standardized	Edentulism	Rate	1992	345.4	405.4	289.3
Incidence	Republic of South Africa	Male	Age-standardized	Edentulism	Rate	1993	338.8	398.6	284.2
Incidence	Republic of South Africa	Female	Age-standardized	Edentulism	Rate	1993	339.2	397.6	283.4
Incidence	Republic of South Africa	Both	Age-standardized	Edentulism	Rate	1993	338.8	398.4	283.6
Incidence	Republic of South Africa	Male	Age-standardized	Edentulism	Rate	1994	334.9	394.9	280.9
Incidence	Republic of South Africa	Female	Age-standardized	Edentulism	Rate	1994	334.1	392.1	279.9
Incidence	Republic of South Africa	Both	Age-standardized	Edentulism	Rate	1994	334.3	393.7	280.0
Incidence	Republic of South Africa	Male	Age-standardized	Edentulism	Rate	1995	332.4	392.9	279.2
Incidence	Republic of South Africa	Female	Age-standardized	Edentulism	Rate	1995	332.0	390.3	278.4
Incidence	Republic of South Africa	Both	Age-standardized	Edentulism	Rate	1995	332.1	390.9	278.7
Incidence	Republic of South Africa	Male	Age-standardized	Edentulism	Rate	1996	333.6	395.2	280.7
Incidence	Republic of South Africa	Female	Age-standardized	Edentulism	Rate	1996	338.2	399.4	284.1
Incidence	Republic of South Africa	Both	Age-standardized	Edentulism	Rate	1996	336.0	397.6	282.0
Incidence	Republic of South Africa	Male	Age-standardized	Edentulism	Rate	1997	339.0	401.3	285.7
Incidence	Republic of South Africa	Female	Age-standardized	Edentulism	Rate	1997	353.9	420.1	298.6
Incidence	Republic of South Africa	Both	Age-standardized	Edentulism	Rate	1997	347.0	411.2	292.9
Incidence	Republic of South Africa	Male	Age-standardized	Edentulism	Rate	1998	346.1	410.4	292.6
Incidence	Republic of South Africa	Female	Age-standardized	Edentulism	Rate	1998	372.8	444.4	315.1
Incidence	Republic of South Africa	Both	Age-standardized	Edentulism	Rate	1998	360.5	428.7	305.5
Incidence	Republic of South Africa	Male	Age-standardized	Edentulism	Rate	1999	352.2	418.3	298.6
Incidence	Republic of South Africa	Female	Age-standardized	Edentulism	Rate	1999	388.8	464.6	327.8
Incidence	Republic of South	Both	Age-standardized	Edentulism	Rate	1999	371.9	443.3	315.3

	Africa								
Incidence	Republic of South Africa	Male	Age-standardized	Edentulism	Rate	2000	354.7	421.1	300.9
Incidence	Republic of South Africa	Female	Age-standardized	Edentulism	Rate	2000	395.4	472.9	333.5
Incidence	Republic of South Africa	Both	Age-standardized	Edentulism	Rate	2000	376.8	449.0	319.4
Incidence	Republic of South Africa	Male	Age-standardized	Edentulism	Rate	2001	353.7	418.9	298.8
Incidence	Republic of South Africa	Female	Age-standardized	Edentulism	Rate	2001	389.3	464.5	328.9
Incidence	Republic of South Africa	Both	Age-standardized	Edentulism	Rate	2001	373.0	443.6	316.4
Incidence	Republic of South Africa	Male	Age-standardized	Edentulism	Rate	2002	351.1	415.5	294.1
Incidence	Republic of South Africa	Female	Age-standardized	Edentulism	Rate	2002	374.7	443.4	313.6
Incidence	Republic of South Africa	Both	Age-standardized	Edentulism	Rate	2002	363.9	430.9	304.5
Incidence	Republic of South Africa	Male	Age-standardized	Edentulism	Rate	2003	347.4	406.8	291.8
Incidence	Republic of South Africa	Female	Age-standardized	Edentulism	Rate	2003	356.7	413.9	298.9
Incidence	Republic of South Africa	Both	Age-standardized	Edentulism	Rate	2003	352.4	409.6	295.6
Incidence	Republic of South Africa	Male	Age-standardized	Edentulism	Rate	2004	343.5	400.9	289.1
Incidence	Republic of South Africa	Female	Age-standardized	Edentulism	Rate	2004	340.6	399.2	286.6
Incidence	Republic of South Africa	Both	Age-standardized	Edentulism	Rate	2004	341.9	400.0	287.6
Incidence	Republic of South Africa	Male	Age-standardized	Edentulism	Rate	2005	339.8	401.1	285.9
Incidence	Republic of South Africa	Female	Age-standardized	Edentulism	Rate	2005	331.7	391.9	279.5
Incidence	Republic of South Africa	Both	Age-standardized	Edentulism	Rate	2005	335.4	396.9	282.1
Incidence	Republic of South Africa	Male	Age-standardized	Edentulism	Rate	2006	328.2	386.3	275.4
Incidence	Republic of South Africa	Female	Age-standardized	Edentulism	Rate	2006	320.5	378.7	269.8
Incidence	Republic of South Africa	Both	Age-standardized	Edentulism	Rate	2006	324.0	382.3	272.1
Incidence	Republic of South	Male	Age-standardized	Edentulism	Rate	2007	305.5	356.5	256.5

	Africa								
Incidence	Republic of South Africa	Female	Age-standardized	Edentulism	Rate	2007	299.1	350.7	251.8
Incidence	Republic of South Africa	Both	Age-standardized	Edentulism	Rate	2007	301.8	353.2	253.3
Incidence	Republic of South Africa	Male	Age-standardized	Edentulism	Rate	2008	279.5	329.5	235.2
Incidence	Republic of South Africa	Female	Age-standardized	Edentulism	Rate	2008	274.9	326.0	232.1
Incidence	Republic of South Africa	Both	Age-standardized	Edentulism	Rate	2008	276.6	327.3	233.5
Incidence	Republic of South Africa	Male	Age-standardized	Edentulism	Rate	2009	258.3	307.7	217.5
Incidence	Republic of South Africa	Female	Age-standardized	Edentulism	Rate	2009	255.3	304.7	215.8
Incidence	Republic of South Africa	Both	Age-standardized	Edentulism	Rate	2009	256.1	305.4	216.3
Incidence	Republic of South Africa	Male	Age-standardized	Edentulism	Rate	2010	249.9	299.2	209.6
Incidence	Republic of South Africa	Female	Age-standardized	Edentulism	Rate	2010	247.9	296.9	209.0
Incidence	Republic of South Africa	Both	Age-standardized	Edentulism	Rate	2010	248.2	296.9	209.0
Incidence	Republic of South Africa	Male	Age-standardized	Edentulism	Rate	2011	258.2	308.2	217.9
Incidence	Republic of South Africa	Female	Age-standardized	Edentulism	Rate	2011	256.1	306.2	216.2
Incidence	Republic of South Africa	Both	Age-standardized	Edentulism	Rate	2011	256.5	306.5	216.7
Incidence	Republic of South Africa	Male	Age-standardized	Edentulism	Rate	2012	276.9	330.2	235.2
Incidence	Republic of South Africa	Female	Age-standardized	Edentulism	Rate	2012	274.1	327.6	231.5
Incidence	Republic of South Africa	Both	Age-standardized	Edentulism	Rate	2012	274.9	328.3	232.9
Incidence	Republic of South Africa	Male	Age-standardized	Edentulism	Rate	2013	299.1	355.3	252.0
Incidence	Republic of South Africa	Female	Age-standardized	Edentulism	Rate	2013	295.3	350.9	247.9
Incidence	Republic of South Africa	Both	Age-standardized	Edentulism	Rate	2013	296.7	353.0	249.6
Incidence	Republic of South Africa	Male	Age-standardized	Edentulism	Rate	2014	317.7	375.6	266.5
Incidence	Republic of South	Female	Age-standardized	Edentulism	Rate	2014	313.2	369.6	262.5

	Africa								
Incidence	Republic of South Africa	Both	Age-standardized	Edentulism	Rate	2014	315.1	372.4	264.3
Incidence	Republic of South Africa	Male	Age-standardized	Edentulism	Rate	2015	325.7	383.7	273.2
Incidence	Republic of South Africa	Female	Age-standardized	Edentulism	Rate	2015	321.0	377.2	268.7
Incidence	Republic of South Africa	Both	Age-standardized	Edentulism	Rate	2015	323.0	380.1	270.4
Incidence	Republic of South Africa	Male	Age-standardized	Edentulism	Rate	2016	325.9	383.0	273.5
Incidence	Republic of South Africa	Female	Age-standardized	Edentulism	Rate	2016	321.3	378.0	268.9
Incidence	Republic of South Africa	Both	Age-standardized	Edentulism	Rate	2016	323.3	380.0	271.0
Incidence	Republic of South Africa	Male	Age-standardized	Edentulism	Rate	2017	325.8	382.0	273.7
Incidence	Republic of South Africa	Female	Age-standardized	Edentulism	Rate	2017	321.3	378.1	269.6
Incidence	Republic of South Africa	Both	Age-standardized	Edentulism	Rate	2017	323.3	379.4	271.4
Incidence	Republic of South Africa	Male	Age-standardized	Edentulism	Rate	2018	325.7	381.1	273.8
Incidence	Republic of South Africa	Female	Age-standardized	Edentulism	Rate	2018	321.1	378.9	269.9
Incidence	Republic of South Africa	Both	Age-standardized	Edentulism	Rate	2018	323.1	379.0	271.7
Incidence	Republic of South Africa	Male	Age-standardized	Edentulism	Rate	2019	325.6	380.3	273.5
Incidence	Republic of South Africa	Female	Age-standardized	Edentulism	Rate	2019	321.0	379.4	269.9
Incidence	Republic of South Africa	Both	Age-standardized	Edentulism	Rate	2019	323.0	379.3	272.1
Incidence	Republic of South Africa	Male	Age-standardized	Edentulism	Rate	2020	326.2	383.1	274.9
Incidence	Republic of South Africa	Female	Age-standardized	Edentulism	Rate	2020	322.1	380.7	268.8
Incidence	Republic of South Africa	Both	Age-standardized	Edentulism	Rate	2020	324.0	381.9	271.2
Incidence	Republic of South Africa	Male	Age-standardized	Edentulism	Rate	2021	325.6	383.0	272.5
Incidence	Republic of South Africa	Female	Age-standardized	Edentulism	Rate	2021	321.6	378.6	267.9
Incidence	Republic of South	Both	Age-standardized	Edentulism	Rate	2021	323.3	380.1	270.0

	Africa								
Incidence	Republic of South Africa	Male	Age-standardized	Edentulism	Rate	2022	325.2	383.6	273.4
Incidence	Republic of South Africa	Female	Age-standardized	Edentulism	Rate	2022	323.0	381.7	271.5
Incidence	Republic of South Africa	Both	Age-standardized	Edentulism	Rate	2022	323.8	382.0	272.4
Incidence	Republic of South Africa	Male	Age-standardized	Edentulism	Rate	2023	323.3	381.3	271.9
Incidence	Republic of South Africa	Female	Age-standardized	Edentulism	Rate	2023	320.8	379.2	269.7
Incidence	Republic of South Africa	Both	Age-standardized	Edentulism	Rate	2023	321.8	379.5	270.7
Incidence	European Union	Male	Age-standardized	Edentulism	Rate	1990	327.3	395.0	268.7
Incidence	European Union	Female	Age-standardized	Edentulism	Rate	1990	391.4	467.7	326.3
Incidence	European Union	Both	Age-standardized	Edentulism	Rate	1990	359.8	430.9	297.2
Incidence	European Union	Male	Age-standardized	Edentulism	Rate	1991	324.2	388.4	268.8
Incidence	European Union	Female	Age-standardized	Edentulism	Rate	1991	387.0	458.8	324.5
Incidence	European Union	Both	Age-standardized	Edentulism	Rate	1991	355.9	422.8	296.8
Incidence	European Union	Male	Age-standardized	Edentulism	Rate	1992	321.6	382.9	268.7
Incidence	European Union	Female	Age-standardized	Edentulism	Rate	1992	383.2	451.2	323.2
Incidence	European Union	Both	Age-standardized	Edentulism	Rate	1992	352.7	415.7	296.1
Incidence	European Union	Male	Age-standardized	Edentulism	Rate	1993	319.6	378.8	268.2
Incidence	European Union	Female	Age-standardized	Edentulism	Rate	1993	380.3	445.1	323.0
Incidence	European Union	Both	Age-standardized	Edentulism	Rate	1993	350.2	409.9	296.0
Incidence	European Union	Male	Age-standardized	Edentulism	Rate	1994	318.4	375.2	268.8
Incidence	European Union	Female	Age-standardized	Edentulism	Rate	1994	378.4	440.6	323.1
Incidence	European Union	Both	Age-standardized	Edentulism	Rate	1994	348.6	405.6	296.5
Incidence	European Union	Male	Age-standardized	Edentulism	Rate	1995	318.1	373.3	269.9
Incidence	European Union	Female	Age-standardized	Edentulism	Rate	1995	377.9	438.1	324.3
Incidence	European Union	Both	Age-standardized	Edentulism	Rate	1995	348.2	403.2	297.9
Incidence	European Union	Male	Age-standardized	Edentulism	Rate	1996	318.7	372.5	271.0
Incidence	European Union	Female	Age-standardized	Edentulism	Rate	1996	378.6	437.1	325.8
Incidence	European Union	Both	Age-standardized	Edentulism	Rate	1996	348.8	402.3	299.5
Incidence	European Union	Male	Age-standardized	Edentulism	Rate	1997	319.9	372.1	272.8
Incidence	European Union	Female	Age-standardized	Edentulism	Rate	1997	380.2	437.1	328.5
Incidence	European Union	Both	Age-standardized	Edentulism	Rate	1997	350.1	402.4	301.6
Incidence	European Union	Male	Age-standardized	Edentulism	Rate	1998	321.3	372.0	274.7
Incidence	European Union	Female	Age-standardized	Edentulism	Rate	1998	382.0	437.9	331.4
Incidence	European Union	Both	Age-standardized	Edentulism	Rate	1998	351.6	403.2	303.6
Incidence	European Union	Male	Age-standardized	Edentulism	Rate	1999	322.5	372.0	276.4
Incidence	European Union	Female	Age-standardized	Edentulism	Rate	1999	383.5	438.9	333.6
Incidence	European Union	Both	Age-standardized	Edentulism	Rate	1999	353.0	404.6	305.5
Incidence	European Union	Male	Age-standardized	Edentulism	Rate	2000	323.1	372.9	277.7

Incidence	European Union	Female	Age-standardized	Edentulism	Rate	2000	384.3	439.3	334.7
Incidence	European Union	Both	Age-standardized	Edentulism	Rate	2000	353.6	405.5	306.8
Incidence	European Union	Male	Age-standardized	Edentulism	Rate	2001	324.9	373.1	280.9
Incidence	European Union	Female	Age-standardized	Edentulism	Rate	2001	384.3	438.5	335.8
Incidence	European Union	Both	Age-standardized	Edentulism	Rate	2001	354.4	405.2	308.6
Incidence	European Union	Male	Age-standardized	Edentulism	Rate	2002	328.6	375.3	285.0
Incidence	European Union	Female	Age-standardized	Edentulism	Rate	2002	383.8	436.7	336.7
Incidence	European Union	Both	Age-standardized	Edentulism	Rate	2002	355.8	405.3	310.4
Incidence	European Union	Male	Age-standardized	Edentulism	Rate	2003	332.4	378.1	288.9
Incidence	European Union	Female	Age-standardized	Edentulism	Rate	2003	382.5	433.7	336.4
Incidence	European Union	Both	Age-standardized	Edentulism	Rate	2003	357.0	405.1	312.4
Incidence	European Union	Male	Age-standardized	Edentulism	Rate	2004	334.8	379.3	291.6
Incidence	European Union	Female	Age-standardized	Edentulism	Rate	2004	380.4	429.8	335.8
Incidence	European Union	Both	Age-standardized	Edentulism	Rate	2004	356.9	403.5	313.6
Incidence	European Union	Male	Age-standardized	Edentulism	Rate	2005	333.8	377.1	292.2
Incidence	European Union	Female	Age-standardized	Edentulism	Rate	2005	377.1	425.0	333.7
Incidence	European Union	Both	Age-standardized	Edentulism	Rate	2005	354.7	399.7	312.8
Incidence	European Union	Male	Age-standardized	Edentulism	Rate	2006	321.6	363.2	284.2
Incidence	European Union	Female	Age-standardized	Edentulism	Rate	2006	365.9	409.7	325.8
Incidence	European Union	Both	Age-standardized	Edentulism	Rate	2006	343.1	385.2	303.7
Incidence	European Union	Male	Age-standardized	Edentulism	Rate	2007	297.1	333.9	264.1
Incidence	European Union	Female	Age-standardized	Edentulism	Rate	2007	344.7	383.4	309.0
Incidence	European Union	Both	Age-standardized	Edentulism	Rate	2007	320.5	357.8	286.0
Incidence	European Union	Male	Age-standardized	Edentulism	Rate	2008	268.8	299.8	241.7
Incidence	European Union	Female	Age-standardized	Edentulism	Rate	2008	320.8	354.4	288.4
Incidence	European Union	Both	Age-standardized	Edentulism	Rate	2008	294.6	326.5	265.0
Incidence	European Union	Male	Age-standardized	Edentulism	Rate	2009	245.2	271.4	221.6
Incidence	European Union	Female	Age-standardized	Edentulism	Rate	2009	301.1	330.6	272.6
Incidence	European Union	Both	Age-standardized	Edentulism	Rate	2009	273.2	300.7	247.0
Incidence	European Union	Male	Age-standardized	Edentulism	Rate	2010	235.2	258.6	212.7
Incidence	European Union	Female	Age-standardized	Edentulism	Rate	2010	293.1	320.5	266.5
Incidence	European Union	Both	Age-standardized	Edentulism	Rate	2010	264.3	289.7	240.0
Incidence	European Union	Male	Age-standardized	Edentulism	Rate	2011	241.1	267.6	216.1
Incidence	European Union	Female	Age-standardized	Edentulism	Rate	2011	298.9	329.7	269.7
Incidence	European Union	Both	Age-standardized	Edentulism	Rate	2011	270.2	298.4	242.4
Incidence	European Union	Male	Age-standardized	Edentulism	Rate	2012	255.5	287.8	224.7
Incidence	European Union	Female	Age-standardized	Edentulism	Rate	2012	312.6	349.7	277.9
Incidence	European Union	Both	Age-standardized	Edentulism	Rate	2012	284.2	318.6	251.3
Incidence	European Union	Male	Age-standardized	Edentulism	Rate	2013	272.6	313.3	233.4
Incidence	European Union	Female	Age-standardized	Edentulism	Rate	2013	328.9	374.6	286.3
Incidence	European Union	Both	Age-standardized	Edentulism	Rate	2013	300.9	343.7	260.1
Incidence	European Union	Male	Age-standardized	Edentulism	Rate	2014	286.9	338.9	238.0
Incidence	European Union	Female	Age-standardized	Edentulism	Rate	2014	342.5	401.5	290.2
Incidence	European Union	Both	Age-standardized	Edentulism	Rate	2014	314.8	367.9	264.3

Incidence	European Union	Male	Age-standardized	Edentulism	Rate	2015	292.6	355.7	236.1
Incidence	European Union	Female	Age-standardized	Edentulism	Rate	2015	348.2	418.1	288.3
Incidence	European Union	Both	Age-standardized	Edentulism	Rate	2015	320.5	385.8	262.7
Incidence	European Union	Male	Age-standardized	Edentulism	Rate	2016	291.8	355.3	235.7
Incidence	European Union	Female	Age-standardized	Edentulism	Rate	2016	347.9	418.2	287.7
Incidence	European Union	Both	Age-standardized	Edentulism	Rate	2016	320.0	385.5	262.1
Incidence	European Union	Male	Age-standardized	Edentulism	Rate	2017	290.8	354.3	235.0
Incidence	European Union	Female	Age-standardized	Edentulism	Rate	2017	347.5	417.9	286.8
Incidence	European Union	Both	Age-standardized	Edentulism	Rate	2017	319.3	385.4	261.2
Incidence	European Union	Male	Age-standardized	Edentulism	Rate	2018	289.8	353.0	234.9
Incidence	European Union	Female	Age-standardized	Edentulism	Rate	2018	347.2	418.1	286.0
Incidence	European Union	Both	Age-standardized	Edentulism	Rate	2018	318.7	385.2	260.3
Incidence	European Union	Male	Age-standardized	Edentulism	Rate	2019	289.5	352.7	234.9
Incidence	European Union	Female	Age-standardized	Edentulism	Rate	2019	347.6	419.3	285.6
Incidence	European Union	Both	Age-standardized	Edentulism	Rate	2019	318.6	385.6	260.0
Incidence	European Union	Male	Age-standardized	Edentulism	Rate	2020	295.2	357.9	238.9
Incidence	European Union	Female	Age-standardized	Edentulism	Rate	2020	354.5	425.9	291.3
Incidence	European Union	Both	Age-standardized	Edentulism	Rate	2020	325.0	390.0	266.1
Incidence	European Union	Male	Age-standardized	Edentulism	Rate	2021	295.0	359.8	239.5
Incidence	European Union	Female	Age-standardized	Edentulism	Rate	2021	354.1	425.6	291.4
Incidence	European Union	Both	Age-standardized	Edentulism	Rate	2021	324.7	391.6	265.1
Incidence	European Union	Male	Age-standardized	Edentulism	Rate	2022	295.1	358.2	239.2
Incidence	European Union	Female	Age-standardized	Edentulism	Rate	2022	354.5	426.2	292.5
Incidence	European Union	Both	Age-standardized	Edentulism	Rate	2022	324.9	392.0	264.9
Incidence	European Union	Male	Age-standardized	Edentulism	Rate	2023	293.4	356.1	237.9
Incidence	European Union	Female	Age-standardized	Edentulism	Rate	2023	352.3	423.6	290.7
Incidence	European Union	Both	Age-standardized	Edentulism	Rate	2023	322.9	389.6	263.3
DALYs (Disability-Adjusted Life Years)	Russian Federation	Male	Age-standardized	Edentulism	Rate	1990	154.0	208.0	98.5
DALYs (Disability-Adjusted Life Years)	Russian Federation	Female	Age-standardized	Edentulism	Rate	1990	165.3	224.5	106.4
DALYs (Disability-Adjusted Life Years)	Russian Federation	Both	Age-standardized	Edentulism	Rate	1990	162.6	220.3	104.8
DALYs (Disability-Adjusted Life Years)	Federative Republic of Brazil	Male	Age-standardized	Edentulism	Rate	1990	200.2	280.6	128.9
DALYs (Disability-Adjusted Life Years)	Federative Republic of Brazil	Female	Age-standardized	Edentulism	Rate	1990	271.3	372.0	180.4
DALYs (Disability-Adjusted	Federative Republic of Brazil	Both	Age-standardized	Edentulism	Rate	1990	237.5	327.1	156.7

Life Years)									
DALYs (Disability-Adjusted Life Years)	Republic of South Africa	Male	Age-standardized	Edentulism	Rate	1990	200.4	285.8	123.0
DALYs (Disability-Adjusted Life Years)	Republic of South Africa	Female	Age-standardized	Edentulism	Rate	1990	211.4	305.5	131.4
DALYs (Disability-Adjusted Life Years)	Republic of South Africa	Both	Age-standardized	Edentulism	Rate	1990	206.1	296.6	127.6
DALYs (Disability-Adjusted Life Years)	Republic of Turkey	Male	Age-standardized	Edentulism	Rate	1990	225.5	306.8	143.6
DALYs (Disability-Adjusted Life Years)	Republic of Turkey	Female	Age-standardized	Edentulism	Rate	1990	197.2	271.4	127.9
DALYs (Disability-Adjusted Life Years)	Republic of Turkey	Both	Age-standardized	Edentulism	Rate	1990	210.3	287.8	135.1
DALYs (Disability-Adjusted Life Years)	People's Republic of China	Male	Age-standardized	Edentulism	Rate	1990	82.3	117.6	54.6
DALYs (Disability-Adjusted Life Years)	People's Republic of China	Female	Age-standardized	Edentulism	Rate	1990	102.2	145.1	66.4
DALYs (Disability-Adjusted Life Years)	People's Republic of China	Both	Age-standardized	Edentulism	Rate	1990	92.7	132.5	60.9
DALYs (Disability-Adjusted Life Years)	Federal Republic of Germany	Male	Age-standardized	Edentulism	Rate	1990	101.3	141.8	66.4
DALYs (Disability-Adjusted Life Years)	Federal Republic of Germany	Female	Age-standardized	Edentulism	Rate	1990	131.1	185.2	83.8
DALYs (Disability-Adjusted Life Years)	Federal Republic of Germany	Both	Age-standardized	Edentulism	Rate	1990	119.0	168.6	75.7
DALYs (Disability-Adjusted Life Years)	Kingdom of Saudi Arabia	Male	Age-standardized	Edentulism	Rate	1990	166.1	227.9	105.1
DALYs (Disability-Adjusted Life Years)	Kingdom of Saudi Arabia	Female	Age-standardized	Edentulism	Rate	1990	112.3	153.6	72.3
DALYs	Kingdom of Saudi	Both	Age-standardized	Edentulism	Rate	1990	143.7	196.4	91.9

(Disability-Adjusted Life Years)	Arabia								
DALYs (Disability-Adjusted Life Years)	Argentine Republic	Male	Age-standardized	Edentulism	Rate	1990	96.5	136.0	60.5
DALYs (Disability-Adjusted Life Years)	Argentine Republic	Female	Age-standardized	Edentulism	Rate	1990	155.5	220.4	96.6
DALYs (Disability-Adjusted Life Years)	Argentine Republic	Both	Age-standardized	Edentulism	Rate	1990	129.4	183.5	80.3
DALYs (Disability-Adjusted Life Years)	Republic of Korea	Male	Age-standardized	Edentulism	Rate	1991	58.1	82.4	38.0
DALYs (Disability-Adjusted Life Years)	Republic of Korea	Female	Age-standardized	Edentulism	Rate	1991	54.3	75.7	35.9
DALYs (Disability-Adjusted Life Years)	Republic of Korea	Both	Age-standardized	Edentulism	Rate	1991	57.0	80.0	37.8
DALYs (Disability-Adjusted Life Years)	Republic of Italy	Male	Age-standardized	Edentulism	Rate	1990	95.4	133.7	60.8
DALYs (Disability-Adjusted Life Years)	Republic of Italy	Female	Age-standardized	Edentulism	Rate	1990	122.1	172.3	77.2
DALYs (Disability-Adjusted Life Years)	Republic of Italy	Both	Age-standardized	Edentulism	Rate	1990	110.2	155.4	69.9
DALYs (Disability-Adjusted Life Years)	Kingdom of Saudi Arabia	Male	Age-standardized	Edentulism	Rate	1991	166.4	227.5	106.5
DALYs (Disability-Adjusted Life Years)	Kingdom of Saudi Arabia	Female	Age-standardized	Edentulism	Rate	1991	112.3	153.6	72.8
DALYs (Disability-Adjusted Life Years)	Kingdom of Saudi Arabia	Both	Age-standardized	Edentulism	Rate	1991	143.8	195.1	92.7
DALYs (Disability-Adjusted Life Years)	Republic of Korea	Male	Age-standardized	Edentulism	Rate	1990	58.7	82.1	38.1
DALYs (Disability-Adjusted Life Years)	Republic of Korea	Female	Age-standardized	Edentulism	Rate	1990	55.2	78.0	37.1

DALYs (Disability-Adjusted Life Years)	Republic of Korea	Both	Age-standardized	Edentulism	Rate	1990	57.8	81.2	38.7
DALYs (Disability-Adjusted Life Years)	Federal Republic of Germany	Male	Age-standardized	Edentulism	Rate	1991	102.3	140.8	68.7
DALYs (Disability-Adjusted Life Years)	Federal Republic of Germany	Female	Age-standardized	Edentulism	Rate	1991	131.1	183.1	86.5
DALYs (Disability-Adjusted Life Years)	Federal Republic of Germany	Both	Age-standardized	Edentulism	Rate	1991	119.3	166.5	79.1
DALYs (Disability-Adjusted Life Years)	Republic of India	Male	Age-standardized	Edentulism	Rate	1991	96.1	132.6	61.8
DALYs (Disability-Adjusted Life Years)	Republic of India	Female	Age-standardized	Edentulism	Rate	1991	101.6	140.6	65.3
DALYs (Disability-Adjusted Life Years)	Republic of India	Both	Age-standardized	Edentulism	Rate	1991	98.8	136.5	63.5
DALYs (Disability-Adjusted Life Years)	Republic of South Africa	Male	Age-standardized	Edentulism	Rate	1991	192.9	275.5	118.5
DALYs (Disability-Adjusted Life Years)	Republic of South Africa	Female	Age-standardized	Edentulism	Rate	1991	198.8	288.0	123.4
DALYs (Disability-Adjusted Life Years)	Republic of South Africa	Both	Age-standardized	Edentulism	Rate	1991	195.9	281.9	121.0
DALYs (Disability-Adjusted Life Years)	Republic of India	Male	Age-standardized	Edentulism	Rate	1990	96.2	133.3	61.8
DALYs (Disability-Adjusted Life Years)	Republic of India	Female	Age-standardized	Edentulism	Rate	1990	101.7	139.9	65.2
DALYs (Disability-Adjusted Life Years)	Republic of India	Both	Age-standardized	Edentulism	Rate	1990	98.9	136.5	63.5
DALYs (Disability-Adjusted Life Years)	Republic of South Africa	Male	Age-standardized	Edentulism	Rate	1992	186.4	265.6	114.0
DALYs (Disability-Adjusted	Republic of South Africa	Female	Age-standardized	Edentulism	Rate	1992	188.7	271.1	117.9

Life Years)									
DALYs (Disability-Adjusted Life Years)	Republic of South Africa	Both	Age-standardized	Edentulism	Rate	1992	187.5	269.1	116.0
DALYs (Disability-Adjusted Life Years)	Federative Republic of Brazil	Male	Age-standardized	Edentulism	Rate	1991	200.1	280.7	128.4
DALYs (Disability-Adjusted Life Years)	Federative Republic of Brazil	Female	Age-standardized	Edentulism	Rate	1991	271.4	371.2	180.8
DALYs (Disability-Adjusted Life Years)	Federative Republic of Brazil	Both	Age-standardized	Edentulism	Rate	1991	237.6	327.0	156.7
DALYs (Disability-Adjusted Life Years)	Republic of Korea	Male	Age-standardized	Edentulism	Rate	1993	56.9	79.8	36.9
DALYs (Disability-Adjusted Life Years)	Republic of Korea	Female	Age-standardized	Edentulism	Rate	1993	53.0	74.1	35.1
DALYs (Disability-Adjusted Life Years)	Republic of Korea	Both	Age-standardized	Edentulism	Rate	1993	55.7	77.9	36.9
DALYs (Disability-Adjusted Life Years)	Federative Republic of Brazil	Male	Age-standardized	Edentulism	Rate	1992	199.9	280.7	128.7
DALYs (Disability-Adjusted Life Years)	Federative Republic of Brazil	Female	Age-standardized	Edentulism	Rate	1992	271.3	371.5	180.0
DALYs (Disability-Adjusted Life Years)	Federative Republic of Brazil	Both	Age-standardized	Edentulism	Rate	1992	237.5	327.8	156.4
DALYs (Disability-Adjusted Life Years)	Japan	Male	Age-standardized	Edentulism	Rate	1990	94.7	135.3	61.6
DALYs (Disability-Adjusted Life Years)	Japan	Female	Age-standardized	Edentulism	Rate	1990	78.2	110.7	52.0
DALYs (Disability-Adjusted Life Years)	Japan	Both	Age-standardized	Edentulism	Rate	1990	86.2	122.6	58.3
DALYs (Disability-Adjusted Life Years)	Federal Republic of Germany	Male	Age-standardized	Edentulism	Rate	1992	103.1	141.5	70.0
DALYs	Federal Republic	Female	Age-standardized	Edentulism	Rate	1992	131.2	182.0	88.6

(Disability-Adjusted Life Years)	of Germany								
DALYs (Disability-Adjusted Life Years)	Federal Republic of Germany	Both	Age-standardized	Edentulism	Rate	1992	119.7	164.9	80.8
DALYs (Disability-Adjusted Life Years)	United States of America	Male	Age-standardized	Edentulism	Rate	1990	145.4	202.0	90.9
DALYs (Disability-Adjusted Life Years)	United States of America	Female	Age-standardized	Edentulism	Rate	1990	151.2	207.5	95.8
DALYs (Disability-Adjusted Life Years)	United States of America	Both	Age-standardized	Edentulism	Rate	1990	148.3	204.6	93.4
DALYs (Disability-Adjusted Life Years)	Republic of Indonesia	Male	Age-standardized	Edentulism	Rate	1991	88.5	123.6	57.2
DALYs (Disability-Adjusted Life Years)	Republic of Indonesia	Female	Age-standardized	Edentulism	Rate	1991	141.9	194.9	90.0
DALYs (Disability-Adjusted Life Years)	Republic of Indonesia	Both	Age-standardized	Edentulism	Rate	1991	116.2	160.1	74.5
DALYs (Disability-Adjusted Life Years)	Republic of Korea	Male	Age-standardized	Edentulism	Rate	1992	57.5	81.6	36.7
DALYs (Disability-Adjusted Life Years)	Republic of Korea	Female	Age-standardized	Edentulism	Rate	1992	53.6	75.2	35.1
DALYs (Disability-Adjusted Life Years)	Republic of Korea	Both	Age-standardized	Edentulism	Rate	1992	56.4	79.3	37.1
DALYs (Disability-Adjusted Life Years)	Australia	Male	Age-standardized	Edentulism	Rate	1990	201.4	275.9	134.0
DALYs (Disability-Adjusted Life Years)	Australia	Female	Age-standardized	Edentulism	Rate	1990	310.7	433.2	208.4
DALYs (Disability-Adjusted Life Years)	Australia	Both	Age-standardized	Edentulism	Rate	1990	258.8	358.7	173.3
DALYs (Disability-Adjusted Life Years)	French Republic	Male	Age-standardized	Edentulism	Rate	1991	81.1	114.5	52.5

DALYs (Disability-Adjusted Life Years)	French Republic	Female	Age-standardized	Edentulism	Rate	1991	113.8	160.6	72.3
DALYs (Disability-Adjusted Life Years)	French Republic	Both	Age-standardized	Edentulism	Rate	1991	99.5	141.4	64.3
DALYs (Disability-Adjusted Life Years)	French Republic	Male	Age-standardized	Edentulism	Rate	1990	88.1	125.2	56.5
DALYs (Disability-Adjusted Life Years)	French Republic	Female	Age-standardized	Edentulism	Rate	1990	124.0	174.7	77.5
DALYs (Disability-Adjusted Life Years)	French Republic	Both	Age-standardized	Edentulism	Rate	1990	108.3	153.3	68.8
DALYs (Disability-Adjusted Life Years)	Kingdom of Saudi Arabia	Male	Age-standardized	Edentulism	Rate	1992	166.7	229.5	105.4
DALYs (Disability-Adjusted Life Years)	Kingdom of Saudi Arabia	Female	Age-standardized	Edentulism	Rate	1992	112.5	153.5	72.9
DALYs (Disability-Adjusted Life Years)	Kingdom of Saudi Arabia	Both	Age-standardized	Edentulism	Rate	1992	144.0	197.8	91.9
DALYs (Disability-Adjusted Life Years)	Federal Republic of Germany	Male	Age-standardized	Edentulism	Rate	1993	104.0	143.7	71.4
DALYs (Disability-Adjusted Life Years)	Federal Republic of Germany	Female	Age-standardized	Edentulism	Rate	1993	131.4	182.7	88.7
DALYs (Disability-Adjusted Life Years)	Federal Republic of Germany	Both	Age-standardized	Edentulism	Rate	1993	120.1	166.4	81.7
DALYs (Disability-Adjusted Life Years)	Canada	Male	Age-standardized	Edentulism	Rate	1990	73.9	101.7	45.8
DALYs (Disability-Adjusted Life Years)	Canada	Female	Age-standardized	Edentulism	Rate	1990	93.9	129.5	59.1
DALYs (Disability-Adjusted Life Years)	Canada	Both	Age-standardized	Edentulism	Rate	1990	84.6	116.5	52.7
DALYs (Disability-Adjusted	Republic of Indonesia	Male	Age-standardized	Edentulism	Rate	1990	88.9	124.4	57.3

Life Years)									
DALYs (Disability-Adjusted Life Years)	Republic of Indonesia	Female	Age-standardized	Edentulism	Rate	1990	142.6	195.7	90.4
DALYs (Disability-Adjusted Life Years)	Republic of Indonesia	Both	Age-standardized	Edentulism	Rate	1990	116.8	160.9	74.9
DALYs (Disability-Adjusted Life Years)	United Kingdom of Great Britain and Northern Ireland	Male	Age-standardized	Edentulism	Rate	1991	105.2	148.1	67.2
DALYs (Disability-Adjusted Life Years)	United Kingdom of Great Britain and Northern Ireland	Female	Age-standardized	Edentulism	Rate	1991	131.1	185.3	82.6
DALYs (Disability-Adjusted Life Years)	United Kingdom of Great Britain and Northern Ireland	Both	Age-standardized	Edentulism	Rate	1991	119.3	168.9	75.4
DALYs (Disability-Adjusted Life Years)	Kingdom of Saudi Arabia	Male	Age-standardized	Edentulism	Rate	1993	166.6	228.0	105.9
DALYs (Disability-Adjusted Life Years)	Kingdom of Saudi Arabia	Female	Age-standardized	Edentulism	Rate	1993	112.4	153.0	71.4
DALYs (Disability-Adjusted Life Years)	Kingdom of Saudi Arabia	Both	Age-standardized	Edentulism	Rate	1993	143.9	196.7	91.5
DALYs (Disability-Adjusted Life Years)	Australia	Male	Age-standardized	Edentulism	Rate	1991	170.9	235.9	112.7
DALYs (Disability-Adjusted Life Years)	Australia	Female	Age-standardized	Edentulism	Rate	1991	256.0	355.9	171.3
DALYs (Disability-Adjusted Life Years)	Australia	Both	Age-standardized	Edentulism	Rate	1991	215.8	300.3	143.6
DALYs (Disability-Adjusted Life Years)	United Mexican States	Male	Age-standardized	Edentulism	Rate	1990	119.1	161.8	75.5
DALYs (Disability-Adjusted Life Years)	United Mexican States	Female	Age-standardized	Edentulism	Rate	1990	205.3	276.7	133.2
DALYs (Disability-Adjusted Life Years)	United Mexican States	Both	Age-standardized	Edentulism	Rate	1990	164.1	222.8	105.6
DALYs	Republic of India	Male	Age-standardized	Edentulism	Rate	1992	95.8	132.6	61.9

(Disability-Adjusted Life Years)									
DALYs (Disability-Adjusted Life Years)	Republic of India	Female	Age-standardized	Edentulism	Rate	1992	101.5	140.1	65.2
DALYs (Disability-Adjusted Life Years)	Republic of India	Both	Age-standardized	Edentulism	Rate	1992	98.5	136.4	63.5
DALYs (Disability-Adjusted Life Years)	European Union	Male	Age-standardized	Edentulism	Rate	1990	116.8	161.3	74.7
DALYs (Disability-Adjusted Life Years)	European Union	Female	Age-standardized	Edentulism	Rate	1990	149.5	208.8	95.5
DALYs (Disability-Adjusted Life Years)	European Union	Both	Age-standardized	Edentulism	Rate	1990	135.3	189.2	86.2
DALYs (Disability-Adjusted Life Years)	United Mexican States	Male	Age-standardized	Edentulism	Rate	1991	119.1	162.1	76.1
DALYs (Disability-Adjusted Life Years)	United Mexican States	Female	Age-standardized	Edentulism	Rate	1991	205.8	277.9	132.9
DALYs (Disability-Adjusted Life Years)	United Mexican States	Both	Age-standardized	Edentulism	Rate	1991	164.3	222.7	105.8
DALYs (Disability-Adjusted Life Years)	Russian Federation	Male	Age-standardized	Edentulism	Rate	1991	154.3	208.2	98.9
DALYs (Disability-Adjusted Life Years)	Russian Federation	Female	Age-standardized	Edentulism	Rate	1991	165.8	225.2	106.9
DALYs (Disability-Adjusted Life Years)	Russian Federation	Both	Age-standardized	Edentulism	Rate	1991	163.0	221.0	105.2
DALYs (Disability-Adjusted Life Years)	Republic of India	Male	Age-standardized	Edentulism	Rate	1993	95.4	132.1	61.5
DALYs (Disability-Adjusted Life Years)	Republic of India	Female	Age-standardized	Edentulism	Rate	1993	101.2	139.7	65.1
DALYs (Disability-Adjusted Life Years)	Republic of India	Both	Age-standardized	Edentulism	Rate	1993	98.2	135.9	63.3

DALYs (Disability-Adjusted Life Years)	Federative Republic of Brazil	Male	Age-standardized	Edentulism	Rate	1993	199.7	280.6	128.7
DALYs (Disability-Adjusted Life Years)	Federative Republic of Brazil	Female	Age-standardized	Edentulism	Rate	1993	271.1	373.2	179.0
DALYs (Disability-Adjusted Life Years)	Federative Republic of Brazil	Both	Age-standardized	Edentulism	Rate	1993	237.4	328.4	155.9
DALYs (Disability-Adjusted Life Years)	United Kingdom of Great Britain and Northern Ireland	Male	Age-standardized	Edentulism	Rate	1990	105.6	148.5	67.4
DALYs (Disability-Adjusted Life Years)	United Kingdom of Great Britain and Northern Ireland	Female	Age-standardized	Edentulism	Rate	1990	131.5	185.5	83.2
DALYs (Disability-Adjusted Life Years)	United Kingdom of Great Britain and Northern Ireland	Both	Age-standardized	Edentulism	Rate	1990	119.7	169.1	75.9
DALYs (Disability-Adjusted Life Years)	Argentine Republic	Male	Age-standardized	Edentulism	Rate	1991	95.7	135.7	60.1
DALYs (Disability-Adjusted Life Years)	Argentine Republic	Female	Age-standardized	Edentulism	Rate	1991	154.6	218.8	95.3
DALYs (Disability-Adjusted Life Years)	Argentine Republic	Both	Age-standardized	Edentulism	Rate	1991	128.6	183.6	79.7
DALYs (Disability-Adjusted Life Years)	Republic of Korea	Male	Age-standardized	Edentulism	Rate	1995	56.0	80.0	36.9
DALYs (Disability-Adjusted Life Years)	Republic of Korea	Female	Age-standardized	Edentulism	Rate	1995	51.9	72.8	34.0
DALYs (Disability-Adjusted Life Years)	Republic of Korea	Both	Age-standardized	Edentulism	Rate	1995	54.7	77.0	36.1
DALYs (Disability-Adjusted Life Years)	Republic of Turkey	Male	Age-standardized	Edentulism	Rate	1991	225.2	306.7	142.8
DALYs (Disability-Adjusted Life Years)	Republic of Turkey	Female	Age-standardized	Edentulism	Rate	1991	196.6	268.7	127.6
DALYs (Disability-Adjusted	Republic of Turkey	Both	Age-standardized	Edentulism	Rate	1991	209.8	286.3	134.9

Life Years)									
DALYs (Disability-Adjusted Life Years)	Republic of South Africa	Male	Age-standardized	Edentulism	Rate	1993	181.1	257.7	111.2
DALYs (Disability-Adjusted Life Years)	Republic of South Africa	Female	Age-standardized	Edentulism	Rate	1993	181.1	259.7	112.8
DALYs (Disability-Adjusted Life Years)	Republic of South Africa	Both	Age-standardized	Edentulism	Rate	1993	181.0	258.8	112.1
DALYs (Disability-Adjusted Life Years)	Federative Republic of Brazil	Male	Age-standardized	Edentulism	Rate	1995	199.4	279.3	128.6
DALYs (Disability-Adjusted Life Years)	Federative Republic of Brazil	Female	Age-standardized	Edentulism	Rate	1995	270.4	370.6	179.1
DALYs (Disability-Adjusted Life Years)	Federative Republic of Brazil	Both	Age-standardized	Edentulism	Rate	1995	236.9	327.5	155.9
DALYs (Disability-Adjusted Life Years)	Republic of South Africa	Male	Age-standardized	Edentulism	Rate	1995	175.0	248.7	106.4
DALYs (Disability-Adjusted Life Years)	Republic of South Africa	Female	Age-standardized	Edentulism	Rate	1995	173.6	247.6	106.7
DALYs (Disability-Adjusted Life Years)	Republic of South Africa	Both	Age-standardized	Edentulism	Rate	1995	174.2	248.2	106.5
DALYs (Disability-Adjusted Life Years)	Federal Republic of Germany	Male	Age-standardized	Edentulism	Rate	1995	105.2	146.3	71.7
DALYs (Disability-Adjusted Life Years)	Federal Republic of Germany	Female	Age-standardized	Edentulism	Rate	1995	131.8	183.3	88.8
DALYs (Disability-Adjusted Life Years)	Federal Republic of Germany	Both	Age-standardized	Edentulism	Rate	1995	120.7	167.5	81.5
DALYs (Disability-Adjusted Life Years)	European Union	Male	Age-standardized	Edentulism	Rate	1991	115.4	159.0	74.6
DALYs (Disability-Adjusted Life Years)	European Union	Female	Age-standardized	Edentulism	Rate	1991	147.2	204.6	95.3
DALYs	European Union	Both	Age-standardized	Edentulism	Rate	1991	133.4	185.4	86.1

(Disability-Adjusted Life Years)									
DALYs (Disability-Adjusted Life Years)	Kingdom of Saudi Arabia	Male	Age-standardized	Edentulism	Rate	1995	166.8	231.6	104.4
DALYs (Disability-Adjusted Life Years)	Kingdom of Saudi Arabia	Female	Age-standardized	Edentulism	Rate	1995	112.3	153.6	72.2
DALYs (Disability-Adjusted Life Years)	Kingdom of Saudi Arabia	Both	Age-standardized	Edentulism	Rate	1995	143.8	196.6	91.4
DALYs (Disability-Adjusted Life Years)	Russian Federation	Male	Age-standardized	Edentulism	Rate	1992	154.7	208.7	99.3
DALYs (Disability-Adjusted Life Years)	Russian Federation	Female	Age-standardized	Edentulism	Rate	1992	166.3	225.5	107.2
DALYs (Disability-Adjusted Life Years)	Russian Federation	Both	Age-standardized	Edentulism	Rate	1992	163.4	221.0	105.5
DALYs (Disability-Adjusted Life Years)	Republic of Italy	Male	Age-standardized	Edentulism	Rate	1992	94.9	132.4	60.5
DALYs (Disability-Adjusted Life Years)	Republic of Italy	Female	Age-standardized	Edentulism	Rate	1992	121.5	171.7	77.1
DALYs (Disability-Adjusted Life Years)	Republic of Italy	Both	Age-standardized	Edentulism	Rate	1992	109.6	154.3	69.6
DALYs (Disability-Adjusted Life Years)	Republic of Italy	Male	Age-standardized	Edentulism	Rate	1991	95.2	133.1	60.6
DALYs (Disability-Adjusted Life Years)	Republic of Italy	Female	Age-standardized	Edentulism	Rate	1991	121.8	172.7	77.4
DALYs (Disability-Adjusted Life Years)	Republic of Italy	Both	Age-standardized	Edentulism	Rate	1991	109.9	155.0	69.8
DALYs (Disability-Adjusted Life Years)	Argentine Republic	Male	Age-standardized	Edentulism	Rate	1992	95.1	134.7	59.8
DALYs (Disability-Adjusted Life Years)	Argentine Republic	Female	Age-standardized	Edentulism	Rate	1992	153.8	218.2	94.7

DALYs (Disability-Adjusted Life Years)	Argentine Republic	Both	Age-standardized	Edentulism	Rate	1992	127.9	182.2	79.2
DALYs (Disability-Adjusted Life Years)	Kingdom of Saudi Arabia	Male	Age-standardized	Edentulism	Rate	1996	166.8	229.5	105.0
DALYs (Disability-Adjusted Life Years)	Kingdom of Saudi Arabia	Female	Age-standardized	Edentulism	Rate	1996	112.4	152.7	72.1
DALYs (Disability-Adjusted Life Years)	Kingdom of Saudi Arabia	Both	Age-standardized	Edentulism	Rate	1996	143.7	196.6	91.6
DALYs (Disability-Adjusted Life Years)	Canada	Male	Age-standardized	Edentulism	Rate	1991	74.4	101.4	46.3
DALYs (Disability-Adjusted Life Years)	Canada	Female	Age-standardized	Edentulism	Rate	1991	95.0	131.5	60.0
DALYs (Disability-Adjusted Life Years)	Canada	Both	Age-standardized	Edentulism	Rate	1991	85.5	117.0	53.7
DALYs (Disability-Adjusted Life Years)	Republic of Korea	Male	Age-standardized	Edentulism	Rate	1996	55.5	78.9	36.2
DALYs (Disability-Adjusted Life Years)	Republic of Korea	Female	Age-standardized	Edentulism	Rate	1996	51.6	72.2	33.9
DALYs (Disability-Adjusted Life Years)	Republic of Korea	Both	Age-standardized	Edentulism	Rate	1996	54.4	76.4	35.7
DALYs (Disability-Adjusted Life Years)	People's Republic of China	Male	Age-standardized	Edentulism	Rate	1991	78.5	112.1	51.7
DALYs (Disability-Adjusted Life Years)	People's Republic of China	Female	Age-standardized	Edentulism	Rate	1991	100.1	143.0	64.7
DALYs (Disability-Adjusted Life Years)	People's Republic of China	Both	Age-standardized	Edentulism	Rate	1991	89.8	128.2	58.7
DALYs (Disability-Adjusted Life Years)	Federal Republic of Germany	Male	Age-standardized	Edentulism	Rate	1996	105.7	148.4	71.3
DALYs (Disability-Adjusted	Federal Republic of Germany	Female	Age-standardized	Edentulism	Rate	1996	132.3	184.8	89.6

Life Years)									
DALYs (Disability-Adjusted Life Years)	Federal Republic of Germany	Both	Age-standardized	Edentulism	Rate	1996	121.2	168.7	82.2
DALYs (Disability-Adjusted Life Years)	People's Republic of China	Male	Age-standardized	Edentulism	Rate	1992	75.0	106.8	49.2
DALYs (Disability-Adjusted Life Years)	People's Republic of China	Female	Age-standardized	Edentulism	Rate	1992	97.5	139.4	63.3
DALYs (Disability-Adjusted Life Years)	People's Republic of China	Both	Age-standardized	Edentulism	Rate	1992	86.8	123.7	56.6
DALYs (Disability-Adjusted Life Years)	Japan	Male	Age-standardized	Edentulism	Rate	1991	88.1	125.2	57.2
DALYs (Disability-Adjusted Life Years)	Japan	Female	Age-standardized	Edentulism	Rate	1991	72.2	102.3	47.9
DALYs (Disability-Adjusted Life Years)	Japan	Both	Age-standardized	Edentulism	Rate	1991	79.9	113.7	53.8
DALYs (Disability-Adjusted Life Years)	Republic of India	Male	Age-standardized	Edentulism	Rate	1995	94.2	130.2	61.0
DALYs (Disability-Adjusted Life Years)	Republic of India	Female	Age-standardized	Edentulism	Rate	1995	100.4	138.5	64.5
DALYs (Disability-Adjusted Life Years)	Republic of India	Both	Age-standardized	Edentulism	Rate	1995	97.2	134.4	62.7
DALYs (Disability-Adjusted Life Years)	United States of America	Male	Age-standardized	Edentulism	Rate	1991	142.6	195.6	89.8
DALYs (Disability-Adjusted Life Years)	United States of America	Female	Age-standardized	Edentulism	Rate	1991	149.3	205.1	95.0
DALYs (Disability-Adjusted Life Years)	United States of America	Both	Age-standardized	Edentulism	Rate	1991	146.2	199.4	92.6
DALYs (Disability-Adjusted Life Years)	Federative Republic of Brazil	Male	Age-standardized	Edentulism	Rate	1996	198.8	279.0	128.2
DALYs	Federative	Female	Age-standardized	Edentulism	Rate	1996	268.7	369.4	177.3

(Disability-Adjusted Life Years)	Republic of Brazil								
DALYs (Disability-Adjusted Life Years)	Federative Republic of Brazil	Both	Age-standardized	Edentulism	Rate	1996	235.8	326.5	154.8
DALYs (Disability-Adjusted Life Years)	Republic of South Africa	Male	Age-standardized	Edentulism	Rate	1996	174.4	247.2	106.2
DALYs (Disability-Adjusted Life Years)	Republic of South Africa	Female	Age-standardized	Edentulism	Rate	1996	175.7	249.1	107.9
DALYs (Disability-Adjusted Life Years)	Republic of South Africa	Both	Age-standardized	Edentulism	Rate	1996	175.1	248.3	107.2
DALYs (Disability-Adjusted Life Years)	Republic of India	Male	Age-standardized	Edentulism	Rate	1996	91.8	127.1	59.1
DALYs (Disability-Adjusted Life Years)	Republic of India	Female	Age-standardized	Edentulism	Rate	1996	97.6	134.3	62.6
DALYs (Disability-Adjusted Life Years)	Republic of India	Both	Age-standardized	Edentulism	Rate	1996	94.6	130.6	60.8
DALYs (Disability-Adjusted Life Years)	Canada	Male	Age-standardized	Edentulism	Rate	1992	74.7	101.5	46.9
DALYs (Disability-Adjusted Life Years)	Canada	Female	Age-standardized	Edentulism	Rate	1992	96.0	132.5	60.7
DALYs (Disability-Adjusted Life Years)	Canada	Both	Age-standardized	Edentulism	Rate	1992	86.2	117.7	54.3
DALYs (Disability-Adjusted Life Years)	Japan	Male	Age-standardized	Edentulism	Rate	1992	83.8	118.6	54.4
DALYs (Disability-Adjusted Life Years)	Japan	Female	Age-standardized	Edentulism	Rate	1992	66.8	94.3	44.4
DALYs (Disability-Adjusted Life Years)	Japan	Both	Age-standardized	Edentulism	Rate	1992	75.0	106.1	50.3
DALYs (Disability-Adjusted Life Years)	Federative Republic of Brazil	Male	Age-standardized	Edentulism	Rate	1994	199.6	279.6	129.0

DALYs (Disability-Adjusted Life Years)	Federative Republic of Brazil	Female	Age-standardized	Edentulism	Rate	1994	270.8	371.8	178.7
DALYs (Disability-Adjusted Life Years)	Federative Republic of Brazil	Both	Age-standardized	Edentulism	Rate	1994	237.2	327.7	156.0
DALYs (Disability-Adjusted Life Years)	Republic of South Africa	Male	Age-standardized	Edentulism	Rate	1994	177.3	251.3	108.3
DALYs (Disability-Adjusted Life Years)	Republic of South Africa	Female	Age-standardized	Edentulism	Rate	1994	176.0	250.5	108.4
DALYs (Disability-Adjusted Life Years)	Republic of South Africa	Both	Age-standardized	Edentulism	Rate	1994	176.5	251.3	108.4
DALYs (Disability-Adjusted Life Years)	United States of America	Male	Age-standardized	Edentulism	Rate	1992	140.0	190.8	88.6
DALYs (Disability-Adjusted Life Years)	United States of America	Female	Age-standardized	Edentulism	Rate	1992	147.5	201.9	94.4
DALYs (Disability-Adjusted Life Years)	United States of America	Both	Age-standardized	Edentulism	Rate	1992	144.2	197.8	91.8
DALYs (Disability-Adjusted Life Years)	Republic of Korea	Male	Age-standardized	Edentulism	Rate	1994	56.4	80.1	36.1
DALYs (Disability-Adjusted Life Years)	Republic of Korea	Female	Age-standardized	Edentulism	Rate	1994	52.4	73.8	34.7
DALYs (Disability-Adjusted Life Years)	Republic of Korea	Both	Age-standardized	Edentulism	Rate	1994	55.2	78.0	36.3
DALYs (Disability-Adjusted Life Years)	Federal Republic of Germany	Male	Age-standardized	Edentulism	Rate	1994	104.6	144.5	71.4
DALYs (Disability-Adjusted Life Years)	Federal Republic of Germany	Female	Age-standardized	Edentulism	Rate	1994	131.5	181.4	88.0
DALYs (Disability-Adjusted Life Years)	Federal Republic of Germany	Both	Age-standardized	Edentulism	Rate	1994	120.3	166.0	81.0
DALYs (Disability-Adjusted	Kingdom of Saudi Arabia	Male	Age-standardized	Edentulism	Rate	1994	166.9	230.6	105.2

Life Years)									
DALYs (Disability-Adjusted Life Years)	Kingdom of Saudi Arabia	Female	Age-standardized	Edentulism	Rate	1994	112.4	151.2	71.5
DALYs (Disability-Adjusted Life Years)	Kingdom of Saudi Arabia	Both	Age-standardized	Edentulism	Rate	1994	143.9	196.9	91.3
DALYs (Disability-Adjusted Life Years)	Republic of Indonesia	Male	Age-standardized	Edentulism	Rate	1992	88.1	122.9	57.0
DALYs (Disability-Adjusted Life Years)	Republic of Indonesia	Female	Age-standardized	Edentulism	Rate	1992	141.4	193.7	90.2
DALYs (Disability-Adjusted Life Years)	Republic of Indonesia	Both	Age-standardized	Edentulism	Rate	1992	115.8	159.4	74.5
DALYs (Disability-Adjusted Life Years)	Republic of India	Male	Age-standardized	Edentulism	Rate	1994	94.9	131.2	61.2
DALYs (Disability-Adjusted Life Years)	Republic of India	Female	Age-standardized	Edentulism	Rate	1994	100.8	139.1	64.8
DALYs (Disability-Adjusted Life Years)	Republic of India	Both	Age-standardized	Edentulism	Rate	1994	97.8	135.3	63.0
DALYs (Disability-Adjusted Life Years)	French Republic	Male	Age-standardized	Edentulism	Rate	1992	75.5	105.3	48.8
DALYs (Disability-Adjusted Life Years)	French Republic	Female	Age-standardized	Edentulism	Rate	1992	105.4	150.3	66.2
DALYs (Disability-Adjusted Life Years)	French Republic	Both	Age-standardized	Edentulism	Rate	1992	92.4	130.6	58.9
DALYs (Disability-Adjusted Life Years)	Australia	Male	Age-standardized	Edentulism	Rate	1992	144.2	199.7	94.9
DALYs (Disability-Adjusted Life Years)	Australia	Female	Age-standardized	Edentulism	Rate	1992	208.2	287.5	139.2
DALYs (Disability-Adjusted Life Years)	Australia	Both	Age-standardized	Edentulism	Rate	1992	178.2	247.1	118.3
DALYs	United Kingdom of	Male	Age-standardized	Edentulism	Rate	1992	104.9	147.3	66.8

(Disability-Adjusted Life Years)	Great Britain and Northern Ireland								
DALYs (Disability-Adjusted Life Years)	United Kingdom of Great Britain and Northern Ireland	Female	Age-standardized	Edentulism	Rate	1992	130.8	185.0	82.4
DALYs (Disability-Adjusted Life Years)	United Kingdom of Great Britain and Northern Ireland	Both	Age-standardized	Edentulism	Rate	1992	119.0	168.0	75.1
DALYs (Disability-Adjusted Life Years)	United Mexican States	Male	Age-standardized	Edentulism	Rate	1992	119.2	162.2	76.2
DALYs (Disability-Adjusted Life Years)	United Mexican States	Female	Age-standardized	Edentulism	Rate	1992	206.0	278.2	132.7
DALYs (Disability-Adjusted Life Years)	United Mexican States	Both	Age-standardized	Edentulism	Rate	1992	164.5	223.5	105.8
DALYs (Disability-Adjusted Life Years)	European Union	Male	Age-standardized	Edentulism	Rate	1992	114.1	157.7	74.2
DALYs (Disability-Adjusted Life Years)	European Union	Female	Age-standardized	Edentulism	Rate	1992	145.3	201.3	94.9
DALYs (Disability-Adjusted Life Years)	European Union	Both	Age-standardized	Edentulism	Rate	1992	131.7	182.8	85.8
DALYs (Disability-Adjusted Life Years)	Republic of Korea	Male	Age-standardized	Edentulism	Rate	1997	55.2	78.1	35.9
DALYs (Disability-Adjusted Life Years)	Republic of Korea	Female	Age-standardized	Edentulism	Rate	1997	51.4	72.1	34.4
DALYs (Disability-Adjusted Life Years)	Republic of Korea	Both	Age-standardized	Edentulism	Rate	1997	54.1	75.9	36.4
DALYs (Disability-Adjusted Life Years)	Federal Republic of Germany	Male	Age-standardized	Edentulism	Rate	1997	106.4	147.7	72.2
DALYs (Disability-Adjusted Life Years)	Federal Republic of Germany	Female	Age-standardized	Edentulism	Rate	1997	133.0	187.2	90.5
DALYs (Disability-Adjusted Life Years)	Federal Republic of Germany	Both	Age-standardized	Edentulism	Rate	1997	121.8	170.1	82.8

DALYs (Disability-Adjusted Life Years)	Republic of Turkey	Male	Age-standardized	Edentulism	Rate	1992	224.8	306.4	142.4
DALYs (Disability-Adjusted Life Years)	Republic of Turkey	Female	Age-standardized	Edentulism	Rate	1992	196.8	269.3	127.1
DALYs (Disability-Adjusted Life Years)	Republic of Turkey	Both	Age-standardized	Edentulism	Rate	1992	209.6	286.3	134.3
DALYs (Disability-Adjusted Life Years)	Kingdom of Saudi Arabia	Male	Age-standardized	Edentulism	Rate	1997	166.7	229.0	105.4
DALYs (Disability-Adjusted Life Years)	Kingdom of Saudi Arabia	Female	Age-standardized	Edentulism	Rate	1997	112.5	153.2	72.6
DALYs (Disability-Adjusted Life Years)	Kingdom of Saudi Arabia	Both	Age-standardized	Edentulism	Rate	1997	143.5	196.3	91.0
DALYs (Disability-Adjusted Life Years)	Republic of India	Male	Age-standardized	Edentulism	Rate	1997	86.7	120.4	55.8
DALYs (Disability-Adjusted Life Years)	Republic of India	Female	Age-standardized	Edentulism	Rate	1997	91.8	126.1	58.9
DALYs (Disability-Adjusted Life Years)	Republic of India	Both	Age-standardized	Edentulism	Rate	1997	89.2	123.0	57.3
DALYs (Disability-Adjusted Life Years)	Federative Republic of Brazil	Male	Age-standardized	Edentulism	Rate	1997	197.5	277.1	126.6
DALYs (Disability-Adjusted Life Years)	Federative Republic of Brazil	Female	Age-standardized	Edentulism	Rate	1997	265.4	364.2	174.5
DALYs (Disability-Adjusted Life Years)	Federative Republic of Brazil	Both	Age-standardized	Edentulism	Rate	1997	233.5	324.0	152.8
DALYs (Disability-Adjusted Life Years)	Republic of South Africa	Male	Age-standardized	Edentulism	Rate	1997	175.3	247.5	107.0
DALYs (Disability-Adjusted Life Years)	Republic of South Africa	Female	Age-standardized	Edentulism	Rate	1997	182.0	255.9	112.7
DALYs (Disability-Adjusted	Republic of South Africa	Both	Age-standardized	Edentulism	Rate	1997	179.1	252.8	110.2

Life Years)									
DALYs (Disability-Adjusted Life Years)	People's Republic of China	Male	Age-standardized	Edentulism	Rate	1993	72.0	102.7	47.0
DALYs (Disability-Adjusted Life Years)	People's Republic of China	Female	Age-standardized	Edentulism	Rate	1993	94.6	134.7	61.8
DALYs (Disability-Adjusted Life Years)	People's Republic of China	Both	Age-standardized	Edentulism	Rate	1993	83.8	119.6	54.5
DALYs (Disability-Adjusted Life Years)	Republic of Indonesia	Male	Age-standardized	Edentulism	Rate	1993	87.7	122.8	57.0
DALYs (Disability-Adjusted Life Years)	Republic of Indonesia	Female	Age-standardized	Edentulism	Rate	1993	140.8	193.1	89.9
DALYs (Disability-Adjusted Life Years)	Republic of Indonesia	Both	Age-standardized	Edentulism	Rate	1993	115.3	159.3	74.2
DALYs (Disability-Adjusted Life Years)	Russian Federation	Male	Age-standardized	Edentulism	Rate	1993	155.1	209.0	99.4
DALYs (Disability-Adjusted Life Years)	Russian Federation	Female	Age-standardized	Edentulism	Rate	1993	166.9	226.3	107.4
DALYs (Disability-Adjusted Life Years)	Russian Federation	Both	Age-standardized	Edentulism	Rate	1993	163.9	221.8	105.7
DALYs (Disability-Adjusted Life Years)	French Republic	Male	Age-standardized	Edentulism	Rate	1993	71.5	100.1	46.3
DALYs (Disability-Adjusted Life Years)	French Republic	Female	Age-standardized	Edentulism	Rate	1993	99.4	140.2	63.8
DALYs (Disability-Adjusted Life Years)	French Republic	Both	Age-standardized	Edentulism	Rate	1993	87.4	123.0	56.2
DALYs (Disability-Adjusted Life Years)	Republic of Turkey	Male	Age-standardized	Edentulism	Rate	1993	224.4	305.2	142.7
DALYs (Disability-Adjusted Life Years)	Republic of Turkey	Female	Age-standardized	Edentulism	Rate	1993	196.4	267.4	127.3
DALYs	Republic of Turkey	Both	Age-standardized	Edentulism	Rate	1993	209.2	286.2	134.7

(Disability-Adjusted Life Years)									
DALYs (Disability-Adjusted Life Years)	United Mexican States	Male	Age-standardized	Edentulism	Rate	1993	119.3	162.1	76.3
DALYs (Disability-Adjusted Life Years)	United Mexican States	Female	Age-standardized	Edentulism	Rate	1993	206.0	278.5	132.8
DALYs (Disability-Adjusted Life Years)	United Mexican States	Both	Age-standardized	Edentulism	Rate	1993	164.6	223.6	105.9
DALYs (Disability-Adjusted Life Years)	United Kingdom of Great Britain and Northern Ireland	Male	Age-standardized	Edentulism	Rate	1993	104.8	147.1	66.7
DALYs (Disability-Adjusted Life Years)	United Kingdom of Great Britain and Northern Ireland	Female	Age-standardized	Edentulism	Rate	1993	130.8	185.1	82.5
DALYs (Disability-Adjusted Life Years)	United Kingdom of Great Britain and Northern Ireland	Both	Age-standardized	Edentulism	Rate	1993	118.9	168.1	75.2
DALYs (Disability-Adjusted Life Years)	Australia	Male	Age-standardized	Edentulism	Rate	1993	123.3	170.5	82.1
DALYs (Disability-Adjusted Life Years)	Australia	Female	Age-standardized	Edentulism	Rate	1993	170.6	229.6	113.3
DALYs (Disability-Adjusted Life Years)	Australia	Both	Age-standardized	Edentulism	Rate	1993	148.7	202.3	99.4
DALYs (Disability-Adjusted Life Years)	European Union	Male	Age-standardized	Edentulism	Rate	1993	113.2	156.3	74.1
DALYs (Disability-Adjusted Life Years)	European Union	Female	Age-standardized	Edentulism	Rate	1993	143.8	198.9	94.9
DALYs (Disability-Adjusted Life Years)	European Union	Both	Age-standardized	Edentulism	Rate	1993	130.5	180.4	85.5
DALYs (Disability-Adjusted Life Years)	Republic of Italy	Male	Age-standardized	Edentulism	Rate	1993	94.7	133.1	60.3
DALYs (Disability-Adjusted Life Years)	Republic of Italy	Female	Age-standardized	Edentulism	Rate	1993	121.2	171.7	77.4

DALYs (Disability-Adjusted Life Years)	Republic of Italy	Both	Age-standardized	Edentulism	Rate	1993	109.3	154.4	69.6
DALYs (Disability-Adjusted Life Years)	Canada	Male	Age-standardized	Edentulism	Rate	1993	74.9	102.2	47.1
DALYs (Disability-Adjusted Life Years)	Canada	Female	Age-standardized	Edentulism	Rate	1993	97.0	134.8	62.1
DALYs (Disability-Adjusted Life Years)	Canada	Both	Age-standardized	Edentulism	Rate	1993	86.8	119.1	55.5
DALYs (Disability-Adjusted Life Years)	Argentine Republic	Male	Age-standardized	Edentulism	Rate	1993	94.4	133.9	59.1
DALYs (Disability-Adjusted Life Years)	Argentine Republic	Female	Age-standardized	Edentulism	Rate	1993	152.8	217.3	93.9
DALYs (Disability-Adjusted Life Years)	Argentine Republic	Both	Age-standardized	Edentulism	Rate	1993	127.1	181.0	78.9
DALYs (Disability-Adjusted Life Years)	Republic of Korea	Male	Age-standardized	Edentulism	Rate	1998	54.8	77.7	35.2
DALYs (Disability-Adjusted Life Years)	Republic of Korea	Female	Age-standardized	Edentulism	Rate	1998	51.2	71.1	33.8
DALYs (Disability-Adjusted Life Years)	Republic of Korea	Both	Age-standardized	Edentulism	Rate	1998	53.9	75.4	35.9
DALYs (Disability-Adjusted Life Years)	Federal Republic of Germany	Male	Age-standardized	Edentulism	Rate	1998	107.0	147.6	72.0
DALYs (Disability-Adjusted Life Years)	Federal Republic of Germany	Female	Age-standardized	Edentulism	Rate	1998	133.6	187.8	90.8
DALYs (Disability-Adjusted Life Years)	Federal Republic of Germany	Both	Age-standardized	Edentulism	Rate	1998	122.3	170.5	82.8
DALYs (Disability-Adjusted Life Years)	Russian Federation	Male	Age-standardized	Edentulism	Rate	1995	156.0	210.5	100.1
DALYs (Disability-Adjusted	Russian Federation	Female	Age-standardized	Edentulism	Rate	1995	167.8	227.8	107.8

Life Years)									
DALYs (Disability-Adjusted Life Years)	Russian Federation	Both	Age-standardized	Edentulism	Rate	1995	164.8	223.1	106.2
DALYs (Disability-Adjusted Life Years)	People's Republic of China	Male	Age-standardized	Edentulism	Rate	1995	68.5	97.6	44.8
DALYs (Disability-Adjusted Life Years)	People's Republic of China	Female	Age-standardized	Edentulism	Rate	1995	88.5	125.5	58.0
DALYs (Disability-Adjusted Life Years)	People's Republic of China	Both	Age-standardized	Edentulism	Rate	1995	78.9	112.3	51.5
DALYs (Disability-Adjusted Life Years)	Republic of Italy	Male	Age-standardized	Edentulism	Rate	1995	94.4	132.7	60.3
DALYs (Disability-Adjusted Life Years)	Republic of Italy	Female	Age-standardized	Edentulism	Rate	1995	120.8	171.2	76.9
DALYs (Disability-Adjusted Life Years)	Republic of Italy	Both	Age-standardized	Edentulism	Rate	1995	108.9	154.1	69.3
DALYs (Disability-Adjusted Life Years)	Federative Republic of Brazil	Male	Age-standardized	Edentulism	Rate	1998	196.2	275.2	125.2
DALYs (Disability-Adjusted Life Years)	Federative Republic of Brazil	Female	Age-standardized	Edentulism	Rate	1998	261.6	358.6	171.6
DALYs (Disability-Adjusted Life Years)	Federative Republic of Brazil	Both	Age-standardized	Edentulism	Rate	1998	230.8	319.8	150.2
DALYs (Disability-Adjusted Life Years)	Japan	Male	Age-standardized	Edentulism	Rate	1993	81.5	115.2	52.8
DALYs (Disability-Adjusted Life Years)	Japan	Female	Age-standardized	Edentulism	Rate	1993	62.5	88.0	41.7
DALYs (Disability-Adjusted Life Years)	Japan	Both	Age-standardized	Edentulism	Rate	1993	71.5	101.1	47.8
DALYs (Disability-Adjusted Life Years)	United States of America	Male	Age-standardized	Edentulism	Rate	1993	137.9	188.5	87.3
DALYs	United States of	Female	Age-standardized	Edentulism	Rate	1993	146.0	199.5	93.7

(Disability-Adjusted Life Years)	America								
DALYs (Disability-Adjusted Life Years)	United States of America	Both	Age-standardized	Edentulism	Rate	1993	142.6	194.8	91.0
DALYs (Disability-Adjusted Life Years)	Kingdom of Saudi Arabia	Male	Age-standardized	Edentulism	Rate	1998	166.5	229.9	105.7
DALYs (Disability-Adjusted Life Years)	Kingdom of Saudi Arabia	Female	Age-standardized	Edentulism	Rate	1998	112.6	153.3	71.6
DALYs (Disability-Adjusted Life Years)	Kingdom of Saudi Arabia	Both	Age-standardized	Edentulism	Rate	1998	143.3	195.9	90.9
DALYs (Disability-Adjusted Life Years)	Republic of South Africa	Male	Age-standardized	Edentulism	Rate	1998	177.0	249.1	108.3
DALYs (Disability-Adjusted Life Years)	Republic of South Africa	Female	Age-standardized	Edentulism	Rate	1998	190.1	265.0	117.6
DALYs (Disability-Adjusted Life Years)	Republic of South Africa	Both	Age-standardized	Edentulism	Rate	1998	184.5	258.2	113.6
DALYs (Disability-Adjusted Life Years)	Republic of India	Male	Age-standardized	Edentulism	Rate	1998	80.9	112.3	52.0
DALYs (Disability-Adjusted Life Years)	Republic of India	Female	Age-standardized	Edentulism	Rate	1998	85.1	117.1	54.5
DALYs (Disability-Adjusted Life Years)	Republic of India	Both	Age-standardized	Edentulism	Rate	1998	83.0	114.6	53.3
DALYs (Disability-Adjusted Life Years)	Canada	Male	Age-standardized	Edentulism	Rate	1995	75.0	103.3	47.2
DALYs (Disability-Adjusted Life Years)	Canada	Female	Age-standardized	Edentulism	Rate	1995	97.7	134.3	61.8
DALYs (Disability-Adjusted Life Years)	Canada	Both	Age-standardized	Edentulism	Rate	1995	87.2	119.4	55.2
DALYs (Disability-Adjusted Life Years)	United States of America	Male	Age-standardized	Edentulism	Rate	1995	135.8	185.1	86.1

DALYs (Disability-Adjusted Life Years)	United States of America	Female	Age-standardized	Edentulism	Rate	1995	144.3	197.6	92.9
DALYs (Disability-Adjusted Life Years)	United States of America	Both	Age-standardized	Edentulism	Rate	1995	140.7	192.0	90.0
DALYs (Disability-Adjusted Life Years)	Japan	Male	Age-standardized	Edentulism	Rate	1995	80.3	112.6	51.9
DALYs (Disability-Adjusted Life Years)	Japan	Female	Age-standardized	Edentulism	Rate	1995	58.5	81.6	39.1
DALYs (Disability-Adjusted Life Years)	Japan	Both	Age-standardized	Edentulism	Rate	1995	68.7	96.5	45.7
DALYs (Disability-Adjusted Life Years)	Republic of Indonesia	Male	Age-standardized	Edentulism	Rate	1995	86.8	121.1	56.5
DALYs (Disability-Adjusted Life Years)	Republic of Indonesia	Female	Age-standardized	Edentulism	Rate	1995	139.5	191.7	88.9
DALYs (Disability-Adjusted Life Years)	Republic of Indonesia	Both	Age-standardized	Edentulism	Rate	1995	114.2	157.3	73.7
DALYs (Disability-Adjusted Life Years)	French Republic	Male	Age-standardized	Edentulism	Rate	1995	68.0	96.3	44.5
DALYs (Disability-Adjusted Life Years)	French Republic	Female	Age-standardized	Edentulism	Rate	1995	94.5	131.2	61.2
DALYs (Disability-Adjusted Life Years)	French Republic	Both	Age-standardized	Edentulism	Rate	1995	83.0	116.1	54.1
DALYs (Disability-Adjusted Life Years)	Republic of Turkey	Male	Age-standardized	Edentulism	Rate	1995	223.3	305.7	139.1
DALYs (Disability-Adjusted Life Years)	Republic of Turkey	Female	Age-standardized	Edentulism	Rate	1995	195.6	268.1	124.4
DALYs (Disability-Adjusted Life Years)	Republic of Turkey	Both	Age-standardized	Edentulism	Rate	1995	208.2	284.4	130.9
DALYs (Disability-Adjusted	Republic of Korea	Male	Age-standardized	Edentulism	Rate	1999	54.5	77.4	34.7

Life Years)									
DALYs (Disability-Adjusted Life Years)	Republic of Korea	Female	Age-standardized	Edentulism	Rate	1999	51.0	71.7	33.6
DALYs (Disability-Adjusted Life Years)	Republic of Korea	Both	Age-standardized	Edentulism	Rate	1999	53.6	75.7	35.5
DALYs (Disability-Adjusted Life Years)	Australia	Male	Age-standardized	Edentulism	Rate	1995	104.4	148.4	68.4
DALYs (Disability-Adjusted Life Years)	Australia	Female	Age-standardized	Edentulism	Rate	1995	136.8	190.2	88.0
DALYs (Disability-Adjusted Life Years)	Australia	Both	Age-standardized	Edentulism	Rate	1995	122.1	172.1	78.5
DALYs (Disability-Adjusted Life Years)	Federal Republic of Germany	Male	Age-standardized	Edentulism	Rate	1999	107.5	147.8	72.4
DALYs (Disability-Adjusted Life Years)	Federal Republic of Germany	Female	Age-standardized	Edentulism	Rate	1999	134.2	188.0	91.0
DALYs (Disability-Adjusted Life Years)	Federal Republic of Germany	Both	Age-standardized	Edentulism	Rate	1999	122.8	170.3	83.1
DALYs (Disability-Adjusted Life Years)	United Mexican States	Male	Age-standardized	Edentulism	Rate	1995	119.4	162.4	76.6
DALYs (Disability-Adjusted Life Years)	United Mexican States	Female	Age-standardized	Edentulism	Rate	1995	205.6	278.0	132.2
DALYs (Disability-Adjusted Life Years)	United Mexican States	Both	Age-standardized	Edentulism	Rate	1995	164.4	223.7	105.7
DALYs (Disability-Adjusted Life Years)	United Kingdom of Great Britain and Northern Ireland	Male	Age-standardized	Edentulism	Rate	1995	104.8	147.9	66.6
DALYs (Disability-Adjusted Life Years)	United Kingdom of Great Britain and Northern Ireland	Female	Age-standardized	Edentulism	Rate	1995	130.8	184.7	82.0
DALYs (Disability-Adjusted Life Years)	United Kingdom of Great Britain and Northern Ireland	Both	Age-standardized	Edentulism	Rate	1995	118.8	167.9	74.8
DALYs	European Union	Male	Age-standardized	Edentulism	Rate	1995	112.1	154.5	74.1

(Disability-Adjusted Life Years)									
DALYs (Disability-Adjusted Life Years)	European Union	Female	Age-standardized	Edentulism	Rate	1995	142.3	196.9	94.6
DALYs (Disability-Adjusted Life Years)	European Union	Both	Age-standardized	Edentulism	Rate	1995	129.1	178.7	85.4
DALYs (Disability-Adjusted Life Years)	Federative Republic of Brazil	Male	Age-standardized	Edentulism	Rate	1999	195.2	272.6	125.7
DALYs (Disability-Adjusted Life Years)	Federative Republic of Brazil	Female	Age-standardized	Edentulism	Rate	1999	258.6	354.1	169.8
DALYs (Disability-Adjusted Life Years)	Federative Republic of Brazil	Both	Age-standardized	Edentulism	Rate	1999	228.6	316.9	148.6
DALYs (Disability-Adjusted Life Years)	Kingdom of Saudi Arabia	Male	Age-standardized	Edentulism	Rate	1999	166.4	230.0	107.1
DALYs (Disability-Adjusted Life Years)	Kingdom of Saudi Arabia	Female	Age-standardized	Edentulism	Rate	1999	112.7	153.1	72.0
DALYs (Disability-Adjusted Life Years)	Kingdom of Saudi Arabia	Both	Age-standardized	Edentulism	Rate	1999	143.1	196.3	91.5
DALYs (Disability-Adjusted Life Years)	Republic of South Africa	Male	Age-standardized	Edentulism	Rate	1999	178.5	251.1	109.7
DALYs (Disability-Adjusted Life Years)	Republic of South Africa	Female	Age-standardized	Edentulism	Rate	1999	197.0	273.9	121.5
DALYs (Disability-Adjusted Life Years)	Republic of South Africa	Both	Age-standardized	Edentulism	Rate	1999	189.0	263.9	116.4
DALYs (Disability-Adjusted Life Years)	Republic of India	Male	Age-standardized	Edentulism	Rate	1999	76.1	105.7	49.4
DALYs (Disability-Adjusted Life Years)	Republic of India	Female	Age-standardized	Edentulism	Rate	1999	79.4	110.0	51.4
DALYs (Disability-Adjusted Life Years)	Republic of India	Both	Age-standardized	Edentulism	Rate	1999	77.7	107.7	50.4

DALYs (Disability-Adjusted Life Years)	Republic of Indonesia	Male	Age-standardized	Edentulism	Rate	1996	85.2	118.4	55.8
DALYs (Disability-Adjusted Life Years)	Republic of Indonesia	Female	Age-standardized	Edentulism	Rate	1996	137.2	187.9	88.4
DALYs (Disability-Adjusted Life Years)	Republic of Indonesia	Both	Age-standardized	Edentulism	Rate	1996	112.3	155.6	73.0
DALYs (Disability-Adjusted Life Years)	Argentine Republic	Male	Age-standardized	Edentulism	Rate	1995	93.0	130.9	58.6
DALYs (Disability-Adjusted Life Years)	Argentine Republic	Female	Age-standardized	Edentulism	Rate	1995	150.8	216.4	93.6
DALYs (Disability-Adjusted Life Years)	Argentine Republic	Both	Age-standardized	Edentulism	Rate	1995	125.4	178.9	78.7
DALYs (Disability-Adjusted Life Years)	United Kingdom of Great Britain and Northern Ireland	Male	Age-standardized	Edentulism	Rate	1996	108.8	153.2	69.6
DALYs (Disability-Adjusted Life Years)	United Kingdom of Great Britain and Northern Ireland	Female	Age-standardized	Edentulism	Rate	1996	134.0	189.5	84.2
DALYs (Disability-Adjusted Life Years)	United Kingdom of Great Britain and Northern Ireland	Both	Age-standardized	Edentulism	Rate	1996	122.4	173.0	77.4
DALYs (Disability-Adjusted Life Years)	Australia	Male	Age-standardized	Edentulism	Rate	1996	107.5	150.3	69.8
DALYs (Disability-Adjusted Life Years)	Australia	Female	Age-standardized	Edentulism	Rate	1996	142.3	197.9	91.7
DALYs (Disability-Adjusted Life Years)	Australia	Both	Age-standardized	Edentulism	Rate	1996	126.3	175.4	81.8
DALYs (Disability-Adjusted Life Years)	United Mexican States	Male	Age-standardized	Edentulism	Rate	1996	119.8	162.7	77.0
DALYs (Disability-Adjusted Life Years)	United Mexican States	Female	Age-standardized	Edentulism	Rate	1996	203.7	275.6	131.0
DALYs (Disability-Adjusted	United Mexican States	Both	Age-standardized	Edentulism	Rate	1996	163.6	222.3	105.3

Life Years)									
DALYs (Disability-Adjusted Life Years)	Argentine Republic	Male	Age-standardized	Edentulism	Rate	1996	92.3	129.9	57.8
DALYs (Disability-Adjusted Life Years)	Argentine Republic	Female	Age-standardized	Edentulism	Rate	1996	149.5	212.7	94.1
DALYs (Disability-Adjusted Life Years)	Argentine Republic	Both	Age-standardized	Edentulism	Rate	1996	124.4	177.4	78.6
DALYs (Disability-Adjusted Life Years)	Republic of Italy	Male	Age-standardized	Edentulism	Rate	1996	94.3	132.6	59.9
DALYs (Disability-Adjusted Life Years)	Republic of Italy	Female	Age-standardized	Edentulism	Rate	1996	120.7	170.9	76.6
DALYs (Disability-Adjusted Life Years)	Republic of Italy	Both	Age-standardized	Edentulism	Rate	1996	108.8	154.0	69.0
DALYs (Disability-Adjusted Life Years)	Republic of Turkey	Male	Age-standardized	Edentulism	Rate	1996	222.8	303.4	140.8
DALYs (Disability-Adjusted Life Years)	Republic of Turkey	Female	Age-standardized	Edentulism	Rate	1996	195.0	266.8	124.5
DALYs (Disability-Adjusted Life Years)	Republic of Turkey	Both	Age-standardized	Edentulism	Rate	1996	207.6	282.9	132.0
DALYs (Disability-Adjusted Life Years)	Russian Federation	Male	Age-standardized	Edentulism	Rate	1996	156.7	211.3	100.4
DALYs (Disability-Adjusted Life Years)	Russian Federation	Female	Age-standardized	Edentulism	Rate	1996	168.4	228.4	108.0
DALYs (Disability-Adjusted Life Years)	Russian Federation	Both	Age-standardized	Edentulism	Rate	1996	165.3	224.1	106.3
DALYs (Disability-Adjusted Life Years)	European Union	Male	Age-standardized	Edentulism	Rate	1996	112.1	154.7	74.4
DALYs (Disability-Adjusted Life Years)	European Union	Female	Age-standardized	Edentulism	Rate	1996	142.5	196.9	95.0
DALYs	European Union	Both	Age-standardized	Edentulism	Rate	1996	129.2	178.6	85.7

(Disability-Adjusted Life Years)									
DALYs (Disability-Adjusted Life Years)	People's Republic of China	Male	Age-standardized	Edentulism	Rate	1996	67.9	97.1	44.7
DALYs (Disability-Adjusted Life Years)	People's Republic of China	Female	Age-standardized	Edentulism	Rate	1996	84.4	120.0	55.2
DALYs (Disability-Adjusted Life Years)	People's Republic of China	Both	Age-standardized	Edentulism	Rate	1996	76.4	108.7	50.0
DALYs (Disability-Adjusted Life Years)	Canada	Male	Age-standardized	Edentulism	Rate	1996	75.0	102.8	46.9
DALYs (Disability-Adjusted Life Years)	Canada	Female	Age-standardized	Edentulism	Rate	1996	97.8	135.2	62.7
DALYs (Disability-Adjusted Life Years)	Canada	Both	Age-standardized	Edentulism	Rate	1996	87.3	120.6	55.5
DALYs (Disability-Adjusted Life Years)	United States of America	Male	Age-standardized	Edentulism	Rate	1996	135.8	185.4	85.8
DALYs (Disability-Adjusted Life Years)	United States of America	Female	Age-standardized	Edentulism	Rate	1996	144.0	197.0	92.3
DALYs (Disability-Adjusted Life Years)	United States of America	Both	Age-standardized	Edentulism	Rate	1996	140.4	191.4	89.5
DALYs (Disability-Adjusted Life Years)	Japan	Male	Age-standardized	Edentulism	Rate	1996	84.6	119.1	54.8
DALYs (Disability-Adjusted Life Years)	Japan	Female	Age-standardized	Edentulism	Rate	1996	58.6	82.2	39.1
DALYs (Disability-Adjusted Life Years)	Japan	Both	Age-standardized	Edentulism	Rate	1996	70.7	99.6	47.1
DALYs (Disability-Adjusted Life Years)	Republic of Korea	Male	Age-standardized	Edentulism	Rate	2000	54.0	76.7	34.2
DALYs (Disability-Adjusted Life Years)	Republic of Korea	Female	Age-standardized	Edentulism	Rate	2000	50.6	71.1	33.6

DALYs (Disability-Adjusted Life Years)	Republic of Korea	Both	Age-standardized	Edentulism	Rate	2000	53.2	75.5	35.7
DALYs (Disability-Adjusted Life Years)	French Republic	Male	Age-standardized	Edentulism	Rate	1996	69.7	98.7	45.1
DALYs (Disability-Adjusted Life Years)	French Republic	Female	Age-standardized	Edentulism	Rate	1996	96.8	136.2	62.8
DALYs (Disability-Adjusted Life Years)	French Republic	Both	Age-standardized	Edentulism	Rate	1996	85.0	119.3	55.0
DALYs (Disability-Adjusted Life Years)	Federative Republic of Brazil	Male	Age-standardized	Edentulism	Rate	2000	194.8	272.8	125.8
DALYs (Disability-Adjusted Life Years)	Federative Republic of Brazil	Female	Age-standardized	Edentulism	Rate	2000	257.4	353.4	168.3
DALYs (Disability-Adjusted Life Years)	Federative Republic of Brazil	Both	Age-standardized	Edentulism	Rate	2000	227.8	316.3	147.5
DALYs (Disability-Adjusted Life Years)	People's Republic of China	Male	Age-standardized	Edentulism	Rate	1994	69.7	99.5	45.5
DALYs (Disability-Adjusted Life Years)	People's Republic of China	Female	Age-standardized	Edentulism	Rate	1994	91.6	130.0	60.0
DALYs (Disability-Adjusted Life Years)	People's Republic of China	Both	Age-standardized	Edentulism	Rate	1994	81.1	115.7	52.8
DALYs (Disability-Adjusted Life Years)	Federal Republic of Germany	Male	Age-standardized	Edentulism	Rate	2000	107.5	147.6	72.5
DALYs (Disability-Adjusted Life Years)	Federal Republic of Germany	Female	Age-standardized	Edentulism	Rate	2000	134.3	186.1	92.0
DALYs (Disability-Adjusted Life Years)	Federal Republic of Germany	Both	Age-standardized	Edentulism	Rate	2000	122.7	169.1	83.8
DALYs (Disability-Adjusted Life Years)	Republic of South Africa	Male	Age-standardized	Edentulism	Rate	2000	179.3	250.6	111.0
DALYs (Disability-Adjusted	Republic of South Africa	Female	Age-standardized	Edentulism	Rate	2000	199.9	277.1	123.9

Life Years)									
DALYs (Disability-Adjusted Life Years)	Republic of South Africa	Both	Age-standardized	Edentulism	Rate	2000	191.1	266.1	118.1
DALYs (Disability-Adjusted Life Years)	Kingdom of Saudi Arabia	Male	Age-standardized	Edentulism	Rate	2000	166.3	231.0	106.3
DALYs (Disability-Adjusted Life Years)	Kingdom of Saudi Arabia	Female	Age-standardized	Edentulism	Rate	2000	112.8	152.1	72.2
DALYs (Disability-Adjusted Life Years)	Kingdom of Saudi Arabia	Both	Age-standardized	Edentulism	Rate	2000	143.1	196.4	91.0
DALYs (Disability-Adjusted Life Years)	Canada	Male	Age-standardized	Edentulism	Rate	1994	75.1	102.7	46.9
DALYs (Disability-Adjusted Life Years)	Canada	Female	Age-standardized	Edentulism	Rate	1994	97.5	134.6	62.3
DALYs (Disability-Adjusted Life Years)	Canada	Both	Age-standardized	Edentulism	Rate	1994	87.2	119.5	55.2
DALYs (Disability-Adjusted Life Years)	Republic of Italy	Male	Age-standardized	Edentulism	Rate	1994	94.5	132.8	60.1
DALYs (Disability-Adjusted Life Years)	Republic of Italy	Female	Age-standardized	Edentulism	Rate	1994	121.0	171.0	77.1
DALYs (Disability-Adjusted Life Years)	Republic of Italy	Both	Age-standardized	Edentulism	Rate	1994	109.1	154.0	69.4
DALYs (Disability-Adjusted Life Years)	United States of America	Male	Age-standardized	Edentulism	Rate	1994	136.4	186.0	86.5
DALYs (Disability-Adjusted Life Years)	United States of America	Female	Age-standardized	Edentulism	Rate	1994	144.9	197.8	93.1
DALYs (Disability-Adjusted Life Years)	United States of America	Both	Age-standardized	Edentulism	Rate	1994	141.3	192.4	90.3
DALYs (Disability-Adjusted Life Years)	Republic of India	Male	Age-standardized	Edentulism	Rate	2000	73.9	102.9	48.0
DALYs	Republic of India	Female	Age-standardized	Edentulism	Rate	2000	77.0	106.9	50.0

(Disability-Adjusted Life Years)									
DALYs (Disability-Adjusted Life Years)	Republic of India	Both	Age-standardized	Edentulism	Rate	2000	75.4	104.9	49.0
DALYs (Disability-Adjusted Life Years)	Russian Federation	Male	Age-standardized	Edentulism	Rate	1994	155.6	209.8	99.8
DALYs (Disability-Adjusted Life Years)	Russian Federation	Female	Age-standardized	Edentulism	Rate	1994	167.4	227.0	107.7
DALYs (Disability-Adjusted Life Years)	Russian Federation	Both	Age-standardized	Edentulism	Rate	1994	164.4	222.5	106.0
DALYs (Disability-Adjusted Life Years)	United Mexican States	Male	Age-standardized	Edentulism	Rate	1994	119.4	162.2	76.5
DALYs (Disability-Adjusted Life Years)	United Mexican States	Female	Age-standardized	Edentulism	Rate	1994	205.9	278.5	132.7
DALYs (Disability-Adjusted Life Years)	United Mexican States	Both	Age-standardized	Edentulism	Rate	1994	164.6	224.1	105.9
DALYs (Disability-Adjusted Life Years)	Japan	Male	Age-standardized	Edentulism	Rate	1994	80.5	113.5	52.0
DALYs (Disability-Adjusted Life Years)	Japan	Female	Age-standardized	Edentulism	Rate	1994	59.7	83.8	39.8
DALYs (Disability-Adjusted Life Years)	Japan	Both	Age-standardized	Edentulism	Rate	1994	69.4	97.9	46.3
DALYs (Disability-Adjusted Life Years)	Australia	Male	Age-standardized	Edentulism	Rate	1994	109.4	152.7	71.8
DALYs (Disability-Adjusted Life Years)	Australia	Female	Age-standardized	Edentulism	Rate	1994	145.9	200.4	93.6
DALYs (Disability-Adjusted Life Years)	Australia	Both	Age-standardized	Edentulism	Rate	1994	129.2	178.3	83.3
DALYs (Disability-Adjusted Life Years)	Republic of Turkey	Male	Age-standardized	Edentulism	Rate	1994	224.0	304.8	140.8

DALYs (Disability-Adjusted Life Years)	Republic of Turkey	Female	Age-standardized	Edentulism	Rate	1994	195.8	267.8	125.6
DALYs (Disability-Adjusted Life Years)	Republic of Turkey	Both	Age-standardized	Edentulism	Rate	1994	208.7	284.3	132.7
DALYs (Disability-Adjusted Life Years)	Republic of Korea	Male	Age-standardized	Edentulism	Rate	2001	51.9	71.7	33.5
DALYs (Disability-Adjusted Life Years)	Republic of Korea	Female	Age-standardized	Edentulism	Rate	2001	49.0	68.4	32.8
DALYs (Disability-Adjusted Life Years)	Republic of Korea	Both	Age-standardized	Edentulism	Rate	2001	51.3	71.9	34.3
DALYs (Disability-Adjusted Life Years)	United Kingdom of Great Britain and Northern Ireland	Male	Age-standardized	Edentulism	Rate	1994	104.8	148.0	66.7
DALYs (Disability-Adjusted Life Years)	United Kingdom of Great Britain and Northern Ireland	Female	Age-standardized	Edentulism	Rate	1994	130.7	184.9	82.2
DALYs (Disability-Adjusted Life Years)	United Kingdom of Great Britain and Northern Ireland	Both	Age-standardized	Edentulism	Rate	1994	118.9	168.1	75.0
DALYs (Disability-Adjusted Life Years)	Federative Republic of Brazil	Male	Age-standardized	Edentulism	Rate	2001	200.5	280.3	128.8
DALYs (Disability-Adjusted Life Years)	Federative Republic of Brazil	Female	Age-standardized	Edentulism	Rate	2001	267.2	369.9	177.7
DALYs (Disability-Adjusted Life Years)	Federative Republic of Brazil	Both	Age-standardized	Edentulism	Rate	2001	235.7	327.9	155.8
DALYs (Disability-Adjusted Life Years)	Kingdom of Saudi Arabia	Male	Age-standardized	Edentulism	Rate	2001	165.9	229.0	105.9
DALYs (Disability-Adjusted Life Years)	Kingdom of Saudi Arabia	Female	Age-standardized	Edentulism	Rate	2001	112.7	153.5	71.8
DALYs (Disability-Adjusted Life Years)	Kingdom of Saudi Arabia	Both	Age-standardized	Edentulism	Rate	2001	142.7	196.2	91.0
DALYs (Disability-Adjusted	United Mexican States	Male	Age-standardized	Edentulism	Rate	1997	120.6	163.2	77.5

Life Years)									
DALYs (Disability-Adjusted Life Years)	United Mexican States	Female	Age-standardized	Edentulism	Rate	1997	199.5	270.0	128.0
DALYs (Disability-Adjusted Life Years)	United Mexican States	Both	Age-standardized	Edentulism	Rate	1997	161.9	219.6	104.0
DALYs (Disability-Adjusted Life Years)	European Union	Male	Age-standardized	Edentulism	Rate	1994	112.4	155.5	74.3
DALYs (Disability-Adjusted Life Years)	European Union	Female	Age-standardized	Edentulism	Rate	1994	142.7	197.2	94.6
DALYs (Disability-Adjusted Life Years)	European Union	Both	Age-standardized	Edentulism	Rate	1994	129.5	179.2	85.3
DALYs (Disability-Adjusted Life Years)	Federal Republic of Germany	Male	Age-standardized	Edentulism	Rate	2001	107.0	147.7	71.9
DALYs (Disability-Adjusted Life Years)	Federal Republic of Germany	Female	Age-standardized	Edentulism	Rate	2001	133.5	185.8	91.4
DALYs (Disability-Adjusted Life Years)	Federal Republic of Germany	Both	Age-standardized	Edentulism	Rate	2001	121.8	168.1	83.1
DALYs (Disability-Adjusted Life Years)	Republic of South Africa	Male	Age-standardized	Edentulism	Rate	2001	181.8	256.8	111.6
DALYs (Disability-Adjusted Life Years)	Republic of South Africa	Female	Age-standardized	Edentulism	Rate	2001	200.0	280.0	123.3
DALYs (Disability-Adjusted Life Years)	Republic of South Africa	Both	Age-standardized	Edentulism	Rate	2001	192.2	270.3	118.3
DALYs (Disability-Adjusted Life Years)	French Republic	Male	Age-standardized	Edentulism	Rate	1994	68.9	97.3	44.7
DALYs (Disability-Adjusted Life Years)	French Republic	Female	Age-standardized	Edentulism	Rate	1994	95.7	133.5	62.2
DALYs (Disability-Adjusted Life Years)	French Republic	Both	Age-standardized	Edentulism	Rate	1994	84.2	117.4	54.7
DALYs	United States of	Male	Age-standardized	Edentulism	Rate	1997	135.6	184.6	86.0

(Disability-Adjusted Life Years)	America								
DALYs (Disability-Adjusted Life Years)	United States of America	Female	Age-standardized	Edentulism	Rate	1997	143.6	196.5	91.4
DALYs (Disability-Adjusted Life Years)	United States of America	Both	Age-standardized	Edentulism	Rate	1997	140.0	191.0	89.0
DALYs (Disability-Adjusted Life Years)	Russian Federation	Male	Age-standardized	Edentulism	Rate	1997	157.5	212.3	101.2
DALYs (Disability-Adjusted Life Years)	Russian Federation	Female	Age-standardized	Edentulism	Rate	1997	169.0	229.0	108.6
DALYs (Disability-Adjusted Life Years)	Russian Federation	Both	Age-standardized	Edentulism	Rate	1997	166.0	224.7	107.0
DALYs (Disability-Adjusted Life Years)	Republic of Indonesia	Male	Age-standardized	Edentulism	Rate	1994	87.3	122.1	56.9
DALYs (Disability-Adjusted Life Years)	Republic of Indonesia	Female	Age-standardized	Edentulism	Rate	1994	140.1	192.3	89.2
DALYs (Disability-Adjusted Life Years)	Republic of Indonesia	Both	Age-standardized	Edentulism	Rate	1994	114.7	157.9	73.9
DALYs (Disability-Adjusted Life Years)	Republic of India	Male	Age-standardized	Edentulism	Rate	2001	73.6	102.2	47.9
DALYs (Disability-Adjusted Life Years)	Republic of India	Female	Age-standardized	Edentulism	Rate	2001	77.0	106.6	49.7
DALYs (Disability-Adjusted Life Years)	Republic of India	Both	Age-standardized	Edentulism	Rate	2001	75.3	104.4	48.8
DALYs (Disability-Adjusted Life Years)	Argentine Republic	Male	Age-standardized	Edentulism	Rate	1994	93.7	133.2	58.9
DALYs (Disability-Adjusted Life Years)	Argentine Republic	Female	Age-standardized	Edentulism	Rate	1994	151.8	215.4	94.1
DALYs (Disability-Adjusted Life Years)	Argentine Republic	Both	Age-standardized	Edentulism	Rate	1994	126.2	180.4	79.1

DALYs (Disability-Adjusted Life Years)	Republic of Turkey	Male	Age-standardized	Edentulism	Rate	1997	221.9	300.4	139.6
DALYs (Disability-Adjusted Life Years)	Republic of Turkey	Female	Age-standardized	Edentulism	Rate	1997	194.2	264.1	124.5
DALYs (Disability-Adjusted Life Years)	Republic of Turkey	Both	Age-standardized	Edentulism	Rate	1997	206.7	280.3	131.1
DALYs (Disability-Adjusted Life Years)	Australia	Male	Age-standardized	Edentulism	Rate	1997	114.8	159.8	73.2
DALYs (Disability-Adjusted Life Years)	Australia	Female	Age-standardized	Edentulism	Rate	1997	154.9	213.9	98.6
DALYs (Disability-Adjusted Life Years)	Australia	Both	Age-standardized	Edentulism	Rate	1997	136.4	189.6	87.1
DALYs (Disability-Adjusted Life Years)	Republic of Korea	Male	Age-standardized	Edentulism	Rate	2002	47.8	66.7	31.6
DALYs (Disability-Adjusted Life Years)	Republic of Korea	Female	Age-standardized	Edentulism	Rate	2002	45.4	63.5	30.5
DALYs (Disability-Adjusted Life Years)	Republic of Korea	Both	Age-standardized	Edentulism	Rate	2002	47.3	66.7	31.9
DALYs (Disability-Adjusted Life Years)	United Kingdom of Great Britain and Northern Ireland	Male	Age-standardized	Edentulism	Rate	1997	118.4	165.7	76.0
DALYs (Disability-Adjusted Life Years)	United Kingdom of Great Britain and Northern Ireland	Female	Age-standardized	Edentulism	Rate	1997	141.6	199.4	89.3
DALYs (Disability-Adjusted Life Years)	United Kingdom of Great Britain and Northern Ireland	Both	Age-standardized	Edentulism	Rate	1997	131.0	184.3	83.2
DALYs (Disability-Adjusted Life Years)	People's Republic of China	Male	Age-standardized	Edentulism	Rate	1997	67.4	96.4	44.8
DALYs (Disability-Adjusted Life Years)	People's Republic of China	Female	Age-standardized	Edentulism	Rate	1997	79.3	112.3	51.7
DALYs (Disability-Adjusted	People's Republic of China	Both	Age-standardized	Edentulism	Rate	1997	73.4	104.4	48.2

Life Years)									
DALYs (Disability-Adjusted Life Years)	Canada	Male	Age-standardized	Edentulism	Rate	1997	74.8	101.8	46.4
DALYs (Disability-Adjusted Life Years)	Canada	Female	Age-standardized	Edentulism	Rate	1997	97.7	134.8	60.6
DALYs (Disability-Adjusted Life Years)	Canada	Both	Age-standardized	Edentulism	Rate	1997	87.1	119.8	54.2
DALYs (Disability-Adjusted Life Years)	United States of America	Male	Age-standardized	Edentulism	Rate	1998	135.2	184.7	86.0
DALYs (Disability-Adjusted Life Years)	United States of America	Female	Age-standardized	Edentulism	Rate	1998	143.0	195.1	91.9
DALYs (Disability-Adjusted Life Years)	United States of America	Both	Age-standardized	Edentulism	Rate	1998	139.4	190.2	89.1
DALYs (Disability-Adjusted Life Years)	European Union	Male	Age-standardized	Edentulism	Rate	1997	112.4	155.0	74.9
DALYs (Disability-Adjusted Life Years)	European Union	Female	Age-standardized	Edentulism	Rate	1997	143.0	197.7	95.7
DALYs (Disability-Adjusted Life Years)	European Union	Both	Age-standardized	Edentulism	Rate	1997	129.5	179.2	86.4
DALYs (Disability-Adjusted Life Years)	Federal Republic of Germany	Male	Age-standardized	Edentulism	Rate	2002	105.5	144.6	71.1
DALYs (Disability-Adjusted Life Years)	Federal Republic of Germany	Female	Age-standardized	Edentulism	Rate	2002	131.3	180.6	90.2
DALYs (Disability-Adjusted Life Years)	Federal Republic of Germany	Both	Age-standardized	Edentulism	Rate	2002	119.6	164.2	81.6
DALYs (Disability-Adjusted Life Years)	Republic of Indonesia	Male	Age-standardized	Edentulism	Rate	1997	82.2	115.1	53.6
DALYs (Disability-Adjusted Life Years)	Republic of Indonesia	Female	Age-standardized	Edentulism	Rate	1997	132.8	183.9	86.1
DALYs	Republic of	Both	Age-standardized	Edentulism	Rate	1997	108.6	151.1	70.6

(Disability-Adjusted Life Years)	Indonesia								
DALYs (Disability-Adjusted Life Years)	Republic of Italy	Male	Age-standardized	Edentulism	Rate	1997	94.1	132.4	59.9
DALYs (Disability-Adjusted Life Years)	Republic of Italy	Female	Age-standardized	Edentulism	Rate	1997	120.6	170.4	76.4
DALYs (Disability-Adjusted Life Years)	Republic of Italy	Both	Age-standardized	Edentulism	Rate	1997	108.6	153.8	68.9
DALYs (Disability-Adjusted Life Years)	Federative Republic of Brazil	Male	Age-standardized	Edentulism	Rate	2002	213.6	297.8	138.5
DALYs (Disability-Adjusted Life Years)	Federative Republic of Brazil	Female	Age-standardized	Edentulism	Rate	2002	290.7	405.3	193.7
DALYs (Disability-Adjusted Life Years)	Federative Republic of Brazil	Both	Age-standardized	Edentulism	Rate	2002	254.4	354.6	170.8
DALYs (Disability-Adjusted Life Years)	Kingdom of Saudi Arabia	Male	Age-standardized	Edentulism	Rate	2002	165.5	228.0	105.2
DALYs (Disability-Adjusted Life Years)	Kingdom of Saudi Arabia	Female	Age-standardized	Edentulism	Rate	2002	112.5	152.9	72.0
DALYs (Disability-Adjusted Life Years)	Kingdom of Saudi Arabia	Both	Age-standardized	Edentulism	Rate	2002	142.3	195.1	90.5
DALYs (Disability-Adjusted Life Years)	People's Republic of China	Male	Age-standardized	Edentulism	Rate	1998	67.2	95.6	44.7
DALYs (Disability-Adjusted Life Years)	People's Republic of China	Female	Age-standardized	Edentulism	Rate	1998	74.2	105.3	48.9
DALYs (Disability-Adjusted Life Years)	People's Republic of China	Both	Age-standardized	Edentulism	Rate	1998	70.6	100.8	46.8
DALYs (Disability-Adjusted Life Years)	Republic of South Africa	Male	Age-standardized	Edentulism	Rate	2002	187.4	267.3	114.9
DALYs (Disability-Adjusted Life Years)	Republic of South Africa	Female	Age-standardized	Edentulism	Rate	2002	199.4	280.5	122.1

DALYs (Disability-Adjusted Life Years)	Republic of South Africa	Both	Age-standardized	Edentulism	Rate	2002	194.3	274.9	119.1
DALYs (Disability-Adjusted Life Years)	Republic of Italy	Male	Age-standardized	Edentulism	Rate	1998	93.9	131.9	59.6
DALYs (Disability-Adjusted Life Years)	Republic of Italy	Female	Age-standardized	Edentulism	Rate	1998	120.5	170.6	76.0
DALYs (Disability-Adjusted Life Years)	Republic of Italy	Both	Age-standardized	Edentulism	Rate	1998	108.5	153.8	68.5
DALYs (Disability-Adjusted Life Years)	Japan	Male	Age-standardized	Edentulism	Rate	1997	95.0	134.4	61.3
DALYs (Disability-Adjusted Life Years)	Japan	Female	Age-standardized	Edentulism	Rate	1997	59.0	83.1	39.4
DALYs (Disability-Adjusted Life Years)	Japan	Both	Age-standardized	Edentulism	Rate	1997	75.6	107.0	50.6
DALYs (Disability-Adjusted Life Years)	Canada	Male	Age-standardized	Edentulism	Rate	1998	74.4	102.6	46.6
DALYs (Disability-Adjusted Life Years)	Canada	Female	Age-standardized	Edentulism	Rate	1998	97.6	134.6	61.4
DALYs (Disability-Adjusted Life Years)	Canada	Both	Age-standardized	Edentulism	Rate	1998	86.8	119.4	54.5
DALYs (Disability-Adjusted Life Years)	Republic of India	Male	Age-standardized	Edentulism	Rate	2002	73.6	102.1	47.6
DALYs (Disability-Adjusted Life Years)	Republic of India	Female	Age-standardized	Edentulism	Rate	2002	77.5	107.2	49.9
DALYs (Disability-Adjusted Life Years)	Republic of India	Both	Age-standardized	Edentulism	Rate	2002	75.6	104.6	48.7
DALYs (Disability-Adjusted Life Years)	Argentine Republic	Male	Age-standardized	Edentulism	Rate	1997	91.5	130.0	58.0
DALYs (Disability-Adjusted	Argentine Republic	Female	Age-standardized	Edentulism	Rate	1997	147.9	211.9	91.5

Life Years)									
DALYs (Disability-Adjusted Life Years)	Argentine Republic	Both	Age-standardized	Edentulism	Rate	1997	123.2	175.1	77.1
DALYs (Disability-Adjusted Life Years)	French Republic	Male	Age-standardized	Edentulism	Rate	1997	73.7	103.1	47.9
DALYs (Disability-Adjusted Life Years)	French Republic	Female	Age-standardized	Edentulism	Rate	1997	102.4	143.4	65.6
DALYs (Disability-Adjusted Life Years)	French Republic	Both	Age-standardized	Edentulism	Rate	1997	89.8	125.7	57.9
DALYs (Disability-Adjusted Life Years)	Japan	Male	Age-standardized	Edentulism	Rate	1998	107.9	152.3	68.6
DALYs (Disability-Adjusted Life Years)	Japan	Female	Age-standardized	Edentulism	Rate	1998	59.6	84.3	40.0
DALYs (Disability-Adjusted Life Years)	Japan	Both	Age-standardized	Edentulism	Rate	1998	81.8	115.9	54.7
DALYs (Disability-Adjusted Life Years)	Australia	Male	Age-standardized	Edentulism	Rate	1998	123.7	170.8	80.1
DALYs (Disability-Adjusted Life Years)	Australia	Female	Age-standardized	Edentulism	Rate	1998	170.2	231.7	108.0
DALYs (Disability-Adjusted Life Years)	Australia	Both	Age-standardized	Edentulism	Rate	1998	148.6	202.8	95.2
DALYs (Disability-Adjusted Life Years)	United Kingdom of Great Britain and Northern Ireland	Male	Age-standardized	Edentulism	Rate	1998	129.7	180.2	83.7
DALYs (Disability-Adjusted Life Years)	United Kingdom of Great Britain and Northern Ireland	Female	Age-standardized	Edentulism	Rate	1998	150.7	212.3	94.9
DALYs (Disability-Adjusted Life Years)	United Kingdom of Great Britain and Northern Ireland	Both	Age-standardized	Edentulism	Rate	1998	141.3	197.7	89.9
DALYs (Disability-Adjusted Life Years)	French Republic	Male	Age-standardized	Edentulism	Rate	1998	78.6	109.5	50.3
DALYs	French Republic	Female	Age-standardized	Edentulism	Rate	1998	109.5	153.6	71.1

(Disability-Adjusted Life Years)									
DALYs (Disability-Adjusted Life Years)	French Republic	Both	Age-standardized	Edentulism	Rate	1998	95.8	134.6	62.2
DALYs (Disability-Adjusted Life Years)	Russian Federation	Male	Age-standardized	Edentulism	Rate	1998	158.5	213.1	102.2
DALYs (Disability-Adjusted Life Years)	Russian Federation	Female	Age-standardized	Edentulism	Rate	1998	169.7	230.7	108.9
DALYs (Disability-Adjusted Life Years)	Russian Federation	Both	Age-standardized	Edentulism	Rate	1998	166.8	226.0	107.6
DALYs (Disability-Adjusted Life Years)	Republic of Indonesia	Male	Age-standardized	Edentulism	Rate	1998	78.7	111.0	51.4
DALYs (Disability-Adjusted Life Years)	Republic of Indonesia	Female	Age-standardized	Edentulism	Rate	1998	127.8	177.9	82.4
DALYs (Disability-Adjusted Life Years)	Republic of Indonesia	Both	Age-standardized	Edentulism	Rate	1998	104.4	145.9	67.6
DALYs (Disability-Adjusted Life Years)	United Mexican States	Male	Age-standardized	Edentulism	Rate	1998	121.5	164.8	78.0
DALYs (Disability-Adjusted Life Years)	United Mexican States	Female	Age-standardized	Edentulism	Rate	1998	194.5	262.7	124.8
DALYs (Disability-Adjusted Life Years)	United Mexican States	Both	Age-standardized	Edentulism	Rate	1998	159.8	216.0	102.5
DALYs (Disability-Adjusted Life Years)	Argentine Republic	Male	Age-standardized	Edentulism	Rate	1998	90.6	128.5	57.7
DALYs (Disability-Adjusted Life Years)	Argentine Republic	Female	Age-standardized	Edentulism	Rate	1998	146.5	208.3	92.1
DALYs (Disability-Adjusted Life Years)	Argentine Republic	Both	Age-standardized	Edentulism	Rate	1998	122.0	172.7	77.0
DALYs (Disability-Adjusted Life Years)	European Union	Male	Age-standardized	Edentulism	Rate	1998	112.8	156.1	75.4

DALYs (Disability-Adjusted Life Years)	European Union	Female	Age-standardized	Edentulism	Rate	1998	143.6	198.5	95.9
DALYs (Disability-Adjusted Life Years)	European Union	Both	Age-standardized	Edentulism	Rate	1998	130.1	179.9	86.8
DALYs (Disability-Adjusted Life Years)	Republic of Korea	Male	Age-standardized	Edentulism	Rate	2003	43.1	59.8	29.0
DALYs (Disability-Adjusted Life Years)	Republic of Korea	Female	Age-standardized	Edentulism	Rate	2003	41.4	57.3	28.1
DALYs (Disability-Adjusted Life Years)	Republic of Korea	Both	Age-standardized	Edentulism	Rate	2003	42.9	59.8	29.4
DALYs (Disability-Adjusted Life Years)	Federal Republic of Germany	Male	Age-standardized	Edentulism	Rate	2003	103.3	143.3	70.2
DALYs (Disability-Adjusted Life Years)	Federal Republic of Germany	Female	Age-standardized	Edentulism	Rate	2003	128.1	178.6	87.6
DALYs (Disability-Adjusted Life Years)	Federal Republic of Germany	Both	Age-standardized	Edentulism	Rate	2003	116.5	161.8	79.5
DALYs (Disability-Adjusted Life Years)	Republic of Turkey	Male	Age-standardized	Edentulism	Rate	1998	221.0	303.2	141.4
DALYs (Disability-Adjusted Life Years)	Republic of Turkey	Female	Age-standardized	Edentulism	Rate	1998	193.4	263.8	123.0
DALYs (Disability-Adjusted Life Years)	Republic of Turkey	Both	Age-standardized	Edentulism	Rate	1998	205.8	281.2	131.4
DALYs (Disability-Adjusted Life Years)	Kingdom of Saudi Arabia	Male	Age-standardized	Edentulism	Rate	2003	164.8	226.3	104.9
DALYs (Disability-Adjusted Life Years)	Kingdom of Saudi Arabia	Female	Age-standardized	Edentulism	Rate	2003	112.2	151.6	72.0
DALYs (Disability-Adjusted Life Years)	Kingdom of Saudi Arabia	Both	Age-standardized	Edentulism	Rate	2003	141.7	194.2	90.5
DALYs (Disability-Adjusted	Republic of India	Male	Age-standardized	Edentulism	Rate	2003	74.0	102.8	47.6

Life Years)									
DALYs (Disability-Adjusted Life Years)	Republic of India	Female	Age-standardized	Edentulism	Rate	2003	78.5	108.6	50.3
DALYs (Disability-Adjusted Life Years)	Republic of India	Both	Age-standardized	Edentulism	Rate	2003	76.3	105.8	49.0
DALYs (Disability-Adjusted Life Years)	Republic of South Africa	Male	Age-standardized	Edentulism	Rate	2003	193.7	279.2	117.3
DALYs (Disability-Adjusted Life Years)	Republic of South Africa	Female	Age-standardized	Edentulism	Rate	2003	197.8	281.3	120.7
DALYs (Disability-Adjusted Life Years)	Republic of South Africa	Both	Age-standardized	Edentulism	Rate	2003	196.2	280.6	119.3
DALYs (Disability-Adjusted Life Years)	Federative Republic of Brazil	Male	Age-standardized	Edentulism	Rate	2003	229.1	320.4	151.9
DALYs (Disability-Adjusted Life Years)	Federative Republic of Brazil	Female	Age-standardized	Edentulism	Rate	2003	318.7	441.9	214.7
DALYs (Disability-Adjusted Life Years)	Federative Republic of Brazil	Both	Age-standardized	Edentulism	Rate	2003	276.5	385.3	186.9
DALYs (Disability-Adjusted Life Years)	Russian Federation	Male	Age-standardized	Edentulism	Rate	1999	159.3	214.3	102.7
DALYs (Disability-Adjusted Life Years)	Russian Federation	Female	Age-standardized	Edentulism	Rate	1999	170.2	231.4	109.1
DALYs (Disability-Adjusted Life Years)	Russian Federation	Both	Age-standardized	Edentulism	Rate	1999	167.4	226.8	107.8
DALYs (Disability-Adjusted Life Years)	United Kingdom of Great Britain and Northern Ireland	Male	Age-standardized	Edentulism	Rate	1999	139.2	193.4	90.2
DALYs (Disability-Adjusted Life Years)	United Kingdom of Great Britain and Northern Ireland	Female	Age-standardized	Edentulism	Rate	1999	158.3	222.1	100.1
DALYs (Disability-Adjusted Life Years)	United Kingdom of Great Britain and Northern Ireland	Both	Age-standardized	Edentulism	Rate	1999	149.8	209.8	95.8
DALYs	United Mexican	Male	Age-standardized	Edentulism	Rate	1999	122.4	166.0	79.0

(Disability-Adjusted Life Years)	States								
DALYs (Disability-Adjusted Life Years)	United Mexican States	Female	Age-standardized	Edentulism	Rate	1999	190.2	256.7	121.9
DALYs (Disability-Adjusted Life Years)	United Mexican States	Both	Age-standardized	Edentulism	Rate	1999	158.0	213.1	101.4
DALYs (Disability-Adjusted Life Years)	United States of America	Male	Age-standardized	Edentulism	Rate	1999	134.8	183.9	85.7
DALYs (Disability-Adjusted Life Years)	United States of America	Female	Age-standardized	Edentulism	Rate	1999	142.4	194.1	91.8
DALYs (Disability-Adjusted Life Years)	United States of America	Both	Age-standardized	Edentulism	Rate	1999	138.7	189.1	88.9
DALYs (Disability-Adjusted Life Years)	People's Republic of China	Male	Age-standardized	Edentulism	Rate	1999	67.1	95.5	44.7
DALYs (Disability-Adjusted Life Years)	People's Republic of China	Female	Age-standardized	Edentulism	Rate	1999	70.3	100.2	46.9
DALYs (Disability-Adjusted Life Years)	People's Republic of China	Both	Age-standardized	Edentulism	Rate	1999	68.5	97.5	45.5
DALYs (Disability-Adjusted Life Years)	Republic of Turkey	Male	Age-standardized	Edentulism	Rate	1999	219.7	300.1	141.4
DALYs (Disability-Adjusted Life Years)	Republic of Turkey	Female	Age-standardized	Edentulism	Rate	1999	192.7	262.6	122.9
DALYs (Disability-Adjusted Life Years)	Republic of Turkey	Both	Age-standardized	Edentulism	Rate	1999	204.8	279.2	131.3
DALYs (Disability-Adjusted Life Years)	Australia	Male	Age-standardized	Edentulism	Rate	1999	131.4	181.1	84.9
DALYs (Disability-Adjusted Life Years)	Australia	Female	Age-standardized	Edentulism	Rate	1999	182.8	250.9	116.8
DALYs (Disability-Adjusted Life Years)	Australia	Both	Age-standardized	Edentulism	Rate	1999	158.8	217.9	102.1

DALYs (Disability-Adjusted Life Years)	Canada	Male	Age-standardized	Edentulism	Rate	1999	74.1	101.9	47.1
DALYs (Disability-Adjusted Life Years)	Canada	Female	Age-standardized	Edentulism	Rate	1999	97.5	134.8	60.8
DALYs (Disability-Adjusted Life Years)	Canada	Both	Age-standardized	Edentulism	Rate	1999	86.7	119.5	54.4
DALYs (Disability-Adjusted Life Years)	Argentine Republic	Male	Age-standardized	Edentulism	Rate	1999	90.0	125.3	58.0
DALYs (Disability-Adjusted Life Years)	Argentine Republic	Female	Age-standardized	Edentulism	Rate	1999	145.3	206.3	90.7
DALYs (Disability-Adjusted Life Years)	Argentine Republic	Both	Age-standardized	Edentulism	Rate	1999	121.1	170.2	76.1
DALYs (Disability-Adjusted Life Years)	Republic of Italy	Male	Age-standardized	Edentulism	Rate	1999	93.8	131.5	58.9
DALYs (Disability-Adjusted Life Years)	Republic of Italy	Female	Age-standardized	Edentulism	Rate	1999	120.3	170.6	76.0
DALYs (Disability-Adjusted Life Years)	Republic of Italy	Both	Age-standardized	Edentulism	Rate	1999	108.3	153.6	68.2
DALYs (Disability-Adjusted Life Years)	European Union	Male	Age-standardized	Edentulism	Rate	1999	113.1	156.5	75.4
DALYs (Disability-Adjusted Life Years)	European Union	Female	Age-standardized	Edentulism	Rate	1999	144.2	199.6	96.5
DALYs (Disability-Adjusted Life Years)	European Union	Both	Age-standardized	Edentulism	Rate	1999	130.5	180.7	87.1
DALYs (Disability-Adjusted Life Years)	Japan	Male	Age-standardized	Edentulism	Rate	1999	119.3	168.8	75.9
DALYs (Disability-Adjusted Life Years)	Japan	Female	Age-standardized	Edentulism	Rate	1999	60.4	85.2	40.5
DALYs (Disability-Adjusted	Japan	Both	Age-standardized	Edentulism	Rate	1999	87.4	123.8	58.3

Life Years)									
DALYs (Disability-Adjusted Life Years)	Republic of Korea	Male	Age-standardized	Edentulism	Rate	2004	39.0	54.6	26.3
DALYs (Disability-Adjusted Life Years)	Republic of Korea	Female	Age-standardized	Edentulism	Rate	2004	37.9	52.6	25.7
DALYs (Disability-Adjusted Life Years)	Republic of Korea	Both	Age-standardized	Edentulism	Rate	2004	39.0	54.1	26.3
DALYs (Disability-Adjusted Life Years)	People's Republic of China	Male	Age-standardized	Edentulism	Rate	2000	67.3	95.8	44.8
DALYs (Disability-Adjusted Life Years)	People's Republic of China	Female	Age-standardized	Edentulism	Rate	2000	68.8	97.9	46.0
DALYs (Disability-Adjusted Life Years)	People's Republic of China	Both	Age-standardized	Edentulism	Rate	2000	67.7	96.3	45.1
DALYs (Disability-Adjusted Life Years)	Federal Republic of Germany	Male	Age-standardized	Edentulism	Rate	2004	100.6	140.3	67.3
DALYs (Disability-Adjusted Life Years)	Federal Republic of Germany	Female	Age-standardized	Edentulism	Rate	2004	124.2	171.6	85.4
DALYs (Disability-Adjusted Life Years)	Federal Republic of Germany	Both	Age-standardized	Edentulism	Rate	2004	112.8	156.0	77.0
DALYs (Disability-Adjusted Life Years)	United States of America	Male	Age-standardized	Edentulism	Rate	2000	134.1	182.3	84.8
DALYs (Disability-Adjusted Life Years)	United States of America	Female	Age-standardized	Edentulism	Rate	2000	141.5	192.0	90.4
DALYs (Disability-Adjusted Life Years)	United States of America	Both	Age-standardized	Edentulism	Rate	2000	137.9	187.0	87.7
DALYs (Disability-Adjusted Life Years)	Australia	Male	Age-standardized	Edentulism	Rate	2000	134.4	185.8	86.1
DALYs (Disability-Adjusted Life Years)	Australia	Female	Age-standardized	Edentulism	Rate	2000	188.1	256.7	120.4
DALYs	Australia	Both	Age-standardized	Edentulism	Rate	2000	162.9	224.8	104.6

(Disability-Adjusted Life Years)									
DALYs (Disability-Adjusted Life Years)	Canada	Male	Age-standardized	Edentulism	Rate	2000	74.0	101.4	45.9
DALYs (Disability-Adjusted Life Years)	Canada	Female	Age-standardized	Edentulism	Rate	2000	97.4	134.1	59.5
DALYs (Disability-Adjusted Life Years)	Canada	Both	Age-standardized	Edentulism	Rate	2000	86.6	118.8	53.4
DALYs (Disability-Adjusted Life Years)	Republic of Indonesia	Male	Age-standardized	Edentulism	Rate	1999	75.8	107.3	50.0
DALYs (Disability-Adjusted Life Years)	Republic of Indonesia	Female	Age-standardized	Edentulism	Rate	1999	123.6	172.6	79.8
DALYs (Disability-Adjusted Life Years)	Republic of Indonesia	Both	Age-standardized	Edentulism	Rate	1999	101.0	141.6	65.5
DALYs (Disability-Adjusted Life Years)	Federative Republic of Brazil	Male	Age-standardized	Edentulism	Rate	2004	242.0	335.7	161.4
DALYs (Disability-Adjusted Life Years)	Federative Republic of Brazil	Female	Age-standardized	Edentulism	Rate	2004	342.3	471.5	229.4
DALYs (Disability-Adjusted Life Years)	Federative Republic of Brazil	Both	Age-standardized	Edentulism	Rate	2004	295.1	408.7	197.3
DALYs (Disability-Adjusted Life Years)	Kingdom of Saudi Arabia	Male	Age-standardized	Edentulism	Rate	2004	164.2	226.3	104.0
DALYs (Disability-Adjusted Life Years)	Kingdom of Saudi Arabia	Female	Age-standardized	Edentulism	Rate	2004	112.0	151.7	71.2
DALYs (Disability-Adjusted Life Years)	Kingdom of Saudi Arabia	Both	Age-standardized	Edentulism	Rate	2004	141.4	193.5	89.9
DALYs (Disability-Adjusted Life Years)	French Republic	Male	Age-standardized	Edentulism	Rate	1999	82.7	115.4	53.3
DALYs (Disability-Adjusted Life Years)	French Republic	Female	Age-standardized	Edentulism	Rate	1999	115.4	161.5	73.3

DALYs (Disability-Adjusted Life Years)	French Republic	Both	Age-standardized	Edentulism	Rate	1999	100.8	141.2	64.7
DALYs (Disability-Adjusted Life Years)	Republic of South Africa	Male	Age-standardized	Edentulism	Rate	2004	198.1	287.4	118.3
DALYs (Disability-Adjusted Life Years)	Republic of South Africa	Female	Age-standardized	Edentulism	Rate	2004	194.8	280.7	116.9
DALYs (Disability-Adjusted Life Years)	Republic of South Africa	Both	Age-standardized	Edentulism	Rate	2004	196.4	284.1	117.5
DALYs (Disability-Adjusted Life Years)	Japan	Male	Age-standardized	Edentulism	Rate	2000	125.7	178.0	80.1
DALYs (Disability-Adjusted Life Years)	Japan	Female	Age-standardized	Edentulism	Rate	2000	61.3	86.8	41.1
DALYs (Disability-Adjusted Life Years)	Japan	Both	Age-standardized	Edentulism	Rate	2000	90.9	128.8	60.0
DALYs (Disability-Adjusted Life Years)	United Mexican States	Male	Age-standardized	Edentulism	Rate	2000	122.8	166.0	79.5
DALYs (Disability-Adjusted Life Years)	United Mexican States	Female	Age-standardized	Edentulism	Rate	2000	188.5	254.4	120.9
DALYs (Disability-Adjusted Life Years)	United Mexican States	Both	Age-standardized	Edentulism	Rate	2000	157.3	212.2	101.2
DALYs (Disability-Adjusted Life Years)	Republic of India	Male	Age-standardized	Edentulism	Rate	2004	75.1	103.9	47.8
DALYs (Disability-Adjusted Life Years)	Republic of India	Female	Age-standardized	Edentulism	Rate	2004	80.1	110.5	51.0
DALYs (Disability-Adjusted Life Years)	Republic of India	Both	Age-standardized	Edentulism	Rate	2004	77.6	107.5	49.5
DALYs (Disability-Adjusted Life Years)	United Mexican States	Male	Age-standardized	Edentulism	Rate	2001	122.9	166.3	79.4
DALYs (Disability-Adjusted	United Mexican States	Female	Age-standardized	Edentulism	Rate	2001	188.8	254.9	122.5

Life Years)									
DALYs (Disability-Adjusted Life Years)	United Mexican States	Both	Age-standardized	Edentulism	Rate	2001	157.6	212.5	102.0
DALYs (Disability-Adjusted Life Years)	European Union	Male	Age-standardized	Edentulism	Rate	2000	113.3	156.6	75.6
DALYs (Disability-Adjusted Life Years)	European Union	Female	Age-standardized	Edentulism	Rate	2000	144.4	199.7	96.9
DALYs (Disability-Adjusted Life Years)	European Union	Both	Age-standardized	Edentulism	Rate	2000	130.6	180.6	87.4
DALYs (Disability-Adjusted Life Years)	Federative Republic of Brazil	Male	Age-standardized	Edentulism	Rate	2005	247.2	341.4	164.6
DALYs (Disability-Adjusted Life Years)	Federative Republic of Brazil	Female	Age-standardized	Edentulism	Rate	2005	352.4	481.5	234.3
DALYs (Disability-Adjusted Life Years)	Federative Republic of Brazil	Both	Age-standardized	Edentulism	Rate	2005	302.9	417.4	203.5
DALYs (Disability-Adjusted Life Years)	Republic of Korea	Male	Age-standardized	Edentulism	Rate	2005	37.1	50.7	25.1
DALYs (Disability-Adjusted Life Years)	Republic of Korea	Female	Age-standardized	Edentulism	Rate	2005	36.1	50.2	24.7
DALYs (Disability-Adjusted Life Years)	Republic of Korea	Both	Age-standardized	Edentulism	Rate	2005	37.1	51.2	25.2
DALYs (Disability-Adjusted Life Years)	Russian Federation	Male	Age-standardized	Edentulism	Rate	2000	159.7	214.7	102.9
DALYs (Disability-Adjusted Life Years)	Russian Federation	Female	Age-standardized	Edentulism	Rate	2000	170.5	231.6	109.2
DALYs (Disability-Adjusted Life Years)	Russian Federation	Both	Age-standardized	Edentulism	Rate	2000	167.7	227.2	107.9
DALYs (Disability-Adjusted Life Years)	Federal Republic of Germany	Male	Age-standardized	Edentulism	Rate	2005	97.7	137.5	65.7
DALYs	Federal Republic	Female	Age-standardized	Edentulism	Rate	2005	120.1	165.7	82.1

(Disability-Adjusted Life Years)	of Germany								
DALYs (Disability-Adjusted Life Years)	Federal Republic of Germany	Both	Age-standardized	Edentulism	Rate	2005	109.1	151.6	74.0
DALYs (Disability-Adjusted Life Years)	Kingdom of Saudi Arabia	Male	Age-standardized	Edentulism	Rate	2005	163.4	223.6	102.5
DALYs (Disability-Adjusted Life Years)	Kingdom of Saudi Arabia	Female	Age-standardized	Edentulism	Rate	2005	111.6	152.3	70.7
DALYs (Disability-Adjusted Life Years)	Kingdom of Saudi Arabia	Both	Age-standardized	Edentulism	Rate	2005	140.9	192.4	89.0
DALYs (Disability-Adjusted Life Years)	Republic of Italy	Male	Age-standardized	Edentulism	Rate	2000	93.8	131.5	59.1
DALYs (Disability-Adjusted Life Years)	Republic of Italy	Female	Age-standardized	Edentulism	Rate	2000	120.2	170.6	75.7
DALYs (Disability-Adjusted Life Years)	Republic of Italy	Both	Age-standardized	Edentulism	Rate	2000	108.2	153.3	68.1
DALYs (Disability-Adjusted Life Years)	Republic of South Africa	Male	Age-standardized	Edentulism	Rate	2005	198.2	288.4	117.0
DALYs (Disability-Adjusted Life Years)	Republic of South Africa	Female	Age-standardized	Edentulism	Rate	2005	190.3	275.6	111.9
DALYs (Disability-Adjusted Life Years)	Republic of South Africa	Both	Age-standardized	Edentulism	Rate	2005	193.9	281.6	114.2
DALYs (Disability-Adjusted Life Years)	United Kingdom of Great Britain and Northern Ireland	Male	Age-standardized	Edentulism	Rate	2000	143.2	199.5	92.8
DALYs (Disability-Adjusted Life Years)	United Kingdom of Great Britain and Northern Ireland	Female	Age-standardized	Edentulism	Rate	2000	161.5	227.2	101.9
DALYs (Disability-Adjusted Life Years)	United Kingdom of Great Britain and Northern Ireland	Both	Age-standardized	Edentulism	Rate	2000	153.4	214.9	97.6
DALYs (Disability-Adjusted Life Years)	United States of America	Male	Age-standardized	Edentulism	Rate	2001	131.9	179.3	83.9

DALYs (Disability-Adjusted Life Years)	United States of America	Female	Age-standardized	Edentulism	Rate	2001	139.6	189.5	89.6
DALYs (Disability-Adjusted Life Years)	United States of America	Both	Age-standardized	Edentulism	Rate	2001	135.9	184.6	87.0
DALYs (Disability-Adjusted Life Years)	Russian Federation	Male	Age-standardized	Edentulism	Rate	2001	158.8	213.6	102.3
DALYs (Disability-Adjusted Life Years)	Russian Federation	Female	Age-standardized	Edentulism	Rate	2001	169.3	230.8	108.7
DALYs (Disability-Adjusted Life Years)	Russian Federation	Both	Age-standardized	Edentulism	Rate	2001	166.6	226.2	107.6
DALYs (Disability-Adjusted Life Years)	Republic of Turkey	Male	Age-standardized	Edentulism	Rate	2000	219.5	299.3	140.3
DALYs (Disability-Adjusted Life Years)	Republic of Turkey	Female	Age-standardized	Edentulism	Rate	2000	192.6	264.8	122.8
DALYs (Disability-Adjusted Life Years)	Republic of Turkey	Both	Age-standardized	Edentulism	Rate	2000	204.6	279.7	131.1
DALYs (Disability-Adjusted Life Years)	Republic of India	Male	Age-standardized	Edentulism	Rate	2005	76.9	106.4	48.8
DALYs (Disability-Adjusted Life Years)	Republic of India	Female	Age-standardized	Edentulism	Rate	2005	82.4	114.1	52.3
DALYs (Disability-Adjusted Life Years)	Republic of India	Both	Age-standardized	Edentulism	Rate	2005	79.7	110.5	50.6
DALYs (Disability-Adjusted Life Years)	Republic of Turkey	Male	Age-standardized	Edentulism	Rate	2001	220.9	301.7	142.6
DALYs (Disability-Adjusted Life Years)	Republic of Turkey	Female	Age-standardized	Edentulism	Rate	2001	199.3	271.3	129.3
DALYs (Disability-Adjusted Life Years)	Republic of Turkey	Both	Age-standardized	Edentulism	Rate	2001	208.8	283.7	134.9
DALYs (Disability-Adjusted	United States of America	Male	Age-standardized	Edentulism	Rate	2002	127.6	173.1	81.3

Life Years)									
DALYs (Disability-Adjusted Life Years)	United States of America	Female	Age-standardized	Edentulism	Rate	2002	136.6	185.2	87.9
DALYs (Disability-Adjusted Life Years)	United States of America	Both	Age-standardized	Edentulism	Rate	2002	132.3	179.4	84.9
DALYs (Disability-Adjusted Life Years)	United Kingdom of Great Britain and Northern Ireland	Male	Age-standardized	Edentulism	Rate	2001	140.0	194.7	90.4
DALYs (Disability-Adjusted Life Years)	United Kingdom of Great Britain and Northern Ireland	Female	Age-standardized	Edentulism	Rate	2001	159.0	222.8	100.4
DALYs (Disability-Adjusted Life Years)	United Kingdom of Great Britain and Northern Ireland	Both	Age-standardized	Edentulism	Rate	2001	150.5	210.8	95.9
DALYs (Disability-Adjusted Life Years)	Republic of Indonesia	Male	Age-standardized	Edentulism	Rate	2000	74.7	106.0	49.3
DALYs (Disability-Adjusted Life Years)	Republic of Indonesia	Female	Age-standardized	Edentulism	Rate	2000	122.0	170.9	78.4
DALYs (Disability-Adjusted Life Years)	Republic of Indonesia	Both	Age-standardized	Edentulism	Rate	2000	99.6	140.2	64.8
DALYs (Disability-Adjusted Life Years)	European Union	Male	Age-standardized	Edentulism	Rate	2001	114.0	157.1	76.2
DALYs (Disability-Adjusted Life Years)	European Union	Female	Age-standardized	Edentulism	Rate	2001	144.4	199.7	97.2
DALYs (Disability-Adjusted Life Years)	European Union	Both	Age-standardized	Edentulism	Rate	2001	130.9	180.8	87.9
DALYs (Disability-Adjusted Life Years)	Australia	Male	Age-standardized	Edentulism	Rate	2001	130.9	180.3	84.8
DALYs (Disability-Adjusted Life Years)	Australia	Female	Age-standardized	Edentulism	Rate	2001	182.6	250.9	118.9
DALYs (Disability-Adjusted Life Years)	Australia	Both	Age-standardized	Edentulism	Rate	2001	158.3	218.2	103.1
DALYs	People's Republic	Male	Age-standardized	Edentulism	Rate	2001	68.0	96.5	45.4

(Disability-Adjusted Life Years)	of China								
DALYs (Disability-Adjusted Life Years)	People's Republic of China	Female	Age-standardized	Edentulism	Rate	2001	70.3	100.0	47.0
DALYs (Disability-Adjusted Life Years)	People's Republic of China	Both	Age-standardized	Edentulism	Rate	2001	68.9	97.8	45.9
DALYs (Disability-Adjusted Life Years)	French Republic	Male	Age-standardized	Edentulism	Rate	2000	85.1	119.3	54.9
DALYs (Disability-Adjusted Life Years)	French Republic	Female	Age-standardized	Edentulism	Rate	2000	118.8	166.7	74.3
DALYs (Disability-Adjusted Life Years)	French Republic	Both	Age-standardized	Edentulism	Rate	2000	103.8	145.4	65.9
DALYs (Disability-Adjusted Life Years)	People's Republic of China	Male	Age-standardized	Edentulism	Rate	2002	69.3	98.8	46.2
DALYs (Disability-Adjusted Life Years)	People's Republic of China	Female	Age-standardized	Edentulism	Rate	2002	73.9	104.8	49.2
DALYs (Disability-Adjusted Life Years)	People's Republic of China	Both	Age-standardized	Edentulism	Rate	2002	71.4	101.5	47.7
DALYs (Disability-Adjusted Life Years)	Canada	Male	Age-standardized	Edentulism	Rate	2001	77.6	105.6	50.3
DALYs (Disability-Adjusted Life Years)	Canada	Female	Age-standardized	Edentulism	Rate	2001	99.3	134.9	63.7
DALYs (Disability-Adjusted Life Years)	Canada	Both	Age-standardized	Edentulism	Rate	2001	89.2	120.0	57.7
DALYs (Disability-Adjusted Life Years)	Republic of Italy	Male	Age-standardized	Edentulism	Rate	2001	95.9	135.4	60.6
DALYs (Disability-Adjusted Life Years)	Republic of Italy	Female	Age-standardized	Edentulism	Rate	2001	119.3	168.7	75.7
DALYs (Disability-Adjusted Life Years)	Republic of Italy	Both	Age-standardized	Edentulism	Rate	2001	108.6	153.9	68.7

DALYs (Disability-Adjusted Life Years)	Republic of Korea	Male	Age-standardized	Edentulism	Rate	2006	36.7	50.7	25.1
DALYs (Disability-Adjusted Life Years)	Republic of Korea	Female	Age-standardized	Edentulism	Rate	2006	35.7	48.9	24.3
DALYs (Disability-Adjusted Life Years)	Republic of Korea	Both	Age-standardized	Edentulism	Rate	2006	36.7	50.7	25.1
DALYs (Disability-Adjusted Life Years)	Japan	Male	Age-standardized	Edentulism	Rate	2001	129.5	182.0	82.4
DALYs (Disability-Adjusted Life Years)	Japan	Female	Age-standardized	Edentulism	Rate	2001	62.9	89.3	42.2
DALYs (Disability-Adjusted Life Years)	Japan	Both	Age-standardized	Edentulism	Rate	2001	93.8	132.7	62.6
DALYs (Disability-Adjusted Life Years)	Argentine Republic	Male	Age-standardized	Edentulism	Rate	2000	89.8	125.1	58.2
DALYs (Disability-Adjusted Life Years)	Argentine Republic	Female	Age-standardized	Edentulism	Rate	2000	144.9	204.9	91.0
DALYs (Disability-Adjusted Life Years)	Argentine Republic	Both	Age-standardized	Edentulism	Rate	2000	120.8	169.8	76.1
DALYs (Disability-Adjusted Life Years)	Federal Republic of Germany	Male	Age-standardized	Edentulism	Rate	2006	91.8	127.5	61.8
DALYs (Disability-Adjusted Life Years)	Federal Republic of Germany	Female	Age-standardized	Edentulism	Rate	2006	113.2	155.8	77.9
DALYs (Disability-Adjusted Life Years)	Federal Republic of Germany	Both	Age-standardized	Edentulism	Rate	2006	102.6	141.3	70.1
DALYs (Disability-Adjusted Life Years)	Federative Republic of Brazil	Male	Age-standardized	Edentulism	Rate	2006	242.8	337.3	164.0
DALYs (Disability-Adjusted Life Years)	Federative Republic of Brazil	Female	Age-standardized	Edentulism	Rate	2006	343.8	469.8	227.0
DALYs (Disability-Adjusted	Federative Republic of Brazil	Both	Age-standardized	Edentulism	Rate	2006	296.4	407.6	199.2

Life Years)									
DALYs (Disability-Adjusted Life Years)	Argentine Republic	Male	Age-standardized	Edentulism	Rate	2001	89.9	126.4	58.0
DALYs (Disability-Adjusted Life Years)	Argentine Republic	Female	Age-standardized	Edentulism	Rate	2001	145.0	205.3	90.9
DALYs (Disability-Adjusted Life Years)	Argentine Republic	Both	Age-standardized	Edentulism	Rate	2001	120.9	170.5	76.7
DALYs (Disability-Adjusted Life Years)	Kingdom of Saudi Arabia	Male	Age-standardized	Edentulism	Rate	2006	162.5	222.3	101.4
DALYs (Disability-Adjusted Life Years)	Kingdom of Saudi Arabia	Female	Age-standardized	Edentulism	Rate	2006	111.1	151.4	71.8
DALYs (Disability-Adjusted Life Years)	Kingdom of Saudi Arabia	Both	Age-standardized	Edentulism	Rate	2006	140.3	191.3	88.6
DALYs (Disability-Adjusted Life Years)	French Republic	Male	Age-standardized	Edentulism	Rate	2001	85.8	119.1	55.3
DALYs (Disability-Adjusted Life Years)	French Republic	Female	Age-standardized	Edentulism	Rate	2001	119.6	168.8	76.7
DALYs (Disability-Adjusted Life Years)	French Republic	Both	Age-standardized	Edentulism	Rate	2001	104.6	145.4	67.3
DALYs (Disability-Adjusted Life Years)	Republic of South Africa	Male	Age-standardized	Edentulism	Rate	2006	187.4	273.3	111.7
DALYs (Disability-Adjusted Life Years)	Republic of South Africa	Female	Age-standardized	Edentulism	Rate	2006	178.6	258.7	106.4
DALYs (Disability-Adjusted Life Years)	Republic of South Africa	Both	Age-standardized	Edentulism	Rate	2006	182.5	265.2	108.8
DALYs (Disability-Adjusted Life Years)	Japan	Male	Age-standardized	Edentulism	Rate	2002	134.9	189.0	86.4
DALYs (Disability-Adjusted Life Years)	Japan	Female	Age-standardized	Edentulism	Rate	2002	65.3	92.6	44.0
DALYs	Japan	Both	Age-standardized	Edentulism	Rate	2002	98.0	138.7	65.4

(Disability-Adjusted Life Years)									
DALYs (Disability-Adjusted Life Years)	Canada	Male	Age-standardized	Edentulism	Rate	2002	85.9	116.6	56.8
DALYs (Disability-Adjusted Life Years)	Canada	Female	Age-standardized	Edentulism	Rate	2002	103.3	140.2	68.9
DALYs (Disability-Adjusted Life Years)	Canada	Both	Age-standardized	Edentulism	Rate	2002	95.2	129.9	63.6
DALYs (Disability-Adjusted Life Years)	Republic of India	Male	Age-standardized	Edentulism	Rate	2006	88.8	122.3	56.4
DALYs (Disability-Adjusted Life Years)	Republic of India	Female	Age-standardized	Edentulism	Rate	2006	94.2	129.5	60.0
DALYs (Disability-Adjusted Life Years)	Republic of India	Both	Age-standardized	Edentulism	Rate	2006	91.6	125.7	58.3
DALYs (Disability-Adjusted Life Years)	Republic of Indonesia	Male	Age-standardized	Edentulism	Rate	2001	75.9	107.3	50.2
DALYs (Disability-Adjusted Life Years)	Republic of Indonesia	Female	Age-standardized	Edentulism	Rate	2001	124.3	172.1	81.8
DALYs (Disability-Adjusted Life Years)	Republic of Indonesia	Both	Age-standardized	Edentulism	Rate	2001	101.4	140.6	66.7
DALYs (Disability-Adjusted Life Years)	United Kingdom of Great Britain and Northern Ireland	Male	Age-standardized	Edentulism	Rate	2002	131.9	183.9	85.2
DALYs (Disability-Adjusted Life Years)	United Kingdom of Great Britain and Northern Ireland	Female	Age-standardized	Edentulism	Rate	2002	152.6	214.1	96.4
DALYs (Disability-Adjusted Life Years)	United Kingdom of Great Britain and Northern Ireland	Both	Age-standardized	Edentulism	Rate	2002	143.2	201.3	91.4
DALYs (Disability-Adjusted Life Years)	French Republic	Male	Age-standardized	Edentulism	Rate	2002	86.4	119.9	55.6
DALYs (Disability-Adjusted Life Years)	French Republic	Female	Age-standardized	Edentulism	Rate	2002	119.5	167.7	78.4

DALYs (Disability-Adjusted Life Years)	French Republic	Both	Age-standardized	Edentulism	Rate	2002	104.9	146.4	68.3
DALYs (Disability-Adjusted Life Years)	Republic of Indonesia	Male	Age-standardized	Edentulism	Rate	2002	78.9	110.4	51.9
DALYs (Disability-Adjusted Life Years)	Republic of Indonesia	Female	Age-standardized	Edentulism	Rate	2002	129.7	177.5	86.8
DALYs (Disability-Adjusted Life Years)	Republic of Indonesia	Both	Age-standardized	Edentulism	Rate	2002	105.6	145.9	69.7
DALYs (Disability-Adjusted Life Years)	Australia	Male	Age-standardized	Edentulism	Rate	2002	122.2	167.8	81.3
DALYs (Disability-Adjusted Life Years)	Australia	Female	Age-standardized	Edentulism	Rate	2002	169.2	232.4	111.9
DALYs (Disability-Adjusted Life Years)	Australia	Both	Age-standardized	Edentulism	Rate	2002	147.1	201.9	97.4
DALYs (Disability-Adjusted Life Years)	United Mexican States	Male	Age-standardized	Edentulism	Rate	2002	123.0	166.2	79.3
DALYs (Disability-Adjusted Life Years)	United Mexican States	Female	Age-standardized	Edentulism	Rate	2002	189.8	255.3	125.5
DALYs (Disability-Adjusted Life Years)	United Mexican States	Both	Age-standardized	Edentulism	Rate	2002	158.2	213.3	103.4
DALYs (Disability-Adjusted Life Years)	Russian Federation	Male	Age-standardized	Edentulism	Rate	2002	156.2	210.3	100.4
DALYs (Disability-Adjusted Life Years)	Russian Federation	Female	Age-standardized	Edentulism	Rate	2002	166.4	226.0	107.2
DALYs (Disability-Adjusted Life Years)	Russian Federation	Both	Age-standardized	Edentulism	Rate	2002	163.9	222.4	105.7
DALYs (Disability-Adjusted Life Years)	Argentine Republic	Male	Age-standardized	Edentulism	Rate	2002	90.2	126.6	57.5
DALYs (Disability-Adjusted	Argentine Republic	Female	Age-standardized	Edentulism	Rate	2002	145.1	208.0	91.3

Life Years)									
DALYs (Disability-Adjusted Life Years)	Argentine Republic	Both	Age-standardized	Edentulism	Rate	2002	121.0	171.9	76.6
DALYs (Disability-Adjusted Life Years)	Republic of Italy	Male	Age-standardized	Edentulism	Rate	2002	100.9	142.7	64.5
DALYs (Disability-Adjusted Life Years)	Republic of Italy	Female	Age-standardized	Edentulism	Rate	2002	117.5	165.4	74.5
DALYs (Disability-Adjusted Life Years)	Republic of Italy	Both	Age-standardized	Edentulism	Rate	2002	109.7	155.0	69.7
DALYs (Disability-Adjusted Life Years)	Republic of Korea	Male	Age-standardized	Edentulism	Rate	2007	36.3	49.8	24.6
DALYs (Disability-Adjusted Life Years)	Republic of Korea	Female	Age-standardized	Edentulism	Rate	2007	35.3	49.0	24.2
DALYs (Disability-Adjusted Life Years)	Republic of Korea	Both	Age-standardized	Edentulism	Rate	2007	36.3	49.9	24.7
DALYs (Disability-Adjusted Life Years)	European Union	Male	Age-standardized	Edentulism	Rate	2003	117.1	161.9	78.9
DALYs (Disability-Adjusted Life Years)	European Union	Female	Age-standardized	Edentulism	Rate	2003	144.2	199.9	97.0
DALYs (Disability-Adjusted Life Years)	European Union	Both	Age-standardized	Edentulism	Rate	2003	132.0	182.9	89.1
DALYs (Disability-Adjusted Life Years)	Argentine Republic	Male	Age-standardized	Edentulism	Rate	2003	90.3	126.5	56.8
DALYs (Disability-Adjusted Life Years)	Argentine Republic	Female	Age-standardized	Edentulism	Rate	2003	145.2	209.6	91.7
DALYs (Disability-Adjusted Life Years)	Argentine Republic	Both	Age-standardized	Edentulism	Rate	2003	121.2	172.1	76.7
DALYs (Disability-Adjusted Life Years)	European Union	Male	Age-standardized	Edentulism	Rate	2002	115.5	159.1	77.8
DALYs	European Union	Female	Age-standardized	Edentulism	Rate	2002	144.4	200.2	97.5

(Disability-Adjusted Life Years)									
DALYs (Disability-Adjusted Life Years)	European Union	Both	Age-standardized	Edentulism	Rate	2002	131.5	182.0	88.9
DALYs (Disability-Adjusted Life Years)	Australia	Male	Age-standardized	Edentulism	Rate	2003	111.9	152.6	75.7
DALYs (Disability-Adjusted Life Years)	Australia	Female	Age-standardized	Edentulism	Rate	2003	153.3	208.0	103.2
DALYs (Disability-Adjusted Life Years)	Australia	Both	Age-standardized	Edentulism	Rate	2003	133.9	182.1	90.4
DALYs (Disability-Adjusted Life Years)	Federal Republic of Germany	Male	Age-standardized	Edentulism	Rate	2007	81.7	111.5	55.5
DALYs (Disability-Adjusted Life Years)	Federal Republic of Germany	Female	Age-standardized	Edentulism	Rate	2007	102.4	142.9	70.6
DALYs (Disability-Adjusted Life Years)	Federal Republic of Germany	Both	Age-standardized	Edentulism	Rate	2007	92.4	127.1	63.7
DALYs (Disability-Adjusted Life Years)	Federative Republic of Brazil	Male	Age-standardized	Edentulism	Rate	2007	232.6	322.2	158.4
DALYs (Disability-Adjusted Life Years)	Federative Republic of Brazil	Female	Age-standardized	Edentulism	Rate	2007	322.6	441.2	213.0
DALYs (Disability-Adjusted Life Years)	Federative Republic of Brazil	Both	Age-standardized	Edentulism	Rate	2007	280.5	385.4	188.6
DALYs (Disability-Adjusted Life Years)	Republic of Turkey	Male	Age-standardized	Edentulism	Rate	2002	224.9	309.4	147.7
DALYs (Disability-Adjusted Life Years)	Republic of Turkey	Female	Age-standardized	Edentulism	Rate	2002	215.5	300.4	142.2
DALYs (Disability-Adjusted Life Years)	Republic of Turkey	Both	Age-standardized	Edentulism	Rate	2002	219.2	304.0	144.2
DALYs (Disability-Adjusted Life Years)	Kingdom of Saudi Arabia	Male	Age-standardized	Edentulism	Rate	2007	161.3	220.7	100.8

DALYs (Disability-Adjusted Life Years)	Kingdom of Saudi Arabia	Female	Age-standardized	Edentulism	Rate	2007	110.3	150.6	71.0
DALYs (Disability-Adjusted Life Years)	Kingdom of Saudi Arabia	Both	Age-standardized	Edentulism	Rate	2007	139.4	191.2	87.9
DALYs (Disability-Adjusted Life Years)	Republic of Turkey	Male	Age-standardized	Edentulism	Rate	2003	229.8	317.7	155.0
DALYs (Disability-Adjusted Life Years)	Republic of Turkey	Female	Age-standardized	Edentulism	Rate	2003	235.2	328.6	157.5
DALYs (Disability-Adjusted Life Years)	Republic of Turkey	Both	Age-standardized	Edentulism	Rate	2003	231.9	323.3	156.3
DALYs (Disability-Adjusted Life Years)	Republic of South Africa	Male	Age-standardized	Edentulism	Rate	2007	165.9	239.4	100.1
DALYs (Disability-Adjusted Life Years)	Republic of South Africa	Female	Age-standardized	Edentulism	Rate	2007	158.9	230.1	96.3
DALYs (Disability-Adjusted Life Years)	Republic of South Africa	Both	Age-standardized	Edentulism	Rate	2007	162.0	234.2	98.0
DALYs (Disability-Adjusted Life Years)	Japan	Male	Age-standardized	Edentulism	Rate	2003	140.5	196.0	91.1
DALYs (Disability-Adjusted Life Years)	Japan	Female	Age-standardized	Edentulism	Rate	2003	68.0	96.4	45.5
DALYs (Disability-Adjusted Life Years)	Japan	Both	Age-standardized	Edentulism	Rate	2003	102.5	144.7	68.1
DALYs (Disability-Adjusted Life Years)	Republic of Italy	Male	Age-standardized	Edentulism	Rate	2003	106.8	151.6	69.2
DALYs (Disability-Adjusted Life Years)	Republic of Italy	Female	Age-standardized	Edentulism	Rate	2003	115.1	161.9	73.2
DALYs (Disability-Adjusted Life Years)	Republic of Italy	Both	Age-standardized	Edentulism	Rate	2003	110.9	156.8	71.2
DALYs (Disability-Adjusted	United States of America	Male	Age-standardized	Edentulism	Rate	2004	117.2	159.8	75.1

Life Years)									
DALYs (Disability-Adjusted Life Years)	United States of America	Female	Age-standardized	Edentulism	Rate	2004	129.5	174.9	83.8
DALYs (Disability-Adjusted Life Years)	United States of America	Both	Age-standardized	Edentulism	Rate	2004	123.9	167.6	79.8
DALYs (Disability-Adjusted Life Years)	Republic of India	Male	Age-standardized	Edentulism	Rate	2007	113.8	156.6	72.2
DALYs (Disability-Adjusted Life Years)	Republic of India	Female	Age-standardized	Edentulism	Rate	2007	118.5	162.1	75.6
DALYs (Disability-Adjusted Life Years)	Republic of India	Both	Age-standardized	Edentulism	Rate	2007	116.3	159.1	73.9
DALYs (Disability-Adjusted Life Years)	Canada	Male	Age-standardized	Edentulism	Rate	2003	95.9	130.4	64.9
DALYs (Disability-Adjusted Life Years)	Canada	Female	Age-standardized	Edentulism	Rate	2003	108.2	149.5	73.5
DALYs (Disability-Adjusted Life Years)	Canada	Both	Age-standardized	Edentulism	Rate	2003	102.5	140.3	69.5
DALYs (Disability-Adjusted Life Years)	United States of America	Male	Age-standardized	Edentulism	Rate	2003	122.3	164.9	78.4
DALYs (Disability-Adjusted Life Years)	United States of America	Female	Age-standardized	Edentulism	Rate	2003	133.1	180.5	85.7
DALYs (Disability-Adjusted Life Years)	United States of America	Both	Age-standardized	Edentulism	Rate	2003	128.1	173.5	82.4
DALYs (Disability-Adjusted Life Years)	Russian Federation	Male	Age-standardized	Edentulism	Rate	2003	152.8	205.3	97.9
DALYs (Disability-Adjusted Life Years)	Russian Federation	Female	Age-standardized	Edentulism	Rate	2003	162.8	220.9	105.0
DALYs (Disability-Adjusted Life Years)	Russian Federation	Both	Age-standardized	Edentulism	Rate	2003	160.4	217.1	103.4
DALYs	People's Republic	Male	Age-standardized	Edentulism	Rate	2003	70.8	100.6	47.1

(Disability-Adjusted Life Years)	of China								
DALYs (Disability-Adjusted Life Years)	People's Republic of China	Female	Age-standardized	Edentulism	Rate	2003	78.4	111.0	51.9
DALYs (Disability-Adjusted Life Years)	People's Republic of China	Both	Age-standardized	Edentulism	Rate	2003	74.5	105.7	49.7
DALYs (Disability-Adjusted Life Years)	French Republic	Male	Age-standardized	Edentulism	Rate	2003	86.4	121.5	56.5
DALYs (Disability-Adjusted Life Years)	French Republic	Female	Age-standardized	Edentulism	Rate	2003	118.9	164.9	79.0
DALYs (Disability-Adjusted Life Years)	French Republic	Both	Age-standardized	Edentulism	Rate	2003	104.7	144.0	69.5
DALYs (Disability-Adjusted Life Years)	People's Republic of China	Male	Age-standardized	Edentulism	Rate	2004	72.1	102.0	47.9
DALYs (Disability-Adjusted Life Years)	People's Republic of China	Female	Age-standardized	Edentulism	Rate	2004	82.7	116.8	54.7
DALYs (Disability-Adjusted Life Years)	People's Republic of China	Both	Age-standardized	Edentulism	Rate	2004	77.4	109.7	51.6
DALYs (Disability-Adjusted Life Years)	Canada	Male	Age-standardized	Edentulism	Rate	2004	104.2	139.6	71.2
DALYs (Disability-Adjusted Life Years)	Canada	Female	Age-standardized	Edentulism	Rate	2004	112.3	157.5	77.2
DALYs (Disability-Adjusted Life Years)	Canada	Both	Age-standardized	Edentulism	Rate	2004	108.6	148.8	74.5
DALYs (Disability-Adjusted Life Years)	Japan	Male	Age-standardized	Edentulism	Rate	2004	144.8	202.4	94.5
DALYs (Disability-Adjusted Life Years)	Japan	Female	Age-standardized	Edentulism	Rate	2004	70.0	99.2	46.5
DALYs (Disability-Adjusted Life Years)	Japan	Both	Age-standardized	Edentulism	Rate	2004	106.0	149.1	70.3

DALYs (Disability-Adjusted Life Years)	United Mexican States	Male	Age-standardized	Edentulism	Rate	2003	123.2	166.6	79.3
DALYs (Disability-Adjusted Life Years)	United Mexican States	Female	Age-standardized	Edentulism	Rate	2003	191.2	256.6	128.5
DALYs (Disability-Adjusted Life Years)	United Mexican States	Both	Age-standardized	Edentulism	Rate	2003	159.0	214.0	105.2
DALYs (Disability-Adjusted Life Years)	Republic of Korea	Male	Age-standardized	Edentulism	Rate	2008	36.1	50.1	24.9
DALYs (Disability-Adjusted Life Years)	Republic of Korea	Female	Age-standardized	Edentulism	Rate	2008	35.0	48.5	23.9
DALYs (Disability-Adjusted Life Years)	Republic of Korea	Both	Age-standardized	Edentulism	Rate	2008	36.0	50.2	24.8
DALYs (Disability-Adjusted Life Years)	French Republic	Male	Age-standardized	Edentulism	Rate	2004	86.1	120.2	56.6
DALYs (Disability-Adjusted Life Years)	French Republic	Female	Age-standardized	Edentulism	Rate	2004	117.8	164.5	79.5
DALYs (Disability-Adjusted Life Years)	French Republic	Both	Age-standardized	Edentulism	Rate	2004	104.1	144.5	69.8
DALYs (Disability-Adjusted Life Years)	Federal Republic of Germany	Male	Age-standardized	Edentulism	Rate	2008	70.7	98.2	48.9
DALYs (Disability-Adjusted Life Years)	Federal Republic of Germany	Female	Age-standardized	Edentulism	Rate	2008	91.0	127.0	62.6
DALYs (Disability-Adjusted Life Years)	Federal Republic of Germany	Both	Age-standardized	Edentulism	Rate	2008	81.4	112.8	56.4
DALYs (Disability-Adjusted Life Years)	Federative Republic of Brazil	Male	Age-standardized	Edentulism	Rate	2008	220.5	305.0	153.6
DALYs (Disability-Adjusted Life Years)	Federative Republic of Brazil	Female	Age-standardized	Edentulism	Rate	2008	297.1	407.0	196.6
DALYs (Disability-Adjusted	Federative Republic of Brazil	Both	Age-standardized	Edentulism	Rate	2008	261.4	359.2	175.6

Life Years)									
DALYs (Disability-Adjusted Life Years)	United Kingdom of Great Britain and Northern Ireland	Male	Age-standardized	Edentulism	Rate	2003	121.3	169.9	77.3
DALYs (Disability-Adjusted Life Years)	United Kingdom of Great Britain and Northern Ireland	Female	Age-standardized	Edentulism	Rate	2003	144.0	202.8	91.0
DALYs (Disability-Adjusted Life Years)	United Kingdom of Great Britain and Northern Ireland	Both	Age-standardized	Edentulism	Rate	2003	133.5	188.0	84.4
DALYs (Disability-Adjusted Life Years)	Kingdom of Saudi Arabia	Male	Age-standardized	Edentulism	Rate	2008	159.7	218.2	99.2
DALYs (Disability-Adjusted Life Years)	Kingdom of Saudi Arabia	Female	Age-standardized	Edentulism	Rate	2008	109.7	149.0	70.5
DALYs (Disability-Adjusted Life Years)	Kingdom of Saudi Arabia	Both	Age-standardized	Edentulism	Rate	2008	138.3	188.3	87.1
DALYs (Disability-Adjusted Life Years)	Republic of South Africa	Male	Age-standardized	Edentulism	Rate	2008	141.4	202.6	85.7
DALYs (Disability-Adjusted Life Years)	Republic of South Africa	Female	Age-standardized	Edentulism	Rate	2008	137.2	196.4	84.4
DALYs (Disability-Adjusted Life Years)	Republic of South Africa	Both	Age-standardized	Edentulism	Rate	2008	139.0	198.9	85.0
DALYs (Disability-Adjusted Life Years)	United Kingdom of Great Britain and Northern Ireland	Male	Age-standardized	Edentulism	Rate	2004	110.5	155.3	70.4
DALYs (Disability-Adjusted Life Years)	United Kingdom of Great Britain and Northern Ireland	Female	Age-standardized	Edentulism	Rate	2004	135.0	190.9	84.9
DALYs (Disability-Adjusted Life Years)	United Kingdom of Great Britain and Northern Ireland	Both	Age-standardized	Edentulism	Rate	2004	123.5	174.4	78.0
DALYs (Disability-Adjusted Life Years)	Australia	Male	Age-standardized	Edentulism	Rate	2004	103.3	142.6	70.5
DALYs (Disability-Adjusted Life Years)	Australia	Female	Age-standardized	Edentulism	Rate	2004	139.9	191.0	96.1
DALYs	Australia	Both	Age-standardized	Edentulism	Rate	2004	122.7	167.2	84.2

(Disability-Adjusted Life Years)									
DALYs (Disability-Adjusted Life Years)	Republic of India	Male	Age-standardized	Edentulism	Rate	2008	142.9	197.0	91.5
DALYs (Disability-Adjusted Life Years)	Republic of India	Female	Age-standardized	Edentulism	Rate	2008	146.7	200.0	94.2
DALYs (Disability-Adjusted Life Years)	Republic of India	Both	Age-standardized	Edentulism	Rate	2008	145.0	198.6	93.0
DALYs (Disability-Adjusted Life Years)	Republic of Indonesia	Male	Age-standardized	Edentulism	Rate	2004	85.5	118.4	56.2
DALYs (Disability-Adjusted Life Years)	Republic of Indonesia	Female	Age-standardized	Edentulism	Rate	2004	141.6	189.5	97.0
DALYs (Disability-Adjusted Life Years)	Republic of Indonesia	Both	Age-standardized	Edentulism	Rate	2004	114.8	155.4	78.9
DALYs (Disability-Adjusted Life Years)	Argentine Republic	Male	Age-standardized	Edentulism	Rate	2005	90.7	126.5	57.6
DALYs (Disability-Adjusted Life Years)	Argentine Republic	Female	Age-standardized	Edentulism	Rate	2005	145.4	209.5	92.3
DALYs (Disability-Adjusted Life Years)	Argentine Republic	Both	Age-standardized	Edentulism	Rate	2005	121.4	173.9	77.3
DALYs (Disability-Adjusted Life Years)	Republic of Indonesia	Male	Age-standardized	Edentulism	Rate	2003	82.5	115.2	54.5
DALYs (Disability-Adjusted Life Years)	Republic of Indonesia	Female	Age-standardized	Edentulism	Rate	2003	136.2	183.3	93.4
DALYs (Disability-Adjusted Life Years)	Republic of Indonesia	Both	Age-standardized	Edentulism	Rate	2003	110.6	151.0	74.7
DALYs (Disability-Adjusted Life Years)	European Union	Male	Age-standardized	Edentulism	Rate	2004	118.1	163.1	79.6
DALYs (Disability-Adjusted Life Years)	European Union	Female	Age-standardized	Edentulism	Rate	2004	143.5	198.5	96.5

DALYs (Disability-Adjusted Life Years)	European Union	Both	Age-standardized	Edentulism	Rate	2004	132.0	182.8	88.8
DALYs (Disability-Adjusted Life Years)	European Union	Male	Age-standardized	Edentulism	Rate	2005	117.7	162.2	79.2
DALYs (Disability-Adjusted Life Years)	European Union	Female	Age-standardized	Edentulism	Rate	2005	142.1	196.9	95.9
DALYs (Disability-Adjusted Life Years)	European Union	Both	Age-standardized	Edentulism	Rate	2005	131.0	181.1	88.3
DALYs (Disability-Adjusted Life Years)	Australia	Male	Age-standardized	Edentulism	Rate	2005	99.8	137.2	67.8
DALYs (Disability-Adjusted Life Years)	Australia	Female	Age-standardized	Edentulism	Rate	2005	134.3	183.0	92.5
DALYs (Disability-Adjusted Life Years)	Australia	Both	Age-standardized	Edentulism	Rate	2005	118.1	161.4	80.2
DALYs (Disability-Adjusted Life Years)	United Mexican States	Male	Age-standardized	Edentulism	Rate	2004	123.2	166.1	79.2
DALYs (Disability-Adjusted Life Years)	United Mexican States	Female	Age-standardized	Edentulism	Rate	2004	192.2	256.7	130.1
DALYs (Disability-Adjusted Life Years)	United Mexican States	Both	Age-standardized	Edentulism	Rate	2004	159.6	213.4	106.9
DALYs (Disability-Adjusted Life Years)	Republic of Turkey	Male	Age-standardized	Edentulism	Rate	2005	235.1	330.7	158.3
DALYs (Disability-Adjusted Life Years)	Republic of Turkey	Female	Age-standardized	Edentulism	Rate	2005	260.1	365.4	175.2
DALYs (Disability-Adjusted Life Years)	Republic of Turkey	Both	Age-standardized	Edentulism	Rate	2005	247.7	347.6	167.7
DALYs (Disability-Adjusted Life Years)	United States of America	Male	Age-standardized	Edentulism	Rate	2005	113.6	154.7	72.4
DALYs (Disability-Adjusted	United States of America	Female	Age-standardized	Edentulism	Rate	2005	126.5	171.2	81.7

Life Years)									
DALYs (Disability-Adjusted Life Years)	United States of America	Both	Age-standardized	Edentulism	Rate	2005	120.6	163.4	77.5
DALYs (Disability-Adjusted Life Years)	Russian Federation	Male	Age-standardized	Edentulism	Rate	2005	146.5	196.6	93.5
DALYs (Disability-Adjusted Life Years)	Russian Federation	Female	Age-standardized	Edentulism	Rate	2005	158.3	214.5	102.4
DALYs (Disability-Adjusted Life Years)	Russian Federation	Both	Age-standardized	Edentulism	Rate	2005	155.2	209.9	99.9
DALYs (Disability-Adjusted Life Years)	Argentine Republic	Male	Age-standardized	Edentulism	Rate	2004	90.5	128.1	57.0
DALYs (Disability-Adjusted Life Years)	Argentine Republic	Female	Age-standardized	Edentulism	Rate	2004	145.5	209.2	91.6
DALYs (Disability-Adjusted Life Years)	Argentine Republic	Both	Age-standardized	Edentulism	Rate	2004	121.4	173.9	76.7
DALYs (Disability-Adjusted Life Years)	People's Republic of China	Male	Age-standardized	Edentulism	Rate	2005	72.9	102.9	48.5
DALYs (Disability-Adjusted Life Years)	People's Republic of China	Female	Age-standardized	Edentulism	Rate	2005	85.4	120.5	56.3
DALYs (Disability-Adjusted Life Years)	People's Republic of China	Both	Age-standardized	Edentulism	Rate	2005	79.2	112.2	52.6
DALYs (Disability-Adjusted Life Years)	Republic of Korea	Male	Age-standardized	Edentulism	Rate	2009	35.9	49.8	24.3
DALYs (Disability-Adjusted Life Years)	Republic of Korea	Female	Age-standardized	Edentulism	Rate	2009	34.8	47.8	24.0
DALYs (Disability-Adjusted Life Years)	Republic of Korea	Both	Age-standardized	Edentulism	Rate	2009	35.8	49.5	24.4
DALYs (Disability-Adjusted Life Years)	Federal Republic of Germany	Male	Age-standardized	Edentulism	Rate	2009	61.9	85.0	43.1
DALYs	Federal Republic	Female	Age-standardized	Edentulism	Rate	2009	81.9	113.7	55.9

(Disability-Adjusted Life Years)	of Germany								
DALYs (Disability-Adjusted Life Years)	Federal Republic of Germany	Both	Age-standardized	Edentulism	Rate	2009	72.6	100.7	50.1
DALYs (Disability-Adjusted Life Years)	Japan	Male	Age-standardized	Edentulism	Rate	2005	146.5	205.0	96.1
DALYs (Disability-Adjusted Life Years)	Japan	Female	Age-standardized	Edentulism	Rate	2005	70.9	100.8	47.0
DALYs (Disability-Adjusted Life Years)	Japan	Both	Age-standardized	Edentulism	Rate	2005	107.4	151.0	70.9
DALYs (Disability-Adjusted Life Years)	Federative Republic of Brazil	Male	Age-standardized	Edentulism	Rate	2009	210.1	291.0	144.1
DALYs (Disability-Adjusted Life Years)	Federative Republic of Brazil	Female	Age-standardized	Edentulism	Rate	2009	275.3	378.4	183.5
DALYs (Disability-Adjusted Life Years)	Federative Republic of Brazil	Both	Age-standardized	Edentulism	Rate	2009	245.0	337.8	165.4
DALYs (Disability-Adjusted Life Years)	Russian Federation	Male	Age-standardized	Edentulism	Rate	2004	149.3	200.4	95.4
DALYs (Disability-Adjusted Life Years)	Russian Federation	Female	Age-standardized	Edentulism	Rate	2004	159.7	216.3	102.9
DALYs (Disability-Adjusted Life Years)	Russian Federation	Both	Age-standardized	Edentulism	Rate	2004	157.2	212.5	101.2
DALYs (Disability-Adjusted Life Years)	Kingdom of Saudi Arabia	Male	Age-standardized	Edentulism	Rate	2009	158.4	217.0	98.8
DALYs (Disability-Adjusted Life Years)	Kingdom of Saudi Arabia	Female	Age-standardized	Edentulism	Rate	2009	108.9	148.4	71.4
DALYs (Disability-Adjusted Life Years)	Kingdom of Saudi Arabia	Both	Age-standardized	Edentulism	Rate	2009	137.3	188.1	87.2
DALYs (Disability-Adjusted Life Years)	Republic of Italy	Male	Age-standardized	Edentulism	Rate	2005	113.8	161.9	74.6

DALYs (Disability-Adjusted Life Years)	Republic of Italy	Female	Age-standardized	Edentulism	Rate	2005	111.7	155.8	71.6
DALYs (Disability-Adjusted Life Years)	Republic of Italy	Both	Age-standardized	Edentulism	Rate	2005	112.0	157.8	72.6
DALYs (Disability-Adjusted Life Years)	Republic of South Africa	Male	Age-standardized	Edentulism	Rate	2009	121.2	172.4	73.5
DALYs (Disability-Adjusted Life Years)	Republic of South Africa	Female	Age-standardized	Edentulism	Rate	2009	119.7	169.6	73.4
DALYs (Disability-Adjusted Life Years)	Republic of South Africa	Both	Age-standardized	Edentulism	Rate	2009	120.3	170.5	73.4
DALYs (Disability-Adjusted Life Years)	French Republic	Male	Age-standardized	Edentulism	Rate	2005	85.2	118.3	55.9
DALYs (Disability-Adjusted Life Years)	French Republic	Female	Age-standardized	Edentulism	Rate	2005	116.4	160.7	79.0
DALYs (Disability-Adjusted Life Years)	French Republic	Both	Age-standardized	Edentulism	Rate	2005	102.9	140.9	69.0
DALYs (Disability-Adjusted Life Years)	Republic of Italy	Male	Age-standardized	Edentulism	Rate	2004	111.7	158.5	72.8
DALYs (Disability-Adjusted Life Years)	Republic of Italy	Female	Age-standardized	Edentulism	Rate	2004	113.1	158.2	72.5
DALYs (Disability-Adjusted Life Years)	Republic of Italy	Both	Age-standardized	Edentulism	Rate	2004	111.9	157.6	72.2
DALYs (Disability-Adjusted Life Years)	Republic of India	Male	Age-standardized	Edentulism	Rate	2009	167.1	230.8	106.8
DALYs (Disability-Adjusted Life Years)	Republic of India	Female	Age-standardized	Edentulism	Rate	2009	170.3	232.2	109.8
DALYs (Disability-Adjusted Life Years)	Republic of India	Both	Age-standardized	Edentulism	Rate	2009	168.9	231.5	108.5
DALYs (Disability-Adjusted	Republic of Turkey	Male	Age-standardized	Edentulism	Rate	2004	233.7	326.6	156.0

Life Years)									
DALYs (Disability-Adjusted Life Years)	Republic of Turkey	Female	Age-standardized	Edentulism	Rate	2004	252.1	354.6	168.5
DALYs (Disability-Adjusted Life Years)	Republic of Turkey	Both	Age-standardized	Edentulism	Rate	2004	242.7	338.9	162.3
DALYs (Disability-Adjusted Life Years)	Japan	Male	Age-standardized	Edentulism	Rate	2006	139.8	195.2	91.5
DALYs (Disability-Adjusted Life Years)	Japan	Female	Age-standardized	Edentulism	Rate	2006	70.2	99.0	46.7
DALYs (Disability-Adjusted Life Years)	Japan	Both	Age-standardized	Edentulism	Rate	2006	103.9	145.7	68.7
DALYs (Disability-Adjusted Life Years)	Canada	Male	Age-standardized	Edentulism	Rate	2005	107.6	146.6	73.6
DALYs (Disability-Adjusted Life Years)	Canada	Female	Age-standardized	Edentulism	Rate	2005	114.1	160.3	78.8
DALYs (Disability-Adjusted Life Years)	Canada	Both	Age-standardized	Edentulism	Rate	2005	111.1	154.0	76.0
DALYs (Disability-Adjusted Life Years)	People's Republic of China	Male	Age-standardized	Edentulism	Rate	2006	73.5	104.0	48.9
DALYs (Disability-Adjusted Life Years)	People's Republic of China	Female	Age-standardized	Edentulism	Rate	2006	87.0	122.2	57.3
DALYs (Disability-Adjusted Life Years)	People's Republic of China	Both	Age-standardized	Edentulism	Rate	2006	80.3	113.5	53.4
DALYs (Disability-Adjusted Life Years)	United Mexican States	Male	Age-standardized	Edentulism	Rate	2005	123.1	166.4	79.2
DALYs (Disability-Adjusted Life Years)	United Mexican States	Female	Age-standardized	Edentulism	Rate	2005	192.6	256.0	131.1
DALYs (Disability-Adjusted Life Years)	United Mexican States	Both	Age-standardized	Edentulism	Rate	2005	159.8	213.1	108.2
DALYs	Republic of Korea	Male	Age-standardized	Edentulism	Rate	2010	35.9	49.6	24.5

(Disability-Adjusted Life Years)									
DALYs (Disability-Adjusted Life Years)	Republic of Korea	Female	Age-standardized	Edentulism	Rate	2010	34.9	48.5	24.2
DALYs (Disability-Adjusted Life Years)	Republic of Korea	Both	Age-standardized	Edentulism	Rate	2010	35.8	49.6	24.5
DALYs (Disability-Adjusted Life Years)	Federal Republic of Germany	Male	Age-standardized	Edentulism	Rate	2010	58.2	81.1	40.3
DALYs (Disability-Adjusted Life Years)	Federal Republic of Germany	Female	Age-standardized	Edentulism	Rate	2010	78.1	107.8	52.9
DALYs (Disability-Adjusted Life Years)	Federal Republic of Germany	Both	Age-standardized	Edentulism	Rate	2010	69.0	95.5	47.4
DALYs (Disability-Adjusted Life Years)	Canada	Male	Age-standardized	Edentulism	Rate	2006	107.5	149.3	73.6
DALYs (Disability-Adjusted Life Years)	Canada	Female	Age-standardized	Edentulism	Rate	2006	114.0	157.0	78.4
DALYs (Disability-Adjusted Life Years)	Canada	Both	Age-standardized	Edentulism	Rate	2006	111.1	152.9	76.3
DALYs (Disability-Adjusted Life Years)	Federative Republic of Brazil	Male	Age-standardized	Edentulism	Rate	2010	205.0	282.5	139.4
DALYs (Disability-Adjusted Life Years)	Federative Republic of Brazil	Female	Age-standardized	Edentulism	Rate	2010	265.2	361.5	176.1
DALYs (Disability-Adjusted Life Years)	Federative Republic of Brazil	Both	Age-standardized	Edentulism	Rate	2010	237.3	324.5	160.1
DALYs (Disability-Adjusted Life Years)	Kingdom of Saudi Arabia	Male	Age-standardized	Edentulism	Rate	2010	157.1	213.7	96.9
DALYs (Disability-Adjusted Life Years)	Kingdom of Saudi Arabia	Female	Age-standardized	Edentulism	Rate	2010	108.4	147.8	70.0
DALYs (Disability-Adjusted Life Years)	Kingdom of Saudi Arabia	Both	Age-standardized	Edentulism	Rate	2010	136.6	186.1	85.8

DALYs (Disability-Adjusted Life Years)	Republic of Indonesia	Male	Age-standardized	Edentulism	Rate	2005	86.9	120.4	58.0
DALYs (Disability-Adjusted Life Years)	Republic of Indonesia	Female	Age-standardized	Edentulism	Rate	2005	143.9	191.3	98.1
DALYs (Disability-Adjusted Life Years)	Republic of Indonesia	Both	Age-standardized	Edentulism	Rate	2005	116.7	156.3	80.6
DALYs (Disability-Adjusted Life Years)	Republic of South Africa	Male	Age-standardized	Edentulism	Rate	2010	112.7	159.7	68.4
DALYs (Disability-Adjusted Life Years)	Republic of South Africa	Female	Age-standardized	Edentulism	Rate	2010	112.5	158.9	69.5
DALYs (Disability-Adjusted Life Years)	Republic of South Africa	Both	Age-standardized	Edentulism	Rate	2010	112.5	159.4	68.9
DALYs (Disability-Adjusted Life Years)	French Republic	Male	Age-standardized	Edentulism	Rate	2006	81.7	114.1	54.2
DALYs (Disability-Adjusted Life Years)	French Republic	Female	Age-standardized	Edentulism	Rate	2006	111.4	153.6	76.4
DALYs (Disability-Adjusted Life Years)	French Republic	Both	Age-standardized	Edentulism	Rate	2006	98.5	136.3	67.1
DALYs (Disability-Adjusted Life Years)	United Mexican States	Male	Age-standardized	Edentulism	Rate	2006	120.5	162.2	80.0
DALYs (Disability-Adjusted Life Years)	United Mexican States	Female	Age-standardized	Edentulism	Rate	2006	190.4	251.4	128.8
DALYs (Disability-Adjusted Life Years)	United Mexican States	Both	Age-standardized	Edentulism	Rate	2006	157.4	207.8	107.5
DALYs (Disability-Adjusted Life Years)	Republic of Indonesia	Male	Age-standardized	Edentulism	Rate	2006	87.1	119.8	59.2
DALYs (Disability-Adjusted Life Years)	Republic of Indonesia	Female	Age-standardized	Edentulism	Rate	2006	144.0	192.2	97.6
DALYs (Disability-Adjusted	Republic of Indonesia	Both	Age-standardized	Edentulism	Rate	2006	116.7	156.2	80.3

Life Years)									
DALYs (Disability-Adjusted Life Years)	United Kingdom of Great Britain and Northern Ireland	Male	Age-standardized	Edentulism	Rate	2005	101.9	143.3	64.4
DALYs (Disability-Adjusted Life Years)	United Kingdom of Great Britain and Northern Ireland	Female	Age-standardized	Edentulism	Rate	2005	127.3	180.1	80.2
DALYs (Disability-Adjusted Life Years)	United Kingdom of Great Britain and Northern Ireland	Both	Age-standardized	Edentulism	Rate	2005	115.3	163.1	72.7
DALYs (Disability-Adjusted Life Years)	United Kingdom of Great Britain and Northern Ireland	Male	Age-standardized	Edentulism	Rate	2006	94.4	133.3	60.4
DALYs (Disability-Adjusted Life Years)	United Kingdom of Great Britain and Northern Ireland	Female	Age-standardized	Edentulism	Rate	2006	119.5	168.3	76.2
DALYs (Disability-Adjusted Life Years)	United Kingdom of Great Britain and Northern Ireland	Both	Age-standardized	Edentulism	Rate	2006	107.7	152.5	68.7
DALYs (Disability-Adjusted Life Years)	Republic of India	Male	Age-standardized	Edentulism	Rate	2010	177.2	244.0	112.9
DALYs (Disability-Adjusted Life Years)	Republic of India	Female	Age-standardized	Edentulism	Rate	2010	180.6	246.9	115.8
DALYs (Disability-Adjusted Life Years)	Republic of India	Both	Age-standardized	Edentulism	Rate	2010	179.2	246.5	114.6
DALYs (Disability-Adjusted Life Years)	Argentine Republic	Male	Age-standardized	Edentulism	Rate	2007	89.8	126.3	57.0
DALYs (Disability-Adjusted Life Years)	Argentine Republic	Female	Age-standardized	Edentulism	Rate	2007	144.2	205.2	91.8
DALYs (Disability-Adjusted Life Years)	Argentine Republic	Both	Age-standardized	Edentulism	Rate	2007	120.3	171.5	76.5
DALYs (Disability-Adjusted Life Years)	United States of America	Male	Age-standardized	Edentulism	Rate	2007	105.3	142.5	67.7
DALYs (Disability-Adjusted Life Years)	United States of America	Female	Age-standardized	Edentulism	Rate	2007	116.3	156.5	75.5
DALYs	United States of	Both	Age-standardized	Edentulism	Rate	2007	111.3	150.0	71.8

(Disability-Adjusted Life Years)	America								
DALYs (Disability-Adjusted Life Years)	Argentine Republic	Male	Age-standardized	Edentulism	Rate	2006	90.5	127.1	56.8
DALYs (Disability-Adjusted Life Years)	Argentine Republic	Female	Age-standardized	Edentulism	Rate	2006	145.1	209.7	92.3
DALYs (Disability-Adjusted Life Years)	Argentine Republic	Both	Age-standardized	Edentulism	Rate	2006	121.1	172.4	76.8
DALYs (Disability-Adjusted Life Years)	United States of America	Male	Age-standardized	Edentulism	Rate	2006	110.1	149.4	70.6
DALYs (Disability-Adjusted Life Years)	United States of America	Female	Age-standardized	Edentulism	Rate	2006	122.4	164.8	79.3
DALYs (Disability-Adjusted Life Years)	United States of America	Both	Age-standardized	Edentulism	Rate	2006	116.8	157.7	75.2
DALYs (Disability-Adjusted Life Years)	Russian Federation	Male	Age-standardized	Edentulism	Rate	2006	143.9	193.0	91.9
DALYs (Disability-Adjusted Life Years)	Russian Federation	Female	Age-standardized	Edentulism	Rate	2006	159.9	215.9	105.7
DALYs (Disability-Adjusted Life Years)	Russian Federation	Both	Age-standardized	Edentulism	Rate	2006	155.0	208.9	101.3
DALYs (Disability-Adjusted Life Years)	Republic of Turkey	Male	Age-standardized	Edentulism	Rate	2006	231.9	327.1	154.6
DALYs (Disability-Adjusted Life Years)	Republic of Turkey	Female	Age-standardized	Edentulism	Rate	2006	259.0	365.4	173.9
DALYs (Disability-Adjusted Life Years)	Republic of Turkey	Both	Age-standardized	Edentulism	Rate	2006	245.7	346.4	164.5
DALYs (Disability-Adjusted Life Years)	Republic of India	Male	Age-standardized	Edentulism	Rate	2011	169.9	233.2	107.4
DALYs (Disability-Adjusted Life Years)	Republic of India	Female	Age-standardized	Edentulism	Rate	2011	174.3	237.8	111.2

DALYs (Disability-Adjusted Life Years)	Republic of India	Both	Age-standardized	Edentulism	Rate	2011	172.3	236.1	109.4
DALYs (Disability-Adjusted Life Years)	Republic of Korea	Male	Age-standardized	Edentulism	Rate	2011	38.8	54.0	26.4
DALYs (Disability-Adjusted Life Years)	Republic of Korea	Female	Age-standardized	Edentulism	Rate	2011	38.0	51.9	26.0
DALYs (Disability-Adjusted Life Years)	Republic of Korea	Both	Age-standardized	Edentulism	Rate	2011	39.0	53.9	26.5
DALYs (Disability-Adjusted Life Years)	Russian Federation	Male	Age-standardized	Edentulism	Rate	2007	140.5	188.6	90.5
DALYs (Disability-Adjusted Life Years)	Russian Federation	Female	Age-standardized	Edentulism	Rate	2007	164.3	218.3	109.5
DALYs (Disability-Adjusted Life Years)	Russian Federation	Both	Age-standardized	Edentulism	Rate	2007	156.1	207.4	103.4
DALYs (Disability-Adjusted Life Years)	Australia	Male	Age-standardized	Edentulism	Rate	2007	108.6	151.2	73.9
DALYs (Disability-Adjusted Life Years)	Australia	Female	Age-standardized	Edentulism	Rate	2007	144.4	199.4	98.2
DALYs (Disability-Adjusted Life Years)	Australia	Both	Age-standardized	Edentulism	Rate	2007	127.5	176.4	86.7
DALYs (Disability-Adjusted Life Years)	Federal Republic of Germany	Male	Age-standardized	Edentulism	Rate	2011	61.9	85.1	41.9
DALYs (Disability-Adjusted Life Years)	Federal Republic of Germany	Female	Age-standardized	Edentulism	Rate	2011	82.5	113.2	56.0
DALYs (Disability-Adjusted Life Years)	Federal Republic of Germany	Both	Age-standardized	Edentulism	Rate	2011	73.0	100.6	49.7
DALYs (Disability-Adjusted Life Years)	United Mexican States	Male	Age-standardized	Edentulism	Rate	2007	114.3	151.6	78.0
DALYs (Disability-Adjusted	United Mexican States	Female	Age-standardized	Edentulism	Rate	2007	185.6	244.0	124.5

Life Years)									
DALYs (Disability-Adjusted Life Years)	United Mexican States	Both	Age-standardized	Edentulism	Rate	2007	152.0	200.2	103.0
DALYs (Disability-Adjusted Life Years)	Japan	Male	Age-standardized	Edentulism	Rate	2007	123.5	172.8	81.0
DALYs (Disability-Adjusted Life Years)	Japan	Female	Age-standardized	Edentulism	Rate	2007	68.0	96.0	45.2
DALYs (Disability-Adjusted Life Years)	Japan	Both	Age-standardized	Edentulism	Rate	2007	94.9	132.9	62.9
DALYs (Disability-Adjusted Life Years)	European Union	Male	Age-standardized	Edentulism	Rate	2006	113.3	156.4	76.1
DALYs (Disability-Adjusted Life Years)	European Union	Female	Age-standardized	Edentulism	Rate	2006	137.5	190.3	92.7
DALYs (Disability-Adjusted Life Years)	European Union	Both	Age-standardized	Edentulism	Rate	2006	126.4	174.8	85.2
DALYs (Disability-Adjusted Life Years)	Federative Republic of Brazil	Male	Age-standardized	Edentulism	Rate	2011	205.1	280.4	137.9
DALYs (Disability-Adjusted Life Years)	Federative Republic of Brazil	Female	Age-standardized	Edentulism	Rate	2011	265.6	361.7	176.0
DALYs (Disability-Adjusted Life Years)	Federative Republic of Brazil	Both	Age-standardized	Edentulism	Rate	2011	237.5	324.3	159.7
DALYs (Disability-Adjusted Life Years)	Kingdom of Saudi Arabia	Male	Age-standardized	Edentulism	Rate	2011	156.2	213.3	98.0
DALYs (Disability-Adjusted Life Years)	Kingdom of Saudi Arabia	Female	Age-standardized	Edentulism	Rate	2011	107.9	146.2	69.9
DALYs (Disability-Adjusted Life Years)	Kingdom of Saudi Arabia	Both	Age-standardized	Edentulism	Rate	2011	135.9	186.4	86.5
DALYs (Disability-Adjusted Life Years)	Republic of South Africa	Male	Age-standardized	Edentulism	Rate	2011	118.8	168.0	72.4
DALYs	Republic of South	Female	Age-standardized	Edentulism	Rate	2011	118.1	167.7	73.2

(Disability-Adjusted Life Years)	Africa								
DALYs (Disability-Adjusted Life Years)	Republic of South Africa	Both	Age-standardized	Edentulism	Rate	2011	118.3	167.6	72.7
DALYs (Disability-Adjusted Life Years)	French Republic	Male	Age-standardized	Edentulism	Rate	2007	74.5	104.4	50.1
DALYs (Disability-Adjusted Life Years)	French Republic	Female	Age-standardized	Edentulism	Rate	2007	102.2	142.3	69.5
DALYs (Disability-Adjusted Life Years)	French Republic	Both	Age-standardized	Edentulism	Rate	2007	90.0	125.6	61.5
DALYs (Disability-Adjusted Life Years)	Republic of Turkey	Male	Age-standardized	Edentulism	Rate	2007	224.7	315.3	149.0
DALYs (Disability-Adjusted Life Years)	Republic of Turkey	Female	Age-standardized	Edentulism	Rate	2007	253.3	353.2	171.4
DALYs (Disability-Adjusted Life Years)	Republic of Turkey	Both	Age-standardized	Edentulism	Rate	2007	239.4	334.5	160.7
DALYs (Disability-Adjusted Life Years)	United Mexican States	Male	Age-standardized	Edentulism	Rate	2008	107.0	141.6	72.5
DALYs (Disability-Adjusted Life Years)	United Mexican States	Female	Age-standardized	Edentulism	Rate	2008	179.9	238.2	119.8
DALYs (Disability-Adjusted Life Years)	United Mexican States	Both	Age-standardized	Edentulism	Rate	2008	145.5	192.6	97.0
DALYs (Disability-Adjusted Life Years)	Australia	Male	Age-standardized	Edentulism	Rate	2006	102.3	141.0	69.3
DALYs (Disability-Adjusted Life Years)	Australia	Female	Age-standardized	Edentulism	Rate	2006	137.3	189.9	93.7
DALYs (Disability-Adjusted Life Years)	Australia	Both	Age-standardized	Edentulism	Rate	2006	120.8	166.3	81.6
DALYs (Disability-Adjusted Life Years)	Republic of Indonesia	Male	Age-standardized	Edentulism	Rate	2007	87.2	118.7	60.0

DALYs (Disability-Adjusted Life Years)	Republic of Indonesia	Female	Age-standardized	Edentulism	Rate	2007	143.8	191.4	97.6
DALYs (Disability-Adjusted Life Years)	Republic of Indonesia	Both	Age-standardized	Edentulism	Rate	2007	116.7	155.5	79.9
DALYs (Disability-Adjusted Life Years)	Canada	Male	Age-standardized	Edentulism	Rate	2007	107.5	148.1	73.0
DALYs (Disability-Adjusted Life Years)	Canada	Female	Age-standardized	Edentulism	Rate	2007	113.6	160.0	77.7
DALYs (Disability-Adjusted Life Years)	Canada	Both	Age-standardized	Edentulism	Rate	2007	110.8	154.3	76.0
DALYs (Disability-Adjusted Life Years)	Republic of Korea	Male	Age-standardized	Edentulism	Rate	2012	45.9	63.5	30.8
DALYs (Disability-Adjusted Life Years)	Republic of Korea	Female	Age-standardized	Edentulism	Rate	2012	45.3	62.0	31.2
DALYs (Disability-Adjusted Life Years)	Republic of Korea	Both	Age-standardized	Edentulism	Rate	2012	46.5	63.8	31.6
DALYs (Disability-Adjusted Life Years)	People's Republic of China	Male	Age-standardized	Edentulism	Rate	2007	74.1	105.3	49.3
DALYs (Disability-Adjusted Life Years)	People's Republic of China	Female	Age-standardized	Edentulism	Rate	2007	88.5	124.3	58.3
DALYs (Disability-Adjusted Life Years)	People's Republic of China	Both	Age-standardized	Edentulism	Rate	2007	81.4	115.2	54.1
DALYs (Disability-Adjusted Life Years)	Federal Republic of Germany	Male	Age-standardized	Edentulism	Rate	2012	70.6	96.3	48.2
DALYs (Disability-Adjusted Life Years)	Federal Republic of Germany	Female	Age-standardized	Edentulism	Rate	2012	92.9	126.9	62.7
DALYs (Disability-Adjusted Life Years)	Federal Republic of Germany	Both	Age-standardized	Edentulism	Rate	2012	82.5	112.6	55.8
DALYs (Disability-Adjusted	French Republic	Male	Age-standardized	Edentulism	Rate	2008	66.6	92.9	45.8

Life Years)									
DALYs (Disability-Adjusted Life Years)	French Republic	Female	Age-standardized	Edentulism	Rate	2008	91.6	126.9	62.9
DALYs (Disability-Adjusted Life Years)	French Republic	Both	Age-standardized	Edentulism	Rate	2008	80.5	111.1	55.4
DALYs (Disability-Adjusted Life Years)	Kingdom of Saudi Arabia	Male	Age-standardized	Edentulism	Rate	2012	155.2	209.8	97.8
DALYs (Disability-Adjusted Life Years)	Kingdom of Saudi Arabia	Female	Age-standardized	Edentulism	Rate	2012	107.4	146.6	69.1
DALYs (Disability-Adjusted Life Years)	Kingdom of Saudi Arabia	Both	Age-standardized	Edentulism	Rate	2012	135.1	184.1	85.8
DALYs (Disability-Adjusted Life Years)	United Kingdom of Great Britain and Northern Ireland	Male	Age-standardized	Edentulism	Rate	2007	86.4	122.0	55.9
DALYs (Disability-Adjusted Life Years)	United Kingdom of Great Britain and Northern Ireland	Female	Age-standardized	Edentulism	Rate	2007	110.5	154.7	71.1
DALYs (Disability-Adjusted Life Years)	United Kingdom of Great Britain and Northern Ireland	Both	Age-standardized	Edentulism	Rate	2007	99.2	139.8	64.0
DALYs (Disability-Adjusted Life Years)	People's Republic of China	Male	Age-standardized	Edentulism	Rate	2008	74.7	106.3	49.8
DALYs (Disability-Adjusted Life Years)	People's Republic of China	Female	Age-standardized	Edentulism	Rate	2008	89.8	126.0	59.1
DALYs (Disability-Adjusted Life Years)	People's Republic of China	Both	Age-standardized	Edentulism	Rate	2008	82.3	116.6	54.8
DALYs (Disability-Adjusted Life Years)	Republic of Italy	Male	Age-standardized	Edentulism	Rate	2006	108.4	154.0	70.5
DALYs (Disability-Adjusted Life Years)	Republic of Italy	Female	Age-standardized	Edentulism	Rate	2006	109.5	153.2	69.9
DALYs (Disability-Adjusted Life Years)	Republic of Italy	Both	Age-standardized	Edentulism	Rate	2006	108.5	152.7	69.8
DALYs	Republic of South	Male	Age-standardized	Edentulism	Rate	2012	133.4	188.3	81.6

(Disability-Adjusted Life Years)	Africa								
DALYs (Disability-Adjusted Life Years)	Republic of South Africa	Female	Age-standardized	Edentulism	Rate	2012	131.7	185.5	81.7
DALYs (Disability-Adjusted Life Years)	Republic of South Africa	Both	Age-standardized	Edentulism	Rate	2012	132.4	186.6	81.6
DALYs (Disability-Adjusted Life Years)	Canada	Male	Age-standardized	Edentulism	Rate	2008	107.0	148.1	73.0
DALYs (Disability-Adjusted Life Years)	Canada	Female	Age-standardized	Edentulism	Rate	2008	113.4	157.4	78.4
DALYs (Disability-Adjusted Life Years)	Canada	Both	Age-standardized	Edentulism	Rate	2008	110.4	152.6	75.7
DALYs (Disability-Adjusted Life Years)	Federative Republic of Brazil	Male	Age-standardized	Edentulism	Rate	2012	207.2	280.3	138.6
DALYs (Disability-Adjusted Life Years)	Federative Republic of Brazil	Female	Age-standardized	Edentulism	Rate	2012	268.7	364.9	178.7
DALYs (Disability-Adjusted Life Years)	Federative Republic of Brazil	Both	Age-standardized	Edentulism	Rate	2012	240.2	326.7	160.6
DALYs (Disability-Adjusted Life Years)	Republic of Italy	Male	Age-standardized	Edentulism	Rate	2007	95.7	135.0	61.6
DALYs (Disability-Adjusted Life Years)	Republic of Italy	Female	Age-standardized	Edentulism	Rate	2007	105.4	147.7	66.8
DALYs (Disability-Adjusted Life Years)	Republic of Italy	Both	Age-standardized	Edentulism	Rate	2007	100.6	141.4	64.2
DALYs (Disability-Adjusted Life Years)	United Kingdom of Great Britain and Northern Ireland	Male	Age-standardized	Edentulism	Rate	2008	79.2	111.7	52.0
DALYs (Disability-Adjusted Life Years)	United Kingdom of Great Britain and Northern Ireland	Female	Age-standardized	Edentulism	Rate	2008	101.8	142.3	66.5
DALYs (Disability-Adjusted Life Years)	United Kingdom of Great Britain and Northern Ireland	Both	Age-standardized	Edentulism	Rate	2008	91.2	127.9	59.8

DALYs (Disability-Adjusted Life Years)	Republic of Indonesia	Male	Age-standardized	Edentulism	Rate	2008	87.1	118.2	59.6
DALYs (Disability-Adjusted Life Years)	Republic of Indonesia	Female	Age-standardized	Edentulism	Rate	2008	143.5	191.4	96.7
DALYs (Disability-Adjusted Life Years)	Republic of Indonesia	Both	Age-standardized	Edentulism	Rate	2008	116.5	155.4	79.1
DALYs (Disability-Adjusted Life Years)	Republic of India	Male	Age-standardized	Edentulism	Rate	2012	151.9	208.7	95.4
DALYs (Disability-Adjusted Life Years)	Republic of India	Female	Age-standardized	Edentulism	Rate	2012	158.1	215.7	100.2
DALYs (Disability-Adjusted Life Years)	Republic of India	Both	Age-standardized	Edentulism	Rate	2012	155.2	212.9	97.9
DALYs (Disability-Adjusted Life Years)	Argentine Republic	Male	Age-standardized	Edentulism	Rate	2008	89.1	127.0	56.2
DALYs (Disability-Adjusted Life Years)	Argentine Republic	Female	Age-standardized	Edentulism	Rate	2008	143.3	205.4	90.1
DALYs (Disability-Adjusted Life Years)	Argentine Republic	Both	Age-standardized	Edentulism	Rate	2008	119.4	170.0	75.2
DALYs (Disability-Adjusted Life Years)	United States of America	Male	Age-standardized	Edentulism	Rate	2008	100.3	135.4	64.7
DALYs (Disability-Adjusted Life Years)	United States of America	Female	Age-standardized	Edentulism	Rate	2008	109.8	148.1	71.8
DALYs (Disability-Adjusted Life Years)	United States of America	Both	Age-standardized	Edentulism	Rate	2008	105.4	142.1	68.3
DALYs (Disability-Adjusted Life Years)	European Union	Male	Age-standardized	Edentulism	Rate	2007	104.3	143.7	70.0
DALYs (Disability-Adjusted Life Years)	European Union	Female	Age-standardized	Edentulism	Rate	2007	129.4	179.0	87.1
DALYs (Disability-Adjusted	European Union	Both	Age-standardized	Edentulism	Rate	2007	117.9	162.7	79.3

Life Years)									
DALYs (Disability-Adjusted Life Years)	United States of America	Male	Age-standardized	Edentulism	Rate	2009	96.3	130.0	62.5
DALYs (Disability-Adjusted Life Years)	United States of America	Female	Age-standardized	Edentulism	Rate	2009	104.6	141.5	68.7
DALYs (Disability-Adjusted Life Years)	United States of America	Both	Age-standardized	Edentulism	Rate	2009	100.8	136.0	65.6
DALYs (Disability-Adjusted Life Years)	Republic of India	Male	Age-standardized	Edentulism	Rate	2013	129.9	178.8	80.7
DALYs (Disability-Adjusted Life Years)	Republic of India	Female	Age-standardized	Edentulism	Rate	2013	138.3	190.5	86.5
DALYs (Disability-Adjusted Life Years)	Republic of India	Both	Age-standardized	Edentulism	Rate	2013	134.2	185.0	83.7
DALYs (Disability-Adjusted Life Years)	Japan	Male	Age-standardized	Edentulism	Rate	2008	102.9	144.3	67.4
DALYs (Disability-Adjusted Life Years)	Japan	Female	Age-standardized	Edentulism	Rate	2008	65.0	91.5	43.7
DALYs (Disability-Adjusted Life Years)	Japan	Both	Age-standardized	Edentulism	Rate	2008	83.5	117.1	55.6
DALYs (Disability-Adjusted Life Years)	Republic of Korea	Male	Age-standardized	Edentulism	Rate	2013	54.4	75.2	36.8
DALYs (Disability-Adjusted Life Years)	Republic of Korea	Female	Age-standardized	Edentulism	Rate	2013	54.2	74.5	37.0
DALYs (Disability-Adjusted Life Years)	Republic of Korea	Both	Age-standardized	Edentulism	Rate	2013	55.4	76.2	37.9
DALYs (Disability-Adjusted Life Years)	Republic of Turkey	Male	Age-standardized	Edentulism	Rate	2008	216.0	303.1	144.3
DALYs (Disability-Adjusted Life Years)	Republic of Turkey	Female	Age-standardized	Edentulism	Rate	2008	245.5	341.6	164.6
DALYs	Republic of Turkey	Both	Age-standardized	Edentulism	Rate	2008	231.4	323.0	154.5

(Disability-Adjusted Life Years)									
DALYs (Disability-Adjusted Life Years)	Federal Republic of Germany	Male	Age-standardized	Edentulism	Rate	2013	81.0	111.2	53.8
DALYs (Disability-Adjusted Life Years)	Federal Republic of Germany	Female	Age-standardized	Edentulism	Rate	2013	105.4	146.2	71.7
DALYs (Disability-Adjusted Life Years)	Federal Republic of Germany	Both	Age-standardized	Edentulism	Rate	2013	94.0	129.8	63.8
DALYs (Disability-Adjusted Life Years)	European Union	Male	Age-standardized	Edentulism	Rate	2008	94.0	129.3	63.2
DALYs (Disability-Adjusted Life Years)	European Union	Female	Age-standardized	Edentulism	Rate	2008	120.3	166.7	80.9
DALYs (Disability-Adjusted Life Years)	European Union	Both	Age-standardized	Edentulism	Rate	2008	108.3	149.5	72.8
DALYs (Disability-Adjusted Life Years)	Federative Republic of Brazil	Male	Age-standardized	Edentulism	Rate	2013	210.0	282.3	139.9
DALYs (Disability-Adjusted Life Years)	Federative Republic of Brazil	Female	Age-standardized	Edentulism	Rate	2013	272.8	368.5	181.4
DALYs (Disability-Adjusted Life Years)	Federative Republic of Brazil	Both	Age-standardized	Edentulism	Rate	2013	243.7	329.8	162.1
DALYs (Disability-Adjusted Life Years)	Japan	Male	Age-standardized	Edentulism	Rate	2009	83.8	117.0	54.9
DALYs (Disability-Adjusted Life Years)	Japan	Female	Age-standardized	Edentulism	Rate	2009	61.8	87.0	41.6
DALYs (Disability-Adjusted Life Years)	Japan	Both	Age-standardized	Edentulism	Rate	2009	72.7	101.6	48.2
DALYs (Disability-Adjusted Life Years)	Kingdom of Saudi Arabia	Male	Age-standardized	Edentulism	Rate	2013	154.5	211.0	99.0
DALYs (Disability-Adjusted Life Years)	Kingdom of Saudi Arabia	Female	Age-standardized	Edentulism	Rate	2013	106.7	146.0	68.4

DALYs (Disability-Adjusted Life Years)	Kingdom of Saudi Arabia	Both	Age-standardized	Edentulism	Rate	2013	134.5	184.1	86.1
DALYs (Disability-Adjusted Life Years)	Republic of South Africa	Male	Age-standardized	Edentulism	Rate	2013	150.8	215.3	93.5
DALYs (Disability-Adjusted Life Years)	Republic of South Africa	Female	Age-standardized	Edentulism	Rate	2013	147.9	208.5	91.7
DALYs (Disability-Adjusted Life Years)	Republic of South Africa	Both	Age-standardized	Edentulism	Rate	2013	149.2	211.4	92.5
DALYs (Disability-Adjusted Life Years)	Russian Federation	Male	Age-standardized	Edentulism	Rate	2008	137.2	184.2	89.0
DALYs (Disability-Adjusted Life Years)	Russian Federation	Female	Age-standardized	Edentulism	Rate	2008	169.6	224.8	113.3
DALYs (Disability-Adjusted Life Years)	Russian Federation	Both	Age-standardized	Edentulism	Rate	2008	157.8	209.1	104.9
DALYs (Disability-Adjusted Life Years)	United Mexican States	Male	Age-standardized	Edentulism	Rate	2009	100.9	134.1	67.6
DALYs (Disability-Adjusted Life Years)	United Mexican States	Female	Age-standardized	Edentulism	Rate	2009	175.2	232.3	117.0
DALYs (Disability-Adjusted Life Years)	United Mexican States	Both	Age-standardized	Edentulism	Rate	2009	140.2	186.3	93.8
DALYs (Disability-Adjusted Life Years)	United Mexican States	Male	Age-standardized	Edentulism	Rate	2010	98.3	131.8	66.2
DALYs (Disability-Adjusted Life Years)	United Mexican States	Female	Age-standardized	Edentulism	Rate	2010	173.4	230.8	115.9
DALYs (Disability-Adjusted Life Years)	United Mexican States	Both	Age-standardized	Edentulism	Rate	2010	138.1	184.2	92.2
DALYs (Disability-Adjusted Life Years)	Republic of Turkey	Male	Age-standardized	Edentulism	Rate	2009	208.4	289.7	140.6
DALYs (Disability-Adjusted	Republic of Turkey	Female	Age-standardized	Edentulism	Rate	2009	237.7	328.9	161.7

Life Years)									
DALYs (Disability-Adjusted Life Years)	Republic of Turkey	Both	Age-standardized	Edentulism	Rate	2009	223.9	310.2	152.0
DALYs (Disability-Adjusted Life Years)	Republic of Indonesia	Male	Age-standardized	Edentulism	Rate	2010	86.8	116.4	58.2
DALYs (Disability-Adjusted Life Years)	Republic of Indonesia	Female	Age-standardized	Edentulism	Rate	2010	142.6	191.7	95.1
DALYs (Disability-Adjusted Life Years)	Republic of Indonesia	Both	Age-standardized	Edentulism	Rate	2010	115.9	155.3	77.5
DALYs (Disability-Adjusted Life Years)	Russian Federation	Male	Age-standardized	Edentulism	Rate	2009	134.6	180.3	88.0
DALYs (Disability-Adjusted Life Years)	Russian Federation	Female	Age-standardized	Edentulism	Rate	2009	174.1	230.2	116.4
DALYs (Disability-Adjusted Life Years)	Russian Federation	Both	Age-standardized	Edentulism	Rate	2009	159.3	211.4	106.4
DALYs (Disability-Adjusted Life Years)	Australia	Male	Age-standardized	Edentulism	Rate	2008	115.9	161.1	79.5
DALYs (Disability-Adjusted Life Years)	Australia	Female	Age-standardized	Edentulism	Rate	2008	153.4	212.9	104.7
DALYs (Disability-Adjusted Life Years)	Australia	Both	Age-standardized	Edentulism	Rate	2008	135.6	188.4	93.2
DALYs (Disability-Adjusted Life Years)	Australia	Male	Age-standardized	Edentulism	Rate	2009	122.2	170.1	83.8
DALYs (Disability-Adjusted Life Years)	Australia	Female	Age-standardized	Edentulism	Rate	2009	162.0	223.1	111.6
DALYs (Disability-Adjusted Life Years)	Australia	Both	Age-standardized	Edentulism	Rate	2009	143.0	197.9	98.4
DALYs (Disability-Adjusted Life Years)	People's Republic of China	Male	Age-standardized	Edentulism	Rate	2010	75.3	106.9	50.2
DALYs	People's Republic	Female	Age-standardized	Edentulism	Rate	2010	91.0	127.3	59.9

(Disability-Adjusted Life Years)	of China								
DALYs (Disability-Adjusted Life Years)	People's Republic of China	Both	Age-standardized	Edentulism	Rate	2010	83.2	117.8	55.4
DALYs (Disability-Adjusted Life Years)	Republic of Indonesia	Male	Age-standardized	Edentulism	Rate	2009	87.1	117.6	59.2
DALYs (Disability-Adjusted Life Years)	Republic of Indonesia	Female	Age-standardized	Edentulism	Rate	2009	143.1	191.5	95.7
DALYs (Disability-Adjusted Life Years)	Republic of Indonesia	Both	Age-standardized	Edentulism	Rate	2009	116.2	155.5	78.3
DALYs (Disability-Adjusted Life Years)	French Republic	Male	Age-standardized	Edentulism	Rate	2010	57.0	78.8	39.1
DALYs (Disability-Adjusted Life Years)	French Republic	Female	Age-standardized	Edentulism	Rate	2010	79.5	111.9	53.7
DALYs (Disability-Adjusted Life Years)	French Republic	Both	Age-standardized	Edentulism	Rate	2010	69.2	96.5	47.3
DALYs (Disability-Adjusted Life Years)	Canada	Male	Age-standardized	Edentulism	Rate	2010	106.0	146.0	72.3
DALYs (Disability-Adjusted Life Years)	Canada	Female	Age-standardized	Edentulism	Rate	2010	112.3	153.4	76.5
DALYs (Disability-Adjusted Life Years)	Canada	Both	Age-standardized	Edentulism	Rate	2010	109.4	151.7	74.5
DALYs (Disability-Adjusted Life Years)	Canada	Male	Age-standardized	Edentulism	Rate	2009	106.7	146.8	72.6
DALYs (Disability-Adjusted Life Years)	Canada	Female	Age-standardized	Edentulism	Rate	2009	113.0	154.9	78.0
DALYs (Disability-Adjusted Life Years)	Canada	Both	Age-standardized	Edentulism	Rate	2009	110.1	151.9	75.5
DALYs (Disability-Adjusted Life Years)	Republic of Italy	Male	Age-standardized	Edentulism	Rate	2008	80.4	112.1	51.1

DALYs (Disability-Adjusted Life Years)	Republic of Italy	Female	Age-standardized	Edentulism	Rate	2008	100.9	141.8	64.0
DALYs (Disability-Adjusted Life Years)	Republic of Italy	Both	Age-standardized	Edentulism	Rate	2008	91.3	128.2	57.7
DALYs (Disability-Adjusted Life Years)	People's Republic of China	Male	Age-standardized	Edentulism	Rate	2009	75.2	107.1	50.3
DALYs (Disability-Adjusted Life Years)	People's Republic of China	Female	Age-standardized	Edentulism	Rate	2009	90.7	127.1	60.1
DALYs (Disability-Adjusted Life Years)	People's Republic of China	Both	Age-standardized	Edentulism	Rate	2009	83.0	117.7	55.3
DALYs (Disability-Adjusted Life Years)	United Kingdom of Great Britain and Northern Ireland	Male	Age-standardized	Edentulism	Rate	2010	71.9	100.9	47.8
DALYs (Disability-Adjusted Life Years)	United Kingdom of Great Britain and Northern Ireland	Female	Age-standardized	Edentulism	Rate	2010	92.9	129.9	61.8
DALYs (Disability-Adjusted Life Years)	United Kingdom of Great Britain and Northern Ireland	Both	Age-standardized	Edentulism	Rate	2010	83.0	116.2	55.5
DALYs (Disability-Adjusted Life Years)	Republic of Italy	Male	Age-standardized	Edentulism	Rate	2009	67.7	93.9	42.8
DALYs (Disability-Adjusted Life Years)	Republic of Italy	Female	Age-standardized	Edentulism	Rate	2009	97.3	136.4	61.6
DALYs (Disability-Adjusted Life Years)	Republic of Italy	Both	Age-standardized	Edentulism	Rate	2009	83.7	116.7	52.9
DALYs (Disability-Adjusted Life Years)	United Kingdom of Great Britain and Northern Ireland	Male	Age-standardized	Edentulism	Rate	2009	73.9	103.7	49.0
DALYs (Disability-Adjusted Life Years)	United Kingdom of Great Britain and Northern Ireland	Female	Age-standardized	Edentulism	Rate	2009	95.4	133.0	63.2
DALYs (Disability-Adjusted Life Years)	United Kingdom of Great Britain and Northern Ireland	Both	Age-standardized	Edentulism	Rate	2009	85.3	118.9	56.7
DALYs (Disability-Adjusted	French Republic	Male	Age-standardized	Edentulism	Rate	2009	59.7	82.1	40.4

Life Years)									
DALYs (Disability-Adjusted Life Years)	French Republic	Female	Age-standardized	Edentulism	Rate	2009	83.1	115.0	56.3
DALYs (Disability-Adjusted Life Years)	French Republic	Both	Age-standardized	Edentulism	Rate	2009	72.5	99.8	48.9
DALYs (Disability-Adjusted Life Years)	Republic of Korea	Male	Age-standardized	Edentulism	Rate	2014	61.5	86.7	41.8
DALYs (Disability-Adjusted Life Years)	Republic of Korea	Female	Age-standardized	Edentulism	Rate	2014	61.5	84.9	42.0
DALYs (Disability-Adjusted Life Years)	Republic of Korea	Both	Age-standardized	Edentulism	Rate	2014	62.9	87.6	43.3
DALYs (Disability-Adjusted Life Years)	Federal Republic of Germany	Male	Age-standardized	Edentulism	Rate	2014	89.7	126.7	57.5
DALYs (Disability-Adjusted Life Years)	Federal Republic of Germany	Female	Age-standardized	Edentulism	Rate	2014	115.7	159.8	76.6
DALYs (Disability-Adjusted Life Years)	Federal Republic of Germany	Both	Age-standardized	Edentulism	Rate	2014	103.5	143.0	67.1
DALYs (Disability-Adjusted Life Years)	Kingdom of Saudi Arabia	Male	Age-standardized	Edentulism	Rate	2014	153.8	210.5	99.7
DALYs (Disability-Adjusted Life Years)	Kingdom of Saudi Arabia	Female	Age-standardized	Edentulism	Rate	2014	106.4	144.6	68.2
DALYs (Disability-Adjusted Life Years)	Kingdom of Saudi Arabia	Both	Age-standardized	Edentulism	Rate	2014	134.0	182.6	86.6
DALYs (Disability-Adjusted Life Years)	Federative Republic of Brazil	Male	Age-standardized	Edentulism	Rate	2014	212.3	288.1	141.1
DALYs (Disability-Adjusted Life Years)	Federative Republic of Brazil	Female	Age-standardized	Edentulism	Rate	2014	276.6	374.8	184.2
DALYs (Disability-Adjusted Life Years)	Federative Republic of Brazil	Both	Age-standardized	Edentulism	Rate	2014	246.7	332.0	163.5
DALYs	Argentine Republic	Male	Age-standardized	Edentulism	Rate	2009	88.2	124.4	55.8

(Disability-Adjusted Life Years)									
DALYs (Disability-Adjusted Life Years)	Argentine Republic	Female	Age-standardized	Edentulism	Rate	2009	142.2	202.3	88.9
DALYs (Disability-Adjusted Life Years)	Argentine Republic	Both	Age-standardized	Edentulism	Rate	2009	118.4	166.8	74.5
DALYs (Disability-Adjusted Life Years)	Republic of South Africa	Male	Age-standardized	Edentulism	Rate	2014	165.6	237.2	102.6
DALYs (Disability-Adjusted Life Years)	Republic of South Africa	Female	Age-standardized	Edentulism	Rate	2014	161.5	229.0	99.6
DALYs (Disability-Adjusted Life Years)	Republic of South Africa	Both	Age-standardized	Edentulism	Rate	2014	163.3	232.9	101.0
DALYs (Disability-Adjusted Life Years)	United States of America	Male	Age-standardized	Edentulism	Rate	2010	94.6	127.4	61.4
DALYs (Disability-Adjusted Life Years)	United States of America	Female	Age-standardized	Edentulism	Rate	2010	102.3	138.2	67.3
DALYs (Disability-Adjusted Life Years)	United States of America	Both	Age-standardized	Edentulism	Rate	2010	98.8	133.2	64.4
DALYs (Disability-Adjusted Life Years)	Republic of India	Male	Age-standardized	Edentulism	Rate	2014	110.3	153.7	68.1
DALYs (Disability-Adjusted Life Years)	Republic of India	Female	Age-standardized	Edentulism	Rate	2014	121.1	166.5	74.9
DALYs (Disability-Adjusted Life Years)	Republic of India	Both	Age-standardized	Edentulism	Rate	2014	115.7	160.1	71.4
DALYs (Disability-Adjusted Life Years)	Argentine Republic	Male	Age-standardized	Edentulism	Rate	2010	87.6	124.8	55.2
DALYs (Disability-Adjusted Life Years)	Argentine Republic	Female	Age-standardized	Edentulism	Rate	2010	141.2	199.8	89.7
DALYs (Disability-Adjusted Life Years)	Argentine Republic	Both	Age-standardized	Edentulism	Rate	2010	117.6	165.0	74.6

DALYs (Disability-Adjusted Life Years)	United States of America	Male	Age-standardized	Edentulism	Rate	2011	96.1	129.1	62.1
DALYs (Disability-Adjusted Life Years)	United States of America	Female	Age-standardized	Edentulism	Rate	2011	104.1	140.1	68.6
DALYs (Disability-Adjusted Life Years)	United States of America	Both	Age-standardized	Edentulism	Rate	2011	100.4	135.0	65.6
DALYs (Disability-Adjusted Life Years)	Japan	Male	Age-standardized	Edentulism	Rate	2011	64.4	89.7	42.3
DALYs (Disability-Adjusted Life Years)	Japan	Female	Age-standardized	Edentulism	Rate	2011	55.5	77.2	37.6
DALYs (Disability-Adjusted Life Years)	Japan	Both	Age-standardized	Edentulism	Rate	2011	60.1	83.9	40.3
DALYs (Disability-Adjusted Life Years)	Republic of Korea	Male	Age-standardized	Edentulism	Rate	2015	64.5	93.0	42.9
DALYs (Disability-Adjusted Life Years)	Republic of Korea	Female	Age-standardized	Edentulism	Rate	2015	64.7	90.8	44.4
DALYs (Disability-Adjusted Life Years)	Republic of Korea	Both	Age-standardized	Edentulism	Rate	2015	66.0	93.6	45.2
DALYs (Disability-Adjusted Life Years)	European Union	Male	Age-standardized	Edentulism	Rate	2010	81.8	112.3	54.7
DALYs (Disability-Adjusted Life Years)	European Union	Female	Age-standardized	Edentulism	Rate	2010	109.8	151.9	74.0
DALYs (Disability-Adjusted Life Years)	European Union	Both	Age-standardized	Edentulism	Rate	2010	97.0	133.8	65.1
DALYs (Disability-Adjusted Life Years)	Federal Republic of Germany	Male	Age-standardized	Edentulism	Rate	2015	93.4	134.0	59.1
DALYs (Disability-Adjusted Life Years)	Federal Republic of Germany	Female	Age-standardized	Edentulism	Rate	2015	120.0	167.2	76.6
DALYs (Disability-Adjusted	Federal Republic of Germany	Both	Age-standardized	Edentulism	Rate	2015	107.5	151.4	68.0

Life Years)									
DALYs (Disability-Adjusted Life Years)	Federative Republic of Brazil	Male	Age-standardized	Edentulism	Rate	2015	212.8	290.9	141.4
DALYs (Disability-Adjusted Life Years)	Federative Republic of Brazil	Female	Age-standardized	Edentulism	Rate	2015	278.3	374.4	185.4
DALYs (Disability-Adjusted Life Years)	Federative Republic of Brazil	Both	Age-standardized	Edentulism	Rate	2015	247.9	331.0	163.6
DALYs (Disability-Adjusted Life Years)	Japan	Male	Age-standardized	Edentulism	Rate	2010	71.4	99.6	46.8
DALYs (Disability-Adjusted Life Years)	Japan	Female	Age-standardized	Edentulism	Rate	2010	58.9	82.5	39.8
DALYs (Disability-Adjusted Life Years)	Japan	Both	Age-standardized	Edentulism	Rate	2010	65.2	91.2	43.4
DALYs (Disability-Adjusted Life Years)	Republic of Turkey	Male	Age-standardized	Edentulism	Rate	2010	204.5	284.6	138.3
DALYs (Disability-Adjusted Life Years)	Republic of Turkey	Female	Age-standardized	Edentulism	Rate	2010	231.9	320.8	158.5
DALYs (Disability-Adjusted Life Years)	Republic of Turkey	Both	Age-standardized	Edentulism	Rate	2010	219.1	303.9	149.1
DALYs (Disability-Adjusted Life Years)	United Mexican States	Male	Age-standardized	Edentulism	Rate	2012	100.7	133.9	66.9
DALYs (Disability-Adjusted Life Years)	United Mexican States	Female	Age-standardized	Edentulism	Rate	2012	177.6	235.2	118.0
DALYs (Disability-Adjusted Life Years)	United Mexican States	Both	Age-standardized	Edentulism	Rate	2012	141.4	187.3	94.1
DALYs (Disability-Adjusted Life Years)	Republic of Indonesia	Male	Age-standardized	Edentulism	Rate	2012	86.3	117.1	57.7
DALYs (Disability-Adjusted Life Years)	Republic of Indonesia	Female	Age-standardized	Edentulism	Rate	2012	141.3	189.6	94.0
DALYs	Republic of	Both	Age-standardized	Edentulism	Rate	2012	114.8	154.1	76.6

(Disability-Adjusted Life Years)	Indonesia								
DALYs (Disability-Adjusted Life Years)	Russian Federation	Male	Age-standardized	Edentulism	Rate	2010	133.5	178.2	88.2
DALYs (Disability-Adjusted Life Years)	Russian Federation	Female	Age-standardized	Edentulism	Rate	2010	175.9	234.4	116.5
DALYs (Disability-Adjusted Life Years)	Russian Federation	Both	Age-standardized	Edentulism	Rate	2010	159.9	212.1	106.8
DALYs (Disability-Adjusted Life Years)	Australia	Male	Age-standardized	Edentulism	Rate	2010	125.6	174.8	86.6
DALYs (Disability-Adjusted Life Years)	Australia	Female	Age-standardized	Edentulism	Rate	2010	167.4	229.3	115.6
DALYs (Disability-Adjusted Life Years)	Australia	Both	Age-standardized	Edentulism	Rate	2010	147.5	202.6	102.2
DALYs (Disability-Adjusted Life Years)	Kingdom of Saudi Arabia	Male	Age-standardized	Edentulism	Rate	2015	153.4	209.1	100.4
DALYs (Disability-Adjusted Life Years)	Kingdom of Saudi Arabia	Female	Age-standardized	Edentulism	Rate	2015	106.2	144.6	67.9
DALYs (Disability-Adjusted Life Years)	Kingdom of Saudi Arabia	Both	Age-standardized	Edentulism	Rate	2015	133.8	181.9	86.7
DALYs (Disability-Adjusted Life Years)	Republic of South Africa	Male	Age-standardized	Edentulism	Rate	2015	171.8	246.7	106.5
DALYs (Disability-Adjusted Life Years)	Republic of South Africa	Female	Age-standardized	Edentulism	Rate	2015	167.5	238.1	103.4
DALYs (Disability-Adjusted Life Years)	Republic of South Africa	Both	Age-standardized	Edentulism	Rate	2015	169.4	241.8	105.0
DALYs (Disability-Adjusted Life Years)	Republic of Italy	Male	Age-standardized	Edentulism	Rate	2010	62.3	86.7	39.3
DALYs (Disability-Adjusted Life Years)	Republic of Italy	Female	Age-standardized	Edentulism	Rate	2010	96.1	135.3	60.7

DALYs (Disability-Adjusted Life Years)	Republic of Italy	Both	Age-standardized	Edentulism	Rate	2010	80.6	112.9	50.8
DALYs (Disability-Adjusted Life Years)	Russian Federation	Male	Age-standardized	Edentulism	Rate	2011	135.9	180.6	90.6
DALYs (Disability-Adjusted Life Years)	Russian Federation	Female	Age-standardized	Edentulism	Rate	2011	176.7	235.0	117.4
DALYs (Disability-Adjusted Life Years)	Russian Federation	Both	Age-standardized	Edentulism	Rate	2011	161.3	213.3	107.6
DALYs (Disability-Adjusted Life Years)	Australia	Male	Age-standardized	Edentulism	Rate	2011	123.1	167.5	84.9
DALYs (Disability-Adjusted Life Years)	Australia	Female	Age-standardized	Edentulism	Rate	2011	170.6	234.3	115.6
DALYs (Disability-Adjusted Life Years)	Australia	Both	Age-standardized	Edentulism	Rate	2011	147.9	200.9	101.4
DALYs (Disability-Adjusted Life Years)	Republic of India	Male	Age-standardized	Edentulism	Rate	2015	99.6	138.3	61.4
DALYs (Disability-Adjusted Life Years)	Republic of India	Female	Age-standardized	Edentulism	Rate	2015	112.7	155.0	69.0
DALYs (Disability-Adjusted Life Years)	Republic of India	Both	Age-standardized	Edentulism	Rate	2015	106.1	146.3	65.0
DALYs (Disability-Adjusted Life Years)	European Union	Male	Age-standardized	Edentulism	Rate	2009	85.5	117.6	57.3
DALYs (Disability-Adjusted Life Years)	European Union	Female	Age-standardized	Edentulism	Rate	2009	112.8	156.5	75.9
DALYs (Disability-Adjusted Life Years)	European Union	Both	Age-standardized	Edentulism	Rate	2009	100.4	138.7	67.5
DALYs (Disability-Adjusted Life Years)	Republic of Turkey	Male	Age-standardized	Edentulism	Rate	2011	203.4	282.8	135.2
DALYs (Disability-Adjusted	Republic of Turkey	Female	Age-standardized	Edentulism	Rate	2011	224.0	307.8	150.5

Life Years)									
DALYs (Disability-Adjusted Life Years)	Republic of Turkey	Both	Age-standardized	Edentulism	Rate	2011	214.4	295.9	142.9
DALYs (Disability-Adjusted Life Years)	French Republic	Male	Age-standardized	Edentulism	Rate	2011	59.7	81.8	40.8
DALYs (Disability-Adjusted Life Years)	French Republic	Female	Age-standardized	Edentulism	Rate	2011	83.0	116.5	56.5
DALYs (Disability-Adjusted Life Years)	French Republic	Both	Age-standardized	Edentulism	Rate	2011	72.4	100.2	49.5
DALYs (Disability-Adjusted Life Years)	United Mexican States	Male	Age-standardized	Edentulism	Rate	2011	98.9	132.2	66.2
DALYs (Disability-Adjusted Life Years)	United Mexican States	Female	Age-standardized	Edentulism	Rate	2011	174.7	232.4	116.8
DALYs (Disability-Adjusted Life Years)	United Mexican States	Both	Age-standardized	Edentulism	Rate	2011	139.0	185.2	92.9
DALYs (Disability-Adjusted Life Years)	Republic of Italy	Male	Age-standardized	Edentulism	Rate	2011	65.6	91.1	41.4
DALYs (Disability-Adjusted Life Years)	Republic of Italy	Female	Age-standardized	Edentulism	Rate	2011	99.0	139.3	62.4
DALYs (Disability-Adjusted Life Years)	Republic of Italy	Both	Age-standardized	Edentulism	Rate	2011	83.6	117.3	52.6
DALYs (Disability-Adjusted Life Years)	Republic of Indonesia	Male	Age-standardized	Edentulism	Rate	2011	86.6	116.9	58.1
DALYs (Disability-Adjusted Life Years)	Republic of Indonesia	Female	Age-standardized	Edentulism	Rate	2011	142.0	191.1	94.9
DALYs (Disability-Adjusted Life Years)	Republic of Indonesia	Both	Age-standardized	Edentulism	Rate	2011	115.4	154.9	77.3
DALYs (Disability-Adjusted Life Years)	United Kingdom of Great Britain and Northern Ireland	Male	Age-standardized	Edentulism	Rate	2012	83.4	117.0	55.1
DALYs	United Kingdom of	Female	Age-standardized	Edentulism	Rate	2012	105.7	147.4	69.6

(Disability-Adjusted Life Years)	Great Britain and Northern Ireland								
DALYs (Disability-Adjusted Life Years)	United Kingdom of Great Britain and Northern Ireland	Both	Age-standardized	Edentulism	Rate	2012	95.1	133.1	62.6
DALYs (Disability-Adjusted Life Years)	Republic of Korea	Male	Age-standardized	Edentulism	Rate	2016	62.3	89.4	41.7
DALYs (Disability-Adjusted Life Years)	Republic of Korea	Female	Age-standardized	Edentulism	Rate	2016	62.0	87.0	42.2
DALYs (Disability-Adjusted Life Years)	Republic of Korea	Both	Age-standardized	Edentulism	Rate	2016	63.5	89.5	43.8
DALYs (Disability-Adjusted Life Years)	Federal Republic of Germany	Male	Age-standardized	Edentulism	Rate	2016	93.4	135.2	59.6
DALYs (Disability-Adjusted Life Years)	Federal Republic of Germany	Female	Age-standardized	Edentulism	Rate	2016	119.9	167.6	76.4
DALYs (Disability-Adjusted Life Years)	Federal Republic of Germany	Both	Age-standardized	Edentulism	Rate	2016	107.4	151.4	68.2
DALYs (Disability-Adjusted Life Years)	Canada	Male	Age-standardized	Edentulism	Rate	2012	93.2	127.5	63.3
DALYs (Disability-Adjusted Life Years)	Canada	Female	Age-standardized	Edentulism	Rate	2012	104.3	142.2	71.4
DALYs (Disability-Adjusted Life Years)	Canada	Both	Age-standardized	Edentulism	Rate	2012	99.1	135.7	68.0
DALYs (Disability-Adjusted Life Years)	Federative Republic of Brazil	Male	Age-standardized	Edentulism	Rate	2016	210.9	287.9	139.8
DALYs (Disability-Adjusted Life Years)	Federative Republic of Brazil	Female	Age-standardized	Edentulism	Rate	2016	278.3	375.0	185.6
DALYs (Disability-Adjusted Life Years)	Federative Republic of Brazil	Both	Age-standardized	Edentulism	Rate	2016	247.0	330.2	162.9
DALYs (Disability-Adjusted Life Years)	Republic of India	Male	Age-standardized	Edentulism	Rate	2016	94.3	130.1	59.5

DALYs (Disability-Adjusted Life Years)	Republic of India	Female	Age-standardized	Edentulism	Rate	2016	107.5	148.2	67.1
DALYs (Disability-Adjusted Life Years)	Republic of India	Both	Age-standardized	Edentulism	Rate	2016	100.8	139.5	63.3
DALYs (Disability-Adjusted Life Years)	Canada	Male	Age-standardized	Edentulism	Rate	2011	101.9	140.2	69.0
DALYs (Disability-Adjusted Life Years)	Canada	Female	Age-standardized	Edentulism	Rate	2011	109.7	149.4	75.1
DALYs (Disability-Adjusted Life Years)	Canada	Both	Age-standardized	Edentulism	Rate	2011	106.1	145.0	72.2
DALYs (Disability-Adjusted Life Years)	Kingdom of Saudi Arabia	Male	Age-standardized	Edentulism	Rate	2016	153.8	210.0	100.3
DALYs (Disability-Adjusted Life Years)	Kingdom of Saudi Arabia	Female	Age-standardized	Edentulism	Rate	2016	106.2	145.3	68.4
DALYs (Disability-Adjusted Life Years)	Kingdom of Saudi Arabia	Both	Age-standardized	Edentulism	Rate	2016	134.0	183.0	86.8
DALYs (Disability-Adjusted Life Years)	Republic of South Africa	Male	Age-standardized	Edentulism	Rate	2016	172.0	244.8	106.6
DALYs (Disability-Adjusted Life Years)	Republic of South Africa	Female	Age-standardized	Edentulism	Rate	2016	167.7	237.5	103.9
DALYs (Disability-Adjusted Life Years)	Republic of South Africa	Both	Age-standardized	Edentulism	Rate	2016	169.6	240.7	105.1
DALYs (Disability-Adjusted Life Years)	People's Republic of China	Male	Age-standardized	Edentulism	Rate	2011	73.3	103.6	49.1
DALYs (Disability-Adjusted Life Years)	People's Republic of China	Female	Age-standardized	Edentulism	Rate	2011	88.8	124.3	58.7
DALYs (Disability-Adjusted Life Years)	People's Republic of China	Both	Age-standardized	Edentulism	Rate	2011	81.1	114.8	54.1
DALYs (Disability-Adjusted	United Kingdom of Great Britain and	Male	Age-standardized	Edentulism	Rate	2011	75.3	105.3	49.8

Life Years)	Northern Ireland								
DALYs (Disability-Adjusted Life Years)	United Kingdom of Great Britain and Northern Ireland	Female	Age-standardized	Edentulism	Rate	2011	96.7	134.8	64.1
DALYs (Disability-Adjusted Life Years)	United Kingdom of Great Britain and Northern Ireland	Both	Age-standardized	Edentulism	Rate	2011	86.6	121.4	57.5
DALYs (Disability-Adjusted Life Years)	United States of America	Male	Age-standardized	Edentulism	Rate	2012	100.0	134.6	65.0
DALYs (Disability-Adjusted Life Years)	United States of America	Female	Age-standardized	Edentulism	Rate	2012	108.7	146.3	71.2
DALYs (Disability-Adjusted Life Years)	United States of America	Both	Age-standardized	Edentulism	Rate	2012	104.6	140.9	68.3
DALYs (Disability-Adjusted Life Years)	Argentine Republic	Male	Age-standardized	Edentulism	Rate	2012	86.6	123.6	54.8
DALYs (Disability-Adjusted Life Years)	Argentine Republic	Female	Age-standardized	Edentulism	Rate	2012	138.9	197.2	86.3
DALYs (Disability-Adjusted Life Years)	Argentine Republic	Both	Age-standardized	Edentulism	Rate	2012	115.8	162.7	72.5
DALYs (Disability-Adjusted Life Years)	European Union	Male	Age-standardized	Edentulism	Rate	2011	84.0	115.1	56.4
DALYs (Disability-Adjusted Life Years)	European Union	Female	Age-standardized	Edentulism	Rate	2011	112.0	154.5	75.4
DALYs (Disability-Adjusted Life Years)	European Union	Both	Age-standardized	Edentulism	Rate	2011	99.2	136.5	66.6
DALYs (Disability-Adjusted Life Years)	Argentine Republic	Male	Age-standardized	Edentulism	Rate	2011	87.2	124.7	54.9
DALYs (Disability-Adjusted Life Years)	Argentine Republic	Female	Age-standardized	Edentulism	Rate	2011	140.1	198.3	89.0
DALYs (Disability-Adjusted Life Years)	Argentine Republic	Both	Age-standardized	Edentulism	Rate	2011	116.7	162.9	74.1
DALYs	United States of	Male	Age-standardized	Edentulism	Rate	2013	105.2	141.4	67.8

(Disability-Adjusted Life Years)	America								
DALYs (Disability-Adjusted Life Years)	United States of America	Female	Age-standardized	Edentulism	Rate	2013	114.6	153.9	74.0
DALYs (Disability-Adjusted Life Years)	United States of America	Both	Age-standardized	Edentulism	Rate	2013	110.1	148.0	71.1
DALYs (Disability-Adjusted Life Years)	European Union	Male	Age-standardized	Edentulism	Rate	2012	89.3	122.9	59.9
DALYs (Disability-Adjusted Life Years)	European Union	Female	Age-standardized	Edentulism	Rate	2012	117.3	161.4	79.0
DALYs (Disability-Adjusted Life Years)	European Union	Both	Age-standardized	Edentulism	Rate	2012	104.5	143.5	70.3
DALYs (Disability-Adjusted Life Years)	French Republic	Male	Age-standardized	Edentulism	Rate	2012	65.9	92.9	44.9
DALYs (Disability-Adjusted Life Years)	French Republic	Female	Age-standardized	Edentulism	Rate	2012	91.9	125.7	62.0
DALYs (Disability-Adjusted Life Years)	French Republic	Both	Age-standardized	Edentulism	Rate	2012	80.0	110.7	54.0
DALYs (Disability-Adjusted Life Years)	Japan	Male	Age-standardized	Edentulism	Rate	2013	52.7	73.8	35.1
DALYs (Disability-Adjusted Life Years)	Japan	Female	Age-standardized	Edentulism	Rate	2013	46.7	65.1	32.1
DALYs (Disability-Adjusted Life Years)	Japan	Both	Age-standardized	Edentulism	Rate	2013	49.9	69.6	33.9
DALYs (Disability-Adjusted Life Years)	Republic of Korea	Male	Age-standardized	Edentulism	Rate	2017	57.6	81.4	38.2
DALYs (Disability-Adjusted Life Years)	Republic of Korea	Female	Age-standardized	Edentulism	Rate	2017	56.0	77.6	38.1
DALYs (Disability-Adjusted Life Years)	Republic of Korea	Both	Age-standardized	Edentulism	Rate	2017	57.9	81.4	39.6

DALYs (Disability-Adjusted Life Years)	Federal Republic of Germany	Male	Age-standardized	Edentulism	Rate	2017	93.1	133.5	59.3
DALYs (Disability-Adjusted Life Years)	Federal Republic of Germany	Female	Age-standardized	Edentulism	Rate	2017	119.8	168.0	76.1
DALYs (Disability-Adjusted Life Years)	Federal Republic of Germany	Both	Age-standardized	Edentulism	Rate	2017	107.2	152.2	68.0
DALYs (Disability-Adjusted Life Years)	Japan	Male	Age-standardized	Edentulism	Rate	2012	57.9	81.1	38.3
DALYs (Disability-Adjusted Life Years)	Japan	Female	Age-standardized	Edentulism	Rate	2012	51.1	70.9	34.9
DALYs (Disability-Adjusted Life Years)	Japan	Both	Age-standardized	Edentulism	Rate	2012	54.7	76.3	36.9
DALYs (Disability-Adjusted Life Years)	United Mexican States	Male	Age-standardized	Edentulism	Rate	2013	103.1	136.8	68.4
DALYs (Disability-Adjusted Life Years)	United Mexican States	Female	Age-standardized	Edentulism	Rate	2013	181.4	240.6	120.1
DALYs (Disability-Adjusted Life Years)	United Mexican States	Both	Age-standardized	Edentulism	Rate	2013	144.5	191.6	95.9
DALYs (Disability-Adjusted Life Years)	Australia	Male	Age-standardized	Edentulism	Rate	2012	115.7	158.8	78.4
DALYs (Disability-Adjusted Life Years)	Australia	Female	Age-standardized	Edentulism	Rate	2012	173.2	235.9	116.5
DALYs (Disability-Adjusted Life Years)	Australia	Both	Age-standardized	Edentulism	Rate	2012	145.8	198.3	97.7
DALYs (Disability-Adjusted Life Years)	Federative Republic of Brazil	Male	Age-standardized	Edentulism	Rate	2017	207.9	283.3	137.9
DALYs (Disability-Adjusted Life Years)	Federative Republic of Brazil	Female	Age-standardized	Edentulism	Rate	2017	277.9	373.7	185.0
DALYs (Disability-Adjusted	Federative Republic of Brazil	Both	Age-standardized	Edentulism	Rate	2017	245.4	328.8	161.5

Life Years)									
DALYs (Disability-Adjusted Life Years)	Republic of India	Male	Age-standardized	Edentulism	Rate	2017	87.9	119.8	56.7
DALYs (Disability-Adjusted Life Years)	Republic of India	Female	Age-standardized	Edentulism	Rate	2017	98.7	134.3	63.4
DALYs (Disability-Adjusted Life Years)	Republic of India	Both	Age-standardized	Edentulism	Rate	2017	93.3	127.4	60.1
DALYs (Disability-Adjusted Life Years)	Australia	Male	Age-standardized	Edentulism	Rate	2013	106.4	145.0	70.7
DALYs (Disability-Adjusted Life Years)	Australia	Female	Age-standardized	Edentulism	Rate	2013	175.5	240.3	119.4
DALYs (Disability-Adjusted Life Years)	Australia	Both	Age-standardized	Edentulism	Rate	2013	142.6	195.7	94.9
DALYs (Disability-Adjusted Life Years)	Kingdom of Saudi Arabia	Male	Age-standardized	Edentulism	Rate	2017	154.0	209.2	98.9
DALYs (Disability-Adjusted Life Years)	Kingdom of Saudi Arabia	Female	Age-standardized	Edentulism	Rate	2017	106.3	144.5	68.5
DALYs (Disability-Adjusted Life Years)	Kingdom of Saudi Arabia	Both	Age-standardized	Edentulism	Rate	2017	134.2	182.4	86.1
DALYs (Disability-Adjusted Life Years)	Republic of Turkey	Male	Age-standardized	Edentulism	Rate	2012	203.0	282.8	135.1
DALYs (Disability-Adjusted Life Years)	Republic of Turkey	Female	Age-standardized	Edentulism	Rate	2012	211.1	288.4	140.1
DALYs (Disability-Adjusted Life Years)	Republic of Turkey	Both	Age-standardized	Edentulism	Rate	2012	207.2	285.8	137.9
DALYs (Disability-Adjusted Life Years)	Russian Federation	Male	Age-standardized	Edentulism	Rate	2013	148.5	196.3	99.8
DALYs (Disability-Adjusted Life Years)	Russian Federation	Female	Age-standardized	Edentulism	Rate	2013	181.2	241.0	120.3
DALYs	Russian Federation	Both	Age-standardized	Edentulism	Rate	2013	169.1	224.5	112.6

(Disability-Adjusted Life Years)									
DALYs (Disability-Adjusted Life Years)	Republic of Turkey	Male	Age-standardized	Edentulism	Rate	2013	202.8	278.3	135.0
DALYs (Disability-Adjusted Life Years)	Republic of Turkey	Female	Age-standardized	Edentulism	Rate	2013	196.6	269.5	130.3
DALYs (Disability-Adjusted Life Years)	Republic of Turkey	Both	Age-standardized	Edentulism	Rate	2013	199.2	273.0	131.9
DALYs (Disability-Adjusted Life Years)	Russian Federation	Male	Age-standardized	Edentulism	Rate	2012	141.6	187.4	94.9
DALYs (Disability-Adjusted Life Years)	Russian Federation	Female	Age-standardized	Edentulism	Rate	2012	178.7	237.4	118.6
DALYs (Disability-Adjusted Life Years)	Russian Federation	Both	Age-standardized	Edentulism	Rate	2012	164.8	217.6	110.0
DALYs (Disability-Adjusted Life Years)	Republic of South Africa	Male	Age-standardized	Edentulism	Rate	2017	172.0	245.5	106.4
DALYs (Disability-Adjusted Life Years)	Republic of South Africa	Female	Age-standardized	Edentulism	Rate	2017	167.8	235.5	103.6
DALYs (Disability-Adjusted Life Years)	Republic of South Africa	Both	Age-standardized	Edentulism	Rate	2017	169.7	239.8	104.8
DALYs (Disability-Adjusted Life Years)	French Republic	Male	Age-standardized	Edentulism	Rate	2013	73.5	102.3	48.7
DALYs (Disability-Adjusted Life Years)	French Republic	Female	Age-standardized	Edentulism	Rate	2013	102.1	142.6	68.5
DALYs (Disability-Adjusted Life Years)	French Republic	Both	Age-standardized	Edentulism	Rate	2013	88.9	123.5	59.4
DALYs (Disability-Adjusted Life Years)	Republic of Italy	Male	Age-standardized	Edentulism	Rate	2012	73.2	101.6	46.4
DALYs (Disability-Adjusted Life Years)	Republic of Italy	Female	Age-standardized	Edentulism	Rate	2012	105.0	148.0	66.3

DALYs (Disability-Adjusted Life Years)	Republic of Italy	Both	Age-standardized	Edentulism	Rate	2012	90.3	127.1	57.0
DALYs (Disability-Adjusted Life Years)	Republic of Italy	Male	Age-standardized	Edentulism	Rate	2013	82.4	115.6	52.1
DALYs (Disability-Adjusted Life Years)	Republic of Italy	Female	Age-standardized	Edentulism	Rate	2013	111.9	158.0	70.8
DALYs (Disability-Adjusted Life Years)	Republic of Italy	Both	Age-standardized	Edentulism	Rate	2013	98.2	138.7	62.0
DALYs (Disability-Adjusted Life Years)	Republic of Indonesia	Male	Age-standardized	Edentulism	Rate	2013	85.9	117.2	57.4
DALYs (Disability-Adjusted Life Years)	Republic of Indonesia	Female	Age-standardized	Edentulism	Rate	2013	140.5	188.9	93.1
DALYs (Disability-Adjusted Life Years)	Republic of Indonesia	Both	Age-standardized	Edentulism	Rate	2013	114.2	153.8	75.9
DALYs (Disability-Adjusted Life Years)	United Mexican States	Male	Age-standardized	Edentulism	Rate	2014	105.7	139.9	69.9
DALYs (Disability-Adjusted Life Years)	United Mexican States	Female	Age-standardized	Edentulism	Rate	2014	185.6	246.5	122.6
DALYs (Disability-Adjusted Life Years)	United Mexican States	Both	Age-standardized	Edentulism	Rate	2014	148.0	196.3	97.9
DALYs (Disability-Adjusted Life Years)	People's Republic of China	Male	Age-standardized	Edentulism	Rate	2012	68.6	97.1	46.0
DALYs (Disability-Adjusted Life Years)	People's Republic of China	Female	Age-standardized	Edentulism	Rate	2012	83.5	117.1	55.4
DALYs (Disability-Adjusted Life Years)	People's Republic of China	Both	Age-standardized	Edentulism	Rate	2012	76.1	107.1	50.8
DALYs (Disability-Adjusted Life Years)	Canada	Male	Age-standardized	Edentulism	Rate	2013	82.9	112.9	55.3
DALYs (Disability-Adjusted	Canada	Female	Age-standardized	Edentulism	Rate	2013	97.7	133.0	65.7

Life Years)									
DALYs (Disability-Adjusted Life Years)	Canada	Both	Age-standardized	Edentulism	Rate	2013	90.7	123.7	61.0
DALYs (Disability-Adjusted Life Years)	United Kingdom of Great Britain and Northern Ireland	Male	Age-standardized	Edentulism	Rate	2013	93.2	130.0	61.6
DALYs (Disability-Adjusted Life Years)	United Kingdom of Great Britain and Northern Ireland	Female	Age-standardized	Edentulism	Rate	2013	116.5	162.1	76.7
DALYs (Disability-Adjusted Life Years)	United Kingdom of Great Britain and Northern Ireland	Both	Age-standardized	Edentulism	Rate	2013	105.4	146.4	69.5
DALYs (Disability-Adjusted Life Years)	People's Republic of China	Male	Age-standardized	Edentulism	Rate	2013	63.0	88.9	42.5
DALYs (Disability-Adjusted Life Years)	People's Republic of China	Female	Age-standardized	Edentulism	Rate	2013	77.2	108.6	51.1
DALYs (Disability-Adjusted Life Years)	People's Republic of China	Both	Age-standardized	Edentulism	Rate	2013	70.2	98.7	46.9
DALYs (Disability-Adjusted Life Years)	Argentine Republic	Male	Age-standardized	Edentulism	Rate	2013	86.1	121.6	54.3
DALYs (Disability-Adjusted Life Years)	Argentine Republic	Female	Age-standardized	Edentulism	Rate	2013	137.8	195.7	86.2
DALYs (Disability-Adjusted Life Years)	Argentine Republic	Both	Age-standardized	Edentulism	Rate	2013	114.9	162.3	71.8
DALYs (Disability-Adjusted Life Years)	Federal Republic of Germany	Male	Age-standardized	Edentulism	Rate	2018	92.6	130.0	59.2
DALYs (Disability-Adjusted Life Years)	Federal Republic of Germany	Female	Age-standardized	Edentulism	Rate	2018	119.6	169.5	75.4
DALYs (Disability-Adjusted Life Years)	Federal Republic of Germany	Both	Age-standardized	Edentulism	Rate	2018	106.8	150.7	67.2
DALYs (Disability-Adjusted Life Years)	Republic of Indonesia	Male	Age-standardized	Edentulism	Rate	2014	85.6	116.1	57.1
DALYs	Republic of	Female	Age-standardized	Edentulism	Rate	2014	139.7	188.9	92.6

(Disability-Adjusted Life Years)	Indonesia								
DALYs (Disability-Adjusted Life Years)	Republic of Indonesia	Both	Age-standardized	Edentulism	Rate	2014	113.6	153.3	75.5
DALYs (Disability-Adjusted Life Years)	United States of America	Male	Age-standardized	Edentulism	Rate	2014	110.2	148.5	70.3
DALYs (Disability-Adjusted Life Years)	United States of America	Female	Age-standardized	Edentulism	Rate	2014	120.3	161.0	77.2
DALYs (Disability-Adjusted Life Years)	United States of America	Both	Age-standardized	Edentulism	Rate	2014	115.4	155.0	73.9
DALYs (Disability-Adjusted Life Years)	Republic of India	Male	Age-standardized	Edentulism	Rate	2018	82.4	112.2	54.3
DALYs (Disability-Adjusted Life Years)	Republic of India	Female	Age-standardized	Edentulism	Rate	2018	90.3	121.8	59.6
DALYs (Disability-Adjusted Life Years)	Republic of India	Both	Age-standardized	Edentulism	Rate	2018	86.4	117.4	57.0
DALYs (Disability-Adjusted Life Years)	Kingdom of Saudi Arabia	Male	Age-standardized	Edentulism	Rate	2018	154.1	209.8	99.0
DALYs (Disability-Adjusted Life Years)	Kingdom of Saudi Arabia	Female	Age-standardized	Edentulism	Rate	2018	106.4	144.0	67.8
DALYs (Disability-Adjusted Life Years)	Kingdom of Saudi Arabia	Both	Age-standardized	Edentulism	Rate	2018	134.3	181.4	86.2
DALYs (Disability-Adjusted Life Years)	United Kingdom of Great Britain and Northern Ireland	Male	Age-standardized	Edentulism	Rate	2014	101.5	141.1	67.2
DALYs (Disability-Adjusted Life Years)	United Kingdom of Great Britain and Northern Ireland	Female	Age-standardized	Edentulism	Rate	2014	125.8	174.2	83.2
DALYs (Disability-Adjusted Life Years)	United Kingdom of Great Britain and Northern Ireland	Both	Age-standardized	Edentulism	Rate	2014	114.2	158.2	75.5
DALYs (Disability-Adjusted Life Years)	Federative Republic of Brazil	Male	Age-standardized	Edentulism	Rate	2018	205.0	277.5	136.0

DALYs (Disability-Adjusted Life Years)	Federative Republic of Brazil	Female	Age-standardized	Edentulism	Rate	2018	277.3	373.7	184.4
DALYs (Disability-Adjusted Life Years)	Federative Republic of Brazil	Both	Age-standardized	Edentulism	Rate	2018	243.8	327.0	160.4
DALYs (Disability-Adjusted Life Years)	Republic of Korea	Male	Age-standardized	Edentulism	Rate	2018	52.7	73.9	35.1
DALYs (Disability-Adjusted Life Years)	Republic of Korea	Female	Age-standardized	Edentulism	Rate	2018	50.2	70.6	33.7
DALYs (Disability-Adjusted Life Years)	Republic of Korea	Both	Age-standardized	Edentulism	Rate	2018	52.3	73.3	35.3
DALYs (Disability-Adjusted Life Years)	Republic of South Africa	Male	Age-standardized	Edentulism	Rate	2018	171.9	244.5	105.9
DALYs (Disability-Adjusted Life Years)	Republic of South Africa	Female	Age-standardized	Edentulism	Rate	2018	167.8	232.3	104.0
DALYs (Disability-Adjusted Life Years)	Republic of South Africa	Both	Age-standardized	Edentulism	Rate	2018	169.6	237.7	104.9
DALYs (Disability-Adjusted Life Years)	Argentine Republic	Male	Age-standardized	Edentulism	Rate	2014	85.7	120.1	55.0
DALYs (Disability-Adjusted Life Years)	Argentine Republic	Female	Age-standardized	Edentulism	Rate	2014	136.9	194.2	85.4
DALYs (Disability-Adjusted Life Years)	Argentine Republic	Both	Age-standardized	Edentulism	Rate	2014	114.2	161.9	72.1
DALYs (Disability-Adjusted Life Years)	Canada	Male	Age-standardized	Edentulism	Rate	2014	74.4	102.2	48.6
DALYs (Disability-Adjusted Life Years)	Canada	Female	Age-standardized	Edentulism	Rate	2014	92.4	125.3	60.8
DALYs (Disability-Adjusted Life Years)	Canada	Both	Age-standardized	Edentulism	Rate	2014	83.8	114.3	54.8
DALYs (Disability-Adjusted	European Union	Male	Age-standardized	Edentulism	Rate	2013	95.9	132.5	63.7

Life Years)									
DALYs (Disability-Adjusted Life Years)	European Union	Female	Age-standardized	Edentulism	Rate	2013	123.6	170.7	83.2
DALYs (Disability-Adjusted Life Years)	European Union	Both	Age-standardized	Edentulism	Rate	2013	110.9	153.3	74.1
DALYs (Disability-Adjusted Life Years)	Japan	Male	Age-standardized	Edentulism	Rate	2014	49.1	68.8	32.9
DALYs (Disability-Adjusted Life Years)	Japan	Female	Age-standardized	Edentulism	Rate	2014	43.4	60.6	29.8
DALYs (Disability-Adjusted Life Years)	Japan	Both	Age-standardized	Edentulism	Rate	2014	46.4	64.7	31.5
DALYs (Disability-Adjusted Life Years)	People's Republic of China	Male	Age-standardized	Edentulism	Rate	2014	58.4	82.3	39.3
DALYs (Disability-Adjusted Life Years)	People's Republic of China	Female	Age-standardized	Edentulism	Rate	2014	72.0	100.6	47.6
DALYs (Disability-Adjusted Life Years)	People's Republic of China	Both	Age-standardized	Edentulism	Rate	2014	65.3	91.3	43.6
DALYs (Disability-Adjusted Life Years)	Russian Federation	Male	Age-standardized	Edentulism	Rate	2014	154.5	204.2	103.5
DALYs (Disability-Adjusted Life Years)	Russian Federation	Female	Age-standardized	Edentulism	Rate	2014	183.4	243.1	122.1
DALYs (Disability-Adjusted Life Years)	Russian Federation	Both	Age-standardized	Edentulism	Rate	2014	172.8	229.4	114.8
DALYs (Disability-Adjusted Life Years)	United States of America	Male	Age-standardized	Edentulism	Rate	2015	113.8	152.8	72.2
DALYs (Disability-Adjusted Life Years)	United States of America	Female	Age-standardized	Edentulism	Rate	2015	124.3	167.2	78.9
DALYs (Disability-Adjusted Life Years)	United States of America	Both	Age-standardized	Edentulism	Rate	2015	119.2	159.8	75.6
DALYs	European Union	Male	Age-standardized	Edentulism	Rate	2014	101.3	140.4	66.4

(Disability-Adjusted Life Years)									
DALYs (Disability-Adjusted Life Years)	European Union	Female	Age-standardized	Edentulism	Rate	2014	128.8	178.4	84.8
DALYs (Disability-Adjusted Life Years)	European Union	Both	Age-standardized	Edentulism	Rate	2014	116.1	161.0	75.9
DALYs (Disability-Adjusted Life Years)	Republic of Korea	Male	Age-standardized	Edentulism	Rate	2019	50.5	71.5	33.5
DALYs (Disability-Adjusted Life Years)	Republic of Korea	Female	Age-standardized	Edentulism	Rate	2019	47.4	67.4	30.8
DALYs (Disability-Adjusted Life Years)	Republic of Korea	Both	Age-standardized	Edentulism	Rate	2019	49.7	70.2	32.7
DALYs (Disability-Adjusted Life Years)	French Republic	Male	Age-standardized	Edentulism	Rate	2014	79.8	113.1	51.1
DALYs (Disability-Adjusted Life Years)	French Republic	Female	Age-standardized	Edentulism	Rate	2014	110.7	154.7	71.4
DALYs (Disability-Adjusted Life Years)	French Republic	Both	Age-standardized	Edentulism	Rate	2014	96.5	135.2	61.9
DALYs (Disability-Adjusted Life Years)	Australia	Male	Age-standardized	Edentulism	Rate	2014	98.7	135.6	63.6
DALYs (Disability-Adjusted Life Years)	Australia	Female	Age-standardized	Edentulism	Rate	2014	177.0	241.6	116.3
DALYs (Disability-Adjusted Life Years)	Australia	Both	Age-standardized	Edentulism	Rate	2014	139.8	191.2	92.4
DALYs (Disability-Adjusted Life Years)	Federal Republic of Germany	Male	Age-standardized	Edentulism	Rate	2019	92.5	130.7	59.5
DALYs (Disability-Adjusted Life Years)	Federal Republic of Germany	Female	Age-standardized	Edentulism	Rate	2019	119.3	169.8	75.2
DALYs (Disability-Adjusted Life Years)	Federal Republic of Germany	Both	Age-standardized	Edentulism	Rate	2019	106.6	151.6	67.6

DALYs (Disability-Adjusted Life Years)	United Mexican States	Male	Age-standardized	Edentulism	Rate	2016	111.4	147.6	73.0
DALYs (Disability-Adjusted Life Years)	United Mexican States	Female	Age-standardized	Edentulism	Rate	2016	194.6	259.4	128.2
DALYs (Disability-Adjusted Life Years)	United Mexican States	Both	Age-standardized	Edentulism	Rate	2016	155.5	206.8	102.4
DALYs (Disability-Adjusted Life Years)	Federative Republic of Brazil	Male	Age-standardized	Edentulism	Rate	2019	203.5	273.1	134.8
DALYs (Disability-Adjusted Life Years)	Federative Republic of Brazil	Female	Age-standardized	Edentulism	Rate	2019	277.0	373.7	184.2
DALYs (Disability-Adjusted Life Years)	Federative Republic of Brazil	Both	Age-standardized	Edentulism	Rate	2019	242.9	326.0	159.8
DALYs (Disability-Adjusted Life Years)	Japan	Male	Age-standardized	Edentulism	Rate	2015	47.8	67.1	32.3
DALYs (Disability-Adjusted Life Years)	Japan	Female	Age-standardized	Edentulism	Rate	2015	42.0	58.6	28.9
DALYs (Disability-Adjusted Life Years)	Japan	Both	Age-standardized	Edentulism	Rate	2015	45.1	62.8	30.7
DALYs (Disability-Adjusted Life Years)	Republic of Turkey	Male	Age-standardized	Edentulism	Rate	2014	202.6	275.7	132.9
DALYs (Disability-Adjusted Life Years)	Republic of Turkey	Female	Age-standardized	Edentulism	Rate	2014	184.7	252.0	120.1
DALYs (Disability-Adjusted Life Years)	Republic of Turkey	Both	Age-standardized	Edentulism	Rate	2014	192.7	262.5	125.3
DALYs (Disability-Adjusted Life Years)	Kingdom of Saudi Arabia	Male	Age-standardized	Edentulism	Rate	2019	154.2	210.0	97.8
DALYs (Disability-Adjusted Life Years)	Kingdom of Saudi Arabia	Female	Age-standardized	Edentulism	Rate	2019	106.6	145.3	68.9
DALYs (Disability-Adjusted	Kingdom of Saudi Arabia	Both	Age-standardized	Edentulism	Rate	2019	134.5	183.9	85.7

Life Years)									
DALYs (Disability-Adjusted Life Years)	Republic of South Africa	Male	Age-standardized	Edentulism	Rate	2019	172.0	242.6	105.3
DALYs (Disability-Adjusted Life Years)	Republic of South Africa	Female	Age-standardized	Edentulism	Rate	2019	167.8	231.2	104.8
DALYs (Disability-Adjusted Life Years)	Republic of South Africa	Both	Age-standardized	Edentulism	Rate	2019	169.6	236.2	105.1
DALYs (Disability-Adjusted Life Years)	United Mexican States	Male	Age-standardized	Edentulism	Rate	2015	108.2	143.2	71.0
DALYs (Disability-Adjusted Life Years)	United Mexican States	Female	Age-standardized	Edentulism	Rate	2015	189.5	252.0	124.2
DALYs (Disability-Adjusted Life Years)	United Mexican States	Both	Age-standardized	Edentulism	Rate	2015	151.2	200.7	99.7
DALYs (Disability-Adjusted Life Years)	Russian Federation	Male	Age-standardized	Edentulism	Rate	2015	157.4	209.2	104.9
DALYs (Disability-Adjusted Life Years)	Russian Federation	Female	Age-standardized	Edentulism	Rate	2015	184.5	244.1	123.4
DALYs (Disability-Adjusted Life Years)	Russian Federation	Both	Age-standardized	Edentulism	Rate	2015	174.6	231.6	116.1
DALYs (Disability-Adjusted Life Years)	Republic of India	Male	Age-standardized	Edentulism	Rate	2019	80.0	108.1	53.4
DALYs (Disability-Adjusted Life Years)	Republic of India	Female	Age-standardized	Edentulism	Rate	2019	86.2	116.3	58.1
DALYs (Disability-Adjusted Life Years)	Republic of India	Both	Age-standardized	Edentulism	Rate	2019	83.2	112.2	55.8
DALYs (Disability-Adjusted Life Years)	Republic of Turkey	Male	Age-standardized	Edentulism	Rate	2015	202.2	275.3	129.0
DALYs (Disability-Adjusted Life Years)	Republic of Turkey	Female	Age-standardized	Edentulism	Rate	2015	179.3	243.6	113.1
DALYs	Republic of Turkey	Both	Age-standardized	Edentulism	Rate	2015	189.6	258.6	120.8

(Disability-Adjusted Life Years)									
DALYs (Disability-Adjusted Life Years)	Republic of Indonesia	Male	Age-standardized	Edentulism	Rate	2015	85.2	115.4	56.9
DALYs (Disability-Adjusted Life Years)	Republic of Indonesia	Female	Age-standardized	Edentulism	Rate	2015	139.0	187.6	92.1
DALYs (Disability-Adjusted Life Years)	Republic of Indonesia	Both	Age-standardized	Edentulism	Rate	2015	113.0	152.6	75.1
DALYs (Disability-Adjusted Life Years)	People's Republic of China	Male	Age-standardized	Edentulism	Rate	2015	56.4	79.3	38.2
DALYs (Disability-Adjusted Life Years)	People's Republic of China	Female	Age-standardized	Edentulism	Rate	2015	69.9	97.3	46.1
DALYs (Disability-Adjusted Life Years)	People's Republic of China	Both	Age-standardized	Edentulism	Rate	2015	63.2	88.1	42.3
DALYs (Disability-Adjusted Life Years)	United Kingdom of Great Britain and Northern Ireland	Male	Age-standardized	Edentulism	Rate	2016	105.3	146.7	70.1
DALYs (Disability-Adjusted Life Years)	United Kingdom of Great Britain and Northern Ireland	Female	Age-standardized	Edentulism	Rate	2016	130.3	180.5	86.5
DALYs (Disability-Adjusted Life Years)	United Kingdom of Great Britain and Northern Ireland	Both	Age-standardized	Edentulism	Rate	2016	118.3	164.3	78.6
DALYs (Disability-Adjusted Life Years)	Republic of Korea	Male	Age-standardized	Edentulism	Rate	2020	50.6	71.9	32.7
DALYs (Disability-Adjusted Life Years)	Republic of Korea	Female	Age-standardized	Edentulism	Rate	2020	47.4	67.0	31.2
DALYs (Disability-Adjusted Life Years)	Republic of Korea	Both	Age-standardized	Edentulism	Rate	2020	49.7	70.2	33.1
DALYs (Disability-Adjusted Life Years)	United Kingdom of Great Britain and Northern Ireland	Male	Age-standardized	Edentulism	Rate	2015	105.3	146.6	70.3
DALYs (Disability-Adjusted Life Years)	United Kingdom of Great Britain and Northern Ireland	Female	Age-standardized	Edentulism	Rate	2015	130.1	179.8	86.5

DALYs (Disability-Adjusted Life Years)	United Kingdom of Great Britain and Northern Ireland	Both	Age-standardized	Edentulism	Rate	2015	118.2	163.8	78.7
DALYs (Disability-Adjusted Life Years)	Federal Republic of Germany	Male	Age-standardized	Edentulism	Rate	2020	92.6	131.7	60.6
DALYs (Disability-Adjusted Life Years)	Federal Republic of Germany	Female	Age-standardized	Edentulism	Rate	2020	119.0	167.9	75.6
DALYs (Disability-Adjusted Life Years)	Federal Republic of Germany	Both	Age-standardized	Edentulism	Rate	2020	106.5	150.9	68.6
DALYs (Disability-Adjusted Life Years)	French Republic	Male	Age-standardized	Edentulism	Rate	2015	82.3	116.6	52.3
DALYs (Disability-Adjusted Life Years)	French Republic	Female	Age-standardized	Edentulism	Rate	2015	114.2	163.0	73.5
DALYs (Disability-Adjusted Life Years)	French Republic	Both	Age-standardized	Edentulism	Rate	2015	99.5	139.7	63.9
DALYs (Disability-Adjusted Life Years)	Canada	Male	Age-standardized	Edentulism	Rate	2015	71.0	98.9	45.5
DALYs (Disability-Adjusted Life Years)	Canada	Female	Age-standardized	Edentulism	Rate	2015	89.7	123.4	57.4
DALYs (Disability-Adjusted Life Years)	Canada	Both	Age-standardized	Edentulism	Rate	2015	80.8	110.9	51.8
DALYs (Disability-Adjusted Life Years)	Federative Republic of Brazil	Male	Age-standardized	Edentulism	Rate	2020	203.2	273.7	135.6
DALYs (Disability-Adjusted Life Years)	Federative Republic of Brazil	Female	Age-standardized	Edentulism	Rate	2020	276.1	373.5	181.6
DALYs (Disability-Adjusted Life Years)	Federative Republic of Brazil	Both	Age-standardized	Edentulism	Rate	2020	242.3	324.6	159.6
DALYs (Disability-Adjusted Life Years)	Kingdom of Saudi Arabia	Male	Age-standardized	Edentulism	Rate	2020	154.2	210.9	100.5
DALYs (Disability-Adjusted	Kingdom of Saudi Arabia	Female	Age-standardized	Edentulism	Rate	2020	106.6	144.8	67.8

Life Years)									
DALYs (Disability-Adjusted Life Years)	Kingdom of Saudi Arabia	Both	Age-standardized	Edentulism	Rate	2020	134.7	183.4	87.6
DALYs (Disability-Adjusted Life Years)	Republic of Italy	Male	Age-standardized	Edentulism	Rate	2014	90.1	126.3	57.3
DALYs (Disability-Adjusted Life Years)	Republic of Italy	Female	Age-standardized	Edentulism	Rate	2014	117.7	167.0	73.8
DALYs (Disability-Adjusted Life Years)	Republic of Italy	Both	Age-standardized	Edentulism	Rate	2014	104.8	148.7	66.0
DALYs (Disability-Adjusted Life Years)	Republic of India	Male	Age-standardized	Edentulism	Rate	2020	80.1	107.3	54.0
DALYs (Disability-Adjusted Life Years)	Republic of India	Female	Age-standardized	Edentulism	Rate	2020	85.6	114.3	58.1
DALYs (Disability-Adjusted Life Years)	Republic of India	Both	Age-standardized	Edentulism	Rate	2020	82.9	110.8	56.1
DALYs (Disability-Adjusted Life Years)	Argentine Republic	Male	Age-standardized	Edentulism	Rate	2016	85.5	121.5	54.6
DALYs (Disability-Adjusted Life Years)	Argentine Republic	Female	Age-standardized	Edentulism	Rate	2016	136.6	194.2	84.5
DALYs (Disability-Adjusted Life Years)	Argentine Republic	Both	Age-standardized	Edentulism	Rate	2016	113.9	161.7	71.6
DALYs (Disability-Adjusted Life Years)	Republic of Italy	Male	Age-standardized	Edentulism	Rate	2015	93.4	131.8	59.4
DALYs (Disability-Adjusted Life Years)	Republic of Italy	Female	Age-standardized	Edentulism	Rate	2015	120.2	170.8	75.3
DALYs (Disability-Adjusted Life Years)	Republic of Italy	Both	Age-standardized	Edentulism	Rate	2015	107.7	152.8	67.8
DALYs (Disability-Adjusted Life Years)	Republic of South Africa	Male	Age-standardized	Edentulism	Rate	2020	171.9	244.4	106.3
DALYs	Republic of South	Female	Age-standardized	Edentulism	Rate	2020	167.4	236.9	102.2

(Disability-Adjusted Life Years)	Africa								
DALYs (Disability-Adjusted Life Years)	Republic of South Africa	Both	Age-standardized	Edentulism	Rate	2020	169.4	242.0	104.2
DALYs (Disability-Adjusted Life Years)	Canada	Male	Age-standardized	Edentulism	Rate	2016	71.0	98.9	45.1
DALYs (Disability-Adjusted Life Years)	Canada	Female	Age-standardized	Edentulism	Rate	2016	89.2	123.2	56.4
DALYs (Disability-Adjusted Life Years)	Canada	Both	Age-standardized	Edentulism	Rate	2016	80.5	111.9	50.8
DALYs (Disability-Adjusted Life Years)	United States of America	Male	Age-standardized	Edentulism	Rate	2016	118.1	158.4	75.3
DALYs (Disability-Adjusted Life Years)	United States of America	Female	Age-standardized	Edentulism	Rate	2016	129.2	172.4	82.2
DALYs (Disability-Adjusted Life Years)	United States of America	Both	Age-standardized	Edentulism	Rate	2016	123.8	165.4	78.9
DALYs (Disability-Adjusted Life Years)	Argentine Republic	Male	Age-standardized	Edentulism	Rate	2015	85.5	121.1	54.8
DALYs (Disability-Adjusted Life Years)	Argentine Republic	Female	Age-standardized	Edentulism	Rate	2015	136.4	195.9	84.1
DALYs (Disability-Adjusted Life Years)	Argentine Republic	Both	Age-standardized	Edentulism	Rate	2015	113.8	160.5	71.4
DALYs (Disability-Adjusted Life Years)	United States of America	Male	Age-standardized	Edentulism	Rate	2017	124.7	167.1	79.6
DALYs (Disability-Adjusted Life Years)	United States of America	Female	Age-standardized	Edentulism	Rate	2017	136.8	182.3	87.2
DALYs (Disability-Adjusted Life Years)	United States of America	Both	Age-standardized	Edentulism	Rate	2017	131.0	174.9	83.6
DALYs (Disability-Adjusted Life Years)	Russian Federation	Male	Age-standardized	Edentulism	Rate	2016	158.1	210.2	105.1

DALYs (Disability-Adjusted Life Years)	Russian Federation	Female	Age-standardized	Edentulism	Rate	2016	185.3	246.0	124.4
DALYs (Disability-Adjusted Life Years)	Russian Federation	Both	Age-standardized	Edentulism	Rate	2016	175.3	233.0	117.2
DALYs (Disability-Adjusted Life Years)	Australia	Male	Age-standardized	Edentulism	Rate	2016	101.0	140.2	64.8
DALYs (Disability-Adjusted Life Years)	Australia	Female	Age-standardized	Edentulism	Rate	2016	177.5	246.1	114.1
DALYs (Disability-Adjusted Life Years)	Australia	Both	Age-standardized	Edentulism	Rate	2016	141.1	194.6	91.3
DALYs (Disability-Adjusted Life Years)	Republic of Turkey	Male	Age-standardized	Edentulism	Rate	2016	201.8	275.3	129.0
DALYs (Disability-Adjusted Life Years)	Republic of Turkey	Female	Age-standardized	Edentulism	Rate	2016	178.5	243.2	114.0
DALYs (Disability-Adjusted Life Years)	Republic of Turkey	Both	Age-standardized	Edentulism	Rate	2016	189.0	256.1	120.1
DALYs (Disability-Adjusted Life Years)	European Union	Male	Age-standardized	Edentulism	Rate	2015	103.5	143.6	67.0
DALYs (Disability-Adjusted Life Years)	European Union	Female	Age-standardized	Edentulism	Rate	2015	131.0	180.6	84.5
DALYs (Disability-Adjusted Life Years)	European Union	Both	Age-standardized	Edentulism	Rate	2015	118.3	164.2	76.4
DALYs (Disability-Adjusted Life Years)	Japan	Male	Age-standardized	Edentulism	Rate	2016	48.7	68.1	32.7
DALYs (Disability-Adjusted Life Years)	Japan	Female	Age-standardized	Edentulism	Rate	2016	42.2	58.8	28.9
DALYs (Disability-Adjusted Life Years)	Japan	Both	Age-standardized	Edentulism	Rate	2016	45.6	63.6	30.9
DALYs (Disability-Adjusted	Republic of Italy	Male	Age-standardized	Edentulism	Rate	2016	93.5	132.3	59.4

Life Years)									
DALYs (Disability-Adjusted Life Years)	Republic of Italy	Female	Age-standardized	Edentulism	Rate	2016	120.3	170.5	75.3
DALYs (Disability-Adjusted Life Years)	Republic of Italy	Both	Age-standardized	Edentulism	Rate	2016	107.7	152.7	67.8
DALYs (Disability-Adjusted Life Years)	European Union	Male	Age-standardized	Edentulism	Rate	2016	103.4	143.9	67.1
DALYs (Disability-Adjusted Life Years)	European Union	Female	Age-standardized	Edentulism	Rate	2016	131.0	181.3	84.2
DALYs (Disability-Adjusted Life Years)	European Union	Both	Age-standardized	Edentulism	Rate	2016	118.3	164.3	76.3
DALYs (Disability-Adjusted Life Years)	United Mexican States	Male	Age-standardized	Edentulism	Rate	2017	115.7	153.8	75.8
DALYs (Disability-Adjusted Life Years)	United Mexican States	Female	Age-standardized	Edentulism	Rate	2017	201.4	267.9	133.1
DALYs (Disability-Adjusted Life Years)	United Mexican States	Both	Age-standardized	Edentulism	Rate	2017	161.1	214.2	106.3
DALYs (Disability-Adjusted Life Years)	People's Republic of China	Male	Age-standardized	Edentulism	Rate	2016	59.4	83.6	40.3
DALYs (Disability-Adjusted Life Years)	People's Republic of China	Female	Age-standardized	Edentulism	Rate	2016	73.5	102.4	48.8
DALYs (Disability-Adjusted Life Years)	People's Republic of China	Both	Age-standardized	Edentulism	Rate	2016	66.6	93.1	44.6
DALYs (Disability-Adjusted Life Years)	French Republic	Male	Age-standardized	Edentulism	Rate	2016	82.2	116.9	51.9
DALYs (Disability-Adjusted Life Years)	French Republic	Female	Age-standardized	Edentulism	Rate	2016	114.2	162.2	72.8
DALYs (Disability-Adjusted Life Years)	French Republic	Both	Age-standardized	Edentulism	Rate	2016	99.4	140.7	63.1
DALYs	Federative	Male	Age-standardized	Edentulism	Rate	2021	202.6	272.7	134.1

(Disability-Adjusted Life Years)	Republic of Brazil								
DALYs (Disability-Adjusted Life Years)	Federative Republic of Brazil	Female	Age-standardized	Edentulism	Rate	2021	275.8	371.5	183.5
DALYs (Disability-Adjusted Life Years)	Federative Republic of Brazil	Both	Age-standardized	Edentulism	Rate	2021	241.9	323.8	160.1
DALYs (Disability-Adjusted Life Years)	Federal Republic of Germany	Male	Age-standardized	Edentulism	Rate	2021	92.9	132.0	58.0
DALYs (Disability-Adjusted Life Years)	Federal Republic of Germany	Female	Age-standardized	Edentulism	Rate	2021	119.0	166.7	75.8
DALYs (Disability-Adjusted Life Years)	Federal Republic of Germany	Both	Age-standardized	Edentulism	Rate	2021	106.6	150.8	67.2
DALYs (Disability-Adjusted Life Years)	Republic of Korea	Male	Age-standardized	Edentulism	Rate	2021	50.7	71.5	32.9
DALYs (Disability-Adjusted Life Years)	Republic of Korea	Female	Age-standardized	Edentulism	Rate	2021	47.2	67.1	31.3
DALYs (Disability-Adjusted Life Years)	Republic of Korea	Both	Age-standardized	Edentulism	Rate	2021	49.6	70.4	33.5
DALYs (Disability-Adjusted Life Years)	Kingdom of Saudi Arabia	Male	Age-standardized	Edentulism	Rate	2021	155.2	213.2	97.9
DALYs (Disability-Adjusted Life Years)	Kingdom of Saudi Arabia	Female	Age-standardized	Edentulism	Rate	2021	106.8	145.4	68.3
DALYs (Disability-Adjusted Life Years)	Kingdom of Saudi Arabia	Both	Age-standardized	Edentulism	Rate	2021	135.5	184.5	85.1
DALYs (Disability-Adjusted Life Years)	Republic of South Africa	Male	Age-standardized	Edentulism	Rate	2021	170.8	242.1	103.9
DALYs (Disability-Adjusted Life Years)	Republic of South Africa	Female	Age-standardized	Edentulism	Rate	2021	166.7	235.6	104.3
DALYs (Disability-Adjusted Life Years)	Republic of South Africa	Both	Age-standardized	Edentulism	Rate	2021	168.5	240.2	104.3

DALYs (Disability-Adjusted Life Years)	Republic of Indonesia	Male	Age-standardized	Edentulism	Rate	2016	84.8	114.9	56.4
DALYs (Disability-Adjusted Life Years)	Republic of Indonesia	Female	Age-standardized	Edentulism	Rate	2016	138.4	187.6	91.2
DALYs (Disability-Adjusted Life Years)	Republic of Indonesia	Both	Age-standardized	Edentulism	Rate	2016	112.5	151.8	74.3
DALYs (Disability-Adjusted Life Years)	Australia	Male	Age-standardized	Edentulism	Rate	2015	95.9	131.3	60.4
DALYs (Disability-Adjusted Life Years)	Australia	Female	Age-standardized	Edentulism	Rate	2015	177.5	242.5	113.8
DALYs (Disability-Adjusted Life Years)	Australia	Both	Age-standardized	Edentulism	Rate	2015	138.7	189.3	90.2
DALYs (Disability-Adjusted Life Years)	Republic of Turkey	Male	Age-standardized	Edentulism	Rate	2017	201.1	274.0	129.6
DALYs (Disability-Adjusted Life Years)	Republic of Turkey	Female	Age-standardized	Edentulism	Rate	2017	177.8	240.5	113.2
DALYs (Disability-Adjusted Life Years)	Republic of Turkey	Both	Age-standardized	Edentulism	Rate	2017	188.4	255.1	120.7
DALYs (Disability-Adjusted Life Years)	Republic of India	Male	Age-standardized	Edentulism	Rate	2021	79.2	105.5	54.2
DALYs (Disability-Adjusted Life Years)	Republic of India	Female	Age-standardized	Edentulism	Rate	2021	86.2	114.1	58.2
DALYs (Disability-Adjusted Life Years)	Republic of India	Both	Age-standardized	Edentulism	Rate	2021	82.8	110.0	56.4
DALYs (Disability-Adjusted Life Years)	Japan	Male	Age-standardized	Edentulism	Rate	2017	50.8	70.9	34.2
DALYs (Disability-Adjusted Life Years)	Japan	Female	Age-standardized	Edentulism	Rate	2017	42.7	59.3	29.3
DALYs (Disability-Adjusted	Japan	Both	Age-standardized	Edentulism	Rate	2017	46.9	65.3	31.9

Life Years)									
DALYs (Disability-Adjusted Life Years)	Russian Federation	Male	Age-standardized	Edentulism	Rate	2017	158.7	211.5	105.6
DALYs (Disability-Adjusted Life Years)	Russian Federation	Female	Age-standardized	Edentulism	Rate	2017	186.6	248.3	124.6
DALYs (Disability-Adjusted Life Years)	Russian Federation	Both	Age-standardized	Edentulism	Rate	2017	176.2	234.7	118.2
DALYs (Disability-Adjusted Life Years)	Republic of Italy	Male	Age-standardized	Edentulism	Rate	2017	93.5	131.7	59.4
DALYs (Disability-Adjusted Life Years)	Republic of Italy	Female	Age-standardized	Edentulism	Rate	2017	120.1	170.5	75.7
DALYs (Disability-Adjusted Life Years)	Republic of Italy	Both	Age-standardized	Edentulism	Rate	2017	107.6	153.0	68.0
DALYs (Disability-Adjusted Life Years)	Republic of Indonesia	Male	Age-standardized	Edentulism	Rate	2017	84.4	113.7	56.2
DALYs (Disability-Adjusted Life Years)	Republic of Indonesia	Female	Age-standardized	Edentulism	Rate	2017	137.8	185.4	91.2
DALYs (Disability-Adjusted Life Years)	Republic of Indonesia	Both	Age-standardized	Edentulism	Rate	2017	111.9	150.5	74.3
DALYs (Disability-Adjusted Life Years)	Canada	Male	Age-standardized	Edentulism	Rate	2017	71.4	98.3	44.7
DALYs (Disability-Adjusted Life Years)	Canada	Female	Age-standardized	Edentulism	Rate	2017	88.8	121.8	55.9
DALYs (Disability-Adjusted Life Years)	Canada	Both	Age-standardized	Edentulism	Rate	2017	80.4	110.9	50.5
DALYs (Disability-Adjusted Life Years)	French Republic	Male	Age-standardized	Edentulism	Rate	2017	82.0	116.6	51.9
DALYs (Disability-Adjusted Life Years)	French Republic	Female	Age-standardized	Edentulism	Rate	2017	114.1	161.1	72.7
DALYs	French Republic	Both	Age-standardized	Edentulism	Rate	2017	99.3	140.5	63.3

(Disability-Adjusted Life Years)									
DALYs (Disability-Adjusted Life Years)	People's Republic of China	Male	Age-standardized	Edentulism	Rate	2017	66.1	93.0	44.8
DALYs (Disability-Adjusted Life Years)	People's Republic of China	Female	Age-standardized	Edentulism	Rate	2017	81.7	114.5	54.4
DALYs (Disability-Adjusted Life Years)	People's Republic of China	Both	Age-standardized	Edentulism	Rate	2017	74.0	103.9	49.7
DALYs (Disability-Adjusted Life Years)	United Kingdom of Great Britain and Northern Ireland	Male	Age-standardized	Edentulism	Rate	2017	104.9	145.7	69.5
DALYs (Disability-Adjusted Life Years)	United Kingdom of Great Britain and Northern Ireland	Female	Age-standardized	Edentulism	Rate	2017	130.2	180.1	86.4
DALYs (Disability-Adjusted Life Years)	United Kingdom of Great Britain and Northern Ireland	Both	Age-standardized	Edentulism	Rate	2017	118.0	163.8	78.2
DALYs (Disability-Adjusted Life Years)	Argentine Republic	Male	Age-standardized	Edentulism	Rate	2017	85.5	119.3	54.3
DALYs (Disability-Adjusted Life Years)	Argentine Republic	Female	Age-standardized	Edentulism	Rate	2017	136.5	196.4	83.3
DALYs (Disability-Adjusted Life Years)	Argentine Republic	Both	Age-standardized	Edentulism	Rate	2017	113.8	161.1	70.9
DALYs (Disability-Adjusted Life Years)	Australia	Male	Age-standardized	Edentulism	Rate	2017	111.7	154.1	72.7
DALYs (Disability-Adjusted Life Years)	Australia	Female	Age-standardized	Edentulism	Rate	2017	177.2	245.9	115.6
DALYs (Disability-Adjusted Life Years)	Australia	Both	Age-standardized	Edentulism	Rate	2017	146.0	201.6	95.1
DALYs (Disability-Adjusted Life Years)	Republic of Korea	Male	Age-standardized	Edentulism	Rate	2022	50.4	70.2	32.4
DALYs (Disability-Adjusted Life Years)	Republic of Korea	Female	Age-standardized	Edentulism	Rate	2022	47.0	67.2	31.0

DALYs (Disability-Adjusted Life Years)	Republic of Korea	Both	Age-standardized	Edentulism	Rate	2022	49.4	69.7	33.1
DALYs (Disability-Adjusted Life Years)	Federal Republic of Germany	Male	Age-standardized	Edentulism	Rate	2022	92.7	130.1	58.2
DALYs (Disability-Adjusted Life Years)	Federal Republic of Germany	Female	Age-standardized	Edentulism	Rate	2022	119.6	168.1	75.7
DALYs (Disability-Adjusted Life Years)	Federal Republic of Germany	Both	Age-standardized	Edentulism	Rate	2022	106.8	151.8	67.3
DALYs (Disability-Adjusted Life Years)	United Mexican States	Male	Age-standardized	Edentulism	Rate	2018	119.6	159.3	78.4
DALYs (Disability-Adjusted Life Years)	United Mexican States	Female	Age-standardized	Edentulism	Rate	2018	207.6	277.3	137.3
DALYs (Disability-Adjusted Life Years)	United Mexican States	Both	Age-standardized	Edentulism	Rate	2018	166.3	221.8	109.6
DALYs (Disability-Adjusted Life Years)	United States of America	Male	Age-standardized	Edentulism	Rate	2018	131.0	176.1	83.7
DALYs (Disability-Adjusted Life Years)	United States of America	Female	Age-standardized	Edentulism	Rate	2018	143.8	191.4	91.6
DALYs (Disability-Adjusted Life Years)	United States of America	Both	Age-standardized	Edentulism	Rate	2018	137.7	184.1	87.8
DALYs (Disability-Adjusted Life Years)	Republic of India	Male	Age-standardized	Edentulism	Rate	2022	79.4	106.6	52.7
DALYs (Disability-Adjusted Life Years)	Republic of India	Female	Age-standardized	Edentulism	Rate	2022	85.9	113.7	55.8
DALYs (Disability-Adjusted Life Years)	Republic of India	Both	Age-standardized	Edentulism	Rate	2022	82.7	110.4	54.6
DALYs (Disability-Adjusted Life Years)	Kingdom of Saudi Arabia	Male	Age-standardized	Edentulism	Rate	2022	155.3	213.2	100.2
DALYs (Disability-Adjusted	Kingdom of Saudi Arabia	Female	Age-standardized	Edentulism	Rate	2022	107.2	145.9	69.2

Life Years)									
DALYs (Disability-Adjusted Life Years)	Kingdom of Saudi Arabia	Both	Age-standardized	Edentulism	Rate	2022	135.7	185.2	88.0
DALYs (Disability-Adjusted Life Years)	European Union	Male	Age-standardized	Edentulism	Rate	2017	103.2	143.1	67.1
DALYs (Disability-Adjusted Life Years)	European Union	Female	Age-standardized	Edentulism	Rate	2017	131.1	181.5	84.4
DALYs (Disability-Adjusted Life Years)	European Union	Both	Age-standardized	Edentulism	Rate	2017	118.2	163.7	76.3
DALYs (Disability-Adjusted Life Years)	Federative Republic of Brazil	Male	Age-standardized	Edentulism	Rate	2022	202.6	272.5	135.3
DALYs (Disability-Adjusted Life Years)	Federative Republic of Brazil	Female	Age-standardized	Edentulism	Rate	2022	275.9	371.2	183.8
DALYs (Disability-Adjusted Life Years)	Federative Republic of Brazil	Both	Age-standardized	Edentulism	Rate	2022	242.0	322.1	159.6
DALYs (Disability-Adjusted Life Years)	United Kingdom of Great Britain and Northern Ireland	Male	Age-standardized	Edentulism	Rate	2018	104.3	145.4	69.0
DALYs (Disability-Adjusted Life Years)	United Kingdom of Great Britain and Northern Ireland	Female	Age-standardized	Edentulism	Rate	2018	129.9	179.8	86.2
DALYs (Disability-Adjusted Life Years)	United Kingdom of Great Britain and Northern Ireland	Both	Age-standardized	Edentulism	Rate	2018	117.6	163.5	77.9
DALYs (Disability-Adjusted Life Years)	Argentine Republic	Male	Age-standardized	Edentulism	Rate	2018	85.6	120.1	53.8
DALYs (Disability-Adjusted Life Years)	Argentine Republic	Female	Age-standardized	Edentulism	Rate	2018	136.6	197.3	83.7
DALYs (Disability-Adjusted Life Years)	Argentine Republic	Both	Age-standardized	Edentulism	Rate	2018	113.9	163.0	70.8
DALYs (Disability-Adjusted Life Years)	Republic of South Africa	Male	Age-standardized	Edentulism	Rate	2022	171.1	243.9	105.0
DALYs	Republic of South	Female	Age-standardized	Edentulism	Rate	2022	168.1	236.6	104.4

(Disability-Adjusted Life Years)	Africa								
DALYs (Disability-Adjusted Life Years)	Republic of South Africa	Both	Age-standardized	Edentulism	Rate	2022	169.4	240.0	104.7
DALYs (Disability-Adjusted Life Years)	Japan	Male	Age-standardized	Edentulism	Rate	2019	56.3	77.5	37.9
DALYs (Disability-Adjusted Life Years)	Japan	Female	Age-standardized	Edentulism	Rate	2019	44.8	62.0	30.6
DALYs (Disability-Adjusted Life Years)	Japan	Both	Age-standardized	Edentulism	Rate	2019	50.7	70.0	34.4
DALYs (Disability-Adjusted Life Years)	United States of America	Male	Age-standardized	Edentulism	Rate	2019	134.5	181.1	86.4
DALYs (Disability-Adjusted Life Years)	United States of America	Female	Age-standardized	Edentulism	Rate	2019	147.4	196.7	94.7
DALYs (Disability-Adjusted Life Years)	United States of America	Both	Age-standardized	Edentulism	Rate	2019	141.2	189.2	90.7
DALYs (Disability-Adjusted Life Years)	Republic of Korea	Male	Age-standardized	Edentulism	Rate	2023	50.2	70.7	32.4
DALYs (Disability-Adjusted Life Years)	Republic of Korea	Female	Age-standardized	Edentulism	Rate	2023	46.7	67.0	31.0
DALYs (Disability-Adjusted Life Years)	Republic of Korea	Both	Age-standardized	Edentulism	Rate	2023	49.1	69.7	32.7
DALYs (Disability-Adjusted Life Years)	Federal Republic of Germany	Male	Age-standardized	Edentulism	Rate	2023	92.2	131.7	58.1
DALYs (Disability-Adjusted Life Years)	Federal Republic of Germany	Female	Age-standardized	Edentulism	Rate	2023	119.1	168.4	75.8
DALYs (Disability-Adjusted Life Years)	Federal Republic of Germany	Both	Age-standardized	Edentulism	Rate	2023	106.4	151.1	67.5
DALYs (Disability-Adjusted Life Years)	Russian Federation	Male	Age-standardized	Edentulism	Rate	2019	159.4	212.2	107.1

DALYs (Disability-Adjusted Life Years)	Russian Federation	Female	Age-standardized	Edentulism	Rate	2019	188.4	251.8	125.1
DALYs (Disability-Adjusted Life Years)	Russian Federation	Both	Age-standardized	Edentulism	Rate	2019	177.5	236.9	118.9
DALYs (Disability-Adjusted Life Years)	Russian Federation	Male	Age-standardized	Edentulism	Rate	2018	159.2	212.0	106.3
DALYs (Disability-Adjusted Life Years)	Russian Federation	Female	Age-standardized	Edentulism	Rate	2018	187.8	250.3	125.1
DALYs (Disability-Adjusted Life Years)	Russian Federation	Both	Age-standardized	Edentulism	Rate	2018	177.1	236.0	118.7
DALYs (Disability-Adjusted Life Years)	Republic of Turkey	Male	Age-standardized	Edentulism	Rate	2019	199.9	274.3	126.1
DALYs (Disability-Adjusted Life Years)	Republic of Turkey	Female	Age-standardized	Edentulism	Rate	2019	177.0	238.9	112.9
DALYs (Disability-Adjusted Life Years)	Republic of Turkey	Both	Age-standardized	Edentulism	Rate	2019	187.4	253.2	119.1
DALYs (Disability-Adjusted Life Years)	European Union	Male	Age-standardized	Edentulism	Rate	2018	103.0	143.1	66.8
DALYs (Disability-Adjusted Life Years)	European Union	Female	Age-standardized	Edentulism	Rate	2018	131.2	181.9	84.7
DALYs (Disability-Adjusted Life Years)	European Union	Both	Age-standardized	Edentulism	Rate	2018	118.2	163.5	76.4
DALYs (Disability-Adjusted Life Years)	Federative Republic of Brazil	Male	Age-standardized	Edentulism	Rate	2023	201.3	271.2	134.3
DALYs (Disability-Adjusted Life Years)	Federative Republic of Brazil	Female	Age-standardized	Edentulism	Rate	2023	273.5	367.6	182.1
DALYs (Disability-Adjusted Life Years)	Federative Republic of Brazil	Both	Age-standardized	Edentulism	Rate	2023	240.1	319.9	158.2
DALYs (Disability-Adjusted	Republic of Italy	Male	Age-standardized	Edentulism	Rate	2019	93.3	131.6	59.5

Life Years)									
DALYs (Disability-Adjusted Life Years)	Republic of Italy	Female	Age-standardized	Edentulism	Rate	2019	119.9	169.6	76.1
DALYs (Disability-Adjusted Life Years)	Republic of Italy	Both	Age-standardized	Edentulism	Rate	2019	107.3	152.3	68.2
DALYs (Disability-Adjusted Life Years)	Republic of Italy	Male	Age-standardized	Edentulism	Rate	2018	93.4	132.2	59.3
DALYs (Disability-Adjusted Life Years)	Republic of Italy	Female	Age-standardized	Edentulism	Rate	2018	120.0	170.4	75.9
DALYs (Disability-Adjusted Life Years)	Republic of Italy	Both	Age-standardized	Edentulism	Rate	2018	107.5	152.6	68.1
DALYs (Disability-Adjusted Life Years)	Republic of Turkey	Male	Age-standardized	Edentulism	Rate	2018	200.6	273.4	127.3
DALYs (Disability-Adjusted Life Years)	Republic of Turkey	Female	Age-standardized	Edentulism	Rate	2018	177.4	239.6	112.6
DALYs (Disability-Adjusted Life Years)	Republic of Turkey	Both	Age-standardized	Edentulism	Rate	2018	187.9	254.9	119.2
DALYs (Disability-Adjusted Life Years)	Kingdom of Saudi Arabia	Male	Age-standardized	Edentulism	Rate	2023	154.0	212.7	99.2
DALYs (Disability-Adjusted Life Years)	Kingdom of Saudi Arabia	Female	Age-standardized	Edentulism	Rate	2023	106.1	145.7	69.1
DALYs (Disability-Adjusted Life Years)	Kingdom of Saudi Arabia	Both	Age-standardized	Edentulism	Rate	2023	134.6	184.7	87.0
DALYs (Disability-Adjusted Life Years)	Australia	Male	Age-standardized	Edentulism	Rate	2018	122.1	169.4	79.0
DALYs (Disability-Adjusted Life Years)	Australia	Female	Age-standardized	Edentulism	Rate	2018	177.1	244.6	114.9
DALYs (Disability-Adjusted Life Years)	Australia	Both	Age-standardized	Edentulism	Rate	2018	150.8	207.4	98.2
DALYs	Republic of South	Male	Age-standardized	Edentulism	Rate	2023	170.2	243.0	104.5

(Disability-Adjusted Life Years)	Africa								
DALYs (Disability-Adjusted Life Years)	Republic of South Africa	Female	Age-standardized	Edentulism	Rate	2023	167.1	234.5	103.8
DALYs (Disability-Adjusted Life Years)	Republic of South Africa	Both	Age-standardized	Edentulism	Rate	2023	168.5	238.4	104.1
DALYs (Disability-Adjusted Life Years)	French Republic	Male	Age-standardized	Edentulism	Rate	2019	81.3	114.9	51.4
DALYs (Disability-Adjusted Life Years)	French Republic	Female	Age-standardized	Edentulism	Rate	2019	114.0	161.5	71.6
DALYs (Disability-Adjusted Life Years)	French Republic	Both	Age-standardized	Edentulism	Rate	2019	98.9	139.8	62.2
DALYs (Disability-Adjusted Life Years)	Republic of India	Male	Age-standardized	Edentulism	Rate	2023	78.5	105.5	52.0
DALYs (Disability-Adjusted Life Years)	Republic of India	Female	Age-standardized	Edentulism	Rate	2023	84.7	112.5	55.0
DALYs (Disability-Adjusted Life Years)	Republic of India	Both	Age-standardized	Edentulism	Rate	2023	81.7	109.3	53.8
DALYs (Disability-Adjusted Life Years)	People's Republic of China	Male	Age-standardized	Edentulism	Rate	2018	73.0	102.2	49.8
DALYs (Disability-Adjusted Life Years)	People's Republic of China	Female	Age-standardized	Edentulism	Rate	2018	90.0	126.1	60.3
DALYs (Disability-Adjusted Life Years)	People's Republic of China	Both	Age-standardized	Edentulism	Rate	2018	81.7	114.9	55.2
DALYs (Disability-Adjusted Life Years)	Republic of Indonesia	Male	Age-standardized	Edentulism	Rate	2019	83.7	112.0	55.2
DALYs (Disability-Adjusted Life Years)	Republic of Indonesia	Female	Age-standardized	Edentulism	Rate	2019	136.7	183.6	91.0
DALYs (Disability-Adjusted Life Years)	Republic of Indonesia	Both	Age-standardized	Edentulism	Rate	2019	111.0	148.7	73.7

DALYs (Disability-Adjusted Life Years)	People's Republic of China	Male	Age-standardized	Edentulism	Rate	2019	76.5	107.6	52.2
DALYs (Disability-Adjusted Life Years)	People's Republic of China	Female	Age-standardized	Edentulism	Rate	2019	94.4	131.6	63.5
DALYs (Disability-Adjusted Life Years)	People's Republic of China	Both	Age-standardized	Edentulism	Rate	2019	85.6	120.0	58.0
DALYs (Disability-Adjusted Life Years)	United Mexican States	Male	Age-standardized	Edentulism	Rate	2019	121.6	162.4	79.6
DALYs (Disability-Adjusted Life Years)	United Mexican States	Female	Age-standardized	Edentulism	Rate	2019	210.9	281.2	139.4
DALYs (Disability-Adjusted Life Years)	United Mexican States	Both	Age-standardized	Edentulism	Rate	2019	169.0	225.4	111.3
DALYs (Disability-Adjusted Life Years)	Canada	Male	Age-standardized	Edentulism	Rate	2019	72.1	100.0	44.7
DALYs (Disability-Adjusted Life Years)	Canada	Female	Age-standardized	Edentulism	Rate	2019	88.4	121.0	55.9
DALYs (Disability-Adjusted Life Years)	Canada	Both	Age-standardized	Edentulism	Rate	2019	80.6	110.7	50.7
DALYs (Disability-Adjusted Life Years)	French Republic	Male	Age-standardized	Edentulism	Rate	2018	81.5	114.7	51.6
DALYs (Disability-Adjusted Life Years)	French Republic	Female	Age-standardized	Edentulism	Rate	2018	114.0	160.7	71.8
DALYs (Disability-Adjusted Life Years)	French Republic	Both	Age-standardized	Edentulism	Rate	2018	99.0	139.4	62.5
DALYs (Disability-Adjusted Life Years)	Japan	Male	Age-standardized	Edentulism	Rate	2018	53.6	74.4	36.0
DALYs (Disability-Adjusted Life Years)	Japan	Female	Age-standardized	Edentulism	Rate	2018	43.6	60.0	30.0
DALYs (Disability-Adjusted	Japan	Both	Age-standardized	Edentulism	Rate	2018	48.7	67.6	33.1

Life Years)									
DALYs (Disability-Adjusted Life Years)	Canada	Male	Age-standardized	Edentulism	Rate	2018	71.9	98.0	45.7
DALYs (Disability-Adjusted Life Years)	Canada	Female	Age-standardized	Edentulism	Rate	2018	88.5	122.0	55.8
DALYs (Disability-Adjusted Life Years)	Canada	Both	Age-standardized	Edentulism	Rate	2018	80.6	110.9	50.8
DALYs (Disability-Adjusted Life Years)	United Kingdom of Great Britain and Northern Ireland	Male	Age-standardized	Edentulism	Rate	2019	104.1	145.1	68.7
DALYs (Disability-Adjusted Life Years)	United Kingdom of Great Britain and Northern Ireland	Female	Age-standardized	Edentulism	Rate	2019	129.9	180.2	86.7
DALYs (Disability-Adjusted Life Years)	United Kingdom of Great Britain and Northern Ireland	Both	Age-standardized	Edentulism	Rate	2019	117.5	163.5	78.0
DALYs (Disability-Adjusted Life Years)	Republic of Indonesia	Male	Age-standardized	Edentulism	Rate	2018	84.0	112.7	55.6
DALYs (Disability-Adjusted Life Years)	Republic of Indonesia	Female	Age-standardized	Edentulism	Rate	2018	137.1	184.4	90.6
DALYs (Disability-Adjusted Life Years)	Republic of Indonesia	Both	Age-standardized	Edentulism	Rate	2018	111.4	149.0	73.8
DALYs (Disability-Adjusted Life Years)	United States of America	Male	Age-standardized	Edentulism	Rate	2020	135.2	181.6	87.0
DALYs (Disability-Adjusted Life Years)	United States of America	Female	Age-standardized	Edentulism	Rate	2020	147.7	198.1	95.5
DALYs (Disability-Adjusted Life Years)	United States of America	Both	Age-standardized	Edentulism	Rate	2020	141.6	190.3	91.4
DALYs (Disability-Adjusted Life Years)	European Union	Male	Age-standardized	Edentulism	Rate	2019	102.9	142.7	66.9
DALYs (Disability-Adjusted Life Years)	European Union	Female	Age-standardized	Edentulism	Rate	2019	131.5	183.0	85.0
DALYs	European Union	Both	Age-standardized	Edentulism	Rate	2019	118.3	164.1	76.6

(Disability-Adjusted Life Years)									
DALYs (Disability-Adjusted Life Years)	Argentine Republic	Male	Age-standardized	Edentulism	Rate	2020	85.3	120.3	54.1
DALYs (Disability-Adjusted Life Years)	Argentine Republic	Female	Age-standardized	Edentulism	Rate	2020	136.1	195.0	84.3
DALYs (Disability-Adjusted Life Years)	Argentine Republic	Both	Age-standardized	Edentulism	Rate	2020	113.5	160.5	70.7
DALYs (Disability-Adjusted Life Years)	United Mexican States	Male	Age-standardized	Edentulism	Rate	2020	121.5	162.8	78.9
DALYs (Disability-Adjusted Life Years)	United Mexican States	Female	Age-standardized	Edentulism	Rate	2020	210.6	281.1	139.8
DALYs (Disability-Adjusted Life Years)	United Mexican States	Both	Age-standardized	Edentulism	Rate	2020	168.8	225.6	111.5
DALYs (Disability-Adjusted Life Years)	Argentine Republic	Male	Age-standardized	Edentulism	Rate	2019	85.6	119.9	53.8
DALYs (Disability-Adjusted Life Years)	Argentine Republic	Female	Age-standardized	Edentulism	Rate	2019	136.8	197.0	83.9
DALYs (Disability-Adjusted Life Years)	Argentine Republic	Both	Age-standardized	Edentulism	Rate	2019	114.0	162.5	71.2
DALYs (Disability-Adjusted Life Years)	Australia	Male	Age-standardized	Edentulism	Rate	2019	126.9	174.9	81.0
DALYs (Disability-Adjusted Life Years)	Australia	Female	Age-standardized	Edentulism	Rate	2019	176.7	244.2	115.3
DALYs (Disability-Adjusted Life Years)	Australia	Both	Age-standardized	Edentulism	Rate	2019	152.9	210.8	98.6
DALYs (Disability-Adjusted Life Years)	United States of America	Male	Age-standardized	Edentulism	Rate	2021	134.7	181.0	86.6
DALYs (Disability-Adjusted Life Years)	United States of America	Female	Age-standardized	Edentulism	Rate	2021	147.4	197.4	95.3

DALYs (Disability-Adjusted Life Years)	United States of America	Both	Age-standardized	Edentulism	Rate	2021	141.3	189.2	91.2
DALYs (Disability-Adjusted Life Years)	Russian Federation	Male	Age-standardized	Edentulism	Rate	2020	159.3	213.0	106.7
DALYs (Disability-Adjusted Life Years)	Russian Federation	Female	Age-standardized	Edentulism	Rate	2020	190.4	253.8	125.8
DALYs (Disability-Adjusted Life Years)	Russian Federation	Both	Age-standardized	Edentulism	Rate	2020	178.5	238.1	118.2
DALYs (Disability-Adjusted Life Years)	Australia	Male	Age-standardized	Edentulism	Rate	2020	123.7	172.4	77.7
DALYs (Disability-Adjusted Life Years)	Australia	Female	Age-standardized	Edentulism	Rate	2020	171.0	236.4	108.0
DALYs (Disability-Adjusted Life Years)	Australia	Both	Age-standardized	Edentulism	Rate	2020	148.4	205.4	93.6
DALYs (Disability-Adjusted Life Years)	Republic of Italy	Male	Age-standardized	Edentulism	Rate	2020	93.3	131.1	59.3
DALYs (Disability-Adjusted Life Years)	Republic of Italy	Female	Age-standardized	Edentulism	Rate	2020	119.9	170.0	75.8
DALYs (Disability-Adjusted Life Years)	Republic of Italy	Both	Age-standardized	Edentulism	Rate	2020	107.3	152.5	68.0
DALYs (Disability-Adjusted Life Years)	Japan	Male	Age-standardized	Edentulism	Rate	2020	82.3	114.9	53.7
DALYs (Disability-Adjusted Life Years)	Japan	Female	Age-standardized	Edentulism	Rate	2020	60.1	83.6	40.9
DALYs (Disability-Adjusted Life Years)	Japan	Both	Age-standardized	Edentulism	Rate	2020	71.0	98.9	47.6
DALYs (Disability-Adjusted Life Years)	Japan	Male	Age-standardized	Edentulism	Rate	2021	83.1	116.2	54.5
DALYs (Disability-Adjusted	Japan	Female	Age-standardized	Edentulism	Rate	2021	60.8	84.2	41.5

Life Years)									
DALYs (Disability-Adjusted Life Years)	Japan	Both	Age-standardized	Edentulism	Rate	2021	71.8	99.4	48.3
DALYs (Disability-Adjusted Life Years)	Republic of Turkey	Male	Age-standardized	Edentulism	Rate	2021	198.7	273.2	127.7
DALYs (Disability-Adjusted Life Years)	Republic of Turkey	Female	Age-standardized	Edentulism	Rate	2021	177.2	241.5	112.5
DALYs (Disability-Adjusted Life Years)	Republic of Turkey	Both	Age-standardized	Edentulism	Rate	2021	187.0	255.0	119.6
DALYs (Disability-Adjusted Life Years)	French Republic	Male	Age-standardized	Edentulism	Rate	2021	81.8	115.6	53.1
DALYs (Disability-Adjusted Life Years)	French Republic	Female	Age-standardized	Edentulism	Rate	2021	113.8	161.4	73.4
DALYs (Disability-Adjusted Life Years)	French Republic	Both	Age-standardized	Edentulism	Rate	2021	99.0	139.4	64.0
DALYs (Disability-Adjusted Life Years)	European Union	Male	Age-standardized	Edentulism	Rate	2020	105.0	145.4	68.1
DALYs (Disability-Adjusted Life Years)	European Union	Female	Age-standardized	Edentulism	Rate	2020	134.0	186.8	86.8
DALYs (Disability-Adjusted Life Years)	European Union	Both	Age-standardized	Edentulism	Rate	2020	120.5	167.6	78.1
DALYs (Disability-Adjusted Life Years)	Russian Federation	Male	Age-standardized	Edentulism	Rate	2021	160.7	216.0	105.2
DALYs (Disability-Adjusted Life Years)	Russian Federation	Female	Age-standardized	Edentulism	Rate	2021	194.7	258.5	128.3
DALYs (Disability-Adjusted Life Years)	Russian Federation	Both	Age-standardized	Edentulism	Rate	2021	181.5	242.1	118.9
DALYs (Disability-Adjusted Life Years)	Republic of Turkey	Male	Age-standardized	Edentulism	Rate	2020	199.0	273.6	126.8
DALYs	Republic of Turkey	Female	Age-standardized	Edentulism	Rate	2020	176.5	239.8	112.2

(Disability-Adjusted Life Years)									
DALYs (Disability-Adjusted Life Years)	Republic of Turkey	Both	Age-standardized	Edentulism	Rate	2020	186.7	253.1	118.1
DALYs (Disability-Adjusted Life Years)	People's Republic of China	Male	Age-standardized	Edentulism	Rate	2020	76.7	107.6	52.0
DALYs (Disability-Adjusted Life Years)	People's Republic of China	Female	Age-standardized	Edentulism	Rate	2020	95.1	132.1	63.8
DALYs (Disability-Adjusted Life Years)	People's Republic of China	Both	Age-standardized	Edentulism	Rate	2020	86.1	120.3	58.1
DALYs (Disability-Adjusted Life Years)	Republic of Italy	Male	Age-standardized	Edentulism	Rate	2021	93.2	131.0	59.0
DALYs (Disability-Adjusted Life Years)	Republic of Italy	Female	Age-standardized	Edentulism	Rate	2021	119.8	169.1	76.0
DALYs (Disability-Adjusted Life Years)	Republic of Italy	Both	Age-standardized	Edentulism	Rate	2021	107.2	151.8	68.0
DALYs (Disability-Adjusted Life Years)	United Mexican States	Male	Age-standardized	Edentulism	Rate	2021	121.4	161.7	78.7
DALYs (Disability-Adjusted Life Years)	United Mexican States	Female	Age-standardized	Edentulism	Rate	2021	209.4	279.1	139.2
DALYs (Disability-Adjusted Life Years)	United Mexican States	Both	Age-standardized	Edentulism	Rate	2021	168.3	223.8	111.1
DALYs (Disability-Adjusted Life Years)	French Republic	Male	Age-standardized	Edentulism	Rate	2020	81.4	115.1	51.7
DALYs (Disability-Adjusted Life Years)	French Republic	Female	Age-standardized	Edentulism	Rate	2020	113.5	160.4	71.6
DALYs (Disability-Adjusted Life Years)	French Republic	Both	Age-standardized	Edentulism	Rate	2020	98.7	139.4	62.5
DALYs (Disability-Adjusted Life Years)	Canada	Male	Age-standardized	Edentulism	Rate	2021	69.7	95.2	44.7

DALYs (Disability-Adjusted Life Years)	Canada	Female	Age-standardized	Edentulism	Rate	2021	90.0	123.5	57.6
DALYs (Disability-Adjusted Life Years)	Canada	Both	Age-standardized	Edentulism	Rate	2021	80.3	110.7	51.4
DALYs (Disability-Adjusted Life Years)	Canada	Male	Age-standardized	Edentulism	Rate	2020	71.2	99.3	45.0
DALYs (Disability-Adjusted Life Years)	Canada	Female	Age-standardized	Edentulism	Rate	2020	90.1	124.0	58.1
DALYs (Disability-Adjusted Life Years)	Canada	Both	Age-standardized	Edentulism	Rate	2020	81.0	112.1	52.1
DALYs (Disability-Adjusted Life Years)	Republic of Indonesia	Male	Age-standardized	Edentulism	Rate	2020	83.8	113.6	55.6
DALYs (Disability-Adjusted Life Years)	Republic of Indonesia	Female	Age-standardized	Edentulism	Rate	2020	136.3	181.9	90.6
DALYs (Disability-Adjusted Life Years)	Republic of Indonesia	Both	Age-standardized	Edentulism	Rate	2020	110.8	148.7	73.5
DALYs (Disability-Adjusted Life Years)	Republic of Indonesia	Male	Age-standardized	Edentulism	Rate	2021	83.2	112.5	55.3
DALYs (Disability-Adjusted Life Years)	Republic of Indonesia	Female	Age-standardized	Edentulism	Rate	2021	135.7	181.1	90.8
DALYs (Disability-Adjusted Life Years)	Republic of Indonesia	Both	Age-standardized	Edentulism	Rate	2021	110.2	147.6	73.2
DALYs (Disability-Adjusted Life Years)	People's Republic of China	Male	Age-standardized	Edentulism	Rate	2021	76.5	107.3	51.8
DALYs (Disability-Adjusted Life Years)	People's Republic of China	Female	Age-standardized	Edentulism	Rate	2021	94.5	131.0	63.7
DALYs (Disability-Adjusted Life Years)	People's Republic of China	Both	Age-standardized	Edentulism	Rate	2021	85.7	119.8	57.8
DALYs (Disability-Adjusted	United Kingdom of Great Britain and	Male	Age-standardized	Edentulism	Rate	2021	104.9	145.2	69.8

Life Years)	Northern Ireland								
DALYs (Disability-Adjusted Life Years)	United Kingdom of Great Britain and Northern Ireland	Female	Age-standardized	Edentulism	Rate	2021	131.8	181.8	89.0
DALYs (Disability-Adjusted Life Years)	United Kingdom of Great Britain and Northern Ireland	Both	Age-standardized	Edentulism	Rate	2021	118.8	164.2	79.6
DALYs (Disability-Adjusted Life Years)	United States of America	Male	Age-standardized	Edentulism	Rate	2022	134.7	181.2	86.5
DALYs (Disability-Adjusted Life Years)	United States of America	Female	Age-standardized	Edentulism	Rate	2022	147.5	198.2	95.7
DALYs (Disability-Adjusted Life Years)	United States of America	Both	Age-standardized	Edentulism	Rate	2022	141.3	190.0	91.3
DALYs (Disability-Adjusted Life Years)	United Kingdom of Great Britain and Northern Ireland	Male	Age-standardized	Edentulism	Rate	2020	104.6	144.9	69.7
DALYs (Disability-Adjusted Life Years)	United Kingdom of Great Britain and Northern Ireland	Female	Age-standardized	Edentulism	Rate	2020	130.3	180.4	86.9
DALYs (Disability-Adjusted Life Years)	United Kingdom of Great Britain and Northern Ireland	Both	Age-standardized	Edentulism	Rate	2020	117.9	163.0	78.7
DALYs (Disability-Adjusted Life Years)	Argentine Republic	Male	Age-standardized	Edentulism	Rate	2021	85.5	122.1	53.7
DALYs (Disability-Adjusted Life Years)	Argentine Republic	Female	Age-standardized	Edentulism	Rate	2021	136.6	192.4	86.3
DALYs (Disability-Adjusted Life Years)	Argentine Republic	Both	Age-standardized	Edentulism	Rate	2021	113.9	161.0	71.9
DALYs (Disability-Adjusted Life Years)	Argentine Republic	Male	Age-standardized	Edentulism	Rate	2022	85.7	119.9	54.9
DALYs (Disability-Adjusted Life Years)	Argentine Republic	Female	Age-standardized	Edentulism	Rate	2022	137.3	194.4	84.8
DALYs (Disability-Adjusted Life Years)	Argentine Republic	Both	Age-standardized	Edentulism	Rate	2022	114.3	162.9	71.5
DALYs	European Union	Male	Age-standardized	Edentulism	Rate	2021	104.9	146.5	67.9

(Disability-Adjusted Life Years)									
DALYs (Disability-Adjusted Life Years)	European Union	Female	Age-standardized	Edentulism	Rate	2021	133.7	185.7	86.5
DALYs (Disability-Adjusted Life Years)	European Union	Both	Age-standardized	Edentulism	Rate	2021	120.4	166.8	77.9
DALYs (Disability-Adjusted Life Years)	United Mexican States	Male	Age-standardized	Edentulism	Rate	2022	121.3	162.4	78.8
DALYs (Disability-Adjusted Life Years)	United Mexican States	Female	Age-standardized	Edentulism	Rate	2022	210.4	281.5	139.2
DALYs (Disability-Adjusted Life Years)	United Mexican States	Both	Age-standardized	Edentulism	Rate	2022	168.9	225.9	111.1
DALYs (Disability-Adjusted Life Years)	European Union	Male	Age-standardized	Edentulism	Rate	2022	104.8	144.5	67.9
DALYs (Disability-Adjusted Life Years)	European Union	Female	Age-standardized	Edentulism	Rate	2022	133.9	186.3	86.4
DALYs (Disability-Adjusted Life Years)	European Union	Both	Age-standardized	Edentulism	Rate	2022	120.3	166.8	77.8
DALYs (Disability-Adjusted Life Years)	Japan	Male	Age-standardized	Edentulism	Rate	2022	83.5	116.1	55.1
DALYs (Disability-Adjusted Life Years)	Japan	Female	Age-standardized	Edentulism	Rate	2022	61.6	85.4	41.8
DALYs (Disability-Adjusted Life Years)	Japan	Both	Age-standardized	Edentulism	Rate	2022	72.4	99.8	49.0
DALYs (Disability-Adjusted Life Years)	French Republic	Male	Age-standardized	Edentulism	Rate	2023	81.1	115.3	51.1
DALYs (Disability-Adjusted Life Years)	French Republic	Female	Age-standardized	Edentulism	Rate	2023	113.5	160.8	71.6
DALYs (Disability-Adjusted Life Years)	French Republic	Both	Age-standardized	Edentulism	Rate	2023	98.5	139.6	62.0

DALYs (Disability-Adjusted Life Years)	Russian Federation	Male	Age-standardized	Edentulism	Rate	2023	161.2	215.5	105.7
DALYs (Disability-Adjusted Life Years)	Russian Federation	Female	Age-standardized	Edentulism	Rate	2023	194.7	258.0	127.4
DALYs (Disability-Adjusted Life Years)	Russian Federation	Both	Age-standardized	Edentulism	Rate	2023	181.6	241.3	118.1
DALYs (Disability-Adjusted Life Years)	Australia	Male	Age-standardized	Edentulism	Rate	2021	124.3	172.2	80.7
DALYs (Disability-Adjusted Life Years)	Australia	Female	Age-standardized	Edentulism	Rate	2021	172.1	239.0	112.0
DALYs (Disability-Adjusted Life Years)	Australia	Both	Age-standardized	Edentulism	Rate	2021	149.2	206.0	96.8
DALYs (Disability-Adjusted Life Years)	Republic of Italy	Male	Age-standardized	Edentulism	Rate	2022	93.5	131.4	59.3
DALYs (Disability-Adjusted Life Years)	Republic of Italy	Female	Age-standardized	Edentulism	Rate	2022	119.9	169.7	76.1
DALYs (Disability-Adjusted Life Years)	Republic of Italy	Both	Age-standardized	Edentulism	Rate	2022	107.4	152.1	68.1
DALYs (Disability-Adjusted Life Years)	Republic of Turkey	Male	Age-standardized	Edentulism	Rate	2023	198.6	273.6	127.1
DALYs (Disability-Adjusted Life Years)	Republic of Turkey	Female	Age-standardized	Edentulism	Rate	2023	175.3	240.8	112.9
DALYs (Disability-Adjusted Life Years)	Republic of Turkey	Both	Age-standardized	Edentulism	Rate	2023	185.9	254.7	120.3
DALYs (Disability-Adjusted Life Years)	Australia	Male	Age-standardized	Edentulism	Rate	2022	123.8	171.8	79.8
DALYs (Disability-Adjusted Life Years)	Australia	Female	Age-standardized	Edentulism	Rate	2022	171.5	236.4	108.4
DALYs (Disability-Adjusted	Australia	Both	Age-standardized	Edentulism	Rate	2022	148.6	204.2	94.2

Life Years)									
DALYs (Disability-Adjusted Life Years)	United Mexican States	Male	Age-standardized	Edentulism	Rate	2023	120.4	160.8	78.0
DALYs (Disability-Adjusted Life Years)	United Mexican States	Female	Age-standardized	Edentulism	Rate	2023	208.5	279.6	138.0
DALYs (Disability-Adjusted Life Years)	United Mexican States	Both	Age-standardized	Edentulism	Rate	2023	167.5	224.2	110.1
DALYs (Disability-Adjusted Life Years)	Republic of Indonesia	Male	Age-standardized	Edentulism	Rate	2023	82.3	111.1	54.3
DALYs (Disability-Adjusted Life Years)	Republic of Indonesia	Female	Age-standardized	Edentulism	Rate	2023	134.1	180.2	89.4
DALYs (Disability-Adjusted Life Years)	Republic of Indonesia	Both	Age-standardized	Edentulism	Rate	2023	108.9	146.0	72.4
DALYs (Disability-Adjusted Life Years)	Republic of Italy	Male	Age-standardized	Edentulism	Rate	2023	93.0	130.9	59.2
DALYs (Disability-Adjusted Life Years)	Republic of Italy	Female	Age-standardized	Edentulism	Rate	2023	119.1	168.0	75.8
DALYs (Disability-Adjusted Life Years)	Republic of Italy	Both	Age-standardized	Edentulism	Rate	2023	106.7	150.9	67.9
DALYs (Disability-Adjusted Life Years)	People's Republic of China	Male	Age-standardized	Edentulism	Rate	2022	76.6	107.5	52.0
DALYs (Disability-Adjusted Life Years)	People's Republic of China	Female	Age-standardized	Edentulism	Rate	2022	94.5	130.8	63.7
DALYs (Disability-Adjusted Life Years)	People's Republic of China	Both	Age-standardized	Edentulism	Rate	2022	85.8	120.0	58.0
DALYs (Disability-Adjusted Life Years)	Russian Federation	Male	Age-standardized	Edentulism	Rate	2022	162.0	216.5	105.2
DALYs (Disability-Adjusted Life Years)	Russian Federation	Female	Age-standardized	Edentulism	Rate	2022	195.4	259.5	128.0
DALYs	Russian Federation	Both	Age-standardized	Edentulism	Rate	2022	182.4	242.7	118.7

(Disability-Adjusted Life Years)									
DALYs (Disability-Adjusted Life Years)	Republic of Turkey	Male	Age-standardized	Edentulism	Rate	2022	201.1	277.6	129.2
DALYs (Disability-Adjusted Life Years)	Republic of Turkey	Female	Age-standardized	Edentulism	Rate	2022	178.0	244.6	113.1
DALYs (Disability-Adjusted Life Years)	Republic of Turkey	Both	Age-standardized	Edentulism	Rate	2022	188.5	258.8	120.4
DALYs (Disability-Adjusted Life Years)	United Kingdom of Great Britain and Northern Ireland	Male	Age-standardized	Edentulism	Rate	2022	106.7	145.6	72.9
DALYs (Disability-Adjusted Life Years)	United Kingdom of Great Britain and Northern Ireland	Female	Age-standardized	Edentulism	Rate	2022	133.3	182.2	91.3
DALYs (Disability-Adjusted Life Years)	United Kingdom of Great Britain and Northern Ireland	Both	Age-standardized	Edentulism	Rate	2022	120.5	164.7	82.7
DALYs (Disability-Adjusted Life Years)	Canada	Male	Age-standardized	Edentulism	Rate	2022	70.8	98.8	44.9
DALYs (Disability-Adjusted Life Years)	Canada	Female	Age-standardized	Edentulism	Rate	2022	89.2	121.8	55.5
DALYs (Disability-Adjusted Life Years)	Canada	Both	Age-standardized	Edentulism	Rate	2022	80.4	110.7	50.8
DALYs (Disability-Adjusted Life Years)	United Kingdom of Great Britain and Northern Ireland	Male	Age-standardized	Edentulism	Rate	2023	106.2	145.7	72.6
DALYs (Disability-Adjusted Life Years)	United Kingdom of Great Britain and Northern Ireland	Female	Age-standardized	Edentulism	Rate	2023	132.7	181.8	90.7
DALYs (Disability-Adjusted Life Years)	United Kingdom of Great Britain and Northern Ireland	Both	Age-standardized	Edentulism	Rate	2023	119.9	164.0	82.1
DALYs (Disability-Adjusted Life Years)	Canada	Male	Age-standardized	Edentulism	Rate	2023	70.5	96.9	44.2
DALYs (Disability-Adjusted Life Years)	Canada	Female	Age-standardized	Edentulism	Rate	2023	89.0	121.9	54.7

DALYs (Disability-Adjusted Life Years)	Canada	Both	Age-standardized	Edentulism	Rate	2023	80.1	109.7	49.9
DALYs (Disability-Adjusted Life Years)	People's Republic of China	Male	Age-standardized	Edentulism	Rate	2023	75.7	106.2	51.3
DALYs (Disability-Adjusted Life Years)	People's Republic of China	Female	Age-standardized	Edentulism	Rate	2023	93.1	129.4	62.9
DALYs (Disability-Adjusted Life Years)	People's Republic of China	Both	Age-standardized	Edentulism	Rate	2023	84.6	118.3	57.2
DALYs (Disability-Adjusted Life Years)	Argentine Republic	Male	Age-standardized	Edentulism	Rate	2023	85.1	119.7	54.8
DALYs (Disability-Adjusted Life Years)	Argentine Republic	Female	Age-standardized	Edentulism	Rate	2023	136.2	194.6	85.5
DALYs (Disability-Adjusted Life Years)	Argentine Republic	Both	Age-standardized	Edentulism	Rate	2023	113.5	162.0	71.8
DALYs (Disability-Adjusted Life Years)	French Republic	Male	Age-standardized	Edentulism	Rate	2022	81.6	116.5	51.1
DALYs (Disability-Adjusted Life Years)	French Republic	Female	Age-standardized	Edentulism	Rate	2022	114.1	160.2	73.1
DALYs (Disability-Adjusted Life Years)	French Republic	Both	Age-standardized	Edentulism	Rate	2022	99.1	139.5	62.8
DALYs (Disability-Adjusted Life Years)	United States of America	Male	Age-standardized	Edentulism	Rate	2023	134.0	180.2	86.2
DALYs (Disability-Adjusted Life Years)	United States of America	Female	Age-standardized	Edentulism	Rate	2023	146.5	197.0	95.1
DALYs (Disability-Adjusted Life Years)	United States of America	Both	Age-standardized	Edentulism	Rate	2023	140.5	189.0	90.8
DALYs (Disability-Adjusted Life Years)	Republic of Indonesia	Male	Age-standardized	Edentulism	Rate	2022	83.1	112.5	54.9
DALYs (Disability-Adjusted	Republic of Indonesia	Female	Age-standardized	Edentulism	Rate	2022	135.5	181.6	90.0

Life Years)									
DALYs (Disability-Adjusted Life Years)	Republic of Indonesia	Both	Age-standardized	Edentulism	Rate	2022	110.1	147.6	73.1
DALYs (Disability-Adjusted Life Years)	Australia	Male	Age-standardized	Edentulism	Rate	2023	123.2	168.7	78.8
DALYs (Disability-Adjusted Life Years)	Australia	Female	Age-standardized	Edentulism	Rate	2023	170.5	235.1	107.8
DALYs (Disability-Adjusted Life Years)	Australia	Both	Age-standardized	Edentulism	Rate	2023	147.8	202.4	93.9
DALYs (Disability-Adjusted Life Years)	Japan	Male	Age-standardized	Edentulism	Rate	2023	83.1	115.4	54.9
DALYs (Disability-Adjusted Life Years)	Japan	Female	Age-standardized	Edentulism	Rate	2023	61.3	84.4	41.6
DALYs (Disability-Adjusted Life Years)	Japan	Both	Age-standardized	Edentulism	Rate	2023	72.1	99.3	48.8
DALYs (Disability-Adjusted Life Years)	European Union	Male	Age-standardized	Edentulism	Rate	2023	104.2	144.6	67.4
DALYs (Disability-Adjusted Life Years)	European Union	Female	Age-standardized	Edentulism	Rate	2023	133.1	184.8	86.1
DALYs (Disability-Adjusted Life Years)	European Union	Both	Age-standardized	Edentulism	Rate	2023	119.7	166.0	77.3

Table S2. Age-group distributions of edentulism incidence and disability-adjusted life years in China and G20 countries, 1990–2023.

measure_name	location_name	sex_name	age_name	cause_name	metric_name	year	val	upper	lower	measure_name	location_name	sex_name	age_name	cause_name	metric_name	year	val	upper	lower
Incidence	People's Republic of China	Male	5-9 years	Edentulism	Number	2023	0.0	0.0	0.0	Incidence	People's Republic of China	Male	5-9 years	Edentulism	Rate	2023	0.0	0.0	0.0
Incidence	People's Republic of China	Female	5-9 years	Edentulism	Number	2023	0.0	0.0	0.0	Incidence	People's Republic of China	Female	5-9 years	Edentulism	Rate	2023	0.0	0.0	0.0
Incidence	People's Republic of China	Male	10-14 years	Edentulism	Number	2023	0.0	0.0	0.0	Incidence	People's Republic of China	Male	10-14 years	Edentulism	Rate	2023	0.0	0.0	0.0
Incidence	People's Republic of China	Female	10-14 years	Edentulism	Number	2023	0.0	0.0	0.0	Incidence	People's Republic of China	Female	10-14 years	Edentulism	Rate	2023	0.0	0.0	0.0
Incidence	People's Republic of China	Male	15-19 years	Edentulism	Number	2023	901.6	2243.4	198.6	Incidence	People's Republic of China	Male	15-19 years	Edentulism	Rate	2023	2.1	5.2	0.5
Incidence	People's Republic of China	Female	15-19 years	Edentulism	Number	2023	1011.7	2181.8	293.1	Incidence	People's Republic of China	Female	15-19 years	Edentulism	Rate	2023	2.7	5.8	0.8
Incidence	People's Republic of China	Male	20-24 years	Edentulism	Number	2023	2376.4	4460.9	984.2	Incidence	People's Republic of China	Male	20-24 years	Edentulism	Rate	2023	6.3	11.8	2.6
Incidence	People's Republic of China	Female	20-24 years	Edentulism	Number	2023	2862.7	4772.9	1456.8	Incidence	People's Republic of China	Female	20-24 years	Edentulism	Rate	2023	8.7	14.5	4.4
Incidence	People's Republic of China	Male	25-29 years	Edentulism	Number	2023	4403.1	7596.3	2138.6	Incidence	People's Republic of China	Male	25-29 years	Edentulism	Rate	2023	10.6	18.3	5.1
Incidence	People's Republic of China	Female	25-29 years	Edentulism	Number	2023	5644.9	9509.2	3005.5	Incidence	People's Republic of China	Female	25-29 years	Edentulism	Rate	2023	15.5	26.1	8.3
Incidence	People's Republic of China	Male	30-34 years	Edentulism	Number	2023	9791.6	16935.0	4747.8	Incidence	People's Republic of China	Male	30-34 years	Edentulism	Rate	2023	17.4	30.0	8.4
Incidence	People's Republic of China	Female	30-34 years	Edentulism	Number	2023	13048.4	20849.2	7497.1	Incidence	People's Republic of China	Female	30-34 years	Edentulism	Rate	2023	25.6	40.9	14.7
Incidence	People's Republic of China	Male	35-39 years	Edentulism	Number	2023	18773.3	30984.5	10632.9	Incidence	People's Republic of China	Male	35-39 years	Edentulism	Rate	2023	30.9	51.0	17.5

Incidence	People's Republic of China	Female	35-39 years	Edentulism	Number	2023	26227.3	40659.7	15666.4	Incidence	People's Republic of China	Female	35-39 years	Edentulism	Rate	2023	45.7	70.8	27.3
Incidence	People's Republic of China	Male	40-44 years	Edentulism	Number	2023	30019.5	47706.8	17476.9	Incidence	People's Republic of China	Male	40-44 years	Edentulism	Rate	2023	64.3	102.1	37.4
Incidence	People's Republic of China	Female	40-44 years	Edentulism	Number	2023	42684.9	66932.1	25320.7	Incidence	People's Republic of China	Female	40-44 years	Edentulism	Rate	2023	96.5	151.3	57.2
Incidence	People's Republic of China	Male	45-49 years	Edentulism	Number	2023	71638.1	114268.2	40423.6	Incidence	People's Republic of China	Male	45-49 years	Edentulism	Rate	2023	141.3	225.4	79.7
Incidence	People's Republic of China	Female	45-49 years	Edentulism	Number	2023	105276.0	166788.3	61326.1	Incidence	People's Republic of China	Female	45-49 years	Edentulism	Rate	2023	214.6	340.0	125.0
Incidence	People's Republic of China	Male	50-54 years	Edentulism	Number	2023	181076.6	296810.7	116127.8	Incidence	People's Republic of China	Male	50-54 years	Edentulism	Rate	2023	301.3	493.9	193.2
Incidence	People's Republic of China	Female	50-54 years	Edentulism	Number	2023	259347.7	412970.8	170657.1	Incidence	People's Republic of China	Female	50-54 years	Edentulism	Rate	2023	444.9	708.4	292.7
Incidence	People's Republic of China	Male	55-59 years	Edentulism	Number	2023	348848.0	537418.9	217071.6	Incidence	People's Republic of China	Male	55-59 years	Edentulism	Rate	2023	592.4	912.6	368.6
Incidence	People's Republic of China	Female	55-59 years	Edentulism	Number	2023	483375.8	735127.6	306166.2	Incidence	People's Republic of China	Female	55-59 years	Edentulism	Rate	2023	814.7	1239.0	516.0
Incidence	People's Republic of China	Male	60-64 years	Edentulism	Number	2023	423110.4	638636.9	281819.2	Incidence	People's Republic of China	Male	60-64 years	Edentulism	Rate	2023	1044.0	1575.7	695.3
Incidence	People's Republic of China	Female	60-64 years	Edentulism	Number	2023	539574.6	786821.2	363912.4	Incidence	People's Republic of China	Female	60-64 years	Edentulism	Rate	2023	1319.3	1923.8	889.8
Incidence	People's Republic of China	Male	65-69 years	Edentulism	Number	2023	595043.4	889422.6	361756.0	Incidence	People's Republic of China	Male	65-69 years	Edentulism	Rate	2023	1578.4	2359.3	959.6
Incidence	People's Republic of China	Female	65-69 years	Edentulism	Number	2023	710368.0	1037669.0	442301.1	Incidence	People's Republic of China	Female	65-69 years	Edentulism	Rate	2023	1839.7	2687.3	1145.5
Incidence	People's Republic of China	Male	70-74 years	Edentulism	Number	2023	587165.6	844103.3	374945.3	Incidence	People's Republic of China	Male	70-74 years	Edentulism	Rate	2023	2002.0	2878.1	1278.4
Incidence	People's Republic of	Female	70-74 years	Edentulism	Number	2023	682842	976830.7	448455.9	Incidence	People's Republic of	Female	70-74 years	Edentulism	Rate	2023	2152.3	3078.9	1413.5

	China						.7				China								
Incidence	People's Republic of China	Male	75-79 years	Edentulism	Number	2023	391956.6	551937.2	242885.1	Incidence	People's Republic of China	Male	75-79 years	Edentulism	Rate	2023	2274.8	3203.3	1409.6
Incidence	People's Republic of China	Female	75-79 years	Edentulism	Number	2023	441211.5	616858.8	281243.4	Incidence	People's Republic of China	Female	75-79 years	Edentulism	Rate	2023	2256.0	3154.1	1438.0
Incidence	People's Republic of China	Male	<1 year	Edentulism	Number	2023	0.0	0.0	0.0	Incidence	People's Republic of China	Male	<1 year	Edentulism	Rate	2023	0.0	0.0	0.0
Incidence	People's Republic of China	Female	<1 year	Edentulism	Number	2023	0.0	0.0	0.0	Incidence	People's Republic of China	Female	<1 year	Edentulism	Rate	2023	0.0	0.0	0.0
Incidence	People's Republic of China	Male	80-84 years	Edentulism	Number	2023	222904.6	321030.7	141830.6	Incidence	People's Republic of China	Male	80-84 years	Edentulism	Rate	2023	2215.2	3190.4	1409.5
Incidence	People's Republic of China	Female	80-84 years	Edentulism	Number	2023	255098.4	370729.4	161990.0	Incidence	People's Republic of China	Female	80-84 years	Edentulism	Rate	2023	2056.9	2989.2	1306.2
Incidence	People's Republic of China	Male	85-89 years	Edentulism	Number	2023	94771.4	137259.1	62344.4	Incidence	People's Republic of China	Male	85-89 years	Edentulism	Rate	2023	1856.0	2688.1	1221.0
Incidence	People's Republic of China	Female	85-89 years	Edentulism	Number	2023	120603.3	178527.0	77932.1	Incidence	People's Republic of China	Female	85-89 years	Edentulism	Rate	2023	1637.3	2423.7	1058.0
Incidence	People's Republic of China	Male	90-94 years	Edentulism	Number	2023	26326.7	37965.5	16212.6	Incidence	People's Republic of China	Male	90-94 years	Edentulism	Rate	2023	1546.4	2230.1	952.3
Incidence	People's Republic of China	Female	90-94 years	Edentulism	Number	2023	37703.6	56294.4	22489.9	Incidence	People's Republic of China	Female	90-94 years	Edentulism	Rate	2023	1334.1	1992.0	795.8
Incidence	People's Republic of China	Male	2-4 years	Edentulism	Number	2023	0.0	0.0	0.0	Incidence	People's Republic of China	Male	2-4 years	Edentulism	Rate	2023	0.0	0.0	0.0
Incidence	People's Republic of China	Female	2-4 years	Edentulism	Number	2023	0.0	0.0	0.0	Incidence	People's Republic of China	Female	2-4 years	Edentulism	Rate	2023	0.0	0.0	0.0
DALYs (Disability-Adjusted Life Years)	People's Republic of China	Male	5-9 years	Edentulism	Number	2023	0.0	0.0	0.0	DALYs (Disability-Adjusted Life Years)	People's Republic of China	Male	5-9 years	Edentulism	Rate	2023	0.0	0.0	0.0
DALYs (Disabili	People's Republic of	Female	5-9 years	Edentulism	Number	2023	0.0	0.0	0.0	DALYs (Disabili	People's Republic of	Female	5-9 years	Edentulism	Rate	2023	0.0	0.0	0.0

ty-Adjusted Life Years)	China									ty-Adjusted Life Years)	China								
DALYs (Disability-Adjusted Life Years)	People's Republic of China	Male	10-14 years	Edentulism	Number	2023	0.0	0.0	0.0	DALYs (Disability-Adjusted Life Years)	People's Republic of China	Male	10-14 years	Edentulism	Rate	2023	0.0	0.0	0.0
DALYs (Disability-Adjusted Life Years)	People's Republic of China	Female	10-14 years	Edentulism	Number	2023	0.0	0.0	0.0	DALYs (Disability-Adjusted Life Years)	People's Republic of China	Female	10-14 years	Edentulism	Rate	2023	0.0	0.0	0.0
DALYs (Disability-Adjusted Life Years)	People's Republic of China	Male	15-19 years	Edentulism	Number	2023	46.1	122.1	9.2	DALYs (Disability-Adjusted Life Years)	People's Republic of China	Male	15-19 years	Edentulism	Rate	2023	0.1	0.3	0.0
DALYs (Disability-Adjusted Life Years)	People's Republic of China	Female	15-19 years	Edentulism	Number	2023	51.6	119.5	11.6	DALYs (Disability-Adjusted Life Years)	People's Republic of China	Female	15-19 years	Edentulism	Rate	2023	0.1	0.3	0.0
DALYs (Disability-Adjusted Life Years)	People's Republic of China	Male	20-24 years	Edentulism	Number	2023	275.8	575.8	79.1	DALYs (Disability-Adjusted Life Years)	People's Republic of China	Male	20-24 years	Edentulism	Rate	2023	0.7	1.5	0.2
DALYs (Disability-Adjusted Life Years)	People's Republic of China	Female	20-24 years	Edentulism	Number	2023	310.9	596.4	111.0	DALYs (Disability-Adjusted Life Years)	People's Republic of China	Female	20-24 years	Edentulism	Rate	2023	0.9	1.8	0.3
DALYs (Disability-Adjusted Life Years)	People's Republic of China	Male	25-29 years	Edentulism	Number	2023	810.4	1648.7	348.7	DALYs (Disability-Adjusted Life Years)	People's Republic of China	Male	25-29 years	Edentulism	Rate	2023	1.9	4.0	0.8
DALYs (Disability-Adjusted Life Years)	People's Republic of China	Female	25-29 years	Edentulism	Number	2023	1000.5	1779.2	492.6	DALYs (Disability-Adjusted Life Years)	People's Republic of China	Female	25-29 years	Edentulism	Rate	2023	2.7	4.9	1.4
DALYs	People's	Male	30-34	Edentuli	Numbe	2023	222	4020.	1019	DALYs	People's	Male	30-34 years	Edentuli	Rate	2023	3.9	7.1	1.8

(Disability-Adjusted Life Years)	Republic of China		years	sm	r		3.1	8	.6	(Disability-Adjusted Life Years)	Republic of China			sm					
DALYs (Disability-Adjusted Life Years)	People's Republic of China	Female	30-34 years	Edentulism	Number	2023	2879.6	5157.2	1538.4	DALYs (Disability-Adjusted Life Years)	People's Republic of China	Female	30-34 years	Edentulism	Rate	2023	5.6	10.1	3.0
DALYs (Disability-Adjusted Life Years)	People's Republic of China	Male	35-39 years	Edentulism	Number	2023	4455.3	7933.3	2201.7	DALYs (Disability-Adjusted Life Years)	People's Republic of China	Male	35-39 years	Edentulism	Rate	2023	7.3	13.0	3.6
DALYs (Disability-Adjusted Life Years)	People's Republic of China	Female	35-39 years	Edentulism	Number	2023	6139.6	9524.5	3314.8	DALYs (Disability-Adjusted Life Years)	People's Republic of China	Female	35-39 years	Edentulism	Rate	2023	10.7	16.6	5.8
DALYs (Disability-Adjusted Life Years)	People's Republic of China	Male	40-44 years	Edentulism	Number	2023	6374.9	9567.7	3521.2	DALYs (Disability-Adjusted Life Years)	People's Republic of China	Male	40-44 years	Edentulism	Rate	2023	13.6	20.5	7.5
DALYs (Disability-Adjusted Life Years)	People's Republic of China	Female	40-44 years	Edentulism	Number	2023	8900.8	13108.0	5318.5	DALYs (Disability-Adjusted Life Years)	People's Republic of China	Female	40-44 years	Edentulism	Rate	2023	20.1	29.6	12.0
DALYs (Disability-Adjusted Life Years)	People's Republic of China	Male	45-49 years	Edentulism	Number	2023	13715.3	20226.9	7943.7	DALYs (Disability-Adjusted Life Years)	People's Republic of China	Male	45-49 years	Edentulism	Rate	2023	27.1	39.9	15.7
DALYs (Disability-Adjusted Life Years)	People's Republic of China	Female	45-49 years	Edentulism	Number	2023	19589.3	28813.6	11725.6	DALYs (Disability-Adjusted Life Years)	People's Republic of China	Female	45-49 years	Edentulism	Rate	2023	39.9	58.7	23.9
DALYs (Disability-Adjusted Life Years)	People's Republic of China	Male	50-54 years	Edentulism	Number	2023	33403.8	49476.8	18973.8	DALYs (Disability-Adjusted Life Years)	People's Republic of China	Male	50-54 years	Edentulism	Rate	2023	55.6	82.3	31.6

DALYs (Disability-Adjusted Life Years)	People's Republic of China	Female	50-54 years	Edentulism	Number	2023	48101.9	71982.4	27510.0	DALYs (Disability-Adjusted Life Years)	People's Republic of China	Female	50-54 years	Edentulism	Rate	2023	82.5	123.5	47.2
DALYs (Disability-Adjusted Life Years)	People's Republic of China	Male	55-59 years	Edentulism	Number	2023	65243.4	93154.0	37955.3	DALYs (Disability-Adjusted Life Years)	People's Republic of China	Male	55-59 years	Edentulism	Rate	2023	110.8	158.2	64.5
DALYs (Disability-Adjusted Life Years)	People's Republic of China	Female	55-59 years	Edentulism	Number	2023	95149.9	134887.1	56499.5	DALYs (Disability-Adjusted Life Years)	People's Republic of China	Female	55-59 years	Edentulism	Rate	2023	160.4	227.3	95.2
DALYs (Disability-Adjusted Life Years)	People's Republic of China	Male	60-64 years	Edentulism	Number	2023	84328.2	120850.9	49345.4	DALYs (Disability-Adjusted Life Years)	People's Republic of China	Male	60-64 years	Edentulism	Rate	2023	208.1	298.2	121.8
DALYs (Disability-Adjusted Life Years)	People's Republic of China	Female	60-64 years	Edentulism	Number	2023	117413.6	165918.5	69956.6	DALYs (Disability-Adjusted Life Years)	People's Republic of China	Female	60-64 years	Edentulism	Rate	2023	287.1	405.7	171.0
DALYs (Disability-Adjusted Life Years)	People's Republic of China	Male	65-69 years	Edentulism	Number	2023	136503.2	198836.3	81743.0	DALYs (Disability-Adjusted Life Years)	People's Republic of China	Male	65-69 years	Edentulism	Rate	2023	362.1	527.4	216.8
DALYs (Disability-Adjusted Life Years)	People's Republic of China	Female	65-69 years	Edentulism	Number	2023	181936.9	261846.5	109076.7	DALYs (Disability-Adjusted Life Years)	People's Republic of China	Female	65-69 years	Edentulism	Rate	2023	471.2	678.1	282.5
DALYs (Disability-Adjusted Life Years)	People's Republic of China	Male	70-74 years	Edentulism	Number	2023	164107.6	243914.1	104319.1	DALYs (Disability-Adjusted Life Years)	People's Republic of China	Male	70-74 years	Edentulism	Rate	2023	559.6	831.7	355.7
DALYs (Disability-Adjusted Life	People's Republic of China	Female	70-74 years	Edentulism	Number	2023	217768.0	319192.0	141051.8	DALYs (Disability-Adjusted Life	People's Republic of China	Female	70-74 years	Edentulism	Rate	2023	686.4	1006.1	444.6

Years)										Years)									
DALYs (Disability-Adjusted Life Years)	People's Republic of China	Male	75-79 years	Edentulism	Number	2023	132484.6	193240.3	84530.7	DALYs (Disability-Adjusted Life Years)	People's Republic of China	Male	75-79 years	Edentulism	Rate	2023	768.9	1121.5	490.6
DALYs (Disability-Adjusted Life Years)	People's Republic of China	Female	75-79 years	Edentulism	Number	2023	174067.3	248929.4	113418.8	DALYs (Disability-Adjusted Life Years)	People's Republic of China	Female	75-79 years	Edentulism	Rate	2023	890.0	1272.8	579.9
DALYs (Disability-Adjusted Life Years)	People's Republic of China	Male	<1 year	Edentulism	Number	2023	0.0	0.0	0.0	DALYs (Disability-Adjusted Life Years)	People's Republic of China	Male	<1 year	Edentulism	Rate	2023	0.0	0.0	0.0
DALYs (Disability-Adjusted Life Years)	People's Republic of China	Female	<1 year	Edentulism	Number	2023	0.0	0.0	0.0	DALYs (Disability-Adjusted Life Years)	People's Republic of China	Female	<1 year	Edentulism	Rate	2023	0.0	0.0	0.0
DALYs (Disability-Adjusted Life Years)	People's Republic of China	Male	80-84 years	Edentulism	Number	2023	97307.6	136460.4	63370.7	DALYs (Disability-Adjusted Life Years)	People's Republic of China	Male	80-84 years	Edentulism	Rate	2023	967.0	1356.1	629.8
DALYs (Disability-Adjusted Life Years)	People's Republic of China	Female	80-84 years	Edentulism	Number	2023	132005.1	184094.7	87228.2	DALYs (Disability-Adjusted Life Years)	People's Republic of China	Female	80-84 years	Edentulism	Rate	2023	1064.4	1484.4	703.3
DALYs (Disability-Adjusted Life Years)	People's Republic of China	Male	85-89 years	Edentulism	Number	2023	57055.0	78835.8	36932.1	DALYs (Disability-Adjusted Life Years)	People's Republic of China	Male	85-89 years	Edentulism	Rate	2023	1117.4	1543.9	723.3
DALYs (Disability-Adjusted Life Years)	People's Republic of China	Female	85-89 years	Edentulism	Number	2023	86643.0	118655.7	56158.3	DALYs (Disability-Adjusted Life Years)	People's Republic of China	Female	85-89 years	Edentulism	Rate	2023	1176.3	1610.9	762.4
DALYs (Disability-Adjus	People's Republic of China	Male	90-94 years	Edentulism	Number	2023	20803.8	28862.0	13232.1	DALYs (Disability-Adjus	People's Republic of China	Male	90-94 years	Edentulism	Rate	2023	1222.0	1695.4	777.3

ted Life Years)										ted Life Years)									
DALYs (Disability-Adjusted Life Years)	People's Republic of China	Female	90-94 years	Edentulism	Number	2023	34952.0	47622.0	22368.1	DALYs (Disability-Adjusted Life Years)	People's Republic of China	Female	90-94 years	Edentulism	Rate	2023	1236.8	1685.1	791.5
DALYs (Disability-Adjusted Life Years)	People's Republic of China	Male	2-4 years	Edentulism	Number	2023	0.0	0.0	0.0	DALYs (Disability-Adjusted Life Years)	People's Republic of China	Male	2-4 years	Edentulism	Rate	2023	0.0	0.0	0.0
DALYs (Disability-Adjusted Life Years)	People's Republic of China	Female	2-4 years	Edentulism	Number	2023	0.0	0.0	0.0	DALYs (Disability-Adjusted Life Years)	People's Republic of China	Female	2-4 years	Edentulism	Rate	2023	0.0	0.0	0.0
DALYs (Disability-Adjusted Life Years)	People's Republic of China	Male	95+ years	Edentulism	Number	2023	5443.7	7699.9	3505.9	DALYs (Disability-Adjusted Life Years)	People's Republic of China	Male	95+ years	Edentulism	Rate	2023	1284.8	1817.3	827.4
DALYs (Disability-Adjusted Life Years)	People's Republic of China	Female	95+ years	Edentulism	Number	2023	10299.0	14429.8	6623.6	DALYs (Disability-Adjusted Life Years)	People's Republic of China	Female	95+ years	Edentulism	Rate	2023	1259.2	1764.3	809.8
Incidence	G20	Male	5-9 years	Edentulism	Number	2023	0.0	0.0	0.0	Incidence	G20	Male	5-9 years	Edentulism	Rate	2023	0.0	0.0	0.0
Incidence	G20	Female	5-9 years	Edentulism	Number	2023	0.0	0.0	0.0	Incidence	G20	Female	5-9 years	Edentulism	Rate	2023	0.0	0.0	0.0
Incidence	G20	Male	10-14 years	Edentulism	Number	2023	0.0	0.0	0.0	Incidence	G20	Male	10-14 years	Edentulism	Rate	2023	0.0	0.0	0.0
Incidence	G20	Female	10-14 years	Edentulism	Number	2023	0.0	0.0	0.0	Incidence	G20	Female	10-14 years	Edentulism	Rate	2023	0.0	0.0	0.0
Incidence	G20	Male	15-19 years	Edentulism	Number	2023	14379.4	24211.4	6462.3	Incidence	G20	Male	15-19 years	Edentulism	Rate	2023	8.2	13.8	3.7
Incidence	G20	Female	15-19 years	Edentulism	Number	2023	17938.5	29155.9	8396.7	Incidence	G20	Female	15-19 years	Edentulism	Rate	2023	11.1	18.0	5.2
Incidence	G20	Male	20-24 years	Edentulism	Number	2023	50748.5	70490.6	31626.7	Incidence	G20	Male	20-24 years	Edentulism	Rate	2023	29.8	41.3	18.5

Incidence	G20	Female	20-24 years	Edentulism	Number	2023	63268.1	86869.5	40241.1	Incidence	G20	Female	20-24 years	Edentulism	Rate	2023	39.9	54.8	25.4
Incidence	G20	Male	25-29 years	Edentulism	Number	2023	87905.6	131296.6	58623.7	Incidence	G20	Male	25-29 years	Edentulism	Rate	2023	50.9	76.0	33.9
Incidence	G20	Female	25-29 years	Edentulism	Number	2023	109459.9	161477.1	73102.7	Incidence	G20	Female	25-29 years	Edentulism	Rate	2023	67.5	99.6	45.1
Incidence	G20	Male	30-34 years	Edentulism	Number	2023	130822.7	192982.6	84228.5	Incidence	G20	Male	30-34 years	Edentulism	Rate	2023	70.5	103.9	45.4
Incidence	G20	Female	30-34 years	Edentulism	Number	2023	162807.9	236575.3	103557.5	Incidence	G20	Female	30-34 years	Edentulism	Rate	2023	92.9	135.0	59.1
Incidence	G20	Male	35-39 years	Edentulism	Number	2023	190859.2	283794.2	120424.2	Incidence	G20	Male	35-39 years	Edentulism	Rate	2023	102.5	152.4	64.7
Incidence	G20	Female	35-39 years	Edentulism	Number	2023	242668.2	367273.7	152312.5	Incidence	G20	Female	35-39 years	Edentulism	Rate	2023	135.9	205.7	85.3
Incidence	G20	Male	40-44 years	Edentulism	Number	2023	294081.1	442967.1	185343.3	Incidence	G20	Male	40-44 years	Edentulism	Rate	2023	180.5	271.9	113.8
Incidence	G20	Female	40-44 years	Edentulism	Number	2023	375520.1	572715.9	230695.4	Incidence	G20	Female	40-44 years	Edentulism	Rate	2023	238.2	363.2	146.3
Incidence	G20	Male	45-49 years	Edentulism	Number	2023	470589.2	679521.6	317840.7	Incidence	G20	Male	45-49 years	Edentulism	Rate	2023	306.4	442.5	207.0
Incidence	G20	Female	45-49 years	Edentulism	Number	2023	601072.6	843198.2	408678.0	Incidence	G20	Female	45-49 years	Edentulism	Rate	2023	398.3	558.7	270.8
Incidence	G20	Male	50-54 years	Edentulism	Number	2023	743698.7	1106354.1	510704.3	Incidence	G20	Male	50-54 years	Edentulism	Rate	2023	482.3	717.5	331.2
Incidence	G20	Female	50-54 years	Edentulism	Number	2023	964971.7	1427108.0	662829.3	Incidence	G20	Female	50-54 years	Edentulism	Rate	2023	630.1	931.9	432.8
Incidence	G20	Male	55-59 years	Edentulism	Number	2023	1051816.5	1516847.4	722171.8	Incidence	G20	Male	55-59 years	Edentulism	Rate	2023	739.8	1066.9	507.9
Incidence	G20	Female	55-59 years	Edentulism	Number	2023	138958	1956608.6	964625.9	Incidence	G20	Female	55-59 years	Edentulism	Rate	2023	951.8	1340.2	660.7

							0.1												
Incidence	G20	Male	60-64 years	Edentulism	Number	2023	1224433.5	1675243.4	849239.9	Incidence	G20	Male	60-64 years	Edentulism	Rate	2023	1068.4	1461.7	741.0
Incidence	G20	Female	60-64 years	Edentulism	Number	2023	1571173.2	2112985.4	1087481.2	Incidence	G20	Female	60-64 years	Edentulism	Rate	2023	1305.5	1755.8	903.6
Incidence	G20	Male	65-69 years	Edentulism	Number	2023	1363817.0	1905314.4	866629.8	Incidence	G20	Male	65-69 years	Edentulism	Rate	2023	1391.4	1943.9	884.2
Incidence	G20	Female	65-69 years	Edentulism	Number	2023	1697046.5	2347874.6	1088127.4	Incidence	G20	Female	65-69 years	Edentulism	Rate	2023	1606.6	2222.8	1030.2
Incidence	G20	Male	70-74 years	Edentulism	Number	2023	1252135.4	1759344.1	797384.8	Incidence	G20	Male	70-74 years	Edentulism	Rate	2023	1654.4	2324.6	1053.6
Incidence	G20	Female	70-74 years	Edentulism	Number	2023	1535677.1	2110627.0	989157.1	Incidence	G20	Female	70-74 years	Edentulism	Rate	2023	1792.3	2463.4	1154.5
Incidence	G20	Male	75-79 years	Edentulism	Number	2023	873130.4	1225732.0	576786.7	Incidence	G20	Male	75-79 years	Edentulism	Rate	2023	1811.5	2543.0	1196.6
Incidence	G20	Female	75-79 years	Edentulism	Number	2023	1046704.6	1413558.2	702428.1	Incidence	G20	Female	75-79 years	Edentulism	Rate	2023	1819.9	2457.7	1221.3
Incidence	G20	Male	<1 year	Edentulism	Number	2023	0.0	0.0	0.0	Incidence	G20	Male	<1 year	Edentulism	Rate	2023	0.0	0.0	0.0
Incidence	G20	Female	<1 year	Edentulism	Number	2023	0.0	0.0	0.0	Incidence	G20	Female	<1 year	Edentulism	Rate	2023	0.0	0.0	0.0
Incidence	G20	Male	80-84 years	Edentulism	Number	2023	498807.2	701468.2	332702.5	Incidence	G20	Male	80-84 years	Edentulism	Rate	2023	1726.6	2428.0	1151.6
Incidence	G20	Female	80-84 years	Edentulism	Number	2023	621536.9	860882.9	418709.2	Incidence	G20	Female	80-84 years	Edentulism	Rate	2023	1606.6	2225.3	1082.3
Incidence	G20	Male	85-89 years	Edentulism	Number	2023	203675.3	289532.4	142888.0	Incidence	G20	Male	85-89 years	Edentulism	Rate	2023	1403.1	1994.5	984.3
Incidence	G20	Female	85-89 years	Edentulism	Number	2023	280394.6	399004.8	197771.6	Incidence	G20	Female	85-89 years	Edentulism	Rate	2023	1217.0	1731.8	858.4
Incidence	G20	Male	90-94 years	Edentulism	Number	2023	58369.2	84647.5	38120.3	Incidence	G20	Male	90-94 years	Edentulism	Rate	2023	1161.5	1684.4	758.6

Incidence	G20	Female	90-94 years	Edentulism	Number	2023	92444.9	134517.2	59230.2	Incidence	G20	Female	90-94 years	Edentulism	Rate	2023	938.5	1365.7	601.3
Incidence	G20	Male	2-4 years	Edentulism	Number	2023	0.0	0.0	0.0	Incidence	G20	Male	2-4 years	Edentulism	Rate	2023	0.0	0.0	0.0
Incidence	G20	Female	2-4 years	Edentulism	Number	2023	0.0	0.0	0.0	Incidence	G20	Female	2-4 years	Edentulism	Rate	2023	0.0	0.0	0.0
DALYs (Disability-Adjusted Life Years)	G20	Male	5-9 years	Edentulism	Number	2023	0.0	0.0	0.0	DALYs (Disability-Adjusted Life Years)	G20	Male	5-9 years	Edentulism	Rate	2023	0.0	0.0	0.0
DALYs (Disability-Adjusted Life Years)	G20	Female	5-9 years	Edentulism	Number	2023	0.0	0.0	0.0	DALYs (Disability-Adjusted Life Years)	G20	Female	5-9 years	Edentulism	Rate	2023	0.0	0.0	0.0
DALYs (Disability-Adjusted Life Years)	G20	Male	10-14 years	Edentulism	Number	2023	0.0	0.0	0.0	DALYs (Disability-Adjusted Life Years)	G20	Male	10-14 years	Edentulism	Rate	2023	0.0	0.0	0.0
DALYs (Disability-Adjusted Life Years)	G20	Female	10-14 years	Edentulism	Number	2023	0.0	0.0	0.0	DALYs (Disability-Adjusted Life Years)	G20	Female	10-14 years	Edentulism	Rate	2023	0.0	0.0	0.0
DALYs (Disability-Adjusted Life Years)	G20	Male	15-19 years	Edentulism	Number	2023	742.5	1390.2	287.9	DALYs (Disability-Adjusted Life Years)	G20	Male	15-19 years	Edentulism	Rate	2023	0.4	0.8	0.2
DALYs (Disability-Adjusted Life Years)	G20	Female	15-19 years	Edentulism	Number	2023	899.3	1619.6	358.2	DALYs (Disability-Adjusted Life Years)	G20	Female	15-19 years	Edentulism	Rate	2023	0.6	1.0	0.2
DALYs (Disability-Adjusted Life Years)	G20	Male	20-24 years	Edentulism	Number	2023	5388.3	9207.7	2601.8	DALYs (Disability-Adjusted Life Years)	G20	Male	20-24 years	Edentulism	Rate	2023	3.2	5.4	1.5
DALYs (Disabili	G20	Female	20-24 years	Edentulism	Number	2023	6547.3	10443.4	3292.4	DALYs (Disabili	G20	Female	20-24 years	Edentulism	Rate	2023	4.1	6.6	2.1

ty-Adjusted Life Years)										ty-Adjusted Life Years)									
DALYs (Disability-Adjusted Life Years)	G20	Male	25-29 years	Edentulism	Number	2023	15817.6	25537.6	8413.0	DALYs (Disability-Adjusted Life Years)	G20	Male	25-29 years	Edentulism	Rate	2023	9.2	14.8	4.9
DALYs (Disability-Adjusted Life Years)	G20	Female	25-29 years	Edentulism	Number	2023	19329.5	31008.7	11076.2	DALYs (Disability-Adjusted Life Years)	G20	Female	25-29 years	Edentulism	Rate	2023	11.9	19.1	6.8
DALYs (Disability-Adjusted Life Years)	G20	Male	30-34 years	Edentulism	Number	2023	32191.7	50434.0	18356.8	DALYs (Disability-Adjusted Life Years)	G20	Male	30-34 years	Edentulism	Rate	2023	17.3	27.2	9.9
DALYs (Disability-Adjusted Life Years)	G20	Female	30-34 years	Edentulism	Number	2023	39219.4	60684.8	23151.8	DALYs (Disability-Adjusted Life Years)	G20	Female	30-34 years	Edentulism	Rate	2023	22.4	34.6	13.2
DALYs (Disability-Adjusted Life Years)	G20	Male	35-39 years	Edentulism	Number	2023	54881.1	81979.2	33146.1	DALYs (Disability-Adjusted Life Years)	G20	Male	35-39 years	Edentulism	Rate	2023	29.5	44.0	17.8
DALYs (Disability-Adjusted Life Years)	G20	Female	35-39 years	Edentulism	Number	2023	67547.7	100204.2	41599.4	DALYs (Disability-Adjusted Life Years)	G20	Female	35-39 years	Edentulism	Rate	2023	37.8	56.1	23.3
DALYs (Disability-Adjusted Life Years)	G20	Male	40-44 years	Edentulism	Number	2023	81612.9	116685.7	50410.3	DALYs (Disability-Adjusted Life Years)	G20	Male	40-44 years	Edentulism	Rate	2023	50.1	71.6	30.9
DALYs (Disability-Adjusted Life Years)	G20	Female	40-44 years	Edentulism	Number	2023	102514.9	145235.5	62846.7	DALYs (Disability-Adjusted Life Years)	G20	Female	40-44 years	Edentulism	Rate	2023	65.0	92.1	39.9
DALYs	G20	Male	45-49	Edentuli	Numbe	2023	121	17103	7386	DALYs	G20	Male	45-49 years	Edentuli	Rate	2023	79.1	111.4	48.1

(Disability-Adjusted Life Years)			years	sm	r		417.1	6.8	7.2	(Disability-Adjusted Life Years)				sm					
DALYs (Disability-Adjusted Life Years)	G20	Female	45-49 years	Edentulism	Number	2023	155456.4	217322.6	97311.6	DALYs (Disability-Adjusted Life Years)	G20	Female	45-49 years	Edentulism	Rate	2023	103.0	144.0	64.5
DALYs (Disability-Adjusted Life Years)	G20	Male	50-54 years	Edentulism	Number	2023	191972.4	272995.2	118186.8	DALYs (Disability-Adjusted Life Years)	G20	Male	50-54 years	Edentulism	Rate	2023	124.5	177.0	76.6
DALYs (Disability-Adjusted Life Years)	G20	Female	50-54 years	Edentulism	Number	2023	248731.8	349212.1	155344.9	DALYs (Disability-Adjusted Life Years)	G20	Female	50-54 years	Edentulism	Rate	2023	162.4	228.0	101.4
DALYs (Disability-Adjusted Life Years)	G20	Male	55-59 years	Edentulism	Number	2023	278936.4	392195.6	174912.3	DALYs (Disability-Adjusted Life Years)	G20	Male	55-59 years	Edentulism	Rate	2023	196.2	275.8	123.0
DALYs (Disability-Adjusted Life Years)	G20	Female	55-59 years	Edentulism	Number	2023	373408.7	520697.6	234669.4	DALYs (Disability-Adjusted Life Years)	G20	Female	55-59 years	Edentulism	Rate	2023	255.8	356.6	160.7
DALYs (Disability-Adjusted Life Years)	G20	Male	60-64 years	Edentulism	Number	2023	355534.7	489357.0	226663.2	DALYs (Disability-Adjusted Life Years)	G20	Male	60-64 years	Edentulism	Rate	2023	310.2	427.0	197.8
DALYs (Disability-Adjusted Life Years)	G20	Female	60-64 years	Edentulism	Number	2023	478174.4	649867.0	307191.5	DALYs (Disability-Adjusted Life Years)	G20	Female	60-64 years	Edentulism	Rate	2023	397.3	540.0	255.3
DALYs (Disability-Adjusted Life Years)	G20	Male	65-69 years	Edentulism	Number	2023	431789.7	601818.8	276808.5	DALYs (Disability-Adjusted Life Years)	G20	Male	65-69 years	Edentulism	Rate	2023	440.5	614.0	282.4

DALYs (Disability-Adjusted Life Years)	G20	Female	65-69 years	Edentulism	Number	2023	583941.6	798346.4	383548.8	DALYs (Disability-Adjusted Life Years)	G20	Female	65-69 years	Edentulism	Rate	2023	552.8	755.8	363.1
DALYs (Disability-Adjusted Life Years)	G20	Male	70-74 years	Edentulism	Number	2023	449615.3	642808.9	298936.0	DALYs (Disability-Adjusted Life Years)	G20	Male	70-74 years	Edentulism	Rate	2023	594.1	849.3	395.0
DALYs (Disability-Adjusted Life Years)	G20	Female	70-74 years	Edentulism	Number	2023	616990.9	859042.4	413035.5	DALYs (Disability-Adjusted Life Years)	G20	Female	70-74 years	Edentulism	Rate	2023	720.1	1002.6	482.1
DALYs (Disability-Adjusted Life Years)	G20	Male	75-79 years	Edentulism	Number	2023	359476.1	503853.5	240218.2	DALYs (Disability-Adjusted Life Years)	G20	Male	75-79 years	Edentulism	Rate	2023	745.8	1045.3	498.4
DALYs (Disability-Adjusted Life Years)	G20	Female	75-79 years	Edentulism	Number	2023	500292.5	689580.0	339027.6	DALYs (Disability-Adjusted Life Years)	G20	Female	75-79 years	Edentulism	Rate	2023	869.8	1199.0	589.5
DALYs (Disability-Adjusted Life Years)	G20	Male	<1 year	Edentulism	Number	2023	0.0	0.0	0.0	DALYs (Disability-Adjusted Life Years)	G20	Male	<1 year	Edentulism	Rate	2023	0.0	0.0	0.0
DALYs (Disability-Adjusted Life Years)	G20	Female	<1 year	Edentulism	Number	2023	0.0	0.0	0.0	DALYs (Disability-Adjusted Life Years)	G20	Female	<1 year	Edentulism	Rate	2023	0.0	0.0	0.0
DALYs (Disability-Adjusted Life Years)	G20	Male	80-84 years	Edentulism	Number	2023	255364.7	361348.4	170347.0	DALYs (Disability-Adjusted Life Years)	G20	Male	80-84 years	Edentulism	Rate	2023	883.9	1250.8	589.6
DALYs (Disability-Adjusted Life	G20	Female	80-84 years	Edentulism	Number	2023	385354.9	534312.2	261280.0	DALYs (Disability-Adjusted Life	G20	Female	80-84 years	Edentulism	Rate	2023	996.1	1381.1	675.4

Years)										Years)									
DALYs (Disability-Adjusted Life Years)	G20	Male	85-89 years	Edentulism	Number	2023	142037.4	195947.0	92784.7	DALYs (Disability-Adjusted Life Years)	G20	Male	85-89 years	Edentulism	Rate	2023	978.4	1349.8	639.2
DALYs (Disability-Adjusted Life Years)	G20	Female	85-89 years	Edentulism	Number	2023	244653.7	333075.2	162501.3	DALYs (Disability-Adjusted Life Years)	G20	Female	85-89 years	Edentulism	Rate	2023	1061.9	1445.7	705.3
DALYs (Disability-Adjusted Life Years)	G20	Male	90-94 years	Edentulism	Number	2023	51495.2	69817.7	33628.6	DALYs (Disability-Adjusted Life Years)	G20	Male	90-94 years	Edentulism	Rate	2023	1024.7	1389.3	669.2
DALYs (Disability-Adjusted Life Years)	G20	Female	90-94 years	Edentulism	Number	2023	104485.7	140321.6	70006.2	DALYs (Disability-Adjusted Life Years)	G20	Female	90-94 years	Edentulism	Rate	2023	1060.8	1424.6	710.7
DALYs (Disability-Adjusted Life Years)	G20	Male	2-4 years	Edentulism	Number	2023	0.0	0.0	0.0	DALYs (Disability-Adjusted Life Years)	G20	Male	2-4 years	Edentulism	Rate	2023	0.0	0.0	0.0
DALYs (Disability-Adjusted Life Years)	G20	Female	2-4 years	Edentulism	Number	2023	0.0	0.0	0.0	DALYs (Disability-Adjusted Life Years)	G20	Female	2-4 years	Edentulism	Rate	2023	0.0	0.0	0.0
DALYs (Disability-Adjusted Life Years)	G20	Male	95+ years	Edentulism	Number	2023	13764.1	19009.0	9100.3	DALYs (Disability-Adjusted Life Years)	G20	Male	95+ years	Edentulism	Rate	2023	1070.7	1478.7	707.9
DALYs (Disability-Adjusted Life Years)	G20	Female	95+ years	Edentulism	Number	2023	34666.4	46375.2	23036.0	DALYs (Disability-Adjusted Life Years)	G20	Female	95+ years	Edentulism	Rate	2023	1041.6	1393.4	692.2

Table S3. Decomposition analysis of changes in incidence and disability-adjusted life years from edentulism in China and G20 countries.

Item	sex_name	Overll difference	Aging	Population	Epidemiological change	a_percent	p_percent	r_percent	val_1990	val_2023	diffl
China(Incidence)	Both	5204820.4	4358853.0	1154532.6	-308565.3	83.8	22.2	-5.9	2623333.0	6751250.0	4127916.0
	Male	2389508.3	2027345.1	509509.4	-147346.1	84.8	21.3	-6.2	1158938.0	3014828.0	1855890.0
	Female	2816193.1	2349860.0	650382.2	-184049.0	83.4	23.1	-6.5	1464396.0	3736422.0	2272026.0
China(DALY)	Both	1724696.0	1531000.8	360795.8	-167100.5	88.8	20.9	-9.7	710208.0	1961790.5	1251582.5
	Male	750903.1	670303.7	149042.2	-68442.8	89.3	19.9	-9.1	291634.3	824581.8	532947.5
	Female	973881.9	855384.7	213039.9	-94542.6	87.8	21.9	-9.7	418573.7	1137208.7	718635.0
G20(Incidence)	Both	14492985.4	2591642.0	11398963.5	502379.9	17.9	78.7	3.5	9403421.0	19322443.0	9919022.0
	Male	8795967.0	3206021.0	5652815.2	-62869.2	36.5	64.3	-0.7	4108869.0	8522993.0	4414124.0
	Female	6398167.3	153389.8	5927157.2	317620.2	2.4	92.6	5.0	5294552.0	10799450.0	5504898.0
G20(DALY)	Both	10789088.5	5158118.8	6396594.7	-765624.9	47.8	59.3	-7.1	3171700.0	6804252.0	3632552.0
	Male	5653878.4	3082187.8	2978502.1	-406811.5	54.5	52.7	-7.2	1295253.0	2842037.0	1546784.0
	Female	5165715.7	2115544.9	3403674.7	-353503.9	41.0	65.9	-6.8	1876446.0	3962215.0	2085769.0

Table S4. Consolidated Autoregressive Integrated Moving Average(ARIMA) model forecasts of edentulism incidence and disability-adjusted life years, by sex and region (China and G20 countries).

Items	Year	Value	Lower	Upper	Type
China male(Incidence)	1990	280.6			Actual
	1991	270.8			Actual
	1992	261.9			Actual
	1993	254.3			Actual
	1994	248.6			Actual
	1995	245.2			Actual
	1996	244.1			Actual
	1997	244.3			Actual
	1998	245.4			Actual
	1999	246.8			Actual
	2000	247.8			Actual
	2001	249.4			Actual
	2002	252.4			Actual
	2003	256.0			Actual
	2004	259.1			Actual
	2005	260.9			Actual
	2006	262.2			Actual
	2007	264.1			Actual
	2008	266.0			Actual
	2009	267.3			Actual
	2010	267.2			Actual
	2011	260.7			Actual
	2012	246.5			Actual
	2013	229.9			Actual
	2014	216.1			Actual
	2015	210.3			Actual
	2016	218.9			Actual
	2017	238.1			Actual
	2018	257.7			Actual
	2019	267.6			Actual
	2020	268.3			Actual
	2021	267.4			Actual
	2022	268.4			Actual
	2023	264.8			Actual
	2024	254.5	208.2	267.4	Forecast
	2025	243.9	213.3	274.1	Forecast
	2026	237.1	220.6	281.4	Forecast
	2027	235.2	226.8	288.8	Forecast
	2028	237.8	230.1	295.0	Forecast
	2029	243.7	230.3	298.5	Forecast
	2030	251.0	228.2	298.6	Forecast

	2031	257.8	224.6	295.7	Forecast
	2032	262.5	220.2	291.3	Forecast
	2033	264.4	215.8	287.1	Forecast
	2034	263.4	212.1	284.3	Forecast
	2035	260.1	250.3	258.7	Forecast
	2036	255.8	231.6	256.2	Forecast
	2037	251.4	216.5	257.7	Forecast
	2038	248.2	208.6	261.7	Forecast
	1990	329.9			Actual
	1991	325.3			Actual
	1992	319.3			Actual
	1993	312.4			Actual
	1994	305.0			Actual
	1995	297.4			Actual
	1996	286.9			Actual
	1997	273.1			Actual
	1998	259.3			Actual
	1999	249.1			Actual
	2000	245.5			Actual
	2001	250.3			Actual
	2002	260.6			Actual
	2003	273.1			Actual
	2004	284.4			Actual
	2005	291.0			Actual
	2006	294.4			Actual
China Female	2007	298.0			Actual
(Incidence)	2008	301.1			Actual
	2009	303.2			Actual
	2010	303.7			Actual
	2011	297.1			Actual
	2012	282.4			Actual
	2013	265.0			Actual
	2014	250.3			Actual
	2015	243.9			Actual
	2016	253.8			Actual
	2017	276.3			Actual
	2018	299.5			Actual
	2019	311.3			Actual
	2020	313.1			Actual
	2021	311.4			Actual
	2022	311.9			Actual
	2023	307.1			Actual
	2024	293.4	213.8	286.4	Forecast
	2025	274.4	220.4	294.1	Forecast

	2026	257.7	229.9	303.7	Forecast
	2027	249.2	238.9	314.3	Forecast
	2028	250.1	247.0	325.3	Forecast
	2029	257.2	254.6	336.7	Forecast
	2030	266.8	261.1	347.3	Forecast
	2031	276.6	265.2	355.0	Forecast
	2032	286.2	265.3	357.5	Forecast
	2033	295.7	261.3	354.0	Forecast
	2034	304.2	253.5	346.3	Forecast
	2035	310.1	288.5	298.3	Forecast
	2036	311.4	260.1	288.8	Forecast
	2037	307.6	232.7	282.7	Forecast
	2038	299.9	216.4	282.0	Forecast
	1990	303.6			Actual
	1991	296.4			Actual
	1992	288.9			Actual
	1993	281.7			Actual
	1994	275.1			Actual
	1995	269.7			Actual
	1996	263.9			Actual
	1997	257.2			Actual
	1998	251.1			Actual
	1999	246.8			Actual
	2000	245.6			Actual
	2001	248.8			Actual
	2002	255.6			Actual
	2003	263.8			Actual
China Both (Incidence)	2004	271.0			Actual
	2005	275.2			Actual
	2006	277.6			Actual
	2007	280.4			Actual
	2008	283.0			Actual
	2009	284.8			Actual
	2010	285.0			Actual
	2011	278.5			Actual
	2012	264.0			Actual
	2013	247.0			Actual
	2014	232.8			Actual
	2015	226.7			Actual
	2016	235.9			Actual
	2017	256.8			Actual
	2018	278.3			Actual
	2019	289.1			Actual
	2020	290.4			Actual

	2021	289.0			Actual
	2022	289.8			Actual
	2023	285.6			Actual
	2024	272.9	210.5	267.7	Forecast
	2025	255.3	219.8	277.0	Forecast
	2026	240.9	229.7	287.9	Forecast
	2027	235.3	237.4	298.4	Forecast
	2028	239.1	243.7	308.0	Forecast
	2029	248.4	249.2	316.7	Forecast
	2030	258.8	253.4	323.6	Forecast
	2031	267.9	254.7	326.6	Forecast
	2032	275.9	251.6	324.2	Forecast
	2033	283.0	244.3	316.9	Forecast
	2034	288.5	234.0	307.4	Forecast
	2035	290.7	268.6	277.1	Forecast
	2036	287.9	243.1	267.6	Forecast
	2037	280.6	219.9	261.8	Forecast
	2038	270.7	208.6	261.9	Forecast
China Male (DALY)	1990	82.3			Actual
	1991	78.5			Actual
	1992	75.0			Actual
	1993	72.0			Actual
	1994	69.7			Actual
	1995	68.5			Actual
	1996	67.9			Actual
	1997	67.4			Actual
	1998	67.2			Actual
	1999	67.1			Actual
	2000	67.3			Actual
	2001	68.0			Actual
	2002	69.3			Actual
	2003	70.8			Actual
	2004	72.1			Actual
	2005	72.9			Actual
	2006	73.5			Actual
	2007	74.1			Actual
	2008	74.7			Actual
	2009	75.2			Actual
	2010	75.3			Actual
	2011	73.3			Actual
	2012	68.6			Actual
	2013	63.0			Actual
	2014	58.4			Actual
	2015	56.4			Actual

	2016	59.4			Actual
	2017	66.1			Actual
	2018	73.0			Actual
	2019	76.5			Actual
	2020	76.7			Actual
	2021	76.5			Actual
	2022	76.6			Actual
	2023	75.7			Actual
	2024	72.9	54.0	75.9	Forecast
	2025	69.3	55.1	77.5	Forecast
	2026	66.2	57.0	79.4	Forecast
	2027	64.7	58.7	81.5	Forecast
	2028	65.0	60.1	83.8	Forecast
	2029	66.3	61.0	85.9	Forecast
	2030	68.2	61.4	87.4	Forecast
	2031	70.1	61.3	88.0	Forecast
	2032	71.9	60.7	87.6	Forecast
	2033	73.4	59.6	86.6	Forecast
	2034	74.4	58.2	85.2	Forecast
	2035	74.6	71.7	74.2	Forecast
	2036	74.1	65.3	73.2	Forecast
	2037	73.1	58.9	73.4	Forecast
	2038	71.7	55.0	74.5	Forecast
China Female (DALY)	1990	102.2			Actual
	1991	100.1			Actual
	1992	97.5			Actual
	1993	94.6			Actual
	1994	91.6			Actual
	1995	88.5			Actual
	1996	84.4			Actual
	1997	79.3			Actual
	1998	74.2			Actual
	1999	70.3			Actual
	2000	68.8			Actual
	2001	70.3			Actual
	2002	73.9			Actual
	2003	78.4			Actual
	2004	82.7			Actual
	2005	85.4			Actual
	2006	87.0			Actual
	2007	88.5			Actual
	2008	89.8			Actual
	2009	90.7			Actual
	2010	91.0			Actual

	2011	88.8			Actual
	2012	83.5			Actual
	2013	77.2			Actual
	2014	72.0			Actual
	2015	69.9			Actual
	2016	73.5			Actual
	2017	81.7			Actual
	2018	90.0			Actual
	2019	94.4			Actual
	2020	95.1			Actual
	2021	94.5			Actual
	2022	94.5			Actual
	2023	93.1			Actual
	2024	89.3	58.1	86.1	Forecast
	2025	83.5	58.6	87.6	Forecast
	2026	77.6	61.0	90.0	Forecast
	2027	73.5	63.8	93.1	Forecast
	2028	72.1	66.5	96.6	Forecast
	2029	73.1	69.2	100.4	Forecast
	2030	75.5	71.7	104.4	Forecast
	2031	78.4	73.8	108.0	Forecast
	2032	81.6	75.1	110.5	Forecast
	2033	84.8	75.3	111.5	Forecast
	2034	88.0	74.2	110.6	Forecast
	2035	90.9	87.6	90.9	Forecast
	2036	92.8	78.5	88.5	Forecast
	2037	93.4	68.6	86.6	Forecast
	2038	92.4	61.2	85.7	Forecast
China Both (DALY)	1990	92.7			Actual
	1991	89.8			Actual
	1992	86.8			Actual
	1993	83.8			Actual
	1994	81.1			Actual
	1995	78.9			Actual
	1996	76.4			Actual
	1997	73.4			Actual
	1998	70.6			Actual
	1999	68.5			Actual
	2000	67.7			Actual
	2001	68.9			Actual
	2002	71.4			Actual
	2003	74.5			Actual
	2004	77.4			Actual
	2005	79.2			Actual

	2006	80.3			Actual
	2007	81.4			Actual
	2008	82.3			Actual
	2009	83.0			Actual
	2010	83.2			Actual
	2011	81.1			Actual
	2012	76.1			Actual
	2013	70.2			Actual
	2014	65.3			Actual
	2015	63.2			Actual
	2016	66.6			Actual
	2017	74.0			Actual
	2018	81.7			Actual
	2019	85.6			Actual
	2020	86.1			Actual
	2021	85.7			Actual
	2022	85.8			Actual
	2023	84.6			Actual
	2024	82.0	60.4	87.7	Forecast
	2025	78.9	59.7	89.0	Forecast
	2026	76.4	60.4	90.4	Forecast
	2027	74.7	61.8	91.8	Forecast
	2028	74.1	63.2	93.3	Forecast
	2029	74.4	64.1	94.9	Forecast
	2030	75.4	64.5	96.2	Forecast
	2031	76.8	64.5	97.0	Forecast
	2032	78.3	64.2	97.1	Forecast
	2033	79.5	63.7	96.8	Forecast
	2034	80.4	63.0	96.1	Forecast
	2035	80.7	80.7	83.4	Forecast
	2036	80.7	74.5	83.4	Forecast
	2037	80.2	68.1	84.7	Forecast
	2038	79.6	63.2	86.2	Forecast
	1990	306.4			Actual
	1991	299.3			Actual
	1992	293.3			Actual
	1993	288.4			Actual
G20Male (Incidence)	1994	284.9			Actual
	1995	283.0			Actual
	1996	282.7			Actual
	1997	283.5			Actual
	1998	284.9			Actual
	1999	286.1			Actual
	2000	286.5			Actual

	2001	286.4			Actual
	2002	286.5			Actual
	2003	286.7			Actual
	2004	286.8			Actual
	2005	286.6			Actual
	2006	288.5			Actual
	2007	294.0			Actual
	2008	300.6			Actual
	2009	306.1			Actual
	2010	307.9			Actual
	2011	302.6			Actual
	2012	290.9			Actual
	2013	277.1			Actual
	2014	265.4			Actual
	2015	260.0			Actual
	2016	262.6			Actual
	2017	269.0			Actual
	2018	275.8			Actual
	2019	279.7			Actual
	2020	283.3			Actual
	2021	282.9			Actual
	2022	284.0			Actual
	2023	281.3			Actual
	2024	278.8	258.0	303.4	Forecast
	2025	276.5	260.0	309.0	Forecast
	2026	275.7	263.1	312.9	Forecast
	2027	277.4	265.5	315.4	Forecast
	2028	280.7	266.4	316.8	Forecast
	2029	284.5	265.6	317.2	Forecast
	2030	288.0	263.8	316.8	Forecast
	2031	290.5	261.7	315.6	Forecast
	2032	291.6	259.8	314.1	Forecast
	2033	291.4	258.4	312.8	Forecast
	2034	290.3	257.5	311.9	Forecast
	2035	288.7	275.6	281.9	Forecast
	2036	287.0	269.1	283.9	Forecast
	2037	285.6	262.4	289.0	Forecast
	2038	284.7	258.5	296.3	Forecast
G20 Female (Incidence)	1990	348.8			Actual
	1991	344.2			Actual
	1992	339.5			Actual
	1993	335.0			Actual
	1994	331.0			Actual
	1995	327.6			Actual

	1996	323.4			Actual
	1997	317.2			Actual
	1998	310.9			Actual
	1999	305.8			Actual
	2000	303.6			Actual
	2001	305.1			Actual
	2002	309.1			Actual
	2003	314.1			Actual
	2004	318.7			Actual
	2005	321.2			Actual
	2006	324.9			Actual
	2007	332.3			Actual
	2008	340.9			Actual
	2009	347.9			Actual
	2010	350.8			Actual
	2011	345.9			Actual
	2012	334.1			Actual
	2013	320.1			Actual
	2014	308.6			Actual
	2015	304.1			Actual
	2016	307.9			Actual
	2017	315.1			Actual
	2018	322.5			Actual
	2019	326.8			Actual
	2020	329.8			Actual
	2021	330.3			Actual
	2022	331.2			Actual
	2023	327.8			Actual
	2024	321.9	288.8	338.8	Forecast
	2025	315.6	290.9	344.2	Forecast
	2026	312.2	295.1	349.0	Forecast
	2027	311.7	299.4	353.3	Forecast
	2028	313.8	302.4	357.0	Forecast
	2029	317.5	303.6	359.7	Forecast
	2030	322.0	303.1	361.1	Forecast
	2031	326.3	301.4	360.9	Forecast
	2032	329.7	299.2	359.5	Forecast
	2033	331.6	296.8	357.3	Forecast
	2034	332.1	294.6	355.1	Forecast
	2035	331.2	318.4	325.4	Forecast
	2036	329.3	306.2	325.1	Forecast
	2037	327.0	296.1	328.3	Forecast
	2038	324.8	290.2	333.3	Forecast
G20 both	1990	326.9			Actual

(Incidence)	1991	321.1			Actual
	1992	315.8			Actual
	1993	311.2			Actual
	1994	307.4			Actual
	1995	304.8			Actual
	1996	302.4			Actual
	1997	299.7			Actual
	1998	297.1			Actual
	1999	295.1			Actual
	2000	294.2			Actual
	2001	295.0			Actual
	2002	297.1			Actual
	2003	299.9			Actual
	2004	302.4			Actual
	2005	303.6			Actual
	2006	306.4			Actual
	2007	312.9			Actual
	2008	320.5			Actual
	2009	326.9			Actual
	2010	329.2			Actual
	2011	324.1			Actual
	2012	312.4			Actual
	2013	298.4			Actual
	2014	286.8			Actual
	2015	281.8			Actual
	2016	285.0			Actual
	2017	291.9			Actual
	2018	299.0			Actual
	2019	303.2			Actual
	2020	306.4			Actual
	2021	306.5			Actual
	2022	307.5			Actual
	2023	304.4			Actual
	2024	301.5	278.2	320.8	Forecast
	2025	298.1	280.1	325.1	Forecast
	2026	296.7	283.1	328.5	Forecast
	2027	297.3	285.8	331.2	Forecast
	2028	299.5	287.0	333.4	Forecast
	2029	302.6	286.7	334.9	Forecast
	2030	305.8	285.2	335.3	Forecast
	2031	308.5	283.1	334.5	Forecast
	2032	310.2	281.1	333.0	Forecast
	2033	310.8	279.3	331.2	Forecast
	2034	310.2	277.8	329.8	Forecast

	2035	308.8	298.1	304.9	Forecast
	2036	307.0	290.0	306.1	Forecast
	2037	305.3	282.9	310.4	Forecast
	2038	303.8	278.9	315.7	Forecast
	1990	105.7			Actual
	1991	103.1			Actual
	1992	100.8			Actual
	1993	99.0			Actual
	1994	97.6			Actual
	1995	96.9			Actual
	1996	96.5			Actual
	1997	96.3			Actual
	1998	96.2			Actual
	1999	96.2			Actual
	2000	96.3			Actual
	2001	96.6			Actual
	2002	97.4			Actual
	2003	98.2			Actual
	2004	98.8			Actual
	2005	99.1			Actual
	2006	99.8			Actual
	2007	101.7			Actual
	2008	103.9			Actual
G20 Male	2009	105.7			Actual
(DALY)	2010	106.3			Actual
	2011	104.6			Actual
	2012	100.8			Actual
	2013	96.4			Actual
	2014	92.5			Actual
	2015	90.5			Actual
	2016	90.7			Actual
	2017	92.0			Actual
	2018	93.5			Actual
	2019	94.5			Actual
	2020	96.2			Actual
	2021	95.9			Actual
	2022	96.1			Actual
	2023	95.3			Actual
	2024	95.7	92.2	109.0	Forecast
	2025	96.1	91.9	110.3	Forecast
	2026	97.8	92.0	110.9	Forecast
	2027	99.0	91.5	110.4	Forecast
	2028	100.6	91.1	110.1	Forecast
	2029	101.1	90.3	109.4	Forecast

	2030	101.5	89.8	109.2	Forecast
	2031	101.0	89.1	108.9	Forecast
	2032	100.6	88.8	108.8	Forecast
	2033	99.8	88.4	108.6	Forecast
	2034	99.5	88.2	108.5	Forecast
	2035	99.0	94.6	96.9	Forecast
	2036	98.8	93.4	98.9	Forecast
	2037	98.5	92.8	102.8	Forecast
	2038	98.4	92.1	105.9	Forecast
G20 Female (DALY)	1990	126.3			Actual
	1991	124.1			Actual
	1992	122.1			Actual
	1993	120.1			Actual
	1994	118.4			Actual
	1995	117.1			Actual
	1996	115.3			Actual
	1997	112.9			Actual
	1998	110.3			Actual
	1999	108.1			Actual
	2000	107.2			Actual
	2001	107.9			Actual
	2002	109.7			Actual
	2003	112.0			Actual
	2004	114.1			Actual
	2005	115.2			Actual
	2006	116.3			Actual
	2007	118.6			Actual
	2008	121.1			Actual
	2009	123.4			Actual
	2010	124.4			Actual
	2011	123.2			Actual
	2012	120.3			Actual
	2013	116.8			Actual
	2014	113.8			Actual
	2015	112.5			Actual
	2016	113.1			Actual
	2017	114.1			Actual
	2018	115.3			Actual
	2019	116.1			Actual
	2020	117.3			Actual
	2021	117.4			Actual
	2022	117.5			Actual
	2023	116.4			Actual
	2024	115.3	105.0	122.4	Forecast

	2025	114.1	104.6	124.2	Forecast
	2026	113.5	104.9	125.6	Forecast
	2027	113.4	105.6	126.7	Forecast
	2028	113.7	106.5	127.6	Forecast
	2029	114.4	107.2	128.3	Forecast
	2030	115.3	107.5	128.9	Forecast
	2031	116.2	107.5	129.3	Forecast
	2032	117.0	107.3	129.5	Forecast
	2033	117.7	106.9	129.6	Forecast
	2034	118.2	106.4	129.4	Forecast
	2035	118.4	114.1	116.4	Forecast
	2036	118.4	111.2	117.0	Forecast
	2037	118.2	108.3	118.6	Forecast
	2038	117.9	106.2	120.5	Forecast
G20 Both (DALY)	1990	116.8			Actual
	1991	114.4			Actual
	1992	112.2			Actual
	1993	110.3			Actual
	1994	108.8			Actual
	1995	107.7			Actual
	1996	106.6			Actual
	1997	105.1			Actual
	1998	103.6			Actual
	1999	102.4			Actual
	2000	102.0			Actual
	2001	102.5			Actual
	2002	103.8			Actual
	2003	105.4			Actual
	2004	106.8			Actual
	2005	107.5			Actual
	2006	108.5			Actual
	2007	110.5			Actual
	2008	112.9			Actual
	2009	115.0			Actual
	2010	115.8			Actual
	2011	114.3			Actual
	2012	111.0			Actual
	2013	107.0			Actual
	2014	103.6			Actual
	2015	101.9			Actual
	2016	102.3			Actual
	2017	103.5			Actual
	2018	104.8			Actual
	2019	105.8			Actual

2020	107.1			Actual
2021	107.1			Actual
2022	107.2			Actual
2023	106.2			Actual
2024	105.7	98.5	114.8	Forecast
2025	105.4	98.5	116.4	Forecast
2026	105.5	98.9	117.5	Forecast
2027	105.9	99.4	118.3	Forecast
2028	106.6	99.8	118.7	Forecast
2029	107.4	100.0	118.9	Forecast
2030	108.2	99.8	119.1	Forecast
2031	108.8	99.4	119.1	Forecast
2032	109.2	98.8	119.0	Forecast
2033	109.4	98.3	118.8	Forecast
2034	109.4	97.8	118.6	Forecast
2035	109.2	104.6	106.9	Forecast
2036	108.9	102.6	108.2	Forecast
2037	108.6	100.6	110.3	Forecast
2038	108.2	99.2	112.7	Forecast

Supplemental Fig. 1. Age-group distributions of edentulism incidence and disability-adjusted life years in China and G20 countries, 1990–2023.

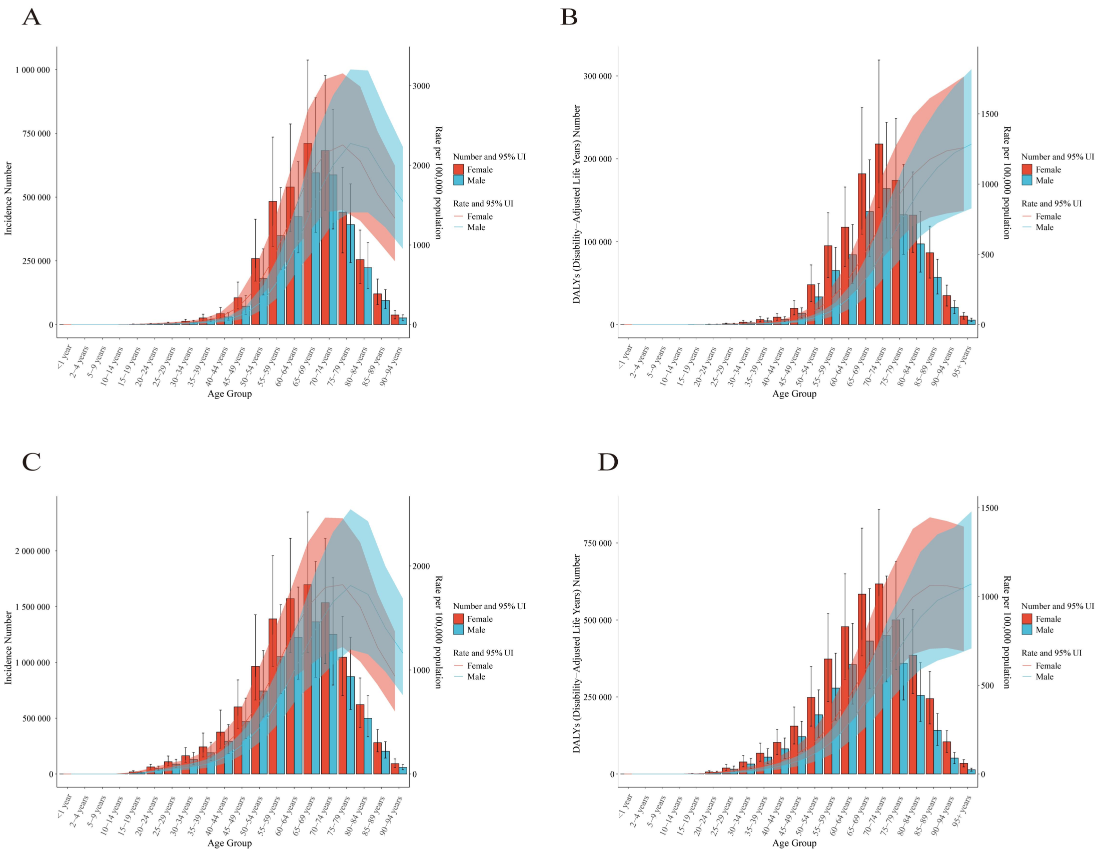

Supplement: Supplementary file 1 [file mmc1.pdf]
